# Mapping child growth failure across low- and middle-income countries

**DOI:** 10.1038/s41586-019-1878-8

**Published:** 2020-01-08

**Authors:** Local Burden of Disease Child Growth Failure Collaborators, Damaris K. Kinyoki, Aaron E. Osgood-Zimmerman, Brandon V. Pickering, Lauren E. Schaeffer, Laurie B. Marczak, Alice Lazzar-Atwood, Michael L. Collison, Nathaniel J. Henry, Zegeye Abebe, Abdu A. Adamu, Victor Adekanmbi, Keivan Ahmadi, Olufemi Ajumobi, Ayman Al-Eyadhy, Rajaa M. Al-Raddadi, Fares Alahdab, Mehran Alijanzadeh, Vahid Alipour, Khalid Altirkawi, Saeed Amini, Catalina Liliana Andrei, Carl Abelardo T. Antonio, Jalal Arabloo, Olatunde Aremu, Mehran Asadi-Aliabadi, Suleman Atique, Marcel Ausloos, Marco Avila, Ashish Awasthi, Beatriz Paulina Ayala Quintanilla, Samad Azari, Alaa Badawi, Till Winfried Bärnighausen, Quique Bassat, Kaleab Baye, Neeraj Bedi, Bayu Begashaw Bekele, Michelle L. Bell, Natalia V. Bhattacharjee, Krittika Bhattacharyya, Suraj Bhattarai, Zulfiqar A. Bhutta, Belete Biadgo, Boris Bikbov, Andrey Nikolaevich Briko, Gabrielle Britton, Roy Burstein, Zahid A. Butt, Josip Car, Carlos A. Castañeda-Orjuela, Franz Castro, Ester Cerin, Michael G. Chipeta, Dinh-Toi Chu, Michael A. Cork, Elizabeth A. Cromwell, Lucía Cuevas-Nasu, Lalit Dandona, Rakhi Dandona, Farah Daoud, Rajat Das Gupta, Nicole Davis Weaver, Diego De Leo, Jan-Walter De Neve, Kebede Deribe, Beruk Berhanu Desalegn, Aniruddha Deshpande, Melaku Desta, Daniel Diaz, Mesfin Tadese Dinberu, David Teye Doku, Manisha Dubey, Andre R. Durães, Laura Dwyer-Lindgren, Lucas Earl, Andem Effiong, Maysaa El Sayed Zaki, Maha El Tantawi, Ziad El-Khatib, Babak Eshrati, Mohammad Fareed, Andre Faro, Seyed-Mohammad Fereshtehnejad, Irina Filip, Florian Fischer, Nataliya A. Foigt, Morenike Oluwatoyin Folayan, Takeshi Fukumoto, Tsegaye Tewelde Gebrehiwot, Kebede Embaye Gezae, Alireza Ghajar, Paramjit Singh Gill, Philimon N. Gona, Sameer Vali Gopalani, Ayman Grada, Yuming Guo, Arvin Haj-Mirzaian, Arya Haj-Mirzaian, Jason B. Hall, Samer Hamidi, Andualem Henok, Bernardo Hernández Prado, Mario Herrero, Claudiu Herteliu, Chi Linh Hoang, Michael K. Hole, Naznin Hossain, Mehdi Hosseinzadeh, Guoqing Hu, Sheikh Mohammed Shariful Islam, Mihajlo Jakovljevic, Ravi Prakash Jha, Jost B. Jonas, Jacek Jerzy Jozwiak, Amaha Kahsay, Tanuj Kanchan, Manoochehr Karami, Amir Kasaeian, Yousef Saleh Khader, Ejaz Ahmad Khan, Mona M. Khater, Yun Jin Kim, Ruth W. Kimokoti, Adnan Kisa, Sonali Kochhar, Soewarta Kosen, Ai Koyanagi, Kewal Krishan, Barthelemy Kuate Defo, G. Anil Kumar, Manasi Kumar, Sheetal D. Lad, Faris Hasan Lami, Paul H. Lee, Aubrey J. Levine, Shanshan Li, Shai Linn, Rakesh Lodha, Hassan Magdy Abd El Razek, Muhammed Magdy Abd El Razek, Marek Majdan, Azeem Majeed, Reza Malekzadeh, Deborah Carvalho Malta, Abdullah A. Mamun, Mohammad Ali Mansournia, Francisco Rogerlândio Martins-Melo, Anthony Masaka, Benjamin Ballard Massenburg, Benjamin K. Mayala, Fabiola Mejia-Rodriguez, Mulugeta Melku, Walter Mendoza, George A. Mensah, Tomasz Miazgowski, Ted R. Miller, G. K. Mini, Erkin M. Mirrakhimov, Babak Moazen, Aso Mohammad Darwesh, Shafiu Mohammed, Farnam Mohebi, Ali H. Mokdad, Yoshan Moodley, Ghobad Moradi, Maziar Moradi-Lakeh, Paula Moraga, Shane Douglas Morrison, Jonathan F. Mosser, Seyyed Meysam Mousavi, Ulrich Otto Mueller, Christopher J. L. Murray, Ghulam Mustafa, Mehdi Naderi, Mohsen Naghavi, Farid Najafi, Vinay Nangia, Duduzile Edith Ndwandwe, Ionut Negoi, Josephine W. Ngunjiri, Huong Lan Thi Nguyen, Long Hoang Nguyen, Son Hoang Nguyen, Jing Nie, Chukwudi A. Nnaji, Jean Jacques Noubiap, Malihe Nourollahpour Shiadeh, Peter S. Nyasulu, Felix Akpojene Ogbo, Andrew T. Olagunju, Bolajoko Olubukunola Olusanya, Jacob Olusegun Olusanya, Eduardo Ortiz-Panozo, Stanislav S. Otstavnov, Mahesh P. A., Adrian Pana, Anamika Pandey, Sanghamitra Pati, Snehal T. Patil, George C. Patton, Norberto Perico, David M. Pigott, Meghdad Pirsaheb, Ellen G. Piwoz, Maarten J. Postma, Akram Pourshams, Swayam Prakash, Hedley Quintana, Amir Radfar, Alireza Rafiei, Vafa Rahimi-Movaghar, Rajesh Kumar Rai, Fatemeh Rajati, David Laith Rawaf, Salman Rawaf, Rahul Rawat, Giuseppe Remuzzi, Andre M. N. Renzaho, Carlos Rios-González, Leonardo Roever, Jennifer M. Ross, Ali Rostami, Nafis Sadat, Yahya Safari, Mahdi Safdarian, Amirhossein Sahebkar, Nasir Salam, Payman Salamati, Yahya Salimi, Hamideh Salimzadeh, Abdallah M. Samy, Benn Sartorius, Brijesh Sathian, Megan F. Schipp, David C. Schwebel, Anbissa Muleta Senbeta, Sadaf G. Sepanlou, Masood Ali Shaikh, Teresa Shamah Levy, Mohammadbagher Shamsi, Kiomars Sharafi, Rajesh Sharma, Aziz Sheikh, Apurba Shil, Diego Augusto Santos Silva, Jasvinder A. Singh, Dhirendra Narain Sinha, Moslem Soofi, Agus Sudaryanto, Mu’awiyyah Babale Sufiyan, Rafael Tabarés-Seisdedos, Birkneh Tilahun Tadesse, Mohamad-Hani Temsah, Abdullah Sulieman Terkawi, Zemenu Tadesse Tessema, Andrew L. Thorne-Lyman, Marcos Roberto Tovani-Palone, Bach Xuan Tran, Khanh Bao Tran, Irfan Ullah, Olalekan A. Uthman, Masoud Vaezghasemi, Afsane Vaezi, Pascual R. Valdez, John Vanderheide, Yousef Veisani, Francesco S. Violante, Vasily Vlassov, Giang Thu Vu, Linh Gia Vu, Yasir Waheed, Judd L. Walson, Yafeng Wang, Yuan-Pang Wang, Elizabeth N. Wangia, Andrea Werdecker, Gelin Xu, Tomohide Yamada, Engida Yisma, Naohiro Yonemoto, Mustafa Z. Younis, Mahmoud Yousefifard, Chuanhua Yu, Sojib Bin Zaman, Mohammad Zamani, Yunquan Zhang, Nicholas J. Kassebaum, Simon I. Hay

**Affiliations:** 1 Institute for Health Metrics and Evaluation, University of Washington, Seattle, WA USA; 2 Department of Health Metrics Sciences, School of Medicine, University of Washington, Seattle, WA USA; 3 Human Nutrition Department, University of Gondar, Gondar, Ethiopia; 4 Department of Global Health, Stellenbosch University, Cape Town, South Africa; 5 Cochrane South Africa, South African Medical Research Council, Cape Town, South Africa; 6 School of Medicine, Cardiff University, Cardiff, UK; 7 Lincoln Medical School, Universities of Nottingham & Lincoln, Lincoln, UK; 8 School of Community Health Sciences, University of Nevada, Reno, NV USA; 9 National Malaria Elimination Program, Federal Ministry of Health, Abuja, Nigeria; 10 Pediatric Intensive Care Unit, King Saud University, Riyadh, Saudi Arabia; 11 Department of Family and Community Medicine, King Abdulaziz University, Jeddah, Saudi Arabia; 12 Evidence Based Practice Center, Mayo Clinic Foundation for Medical Education and Research, Rochester, MN USA; 13 Qazvin University of Medical Sciences, Qazvin, Iran; 14 Health Economics Department, Iran University of Medical Sciences, Tehran, Iran; 15 Health Management and Economics Research Center, Iran University of Medical Sciences, Tehran, Iran; 16 King Saud University, Riyadh, Saudi Arabia; 17 Health Services Management Department, Arak University of Medical Sciences, Arak, Iran; 18 Carol Davila University of Medicine & Pharmacy, Bucharest, Romania; 19 Department of Health Policy & Administration, University of the Philippines Manila, Manila, The Philippines; 20 Department of Applied Social Sciences, Hong Kong Polytechnic University, Hong Kong, China; 21 School of Health Sciences, Birmingham City University, Birmingham, UK; 22 Preventive Medicine and Public Health Research Center, Iran University of Medical Sciences, Tehran, Iran; 23 Department of Health Informatics, University of Ha’il, Ha’il, Saudi Arabia; 24 School of Business, University of Leicester, Leicester, UK; 25 Department of Statistics and Econometrics, Bucharest University of Economic Studies, Bucharest, Romania; 26 Center for Research in Evaluation and Surveys, National Public Health Institute, Cuernavaca, Mexico; 27 Indian Institute of Public Health, Gandhinagar, India; 28 Public Health Foundation of India, Gurugram, India; 29 The Judith Lumley Centre, La Trobe University, Melbourne, Victoria Australia; 30 General Office for Research and Technological Transfer, Peruvian National Institute of Health, Lima, Peru; 31 Public Health Risk Sciences Division, Public Health Agency of Canada, Toronto, Ontario Canada; 32 Department of Nutritional Sciences, University of Toronto, Toronto, Ontario Canada; 33 Heidelberg Institute of Global Health (HIGH), Heidelberg University, Heidelberg, Germany; 34 T. H. Chan School of Public Health, Harvard University, Boston, MA USA; 35 Barcelona Institute for Global Health, University of Barcelona, Barcelona, Spain; 36 Catalan Institution for Research and Advanced Studies (ICREA), Barcelona, Spain; 37 Center for Food Science and Nutrition, Addis Ababa University, Addis Ababa, Ethiopia; 38 Department of Community Medicine, Gandhi Medical College Bhopal, Bhopal, India; 39 Jazan University, Jazan, Saudi Arabia; 40 Institute of Public Health, University of Gondar, Gondar, Ethiopia; 41 Public Health Department, Mizan-Tepi University, Teppi, Ethiopia; 42 School of Forestry and Environmental Studies, Yale University, New Haven, CT USA; 43 Department of Statistical and Computational Genomics, National Institute of Biomedical Genomics, Kalyani, India; 44 Department of Statistics, University of Calcutta, Kolkata, India; 45 Department of Global Health, Global Institute for Interdisciplinary Studies, Kathmandu, Nepal; 46 Centre for Global Child Health, University of Toronto, Toronto, Ontario Canada; 47 Centre of Excellence in Women and Child Health, Aga Khan University, Karachi, Pakistan; 48 Department of Clinical Chemistry, University of Gondar, Gondar, Ethiopia; 49 Istituto di Ricerche Farmacologiche Mario Negri IRCCS, Ranica, Italy; 50 Biomedical Technologies, Bauman Moscow State Technical University, Moscow, Russia; 51 Center for Neuroscience, Instituto de Investigaciones Científicas y Servicios de Alta Tecnología (INDICASAT AIP), Panama, Panama; 52 School of Public Health and Health Systems, University of Waterloo, Waterloo, Ontario Canada; 53 Al Shifa School of Public Health, Al Shifa Trust Eye Hospital, Rawalpindi, Pakistan; 54 Centre for Population Health Sciences, Nanyang Technological University, Singapore, Singapore; 55 Global Health Unit, Imperial College London, London, UK; 56 Colombian National Health Observatory, National Institute of Health, Bogota, Colombia; 57 Epidemiology and Public Health Evaluation Group, National University of Colombia, Bogota, Colombia; 58 Gorgas Memorial Institute for Health Studies, Panama, Panama; 59 Mary Mackillop Institute for Health Research, Australian Catholic University, Melbourne, Victoria Australia; 60 School of Public Health, University of Hong Kong, Hong Kong, China; 61 Big Data Institute, University of Oxford, Oxford, UK; 62 Faculty of Biology, Hanoi National University of Education, Hanoi, Vietnam; 63 Department of Epidemiology and Biostatistics, University of South Carolina, Columbia, SC USA; 64 James P. Grant School of Public Health, BRAC University, Dhaka, Bangladesh; 65 Australian Institute for Suicide Research and Prevention, Griffith University, Mount Gravatt, Queensland Australia; 66 School of Public Health, Addis Ababa University, Addis Ababa, Ethiopia; 67 Department of Global Health and Infection, Brighton and Sussex Medical School, Brighton, UK; 68 School of Nutrition, Food Science and Technology, Hawassa University, Hawassa, Ethiopia; 69 Department of Midwifery, Debre Markos University, Debre Markos, Ethiopia; 70 Faculty of Veterinary Medicine and Zootechnics, Autonomous University of Sinaloa, Culiacan Rosales, Mexico; 71 Center of Complexity Sciences, National Autonomous University of Mexico, Mexico City, Mexico; 72 Department of Midwifery, Debre Berhan University, Debre Berhan, Ethiopia; 73 Department of Population and Health, University of Cape Coast, Cape Coast, Ghana; 74 Faculty of Social Sciences, Health Sciences, University of Tampere, Tampere, Finland; 75 World Food Programme, New Delhi, India; 76 Medical Board, Roberto Santos General Hospital, Salvador, Brazil; 77 Department of Internal Medicine, Bahia School of Medicine and Public Health, Salvador, Brazil; 78 Clinical Epidemiology and Biostatistics, University of Newcastle, Newcastle, New South Wales Australia; 79 Department of Clinical Pathology, Mansoura University, Mansoura, Egypt; 80 Pediatric Dentistry and Dental Public Health, Alexandria University, Alexandria, Egypt; 81 Department of Public Health Sciences, Karolinska Institutet, Stockholm, Sweden; 82 World Health Programme, Université du Québec en Abitibi-Témiscamingue, Rouyn-Noranda, Quebec, Canada; 83 School of Public Health, Arak University of Medical Sciences, Arak, Iran; 84 Center of Communicable Disease Control, Ministry of Health and Medical Education, Tehran, Iran; 85 College of Medicine, Imam Muhammad Ibn Saud Islamic University, Riyadh, Saudi Arabia; 86 Department of Psychology, Federal University of Sergipe, Sao Cristovao, Brazil; 87 Department of Neurobiology, Care Sciences and Society, Karolinska Institutet, Stockholm, Sweden; 88 Division of Neurology, University of Ottawa, Ottawa, Ontario Canada; 89 Psychiatry Department, Kaiser Permanente, Fontana, CA USA; 90 Department of Health Sciences, A. T. Still University, Mesa, AZ USA; 91 Department of Population Health Medicine and Health Services Research, Bielefeld University, Bielefeld, Germany; 92 Laboratory of Population Aging, Institute of Gerontology, National Academy of Medical Sciences of Ukraine, Kyiv, Ukraine; 93 Department of Child Dental Health, Obafemi Awolowo University, Ile-Ife, Nigeria; 94 Gene Expression & Regulation Program, The Wistar Institute, Philadelphia, PA USA; 95 Department of Dermatology, Kobe University, Kobe, Japan; 96 Department of Epidemiology, Jimma University, Jimma, Ethiopia; 97 Department of Biostatistics, Mekelle University, Mekelle, Ethiopia; 98 Endocrinology and Metabolism Research Center (EMRC), Tehran University of Medical Sciences, Tehran, Iran; 99 Department of Medicine, Massachusetts General Hospital, Boston, MA USA; 100 Unit of Academic Primary Care, University of Warwick, Coventry, UK; 101 Nursing and Health Sciences Department, University of Massachusetts Boston, Boston, MA USA; 102 Department of Biostatistics and Epidemiology, University of Oklahoma, Oklahoma City, OK USA; 103 Department of Health and Social Affairs, Government of the Federated States of Micronesia, Palikir, Federated States of Micronesia; 104 Department of Dermatology, Boston University, Boston, MA USA; 105 School of Public Health and Preventive Medicine, Monash University, Melbourne, Victoria Australia; 106 Department of Epidemiology and Biostatistics, College of Public Health, Zhengzhou University, Zhengzhou, China; 107 Department of Pharmacology, Tehran University of Medical Sciences, Tehran, Iran; 108 Obesity Research Center, Shahid Beheshti University of Medical Sciences, Tehran, Iran; 109 Department of Radiology, Johns Hopkins University, Baltimore, MD USA; 110 School of Health and Environmental Studies, Hamdan Bin Mohammed Smart University, Dubai, United Arab Emirates; 111 Agriculture and Food, Commonwealth Scientific and Industrial Research Organisation, St Lucia, Queensland Australia; 112 Department of Statistics and Econometrics, Bucharest University of Economic Studies, Bucharest, Romania; 113 Center of Excellence in Behavioral Medicine, Nguyen Tat Thanh University, Ho Chi Minh City, Vietnam; 114 Department of Pediatrics, Dell Medical School, University of Texas Austin, Austin, TX USA; 115 Department of Pharmacology and Therapeutics, Dhaka Medical College, Dhaka, Bangladesh; 116 Department of Pharmacology, Bangladesh Industrial Gases Limited, Tangail, Bangladesh; 117 Department of Computer Engineering, Islamic Azad Univeristy, Tehran, Iran; 118 Computer Science Department, University of Human Development, Sulaimaniyah, Iraq; 119 Department of Epidemiology and Health Statistics, Central South University, Changsha, China; 120 Institute for Physical Activity and Nutrition, Deakin University, Burwood, Victoria, Australia; 121 Sydney Medical School, University of Sydney, Sydney, New South Wales Australia; 122 Department of Health Care and Public Health, Sechenov First Moscow State Medical University, Moscow, Russia; 123 Department of Community Medicine, Banaras Hindu University, Varanasi, India; 124 Department of Ophthalmology, Heidelberg University, Heidelberg, Germany; 125 Beijing Institute of Ophthalmology, Beijing Tongren Hospital, Beijing, China; 126 Department of Family Medicine and Public Health, University of Opole, Opole, Poland; 127 Department of Nutrition and Dietetics, Mekelle University, Mekelle, Ethiopia; 128 Department of Forensic Medicine and Toxicology, All India Institute of Medical Sciences, Jodhpur, India; 129 Department of Epidemiology, Hamadan University of Medical Sciences, Hamadan, Iran; 130 Pars Advanced and Minimally Invasive Medical Manners Research Center, Iran University of Medical Sciences Tehran, Tehran, Iran; 131 Hematology-Oncology and Stem Cell Transplantation Research Center, Tehran University of Medical Sciences, Tehran, Iran; 132 Department of Public Health, Jordan University of Science and Technology, Irbid, Jordan; 133 Epidemiology and Biostatistics Department, Health Services Academy, Islamabad, Pakistan; 134 Department of Medical Parasitology, Cairo University, Cairo, Egypt; 135 School of Medicine, Xiamen University Malaysia, Sepang, Malaysia; 136 Department of Nutrition, Simmons University, Boston, MA USA; 137 School of Health Sciences, Kristiania University College, Oslo, Norway; 138 Department of Global Health, University of Washington, Seattle, WA USA; 139 Department of Public Health, Erasmus University Medical Center, Rotterdam, The Netherlands; 140 Independent Consultant, Jakarta, Indonesia; 141 CIBERSAM, San Juan de Dios Sanitary Park, Sant Boi De Llobregat, Spain; 142 Department of Anthropology, Panjab University, Chandigarh, India; 143 Department of Social and Preventive Medicine, University of Montreal, Montreal, Quebec Canada; 144 Department of Demography, University of Montreal, Montreal, Quebec Canada; 145 Department of Psychiatry, University of Nairobi, Nairobi, Kenya; 146 Division of Psychology and Language Sciences, University College London, London, UK; 147 Department of Pediatrics, Post Graduate Institute of Medical Education and Research, Chandigarh, India; 148 Department of Community and Family Medicine, University of Baghdad, Baghdad, Iraq; 149 School of Nursing, Hong Kong Polytechnic University, Hong Kong, China; 150 School of Public Health, University of Haifa, Haifa, Israel; 151 Department of Paediatrics, All India Institute of Medical Sciences, Jodhpur, India; 152 Radiology Department, Mansoura Faculty of Medicine, Mansoura, Egypt; 153 Ophthalmology Department, Aswan Faculty of Medicine, Aswan, Egypt; 154 Department of Public Health, Trnava University, Trnava, Slovakia; 155 Department of Primary Care and Public Health, Imperial College London, London, UK; 156 Digestive Diseases Research Institute, Tehran University of Medical Sciences, Tehran, Iran; 157 Non-communicable Diseases Research Center, Shiraz University of Medical Sciences, Shiraz, Iran; 158 Department of Maternal and Child Nursing and Public Health, Federal University of Minas Gerais, Belo Horizonte, Brazil; 159 Institute for Social Science Research, The University of Queensland, Brisbane, Queensland Australia; 160 Department of Epidemiology and Biostatistics, Tehran University of Medical Sciences, Tehran, Iran; 161 Campus Caucaia, Federal Institute of Education, Science and Technology of Ceará, Caucaia, Brazil; 162 Public Health Department, Botho University-Botswana, Gaborone, Botswana; 163 Division of Plastic Surgery, University of Washington, Seattle, WA USA; 164 Research in Nutrition and Health, National Institute of Public Health, Cuernavaca, Mexico; 165 Peru Country Office, United Nations Population Fund (UNFPA), Lima, Peru; 166 Center for Translation Research and Implementation Science, National Institutes of Health, Bethesda, MD USA; 167 Department of Medicine, University of Cape Town, Cape Town, South Africa; 168 Department of Propedeutics of Internal Diseases & Arterial Hypertension, Pomeranian Medical University, Szczecin, Poland; 169 Pacific Institute for Research & Evaluation, Calverton, MD USA; 170 School of Public Health, Curtin University, Perth, Western Australia Australia; 171 Achutha Menon Centre for Health Science Studies, Sree Chitra Tirunal Institute for Medical Sciences and Technology, Trivandrum, India; 172 Global Institute of Public Health (GIPH), Ananthapuri Hospitals and Research Centre, Trivandrum, India; 173 Faculty of Internal Medicine, Kyrgyz State Medical Academy, Bishkek, Kyrgyzstan; 174 Department of Atherosclerosis and Coronary Heart Disease, National Center of Cardiology and Internal Disease, Bishkek, Kyrgyzstan; 175 Institute of Addiction Research (ISFF), Frankfurt University of Applied Sciences, Frankfurt, Germany; 176 Department of Information Technology, University of Human Development, Sulaymaniyah, Iraq; 177 Health Systems and Policy Research Unit, Ahmadu Bello University, Zaria, Nigeria; 178 Non-communicable Diseases Research Center, Tehran University of Medical Sciences, Tehran, Iran; 179 Department of Public Health Medicine, University of Kwazulu-Natal, Durban, South Africa; 180 Department of Epidemiology and Biostatistics, Kurdistan University of Medical Sciences, Sanandaj, Iran; 181 Social Determinants of Health Research Center, Kurdistan University of Medical Sciences, Sanandaj, Iran; 182 Department of Mathematical Sciences, University of Bath, Bath, UK; 183 Department of Surgery, University of Washington, Seattle, WA USA; 184 Department of Health Management and Economics, Tehran University of Medical Sciences, Tehran, Iran; 185 Health Management Research Center, Baqiyatallah Univeristy of Medical Sciences, Tehran, Iran; 186 Federal Institute for Population Research, Wiesbaden, Germany; 187 Center for Population and Health, Wiesbaden, Germany; 188 Department of Pediatric Medicine, Nishtar Medical University, Multan, Pakistan; 189 Department of Pediatrics & Pediatric Pulmonology, Institute of Mother & Child Care, Multan, Pakistan; 190 Clinical Research Development Centre, Kermanshah University of Medical Sciences, Kermanshah, Iran; 191 Department of Epidemiology & Biostatistics, Kermanshah University of Medical Sciences, Kermanshah, Iran; 192 Suraj Eye Institute, Nagpur, India; 193 General Surgery, Carol Davila University of Medicine & Pharmacy, Bucharest, Romania; 194 Department of Biological Sciences, University of Embu, Embu, Kenya; 195 Institute for Global Health Innovations, Duy Tan University, Hanoi, Vietnam; 196 Department of Sociology & Institute for Empirical Social Science Research, Xi’an Jiaotong University, Xi’an, China; 197 School of Public Health and Family Medicine, University of Cape Town, Cape Town, South Africa; 198 Mazandaran University of Medical Sciences, Sari, Iran; 199 Faculty of Medicine & Health Sciences, Stellenbosch University, Cape Town, South Africa; 200 UCIBIO, University of Porto, Porto, Portugal; 201 Department of Psychiatry and Behavioural Neurosciences, McMaster University, Hamilton, Ontario Canada; 202 Department of Psychiatry, University of Lagos, Lagos, Nigeria; 203 Centre for Healthy Start Initiative, Lagos, Nigeria; 204 Center for Population Health Research, National Institute of Public Health, Cuernavaca, Mexico; 205 School of Health and Welfare, Jönköping University, Jönköping, Sweden; 206 Laboratory of Public Health Indicators Analysis and Health Digitalization, Moscow Institute of Physics and Technology, Dolgoprudny, Russia; 207 Department of Project Management, National Research University Higher School of Economics, Moscow, Russia; 208 Department of Respiratory Medicine, Jagadguru Sri Shivarathreeswara Academy of Health Education and Research, Mysore, India; 209 Center for Health Outcomes & Evaluation, Bucharest, Romania; 210 Regional Medical Research Centre, Indian Council of Medical Research, Bhubaneswar, India; 211 Krishna Institute of Medical Sciences, Deemed University, Karad, India; 212 Department of Paediatrics, University of Melbourne, Melbourne, Victoria Australia; 213 Population Health, Murdoch Children’s Research Institute, Melbourne, Victoria Australia; 214 Istituto di Ricerche Farmacologiche Mario Negri IRCCS, Bergamo, Italy; 215 Research Center for Environmental Determinants of Health, Kermanshah University of Medical Sciences, Kermanshah, Iran; 216 Bill & Melinda Gates Foundation, Seattle, WA USA; 217 Department of Economics and Business, University of Groningen, Groningen, The Netherlands; 218 University Medical Center Groningen, University of Groningen, Groningen, The Netherlands; 219 Department of Nephrology, Sanjay Gandhi Postgraduate Institute of Medical Sciences, Lucknow, India; 220 College of Graduate Health Sciences, A. T. Still University, Mesa, AZ USA; 221 College of Medicine, University of Central Florida, Orlando, FL USA; 222 Molecular and Cell Biology Research Center, Mazandaran University of Medical Sciences, Sari, Iran; 223 Department of Immunology, Mazandaran University of Medical Sciences, Sari, Iran; 224 Sina Trauma and Surgery Research Center, Tehran University of Medical Sciences, Tehran, Iran; 225 Society for Health and Demographic Surveillance, Suri, India; 226 Department of Economics, University of Göttingen, Göttingen, Germany; 227 WHO Collaborating Centre for Public Health Education and Training, Imperial College London, London, UK; 228 University College London Hospitals, London, UK; 229 Academic Public Health, Public Health England, London, UK; 230 Department of Primary Care and Public Health, School of Public Health, Imperial College London, London, UK; 231 School of Social Sciences and Psychology, Western Sydney University, Penrith, New South Wales Australia; 232 Translational Health Research Institute, Western Sydney University, Penrith, New South Wales Australia; 233 Research Directorate, Nihon Gakko University, Fernando De La Mora, Paraguay; 234 Research Direction, Universidad Nacional de Caaguazú, Coronel Oviedo, Paraguay; 235 Department of Clinical Research, Federal University of Uberlândia, Uberlândia, Brazil; 236 Infectious Diseases and Tropical Medicine Research Center, Babol University of Medical Sciences, Babol, Iran; 237 Department of Neuroscience, Iran University of Medical Sciences, Tehran, Iran; 238 Neurogenic Inflammation Research Center, Mashhad University of Medical Sciences, Mashhad, Iran; 239 Halal Research Center of IRI, FDA, Tehran, Iran; 240 Department of Pathology, Al-Imam Mohammad Ibn Saud Islamic University, Riyadh, Saudi Arabia; 241 Social Development & Health Promotion Research Center, Kermanshah University of Medical Sciences, Kermanshah, Iran; 242 Department of Entomology, Ain Shams University, Cairo, Egypt; 243 Faculty of Infectious and Tropical Diseases, London School of Hygiene & Tropical Medicine, London, UK; 244 Surgery Department, Hamad Medical Corporation, Doha, Qatar; 245 Faculty of Health & Social Sciences, Bournemouth University, Bournemouth, UK; 246 Department of Psychology, University of Alabama at Birmingham, Birmingham, AL USA; 247 Department of Food Science and Nutrition, Jigjiga University, Jigjiga, Ethiopia; 248 Independent Consultant, Karachi, Pakistan; 249 Department of Sports Medicine & Rehabilitation, Kermanshah University of Medical Sciences, Kermanshah, Iran; 250 University School of Management and Entrepreneurship, Delhi Technological University, New Delhi, India; 251 Division of General Internal Medicine, Harvard University, Boston, MA USA; 252 Centre for Medical Informatics, University of Edinburgh, Edinburgh, UK; 253 Department of Public Health, Ben Gurion University of the Negev, Beersheva, Israel; 254 Department of Physical Education, Federal University of Santa Catarina, Florianopolis, Brazil; 255 Department of Medicine, University of Alabama at Birmingham, Birmingham, AL USA; 256 Department of Epidemiology, University of Alabama at Birmingham, Birmingham, AL USA; 257 Department of Epidemiology, School of Preventive Oncology, Patna, India; 258 Department of Epidemiology, Healis Sekhsaria Institute for Public Health, Mumbai, India; 259 Department of Nursing, Muhammadiyah University of Surakarta, Surakarta, Indonesia; 260 Department of Public Health, China Medical University, Taichung, Taiwan; 261 Department of Community Medicine, Ahmadu Bello University, Zaria, Nigeria; 262 Department of Medicine, University of Valencia, Valencia, Spain; 263 Carlos III Health Institute, Biomedical Research Networking Center for Mental Health Network (CIBERSAM), Madrid, Spain; 264 Department of Pediatrics, Hawassa University, Hawassa, Ethiopia; 265 International Vaccine Institute, Seoul, South Korea; 266 Department of Pediatrics, King Saud University, Riyadh, Saudi Arabia; 267 College of Medicine, Alfaisal University, Riyadh, Saudi Arabia; 268 Department of Anesthesiology, Perioperative, and Pain Medicine, Stanford University, Palo Alto, CA USA; 269 Department of Anesthesiology, King Fahad Medical City, Riyadh, Saudi Arabia; 270 Department of Epidemiology and Biostatistics, University of Gondar, Gondar, Ethiopia; 271 Department of International Health, Johns Hopkins University, Baltimore, MD USA; 272 Department of Pathology and Legal Medicine, University of São Paulo, Ribeirão Preto, Brazil; 273 Department of Health Economics, Hanoi Medical University, Hanoi, Vietnam; 274 Molecular Medicine and Pathology, University of Auckland, Auckland, New Zealand; 275 Clinical Hematology and Toxicology, Military Medical University, Hanoi, Vietnam; 276 Gomal Center of Biochemistry and Biotechnology, Gomal University, Dera Ismail Khan, Pakistan; 277 TB Culture Laboratory, Mufti Mehmood Memorial Teaching Hospital, Dera Ismail Khan, Pakistan; 278 Division of Health Sciences, University of Warwick, Coventry, UK; 279 Department of Epidemiology and Biostatistics, School of Public Health and Nutrition, Umeå University, Umeå, Sweden; 280 Department of Medical Mycology and Parasitology, Mazandaran University of Medical Sciences, Sari, Iran; 281 Argentine Society of Medicine, Ciudad De Buenos Aires, Argentina; 282 Velez Sarsfield Hospital, Buenos Aires, Argentina; 283 Psychosocial Injuries Research Center, Ilam University of Medical Sciences, Ilam, Iran; 284 Department of Medical and Surgical Sciences, University of Bologna, Bologna, Italy; 285 Occupational Health Unit, Sant’orsola Malpighi Hospital, Bologna, Italy; 286 Department of Health Care Administration and Economics, National Research University Higher School of Economics, Moscow, Russia; 287 Foundation University Medical College, Foundation University Islamabad, Islamabad, Pakistan; 288 Department of Epidemiology and Biostatistics, Wuhan University, Wuhan, China; 289 Department of Psychiatry, University of São Paulo, São Paulo, Brazil; 290 University of Nairobi, Nairobi, Kenya; 291 School of Medicine, Nanjing University, Nanjing, China; 292 Department of Diabetes and Metabolic Diseases, University of Tokyo, Tokyo, Japan; 293 School of Allied Health Sciences, Addis Ababa University, Addis Ababa, Ethiopia; 294 Department of Psychopharmacology, National Center of Neurology and Psychiatry, Tokyo, Japan; 295 Health Economics & Finance, Jackson State University, Jackson, MS USA; 296 School of Medicine, Tsinghua University, Beijing, China; 297 Prevention of Cardiovascular Disease Research Center, Shahid Beheshti University of Medical Sciences, Tehran, Iran; 298 Global Health Institute, Wuhan University, Wuhan, China; 299 Department of Medicine, School of Clinical Sciences at Monash Health, Monash University, Melbourne, Victoria Australia; 300 Maternal and Child Health Division, International Centre for Diarrhoeal Disease Research, Bangladesh, Dhaka Bangladesh; 301 Student Research Committee, Babol University of Medical Sciences, Babol, Iran; 302 School of Public Health, Wuhan University of Science and Technology, Wuhan, China; 303 Hubei Province Key Laboratory of Occupational Hazard Identification and Control, Wuhan University of Science and Technology, Wuhan, China; 304 Department of Anesthesiology & Pain Medicine, University of Washington, Seattle, WA USA

**Keywords:** Malnutrition, Risk factors, Signs and symptoms, Social sciences

## Abstract

Childhood malnutrition is associated with high morbidity and mortality globally^1^. Undernourished children are more likely to experience cognitive, physical, and metabolic developmental impairments that can lead to later cardiovascular disease, reduced intellectual ability and school attainment, and reduced economic productivity in adulthood^2^. Child growth failure (CGF), expressed as stunting, wasting, and underweight in children under five years of age (0–59 months), is a specific subset of undernutrition characterized by insufficient height or weight against age-specific growth reference standards^3–5^. The prevalence of stunting, wasting, or underweight in children under five is the proportion of children with a height-for-age, weight-for-height, or weight-for-age *z*-score, respectively, that is more than two standard deviations below the World Health Organization’s median growth reference standards for a healthy population^6^. Subnational estimates of CGF report substantial heterogeneity within countries, but are available primarily at the first administrative level (for example, states or provinces)^7^; the uneven geographical distribution of CGF has motivated further calls for assessments that can match the local scale of many public health programmes^8^. Building from our previous work mapping CGF in Africa^9^, here we provide the first, to our knowledge, mapped high-spatial-resolution estimates of CGF indicators from 2000 to 2017 across 105 low- and middle-income countries (LMICs), where 99% of affected children live^1^, aggregated to policy-relevant first and second (for example, districts or counties) administrative-level units and national levels. Despite remarkable declines over the study period, many LMICs remain far from the ambitious World Health Organization Global Nutrition Targets to reduce stunting by 40% and wasting to less than 5% by 2025. Large disparities in prevalence and progress exist across and within countries; our maps identify high-prevalence areas even within nations otherwise succeeding in reducing overall CGF prevalence. By highlighting where the highest-need populations reside, these geospatial estimates can support policy-makers in planning interventions that are adapted locally and in efficiently directing resources towards reducing CGF and its health implications.

## Abstract (web summary)

High-resolution subnational mapping of child growth failure indicators for 105 low- and middle-income countries between 2000 and 2017 shows that, despite considerable progress, substantial geographical inequalities still exist in some countries.

## Main

**Fig. 1:**
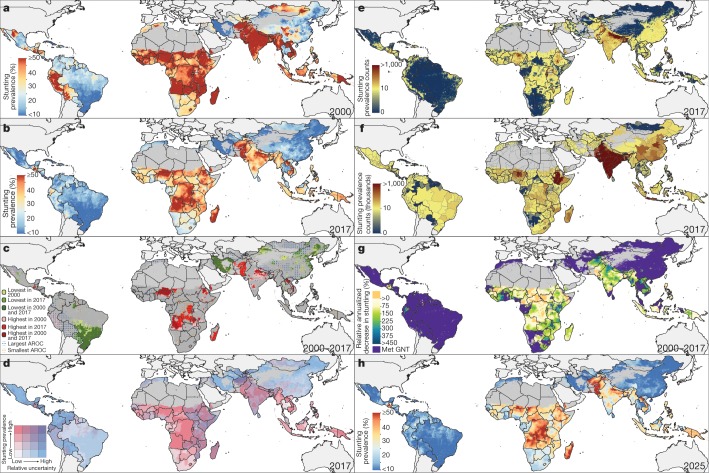
Prevalence of stunting in children under five in LMICs (2000–2017) and progress towards 2025. **a**, **b**, Prevalence of stunting in children under five at the 5 × 5-km resolution in 2000 (**a**) and 2017 (**b**). **c**, Overlapping population-weighted tenth and ninetieth percentiles (lowest and highest) of 5 × 5-km grid cells and AROC in stunting, 2000–2017. **d**, Overlapping population-weighted quartiles of stunting prevalence and relative 95% uncertainty in 2017. **e**, **f**, Number of children under five who were stunted, at the 5 × 5-km (**e**) and first-administrative-unit (**f**) levels. **g**, 2000–2017 annualized decrease in stunting prevalence relative to rates needed during 2017–2025 to meet the WHO GNT. **h**, Grid-cell-level predicted stunting prevalence in 2025. Maps were produced using ArcGIS Desktop 10.6. Interactive visualization tools are available at https://vizhub.healthdata.org/lbd/cgf.

**Extended Data Fig. 1:**
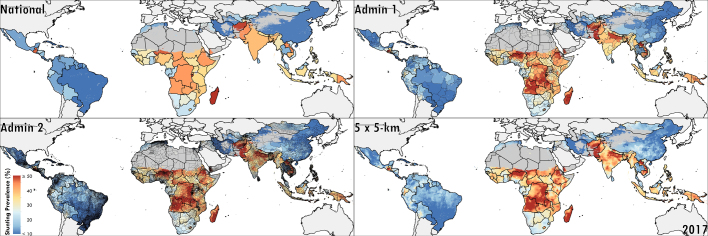
Prevalence of stunting in children under five in LMICs at administrative levels 0, 1, 2, and at 5 × 5-km resolution in 2017. Administrative level 0 are national-level estimates; administrative level 1 are first administrative-level (for example, states or provinces) estimates; administrative level 2 are second administrative-level (for example, districts or departments) estimates. Maps reflect administrative boundaries, land cover, lakes, and population; grey-coloured grid cells had fewer than ten people per 1 × 1-km grid cell and were classified as ‘barren or sparsely vegetated’^15,16,20,21,40–42^, or were not included in these analyses. Maps were produced using ArcGIS Desktop 10.6.

Despite improvements in nearly all LMICs, stunting remained the most widespread and prevalent indicator of CGF throughout the study period. Overall, estimated childhood stunting prevalence across LMICs decreased from 36.9% (95% uncertainty interval, 32.8–41.4%) in 2000 to 26.6% (21.5–32.4%) in 2017. Progress was particularly noticeable in Central America and the Caribbean, Andean South America, North Africa, and East Asia regions, and in some coastal central and western sub-Saharan African (SSA) countries, where most areas with estimated stunting prevalence of at least 50% in 2000 had reduced to 30% or less by 2017 (Fig. 1a, b). By 2017, zones with the highest prevalence of stunting primarily persisted throughout much of the SSA, Central and South Asia, and Oceania regions, where large areas had estimated levels of at least 40%, such as in the first administrative-level units of Nigeria’s Jigawa state (60.6% (51.5–69.7%)), Burundi’s Karuzi province (60.0% (51.4–67.5%)), India’s Uttar Pradesh state (49.0% (48.5–49.5%)), and Laos’s Houaphan province (58.3% (50.7–66.8%)) (Extended Data Fig. 1). In 2017, Guatemala (47.0% (40.2–54.6%)), Niger (47.5% (42.2–53.9%)), Burundi (54.2% (46.3–61.2%)), Madagascar (49.8% (43.2–57.2%)), Timor-Leste (49.8% (43.4–56.2%)), and Yemen (45.4% (38.8–51.5%)) had the highest national-level stunting prevalence.

**Extended Data Fig. 2:**
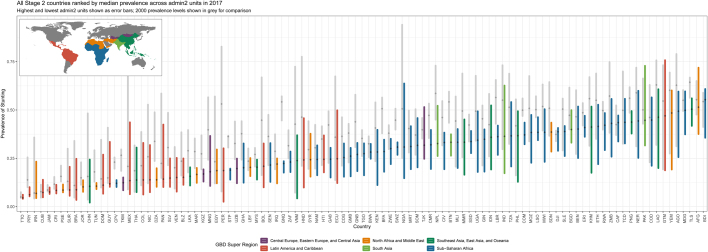
Geographical inequality in the prevalence of child stunting across 105 countries. The bars represent the range of stunting prevalence in children under five in the second administrative-level units in each country. Bars indicating the range in 2017 are coloured according to the regions defined by the Global Burden of Disease (GBD)^1^. Grey bars indicate the range in 2000. The graph was produced using R project v.3.5.1.

Even within the aforementioned regions where reductions were most evident, local-level estimates revealed communities in which levels still approached those seen in SSA and South Asia; areas in southern Mexico and central Ecuador had estimated stunting prevalence of at least 40%, and areas in western Mongolia reached at least 30%. Wide within-country disparities were apparent in several instances, indicating large areas left behind by the general pace of progress that require attention (Fig. 1a, b). Although most countries successfully reduced stunting prevalence, subnational inequalities (disparities between second administrative-level units (henceforth ‘units’)) remained widespread globally—especially evident in Vietnam, Honduras, Nigeria, and India (Extended Data Fig. 2). Among the top quintile of widest disparities, Indonesia experienced a twofold difference in stunting levels in 2017, ranging from 21.0% (16.2–27.0%) in Kota Yogyakarta regency (Yogyakarta province) to 51.5% (40.6–62.3%) in Sumba Barat regency (Nusa Tenggara Timur province). Stunting levels varied fourfold in Nigeria, ranging from 14.7% (9.1–21.0%) in Surulere Local Government Area (Lagos state) to 64.2% (54.2–74.6%) in Gagarawa Local Government Area (Jigawa state) in 2017.

Evaluated from estimates of population-weighted prevalence for areas with the highest and lowest estimated prevalence of stunting (ninetieth and tenth percentiles, respectively), locations in central Chad, Pakistan, and Afghanistan, in northeastern Angola, and throughout the Democratic Republic of the Congo and Madagascar had among the lowest annualized rates of change (AROC), indicating stagnation or increase over the study period (Fig. 1c); in 2017, these countries also had large geographical areas among the most highly prevalent for stunting. By contrast, areas scattered throughout Peru, northwestern Mexico, and eastern Nepal had among the highest stunting levels in 2000, but also the highest rates of decline; by 2017, many of these areas were subsequently no longer in the highest-prevalence decile.

The absolute number of children under five who were stunted was also unequally distributed (Fig. 1e, f), with a large proportion concentrated in a few nations in 2017; overall, 85.1% (84.4–85.7%) of all stunted children under five lived in Africa or Asia. Of the 176.1 million (151.6–203.3 million) children who were stunted in 2017, just over half (50.1% (48.5–52.0%)) lived in only four countries: India (51.5 million (47.7–55.3 million) children; 28.6% (27.1–30.4%) of global stunting), Pakistan (10.7 million (9.3–12.1 million); 6.8% (6.7–6.9%)), Nigeria (11.8 million (10.7–13.0 million); 6.6% (6.4–6.8%)), and China (16.2 million (14.0–18.5 million); 9.0% (9.1–8.9%)). Although China had a low prevalence of national stunting (10.8% (9.1–12.6%)) in 2017, the prevalence was high in India (39.3% (39.1–39.6%)), Pakistan (44.0% (38.4–49.9%)), and Nigeria (38.2% (34.5–42.0%)). Even with moderate levels of stunting (10 to <20%)^10^, these highly populous countries would substantially contribute to the global share owing to their population size, and reducing their levels would markedly decrease the number of stunted children.

**Fig. 2:**
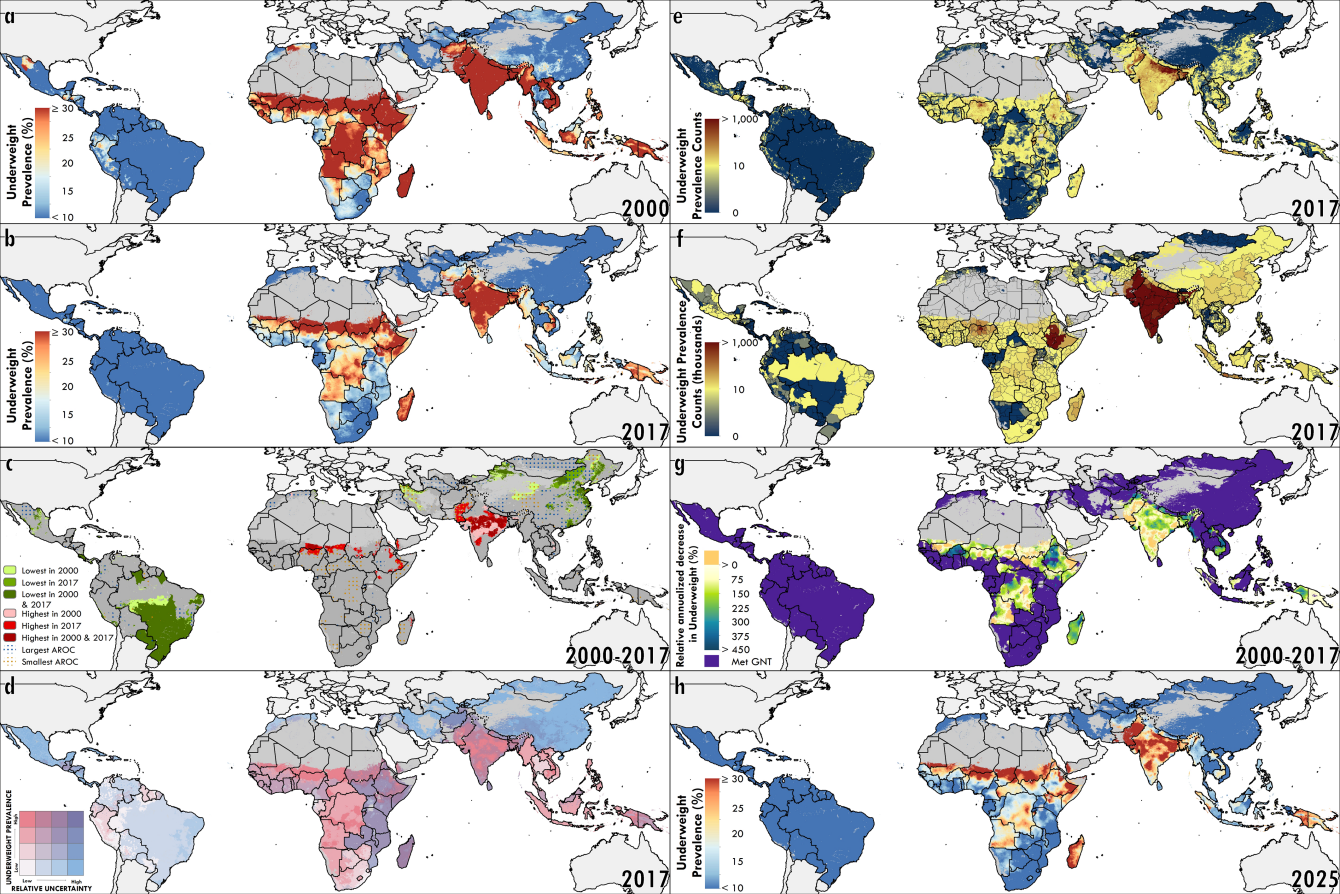
Prevalence of wasting in children under five in LMICs (2000–2017) and progress towards 2025. **a**, **b**, Prevalence of child wasting in children under five at the 5 × 5-km resolution in 2000 (**a**) and 2017 (**b**). **c**, Overlapping population-weighted tenth and ninetieth percentiles (lowest and highest) of 5 × 5-km grid cells and AROC in wasting, 2000–2017. **d**, Overlapping population-weighted quartiles of wasting prevalence and relative 95% uncertainty in 2017. **e**, **f**, Number of children under five affected by wasting, at the 5 × 5-km (**e**) and first-administrative-unit (**f**) levels. **g**, 2000–2017 annualized decrease in wasting prevalence relative to rates needed during 2017–2025 to meet the WHO GNT. **h**, Grid-cell-level predicted wasting prevalence in 2025. Maps were produced using ArcGIS Desktop 10.6. Interactive visualization tools are available at https://vizhub.healthdata.org/lbd/cgf.

**Extended Data Fig. 3:**
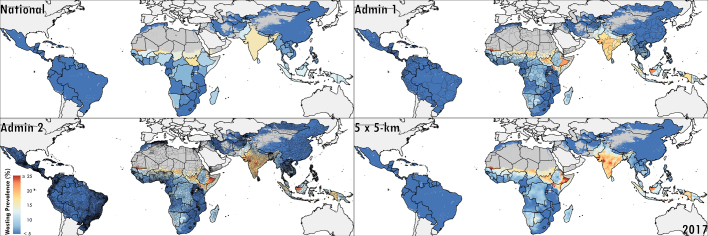
Prevalence of wasting in children under five in LMICs at administrative levels 0, 1, 2, and at 5 × 5-km resolution in 2017. Administrative levels are as described in Extended Data Fig. 1. Maps reflect administrative boundaries, land cover, lakes, and population; grey-coloured grid cells had fewer than ten people per 1 × 1-km grid cell and were classified as ‘barren or sparsely vegetated’^15,16,20,21,40–42^, or were not included in these analyses. Maps were produced using ArcGIS Desktop 10.6.

**Extended Data Fig. 4:**
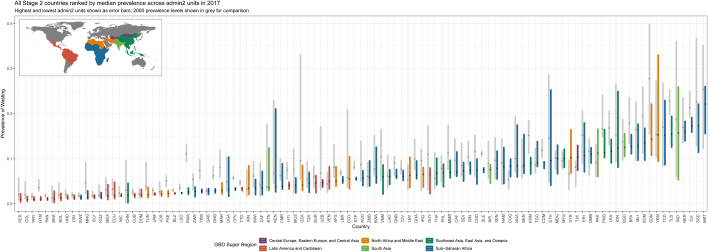
Geographical inequality in prevalence of child wasting across 105 countries. The bars represent the range of wasting prevalence in children under five in the second administrative-level units in each country. Bars indicating the range in 2017 are coloured according to their GBD-defined^1^ regions. Grey bars indicate the range in 2000. The graph was produced using R project v.3.5.1.

Childhood wasting was less widespread than stunting (Fig. 2a, b), affecting 8.4% (7.9–9.9%) of children under five in LMICs in 2000, and 6.4% (4.9–7.9%) by 2017. Wasting reached critical levels (at least 15%)^11^ nationally in 13 LMICs in 2000 and 7 LMICs in 2017, although only in Mauritania (20.7% (16.5–25.6%)) did all units exceed these levels (Extended Data Fig. 3). Critical wasting prevalence was concentrated in few areas across the globe in 2017, including the peri-Sahelian areas of countries stretching from Mauritania to Sudan, as well as areas in South Sudan, Ethiopia, Kenya, Somalia, Yemen, India, Pakistan, Bhutan, and Indonesia. Most LMICs reduced within-country disparities between their highest- and lowest-prevalence units between 2000 and 2017, most notably in Algeria, Uzbekistan, and Egypt (Extended Data Fig. 4). Even against a backdrop of national-level declines, however, broad within-country disparities in wasting remained in countries such as Indonesia, Ethiopia, Nigeria, and Kenya. An estimated ninefold difference in wasting prevalence occurred among Kenya’s units in 2017, ranging from 2.9% (1.6–4.9%) in Tetu constituency (Nyeri county) to 28.3% (20.2–37.3%) in Turkana East constituency (Turkana county); higher-resolution estimates reveal areas with a wasting prevalence of at least 25%. High-prevalence areas in 2000 typically remained within the highest population-weighted decile for wasting in 2017, including the units of Rabkona county (Unity state) in northern South Sudan (27.8% (19.8–37.6%) in 2000; 17.3% (8.8–21.9%) in 2017), the Tanout department (Zinder region) in southern Niger (21.6% (17.3–26.7%) in 2000; 16.5% (11.3–23.3%) in 2017), and Alor regency (Nusa Tenggara Timur province) in southeastern Indonesia (16.4% (9.6–25.8%) in 2000; 20.7% (12.8–30.3%) in 2017) (Fig. 2c).

The absolute number of children affected by wasting was unequal both across and within countries (Fig. 2e, f). Of the 58.3 million (47.6–70.7 million) children affected by wasting in 2017, 57.1% (52.7–61.6%) occurred in four of the most populous countries: India (26.1 million (23.1–29.0 million); 44.7% (41.0–48.6%) of global wasting), Pakistan (3.5 million (2.8–4.3 million); 6.0% (5.8–6.1%)), Bangladesh (1.8 million (1.2–2.4 million); 3.0% (2.6–3.4%)), and Indonesia (2.0 million (1.7–2.3 million); 3.4% (3.3–3.5%)). On the basis of standard thresholds^11^, these countries had serious levels of national wasting prevalence (10 to <15%), ranging from 12.2% (9.7–14.9%) in Pakistan to 15.7% (15.5–15.9%) in India, and all but Bangladesh had areas with estimated wasting levels above 20%; increased efforts, especially in densely populated areas with high prevalence and absolute numbers, could immensely reduce global child wasting.

**Extended Data Fig. 5:**
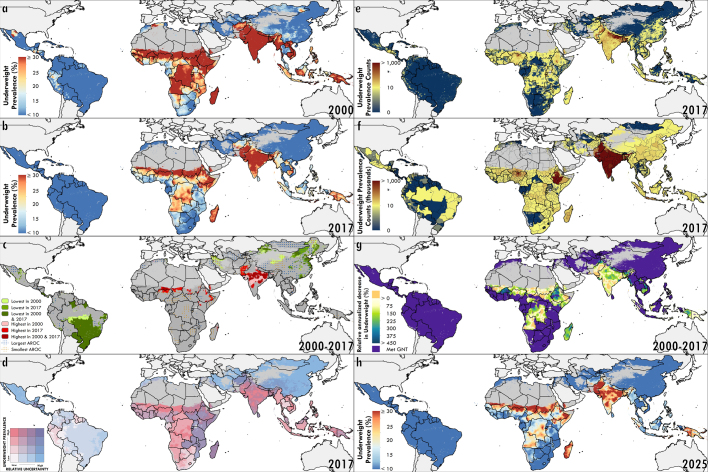
Prevalence of underweight in children under five in LMICs (2000–2017) and progress towards 2025. **a**, **b**, Prevalence of underweight in children under five at the 5 × 5-km resolution in 2000 (**a**) and 2017 (**b**). **c**, Overlapping population-weighted tenth and ninetieth percentiles (lowest and highest) of 5 × 5-km grid cells and AROC in underweight, 2000–2017. **d**, Overlapping population-weighted quartiles of underweight prevalence and relative 95% uncertainty in 2017. **e**, **f**, Number of underweight children under five, at the 5 × 5-km (**e**) and first-administrative-unit (**f**) levels. **g**, 2000–2017 annualized decrease in underweight prevalence relative to rates needed during 2017–2025 to meet WHO GNT. **h**, Grid-cell-level predicted underweight prevalence in 2025. Maps were produced using ArcGIS Desktop 10.6. Interactive visualization tools are available at https://vizhub.healthdata.org/lbd/cgf.

**Extended Data Fig. 6:**
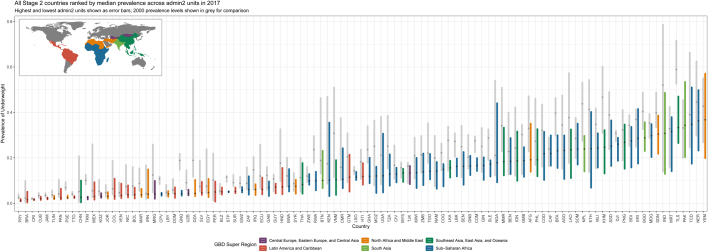
Geographical inequality in prevalence of child underweight across 105 countries. The bars represent the range of underweight prevalence in the second administrative-level units in each country. Bars indicating the range in 2017 are coloured according to their GBD-defined^1^ regions. Grey bars indicate the range in 2000. The graph was produced using R project v.3.5.1.

The prevalence of underweight—a composite indicator of stunting and wasting—followed the scattered pattern of high-stunting areas in SSA and spanning Central Asia to Oceania, and the high prevalence belt of wasting along the African Sahel (Extended Data Fig. 5a, b). Affecting 19.8% (17.3–22.7%) of children under five across LMICs in 2000 and 13.0% (10.4–16.0%) in 2017, reductions in underweight prevalence were most notable for countries in Central and South America, southern SSA, North Africa, and Southeast Asia. For example, by 2017, estimated underweight prevalence had decreased to less than or equal to 20% for nearly all areas in Namibia. By contrast, peri-Sahelian countries stretching from Mauritania to Somalia maintained an estimated underweight prevalence of at least 30% in many areas. Large geographical areas across Central and South Asia also maintained high prevalence of underweight during the study period; in particular, India, Pakistan, and Bangladesh sustained estimated prevalence of at least 30% in most locations. Although levels of child underweight had largely reduced since 2000, within-country disparities remained widespread; 71.4% (75 out of 105) of LMICs experienced at least a twofold difference across units in 2017 (Extended Data Fig. 6).

## Prospects for reaching 2025 targets

**Extended Data Fig. 7:**
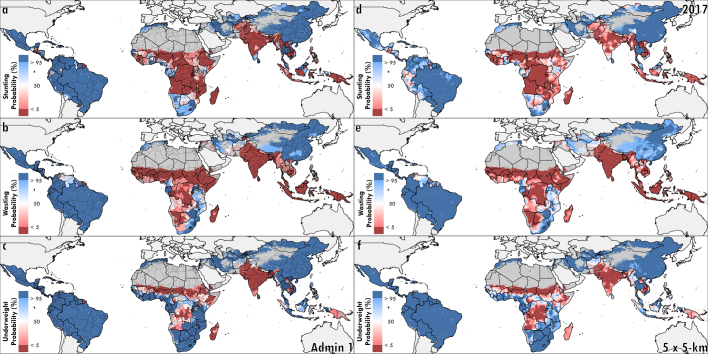
Probability that WHO GNT had been achieved in 2017 at the first administrative and 5 × 5-km grid-cell levels for stunting, wasting, and underweight. **a**–**f**, Probability of WHO GNT achievement in 2017 at the first administrative and 5 × 5-km levels for stunting (**a**, **d**), wasting (**b**, **e**), and underweight (**c**, **f**). Dark-blue and dark-red grid cells indicate >95% and <5% probability, respectively, of having met the WHO GNT in 2017. Given that there was no WHO GNT established for underweight, we based the underweight target on WHO GNT for stunting, as the conditions are similarly widespread and prevalent. Maps were produced using ArcGIS Desktop 10.6.

We estimate that broad areas across Central America and the Caribbean, South America, North Africa, and East Asia had high probability (>95%) of having already achieved targets for both stunting and wasting in 2017 (Extended Data Fig. 7). Exceptions to these regional patterns exist; areas with stagnated progress and less than 50% probability of having achieved the World Health Organization’s Global Nutrition Targets for 2025 (WHO GNTs) in 2017 were found throughout much of Guatemala and Ecuador for stunting and in southern Venezuela for wasting (Figs. 1g, 2g, Extended Data Fig. 7). Even within countries that had achieved targets, there remain areas with slow progress; locations in central Peru for stunting and southwestern South Africa for wasting had not achieved targets in 2017 (less than 5% probability)—nuances otherwise hidden by aggregated estimates. Owing to stagnation or increases in prevalence, broad areas in SSA and substantial portions across Central Asia, South Asia, and Oceania (for example, in the Democratic Republic of the Congo and Pakistan for stunting; in Yemen and Indonesia for wasting) require reversal of trends or acceleration of declines in order to meet international targets (Figs. 1g, 2g).

**Extended Data Fig. 8:**
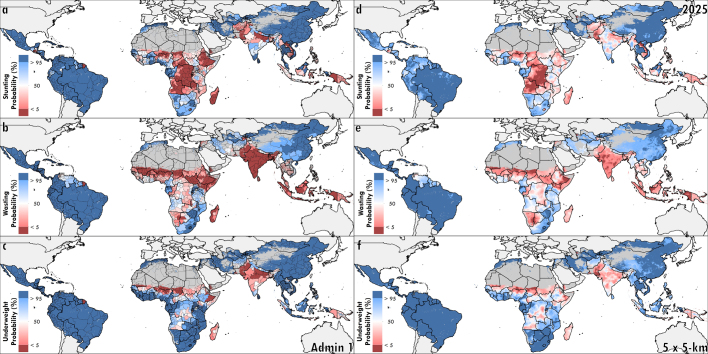
Probability of meeting WHO GNT in 2025 at the first administrative and 5 × 5-km grid-cell levels for stunting, wasting, and underweight. **a**–**f**, Probability of WHO GNT achievement in 2025 at the first administrative and 5 × 5-km levels for stunting (**a**, **d**), wasting (**b**, **e**), and underweight (**c**, **f**). Dark-blue and dark-red grid cells indicate >95% and <5% probability, respectively, of meeting WHO GNT in 2025. Given that there was no WHO GNT established for underweight, we based the underweight target on WHO GNT for stunting as the conditions are similarly widespread and prevalent. Maps were produced using ArcGIS Desktop 10.6.

Despite predicted improvements in AROC for 2017–2025, many highly affected countries are predicted to have areas that maintain estimated stunting levels of at least 40% or wasting levels of at least 15% in 2025 (Figs. 1h, 2h). Accounting for uncertainty in 2000–2017 AROC estimates, and with 2010 national-level estimates as a baseline for the 40% stunting reduction target, 44.8% (47 out of 105) of LMICs are estimated to nationally meet WHO GNT (>95% probability) for stunting by 2025 (Supplementary Table 13). At finer scales, 17.1% (*n* = 18) and 7.6% (*n* = 8) of LMICs will meet the stunting target in all first and second administrative-level units in 2025, respectively (Extended Data Fig. 8a, d, Supplementary Table 13). Similarly, 35.2% (*n* = 37) of LMICs are estimated to reduce to or maintain less than 5% wasting prevalence by 2025 (>95% probability) based on current trajectories (Supplementary Table 13). Fewer countries were estimated to meet wasting targets in all first administrative-level (16.2% (*n* = 17)) or second administrative-level (9.5% (*n* = 10)) units (Extended Data Fig. 8b, e, Supplementary Table 13). Only 26.7% (*n* = 28) of LMICs will meet national-level targets for both stunting and wasting by 2025, and only 4.8% (*n* = 5) will achieve both targets in all units (Supplementary Table 13).

## Discussion

Although commendable declines in CGF have occurred globally, this progress measured at a coarse scale conceals subnational and local underachievement and variation in achieving the WHO GNTs. Supporting conclusions in the Global Nutrition Report^12^, our results show that most LMICs will not reach WHO GNTs nationally, and even fewer will meet targets across subnational units. Our mapped results show broad heterogeneity across areas, and reveal hotspots of persistent CGF even within well-performing regions and countries, where increased and targeted efforts are needed. In 2017, one in four children under five across LMICs still suffered at least one dimension of CGF, and the largest numbers of affected children were often in specific within-country locations. Although the national prevalence of CGF was generally lower in Central America and the Caribbean, South American, and East Asian countries, there are communities in these regions in which levels of CGF remain as high as those in SSA and South Asia. Regardless of overall declines, many subnational areas across LMICs maintained high levels of CGF and require substantial acceleration of progress or reversal of increasing trends to meet nutrition targets and leave no populations behind.

To our knowledge, this study is the first to estimate CGF comprehensively across LMICs at a fine geospatial scale, providing a precision public health tool to support efficient targeting of local-level interventions to vulnerable populations. Although densely populated areas may have relatively low prevalence of CGF, the absolute number of affected children may still be high; thus, both relative and absolute estimates are important to determine where additional attention is needed. To achieve international goals, more concerted efforts are needed in areas with decreasing or stagnating trends, without diminishing support in areas that demonstrate progress nor contributing to increases in obesity. In future work, we plan to determine how to stratify our estimates of CGF by sex and age, assess the double burden of child undernutrition and overweight, analyse important maternal indicators that affect child nutritional status outcomes (such as anaemia), and continue to monitor progress towards the 2025 WHO GNTs. These mapped estimates enable decision-makers to visualize and compare subnational CGF and nutritional inequalities, and identify populations most in need of interventions^13^.

## Methods

### Overview

Building from our previous study of CGF in Africa^9^, we used Bayesian model-based geostatistics^14^—which leveraged geo-referenced survey data and environmental and socioeconomic covariates, and the assumption that points with similar covariate patterns and that are closer to one another in space and time would be expected to have similar patterns of CGF—to produce high-spatial-resolution estimates of the prevalence of stunting, wasting, and underweight among children under five across LMICs. Stunting, wasting, and underweight were defined as *z*-scores that were two or more standard deviations below the WHO healthy population reference median for length/height-for-age, weight-for-length/height, and weight-for-age, respectively, for age- and sex-specific curves^6^. Using an ensemble modelling framework that feeds into a Bayesian generalized linear model with a correlated space–time error, and 1,000 draws from the fitted posterior distribution, we generated estimates of annual prevalence for each indicator of CGF on a 5 × 5-km grid over 105 LMICs for each year from 2000 to 2017 and mapped results at administrative levels to provide relevant subnational information for policy planning and public health action. For this analysis, we compiled an extensive geo-positioned dataset, using data from 460 household surveys and reports representing 4.6 million children. To ensure comparability with national estimates and to facilitate benchmarking, these local-level estimates were calibrated to those produced by the Global Burden of Disease (GBD) Study 2017^1^, and were subsequently aggregated to the first administrative level (for example, states or provinces) and second administrative level (for example, districts or departments) in each LMIC. We also predict CGF prevalence for 2025 based on 2000–2017 trajectories and estimate the AROC required to meet the WHO GNTs by 2025. In addition, we estimate the 2017 absolute numbers of children under five affected by each CGF indicator in LMICs based on our prevalence estimates and the size of the populations of children under five^15,16^. Furthermore, we provide figures that demonstrate subnational disparities between each country’s second administrative-level units with the highest and lowest estimated prevalence for 2000 and 2017 (Extended Data Figs. 2, 4, 6). We re-estimate CGF prevalence for the 51 African countries included in our previous analysis^9^ using 28 additional surveys, and extend time trends to model each year from 2000 to 2017. Owing to these improvements in data availability and methodology, the estimates provided here supersede our previous modelling efforts.

**Extended Data Fig. 9:**
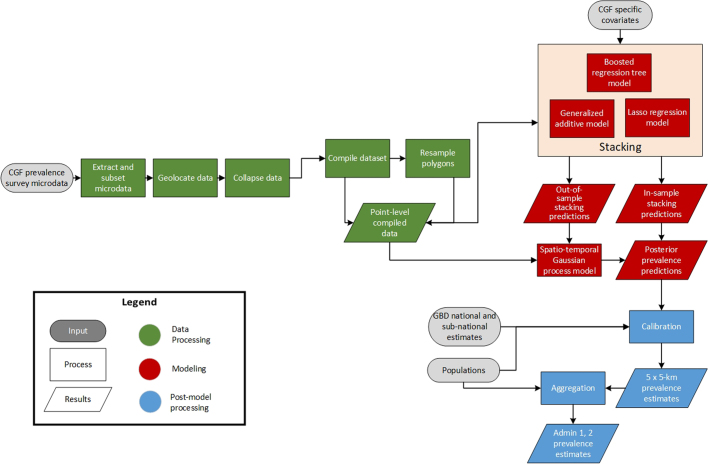
Flowchart of CGF prevalence modelling process. The process used to produce CGF prevalence estimates in LMICs involved three main parts. In the data-processing steps (green), data were identified, extracted, and prepared for use in the models. In the modelling phase (red), we used these data and covariates in stacked generalization ensemble models and spatiotemporal Gaussian process models for each CGF indicator. In post-processing (blue), we calibrated the prevalence estimates to match 2017 GBD study^1^ estimates and aggregated the estimates to the first- and second-administrative-level units in each country.

Countries were selected for inclusion in this study using the socio-demographic index (SDI)—a summary measure of development that combines education, fertility, and poverty, published in the GBD study^1^. The analyses reported here include countries in the low, low-middle, and middle SDI quintiles, with several exceptions (Supplementary Table 3). China, Iran, Libya, and Malaysia were included despite high-middle SDI status in order to create better geographical continuity. Albania and Moldova were excluded owing to geographical discontinuity with other included countries and lack of available survey data. We did not estimate for the island nations of American Samoa, Federated States of Micronesia, Fiji, Kiribati, Marshall Islands, North Korea, Samoa, Solomon Islands, or Tonga, where no available survey data could be sourced. The flowchart of our modelling process is provided in Extended Data Fig. 9.

### Surveys and child anthropometry data

We extracted individual-level height, weight, and age data for children under five from household survey series including the Demographic and Health Surveys (DHS), Multiple Indicator Cluster Surveys (MICS), Living Standards Measurement Study (LSMS), and Core Welfare Indicators Questionnaire (CWIQ), among other country-specific child health and nutrition surveys^7,17–19^ (Supplementary Tables 4, 5). Included in our models were 460 geo-referenced household surveys and reports from 105 countries representing approximately 4.6 million children under five. Each individual child record was associated with a cluster, a group of neighbouring households or a ‘village’ that acts as a primary sampling unit. Some surveys included geographical coordinates or precise place names for each cluster within that survey (138,938 clusters for stunting, 144,460 for wasting, and 147,624 for underweight). In the absence of geographical coordinates for each cluster, we assigned data to the smallest available administrative areal unit in the survey (termed a ‘polygon’) while correcting for the survey sample design (16,554 polygons for stunting, 18,833 for wasting, and 19,564 for underweight). Boundary information for these administrative units was obtained as shapefiles either directly from the surveys or by matching to shapefiles in the Global Administrative Unit Layers (GAUL)^20^ or the Database of Global Administrative Areas (GADM)^21^. In select cases, shapefiles provided by the survey administrator were used, or custom shapefiles were created based on survey documentation. These areal data were resampled to point locations using a population-weighted sampling approach over the relevant areal unit with the number of locations set proportionally to the number of grid cells in the area and the total weights of all the resampled points summing to one^16^.

Select data sources were excluded for the following reasons: missing survey weights for areal data, missing sex variable, insufficient age granularity (in months) for calculations of length/height-for-age *z*-scores and weight-for-age *z*-scores in children ages 0–2 years, incomplete sampling (for example, only children ages 0–3 years measured), or untrustworthy data (as determined by the survey administrator or by inspection). We excluded data for children for whom we could not compute age in both months and weeks. Children with height values ≤0 cm or ≥180 cm, and/or with weight values ≤0 kg or ≥45 kg were also excluded from the study. We also excluded data that were considered outliers according to the 2006 WHO Child Growth Standards recommended range values, which were values <−6 or >6 length/height-for-age *z*-score for stunting, <−5 or >5 weight-for-length/height *z*-score for wasting, and <−6 or >5 weight-for-age *z*-score for underweight^3,4^. Details on the survey data excluded for each country are provided in Supplementary Table 6. Data availability plots for all the CGF indicators by country, type, and year are included in Supplementary Figs. 2–16.

### Child anthropometry

Using the height, weight, age, and sex data for each individual, height-for-age, weight-for-height, and weight-for-age *z*-scores were calculated using the age-, sex-, and indicator-specific LMS (lambda-mu-sigma) values from the 2006 WHO Child Growth Standards^3,4^. The LMS methodology allows for Gaussian *z*-score calculations and comparisons to be applied to skewed, non-Gaussian distributions^22^. We classified stunting, wasting, or underweight if the height/length-for-age, weight-for-height/length, or weight-for-age, respectively, was more than two standard deviations (*z*-scores) below the WHO growth reference population^6^. These individual-level data observations were then collapsed to cluster-level totals for the number of children sampled and total number of children under five affected by stunting, wasting, or underweight.

### Temporal resolution

We estimated the prevalence of stunting, wasting, and underweight annually from 2000 to 2017 using a model that allows us to account for data points measured across survey years. As such, the model would also allow us to predict at monthly or finer temporal resolutions; however, we are limited both computationally and by the temporal resolution of the covariates.

### Seasonality adjustment

Owing to the acute nature of wasting and its relative temporal transience, wasting data were pre-processed to account for seasonality within each year of observation. Across LMICs, large proportions of the population live in rural areas and have livelihoods that rely on agriculture and livestock. Seasonality affects the availability of and access to food, sometimes owing to natural disasters or climate events (for example, floods, monsoons, or droughts) that vary by season. Generalized additive models were fit to wasting data across time using the month of interview and a country-level fixed effect as the explanatory variables, and the wasting *z*-score as the response. A 12-month periodic spline for the interview month was used, as well as a spline that smoothed across the whole duration of the dataset. Once the models were fit, individual weight-for-height/length *z*-score observations were adjusted so that each measurement was consistent with a day that represented a mean day in the periodic spline. The seasonality adjustment had relatively little effect on the raw data^9^.

### Spatial covariates

To leverage strength from locations with observations to the entire spatiotemporal domain, we compiled several 5 × 5-km raster layers of possible socioeconomic and environmental correlates of CGF in the 105 LMICs (Supplementary Table 7, Supplementary Fig. 17). Covariates were selected based on their potential to be predictive for the set of CGF indicators, after reviewing literature on evidence and plausible hypotheses as to their influence. Acquisition of temporally dynamic datasets, where possible, was prioritized to best match our observations and thus predict the changing dynamics of the CGF indicators. Of the twelve covariates included, eight were temporally dynamic and were reformatted as a synoptic mean over each estimation period or as a mid-period year estimate: these covariates included average daily mean rainfall (precipitation), average daily mean temperature, enhanced vegetation index, fertility, malaria incidence, educational attainment in women of reproductive age (15–49 years old), population, and urbanicity. The remaining four covariate layers were static throughout the study period and were applied uniformly across all modelling years; growing season length, irrigation, nutritional yield for vitamin A, and travel time to nearest settlement of >50,000 inhabitants.

To select covariates and capture possible nonlinear effects and complex interactions between them, an ensemble covariate modelling method was implemented^23^. For each region, three sub-models were fit to our dataset using all of our covariate data as explanatory predictors; these sub-models were: generalized additive models, boosted regression trees, and lasso regression. Each sub-model was fit using fivefold cross-validation to avoid overfitting, and the out-of-sample predictions from across the five holdouts were compiled into a single comprehensive set of predictions from that model. In addition, the same sub-models were run using 100% of the data, and a full set of in-sample predictions were created. The three sets of out-of-sample sub-model predictions were fed into the full geostatistical model^14^ as the explanatory covariates when performing the model fit. The in-sample predictions from the sub-models were used as the covariates when generating predictions using the fitted full geostatistical model. A recent study demonstrated that this ensemble approach can improve predictive validity by up to 25% over an individual model^23^.

### Geostatistical model analysis

**Extended Data Fig. 10:**
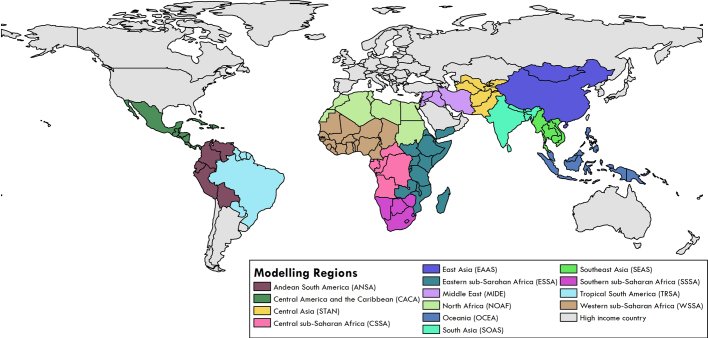
Modelling regions. Modelling regions^24^ were based on geographical and SDI regions from the GBD study^1^, defined as: Andean South America, Central America and the Caribbean, central SSA, East Asia, eastern SSA, Middle East, North Africa, Oceania, Southeast Asia, South Asia, southern SSA, Central Asia, Tropical South America, and western SSA. ‘High income country’ refers to regions not included in our models owing to high-middle or a high SDI. The map was produced using ArcGIS Desktop 10.6.

Binomial count data were modelled within a Bayesian hierarchical modelling framework using a logit link function and a spatially and temporally explicit hierarchical generalized linear regression model to fit prevalence of each of our indicators in 14 regions^24^ of LMICs (North Africa, western SSA, central SSA, eastern SSA, southern SSA, Middle East, Central Asia, East Asia, South Asia, Southeast Asia, Oceania, Central America and the Caribbean, Andean South America, and Tropical South America; see Extended Data Fig. 10). For each region, we explicitly wrote the hierarchy that defines our Bayesian model.

For each binomial CGF indicator, we modelled the average number of children with stunting, wasting, or who were underweight in each survey cluster, *d*. Survey clusters are precisely located by their GPS coordinates and year of observation, which we map to a spatial raster location, *i*, at time, *t*. We observed the number of children reported to be stunted, wasted, or underweight, respectively, as binomial count data, *C*_*d*_, among an observed sample size, *N*_*d*_. As we may have observed several data clusters within a given location, *i*, at time, *t*, we refer to the probability of stunting, wasting, or underweight, *p*, within a given cluster, *d*, by its indexed location, *i*, and time, *t*, as *p*_*i*(*d*),*t*(*d*)_.

$$\begin{array}{l}{C}_{d}|{p}_{i(d),t(d)},\,{N}_{d}\sim {\rm{B}}{\rm{i}}{\rm{n}}{\rm{o}}{\rm{m}}{\rm{i}}{\rm{a}}{\rm{l}}({p}_{i(d),t(d)},\,{N}_{d})\,{\rm{\forall }}\,{\rm{o}}{\rm{b}}{\rm{s}}{\rm{e}}{\rm{r}}{\rm{v}}{\rm{e}}{\rm{d}}\,{\rm{c}}{\rm{l}}{\rm{u}}{\rm{s}}{\rm{t}}{\rm{e}}{\rm{r}}{\rm{s}}\,d\end{array}$$

$$\begin{array}{l}{\rm{l}}{\rm{o}}{\rm{g}}{\rm{i}}{\rm{t}}({p}_{i,t})=\,{\beta }_{0}+{{\bf{X}}}_{i,t}{\boldsymbol{\beta }}+{Z}_{i,t}+{{\epsilon }}_{{\rm{c}}{\rm{t}}{\rm{r}}(i)}+{{\epsilon }}_{i,t}+{Z}_{i,t}\,{\rm{\forall }}\\ i\in {\rm{s}}{\rm{p}}{\rm{a}}{\rm{t}}{\rm{i}}{\rm{a}}{\rm{l}}\,{\rm{d}}{\rm{o}}{\rm{m}}{\rm{a}}{\rm{i}}{\rm{n}}\,{\rm{\forall }}\,t\in {\rm{t}}{\rm{i}}{\rm{m}}{\rm{e}}\,{\rm{d}}{\rm{o}}{\rm{m}}{\rm{a}}{\rm{i}}{\rm{n}}\end{array}$$

$$\begin{array}{l}\mathop{\sum }\limits_{h=1}^{3}{\beta }_{h}\,=1\\ {{\epsilon }}_{{\rm{c}}{\rm{t}}{\rm{r}}}\sim {\rm{i}}{\rm{i}}{\rm{d}}\,{\rm{N}}{\rm{o}}{\rm{r}}{\rm{m}}{\rm{a}}{\rm{l}}(0,\,{\gamma }^{2})\\ {{\epsilon }}_{i,t}\sim {\rm{i}}{\rm{i}}{\rm{d}}\,{\rm{N}}{\rm{o}}{\rm{r}}{\rm{m}}{\rm{a}}{\rm{l}}(0,\,{\sigma }^{2})\\ {\bf{Z}}\sim {\rm{G}}{\rm{P}}(0,{\Sigma }^{{\rm{s}}{\rm{p}}{\rm{a}}{\rm{c}}{\rm{e}}}\otimes {\Sigma }^{{\rm{t}}{\rm{i}}{\rm{m}}{\rm{e}}})\\ {\Sigma }^{{\rm{s}}{\rm{p}}{\rm{a}}{\rm{c}}{\rm{e}}}=\,\frac{{\omega }^{2}}{\Gamma (\nu ){2}^{v-1}}\times {(\kappa D)}^{\nu }\times {{\rm K}}_{\nu }(\kappa D)\\ {\Sigma }_{j,\,k}^{{\rm{t}}{\rm{i}}{\rm{m}}{\rm{e}}\,}={\rho }^{|k-j|}\end{array}$$

For indices *d*, *i*, and *t*, *(index) is the value of * at that index. The probabilities, *p*_*i*,*t*_, represent both the annual prevalence at the space–time location and the probability that an individual child was afflicted with the risk factor given that they lived at that particular location. The annual prevalence, *p*_*i*,*t*_, of each indicator was modelled as a linear combination of the three sub-models (generalized additive model, boosted regression trees, and lasso regression), rasterized covariate values, **X**_*i*,*t*_, a correlated spatiotemporal error term, *Z*_*i*,*t*_, and country random effects, *ϵ*_ctr(*i*)_, with one unstructured country random effect fit for each country in the modelling region and all *ϵ*_ctr_ sharing a common variance parameter, *γ*^2^, and an independent nugget effect, *ϵ*_*i*,*t*_, with variance parameter, *σ*^2^. Coefficients in *β*_*h*_ in the three sub-models *h* = 1, 2, 3 represent their respective predictive weighting in the mean logit link, while the joint error term, *Z*_*i*,*t*_, accounts for residual spatiotemporal autocorrelation between individual data points that remains after accounting for the predictive effect of the sub-model covariates, the country-level random effect, *ϵ*_ctr(*i*)_, and the nugget independent error term, *ϵ*_*i*,*t*_. The residuals, *Z*_*i*,*t*_, are modelled as a three-dimensional Gaussian process (GP) in space–time centred at zero and with a covariance matrix constructed from a Kronecker product of spatial and temporal covariance kernels. The spatial covariance, Σ^space^, is modelled using an isotropic and stationary Matérn function^25^, and temporal covariance, Σ^time^, as an annual autoregressive (AR1) function over the 18 years represented in the model. In the stationary Matérn function, Γ is the gamma function, *Κ*_*v*_ is the modified Bessel function of order *v* > 0, *κ* > 0 is a scaling parameter, *D* denotes the Euclidean distance, and *ω*^2^ is the marginal variance. The scaling parameter, *κ*, is defined to be $\kappa =\sqrt{8v}/\delta$ in which *δ* is a range parameter (which is about the distance where the covariance function approaches 0.1) and *v* is a scaling constant, which is set to 2 rather than fit from the data^26,27^. This parameter is difficult to reliably fit, as documented by many other analyses^26,28,29^ that set this to 2. The number of rows and the number of columns of the spatial Matérn covariance matrix are both equal to the number of spatial mesh points for a given modelling region. In the AR1 function, *ρ* is the autocorrelation function (ACF), and *k* and *j* are points in the time series where |*k* − *j*| defines the lag. The number of rows and the number of columns of the AR1 covariance matrix are both equal to the number of temporal mesh points (18). The number of rows and the number of columns of the  space–time covariance matrix, Σ^space^ ⊗ Σ^time^, for a given modelling region are both equal to: (the number of spatial mesh points × the number of temporal mesh points).

This approach leveraged the residual correlation structure of the data to more accurately predict prevalence estimates for locations with no data, while also propagating the dependence in the data through to uncertainty estimates^14^. The posterior distributions were fit using computationally efficient and accurate approximations in R-INLA^30,31^ (integrated nested Laplace approximation) with the stochastic partial differential equations (SPDE)^27^ approximation to the Gaussian process residuals using R project v.3.5.1. The SPDE approach using INLA has been demonstrated elsewhere, including the estimation of health indicators, particulate air matter, and population age structure^9,32–35^. Uncertainty intervals were generated from 1,000 draws (that is, statistically plausible candidate maps)^36^ created from the posterior-estimated distributions of modelled parameters. Further details on model and estimation processes are provided in the Supplementary Information.

### Post estimation

To leverage national-level data included in the 2017 GBD study^1^ that were not within the scope of our current geospatial modelling framework, and to ensure alignment between these estimates and GBD national-level and subnational estimates, we performed a post hoc calibration to the mean of the 1,000 draws. We calculated population-weighted aggregations to the GBD estimate level, which was either at the national or first administrative level, and compared these estimates to our corresponding year estimates from 2000 to 2017. We defined the calibration factor to be the ratio between the GBD estimates and our current estimates for each year from 2000 to 2017. For some selected countries where GBD estimates were at the first administrative level, the calibration factors were also calculated at the lowest available subnational level. These countries included Brazil, China, Ethiopia, India, Indonesia, Iran, Mexico, and South Africa. Finally, we multiplied each of our estimates in a country-year (or first-administrative-year) by its associated factor. This ensures consistency between our geospatial estimates and those of the 2017 GBD^1^, while preserving our estimated within-country geospatial and temporal variation. To transform grid-cell-level estimates into a range of information useful to a wide constituency of potential users, these estimates were aggregated at first and second administrative-level units specific to each country and at national levels using conditional simulation^37^.

Although the models can predict all locations covered by available raster covariates, all final model outputs for which land cover was classified as ‘barren or sparsely vegetated’ on the basis of the most recently available Moderate Resolution Imaging Spectroradiometer (MODIS) satellite data (2013) were masked^38^. Areas where the total population density was less than ten individuals per 1 × 1-km grid cell were also masked in the final outputs.

### Model validation

We assessed the predictive performance of the models using fivefold out-of-sample cross-validation strategies and found that our prevalence estimates closely matched the survey data. To offer a more stringent analysis by respecting some of the spatial correlation in the data, holdout sets were created by combining sets of data at different spatial resolutions (for example, first administrative level). Validation was performed by calculating bias (mean error), variance (root mean square error), 95% data coverage within prediction intervals, and correlation between observed data and predictions. All validation metrics were calculated on the out-of-sample predictions from the fivefold cross-validation. Furthermore, measures of spatial and temporal autocorrelation pre- and post-modelling were examined to verify correct recognition, fitting, and accounting for the complex spatiotemporal correlation structure in the data. All validation procedures and corresponding results are included in Supplementary Tables 14–22 and Supplementary Figs. 24–41.

### Projections

To compare our estimated rates of improvement in CGF prevalence over the last 18 years with the improvements needed between 2017 and 2025 to meet WHO GNTs, we performed a simple projection using estimated annualized rates of change (AROC) applied to the final year of our estimates.

For each CGF indicator, *u*, we calculated AROC at each grid cell, *m*, by calculating the AROC between each pair of adjacent years, *t*:

$${{\rm{AROC}}}_{u,m,t}={\rm{logit}}\left(\frac{{p}_{u,m,t}}{{p}_{u,m,t-1}}\right)$$

We then calculated a weighted AROC for each indicator by taking a weighted average across the years, where more recent AROCs were given more weight in the average. We defined the weights to be:

$${W}_{t}={(t-2000+1)}^{\gamma }$$

in which *γ* may be chosen to give varying amounts of weight across the years. For any indicator, we then calculated the average AROC to be:

$${{\rm{AROC}}}_{u,m}={\rm{logit}}(\mathop{\sum }\limits_{2001}^{2017}{W}_{t}\times {{\rm{AROC}}}_{u,m,t})\,$$

Finally, we calculated the projections, Proj, by applying the AROC in our 2017 mean prevalence estimates to produce estimates in 8 years from 2017 to 2025. For this set of projections, we selected *γ* = 1.7 for stunting, *γ* = 1.9 for wasting, and *γ* = 1.8 for underweight^1^.

$${{\rm{P}}{\rm{r}}{\rm{o}}{\rm{j}}}_{u,m,2025}={{\rm{l}}{\rm{o}}{\rm{g}}{\rm{i}}{\rm{t}}}^{-1}({\rm{l}}{\rm{o}}{\rm{g}}{\rm{i}}{\rm{t}}({p}_{u,m,2017})+{{\rm{A}}{\rm{R}}{\rm{O}}{\rm{C}}}_{u,m}\times 8)$$

This projection scheme is analogous to the methods used in the 2017 GBD measurement of progress and projected attainment of health-related Sustainable Development Goals^1^. Our projections are based on the assumption that areas will sustain the current AROC, and the precision is dependent on the level of uncertainty emanating from the estimation of annual prevalence.

Although the WHO GNT for wasting was to reduce prevalence to less than 5%, the WHO GNT for stunting was a 40% relative reduction in prevalence. For our analyses, we defined the WHO GNT for stunting and underweight (for which no WHO GNT was established) to be 40% reduction relative to 2010, the year the World Health Assembly requested the development of the WHO GNTs^39^.

### Limitations

The accuracy of our models depends on the volume, representativeness, quality, and validity of surveys available for analysis (Supplementary Tables 4, 5, Supplementary Figs. 2–16). Persistent data gaps in national surveys include a lack of CGF data or household-level characteristics, such as hygiene and sanitation practices. The associated uncertainties of our estimates are higher in areas where data are either missing or less reliable (Figs. 1d, 2d, Extended Data Fig. 5d), and rely more heavily on covariates and borrowing from neighbouring areas for their modelling (Supplementary Table 7, Supplementary Fig. 17). Investments in improvements of health surveillance systems and including child anthropometrics as part of routine data collection for profiling population characteristics could improve the certainty of our estimates and better monitor progress towards international goals. In addition, measurement error in collecting anthropometric information, including the child’s age, height, and weight, could have introduced bias or error in the data across different survey types. The accuracy of age data may be affected by differences in sampling approaches and self-reporting bias, such as long recall period or selective recall. Weight and height measurements may be inaccurate owing to improper calibration of equipment, device inaccuracy, different measurement methods, or human error. We did not include a survey random effect to account for between-survey variability in data accuracy; given that most surveys represent a country-year, it would be difficult to distinguish these biases from temporal effects. Our calibration approach in the post-estimation process used only a ratio estimator and did not account for an additive effect, which may have introduced bias. Owing to the complexity of the boosted regression tree sub-model, we were unable to account for the uncertainty of our three sub-models in our final estimates (see Supplementary Information section 3.2.2 for more detail). It is worth noting that our analyses are descriptive and do not support causal inferences on their own. Future research is required to determine the causal pathways for each CGF indicator across and within LMICs.

### Reporting summary

Further information on research design is available in the Nature Research Reporting Summary linked to this paper.

## Online content

Any methods, additional references, Nature Research reporting summaries, source data, extended data, supplementary information, acknowledgements, peer review information; details of author contributions and competing interests; and statements of data and code availability are available at 10.1038/s41586-019-1878-8.

## Supplementary information

Supplementary Information Supplementary Discussion; Supplementary Tables; Supplementary Figures; Supplementary Methods. Additional discussion of associated causes of child growth failure, interventions, and future work. Supplementary Tables 1–22: data sources, fitted parameters, countries estimated to meet WHO GNTs in 2017 and 2025, predictive metrics. Supplementary Figures 1–41: data availability, covariates, seasonal adjustments, validation metrics. Additional methods details. Detailed author contributions.

Reporting Summary

## Data Availability

CGF estimates can be further explored at various spatial scales (national, administrative, and local levels) through our customized online data visualization tools (https://vizhub.healthdata.org/lbd/cgf). The full output of the analyses and the underlying data used in the analyses are publicly available via the Global Health Data Exchange (GHDx; http://ghdx.healthdata.org/record/ihme-data/lmic-child-growth-failure-geospatial-estimates-2000-2017). Some data sources are under special licenses for the current study and are thus not publicly available. Supplementary Tables 4 and 5 show the incorporated data sources, and data with restrictions are marked with an obelisk symbol (†). All maps presented in this study are generated by the authors and no permissions are required to publish them. The findings of this study are supported by data available in public online repositories, data publicly available upon request of the data provider, and data not publicly available owing to restrictions by the data provider. Non-publicly available data were used under license for the current study but may be available from the authors upon reasonable request and with permission of the data provider. Detailed tables and figures of data sources and availability can be found in Supplementary Tables 4, 5, and Supplementary Figs. 2–16. Administrative boundaries were retrieved from the Global Administrative Unit Layers (GAUL)^20^ or the Database of Global Administrative Areas (GADM)^21^. Land cover was retrieved from the online Data Pool, courtesy of the NASA EOSDIS Land Processes Distributed Active Archive Center (LP DAAC), USGS/Earth Resources Observation and Science (EROS) Center, Sioux Falls, South Dakota^40^. Lakes were retrieved from the Global Lakes and Wetlands Database (GLWD), courtesy of the World Wildlife Fund and the Center for Environmental Systems Research, University of Kassel^41,42^. Populations were retrieved from WorldPop^15,16^. All maps in this study were produced using ArcGIS Desktop 10.6.

## Code availability

Our study follows the Guidelines for Accurate and Transparent Health Estimate Reporting (GATHER; Supplementary Table 1). All code used for these analyses is publicly available online http://ghdx.healthdata.org/record/ihme-data/lmic-child-growth-failure-geospatial-estimates-2000-2017 and at http://github.com/ihmeuw/lbd/tree/cgf-lmic-2019.
