# Supplementary material for: Mapping child growth failure across low- and middle-income countries
Source: Nature. 2020 Jan 8;577(7789):231–4. doi: 10.1038/s41586-019-1878-8 (PMC7015855; doi:10.1038/s41586-019-1878-8)
Supplement: Supplementary file 1 — Supplementary Discussion; Supplementary Tables; Supplementary Figures; Supplementary Methods. Additional discussion of associated causes of child growth failure, interventions, and future work. Supplementary Tables 1–22: data sources, fitted parameters, countries estimated to meet WHO GNTs in 2017 and 2025, predictive metrics. Supplementary Figures 1–41: data availability, covariates, seasonal adjustments, validation metrics. Additional methods details. Detailed author contributions. [file 41586_2019_1878_MOESM1_ESM.pdf]

nature
https://doi.org/10.1038/s41586-019-1878-8

Supplementary information

Mapping child growth failure across low- and middle-income countries

In the format provided by the authors and unedited

Local Burden of Disease Child Growth Failure Collaborators*

Supplementary Information

Table of Contents

Supplementary Figures 2

Supplementary Tables 4

0.0.1 Supplementary Discussion 5

1.0 GATHER Compliance 7

2.0 Data 9

2.1 CGF Indicator Definitions, Socio-demographic Index (SDI) Classification 9
2.2 Data Sources 13
2.3 Data Process 48
2.4 Data Availability by Region 49
2.5 Covariates 65
3.0 Supplementary Methods 69

3.1 Seasonality Adjustment 69
3.2 Geostatistical Model 70
3.2.1 Model geographies 70

3.2.2 Ensemble covariate modelling 70

3.2.3 Model description 72

3.2.4 Priors 73

3.2.5 Mesh construction 74

3.2.6 Model fitting and estimate generation 74

3.3 Model Results 77
4.0 Supplementary Results 85

4.1 Additional information for figure descriptions 85
4.2 Countries estimated to meet WHO GNT in 2017 and 2025 at various spatial levels 86
5.0 Model Validation 94

5.1 In-sample metrics 94
5.2 Metrics of predictive validity 94
5.2.1 Stunting validation metrics 95

5.2.2 Wasting validation metrics 102

5.2.3 Underweight validation metrics 109

6.0 Post-estimation Calibration to National and Subnational Estimates 116

7.0 Supplementary References 117

8.0 Author Contributions 120

Supplementary Figures

Supplementary Figure 1: Flowchart for data processes. 48
Supplementary Figure 2: Stunting data availability by type and country, 2000–2017 in Africa. 50
Supplementary Figure 3: Stunting data availability by type and country, 2000–2017 in Central America and the Caribbean and South America. 51
Supplementary Figure 4: Stunting data availability by type and country, 2000–2017 in East and Southeast Asia. 52
Supplementary Figure 5: Stunting data availability by type and country, 2000–2017 in South Asia. 53
Supplementary Figure 6: Stunting data availability by type and country, 2000–2017 in Middle East and Central Asia. 54
Supplementary Figure 7: Wasting data availability by type and country, 2000–2017 in Africa. 55
Supplementary Figure 8: Wasting data availability by type and country, 2000–2017 in Central America and the Caribbean and South America. 56
Supplementary Figure 9: Wasting data availability by type and country, 2000–2017 in East and Southeast Asia. 57
Supplementary Figure 10: Wasting data availability by type and country, 2000–2017 in South Asia. 58
Supplementary Figure 11: Wasting data availability by type and country, 2000–2017 in Middle East and Central Asia. 59
Supplementary Figure 12: Underweight data availability by type and country, 2000–2017 in Africa. 60
Supplementary Figure 13: Underweight data availability by type and country, 2000–2017 in Central America and the Caribbean and South America. 61
Supplementary Figure 14: Underweight data availability by type and country, 2000–2017 in East and Southeast Asia. 62
Supplementary Figure 15: Underweight data availability by type and country, 2000–2017 in South Asia. 63
Supplementary Figure 16: Underweight data availability by type and country, 2000–2017 in Middle East and Central Asia. 64
Supplementary Figure 17: Covariates. 68
Supplementary Figure 18: Periodic seasonality adjustment. 69
Supplementary Figure 19: Ensemble predicted rasters. 71
Supplementary Figure 20: Finite elements mesh. 76
Supplementary Figure 21: Stunting posterior means and upper and lower 95% uncertainty intervals for 2017. 82
Supplementary Figure 22: Wasting posterior means and upper and lower 95% uncertainty intervals for 2017. 83
Supplementary Figure 23: Underweight posterior means and upper and lower 95% uncertainty intervals for 2017. 84
Supplementary Figure 24: Stunting national-level (Admin 0) aggregation in-sample. 96
Supplementary Figure 25: Stunting national-level (Admin 0) aggregation out-of-sample. 97
Supplementary Figure 26: Stunting first-administrative-level (Admin 1) aggregation in-sample. 98
Supplementary Figure 27: Stunting first-administrative-level (Admin 1) aggregation out-of-sample. 99

Supplementary Figure 28: Stunting second-administrative-level (Admin 2) aggregation in-sample. .. 100
Supplementary Figure 29: Stunting second-administrative-level (Admin 2) aggregation out-of-sample. 101
Supplementary Figure 30: Wasting national-level (Admin 0) aggregation in-sample. 103
Supplementary Figure 31: Wasting national-level (Admin 0) aggregation out-of-sample. 104
Supplementary Figure 32: Wasting first-administrative-level (Admin 1) aggregation in-sample. 105
Supplementary Figure 33: Wasting first-administrative-level (Admin 1) aggregation out-of-sample. 106
Supplementary Figure 34: Wasting second-administrative-level (Admin 2) aggregation in-sample. .. 107
Supplementary Figure 35: Wasting second-administrative-level (Admin 2) aggregation out-of-sample. 108
Supplementary Figure 36: Underweight national-level (Admin 0) aggregation in-sample. 110
Supplementary Figure 37: Underweight national-level (Admin 0) aggregation out-of-sample. 111
Supplementary Figure 38: Underweight first-administrative-level (Admin 1) aggregation in-sample. 112
Supplementary Figure 39: Underweight first-administrative-level (Admin 1) aggregation out-of-sample. 113
Supplementary Figure 40: Underweight second-administrative-level (Admin 2) aggregation in-sample. 114
Supplementary Figure 41: Underweight second-administrative-level (Admin 2) aggregation out-of-sample. 115

Supplementary Tables

Supplementary Table 1: Guidelines for Accurate and Transparent Health Estimates Reporting (GATHER) checklist. 7
Supplementary Table 2: Definitions of CGF indicators. 9
Supplementary Table 3: Socio-demographic Index (SDI) of countries included in model. 10
Supplementary Table 4: Household surveys used in mapping. 13
Supplementary Table 5: Survey Reports added to model. 40
Supplementary Table 6: Data excluded from model. 43
Supplementary Table 7: Covariates used in mapping. 65
Supplementary Table 8: Spatial hyperparameter priors by region. 74
Supplementary Table 9: Stunting fitted parameters. 77
Supplementary Table 10: Wasting fitted parameters. 78
Supplementary Table 11: Underweight fitted parameters. 80
Supplementary Table 12: Countries predicted to have met WHO GNTs by 2017, with >95% probability. 86
Supplementary Table 13: Countries predicted to meet WHO GNTs by 2025, with >95% probability. ... 90
Supplementary Table 14: Predictive metrics for stunting aggregated to the national level (Admin 0). 95
Supplementary Table 15: Predictive metrics for stunting aggregated to the first administrative level (Admin 1). 95
Supplementary Table 16: Predictive metrics for stunting aggregated to the second administrative level (Admin 2). 95
Supplementary Table 17: Predictive metrics for wasting aggregated to the national level (Admin 0). 102
Supplementary Table 18: Predictive metrics for wasting aggregated to the first administrative level (Admin 1). 102
Supplementary Table 19: Predictive metrics for wasting aggregated to the second administrative level (Admin 2). 102
Supplementary Table 20: Predictive metrics for underweight aggregated to the national level (Admin 0). 109
Supplementary Table 21: Predictive metrics for underweight aggregated to the first administrative level (Admin 1). 109
Supplementary Table 22: Predictive metrics for underweight aggregated to the second administrative level (Admin 2). 109

0.0.1 Supplementary Discussion

The two main immediate causes of child growth failure (CGF) are the insufficient quantity and nutritional quality of food, and infections[1,2]. Stunting is a chronic condition marked by inadequate linear growth, often indicative of long-term inadequate nutrition and recurrent infections[3,4]. Wasting encompasses moderate and severe acute malnutrition, indicative of short-term weight loss often due to insufficient food intake or incident infectious disease such as diarrhoea[3,4]. Underweight is a composite of both stunting and wasting, and thus has fewer direct implications for public health decision making[3,4].

Stunting is recognised as a reliable proxy of physical well-being in children and can provide insight into health inequalities faced by a population[4,5]. Cross-cutting community-level solutions are needed to prevent or ameliorate stunting and its multigenerational economic and productivity outcomes on mothers and their children[6–8]. Peru's results-based budgeting strategy (El Presupuesto por Resultados (PpR)), which includes community-level vaccination campaigns, infant and child growth monitoring, and promotion of improved hygiene and feeding practices, has been praised as one of the key drivers in successfully halving stunting levels in less than a decade[9]. In order to achieve reduction of child stunting prevalence to 25% nationally by 2022, India has expanded its National Nutrition Mission programme (POSHAN Abhiyaan) to an additional 315 districts, focusing on areas with particularly high stunting and poor socioeconomic conditions[10]. India's national nutrition policy[11] and nutrition projects in India's Bellary district in Karnataka[12]; the Tamil Nadu and Dular scheme in Bihar[12]; and child malnutrition management in the drought-affected Rajasthan district[13] have shown varied success in improving child growth. Beyond investments supporting proximal solutions for nutritional deficiencies and infectious diseases in pregnant women and children, such as nutrition supplement programmes, breastfeeding and complimentary feeding support, vaccination, and sanitation campaigns[14,7], it is critical to simultaneously invest in breaking the cyclical patterns of poverty that risk affecting all future generations. CGF is implicated as both a cause and consequence of entrenched cycles of poverty, as the wider socioeconomic, environmental, and political contexts of nations and communities are underlying factors that perpetuate intergenerational cycles of poor health and converge with poverty to increase risks of stunting[6,15]. Local estimates aid in identifying communities that are most likely experiencing extreme multidimensional disadvantage.

Wasting is an acute condition associated with food shortages and famine that accompany drought and conflict[5,16]. In spite of years of humanitarian interventions in low- and middle-income countries (LMICs), wasting has persisted along the African Sahel and in South Asia, with some areas exceeding the critical, emergency-level threshold (≥15%)[5]; these levels have spanned more than a decade in areas of Niger, Chad, South Sudan, Pakistan, and India. Arid and semi-arid areas of Somalia, northeastern Kenya, and Ethiopia's Afar and Somali regions continue to experience endemic wasting as increasingly erratic climatic conditions[17], competition for resources, and political or civil instability constantly threaten livelihoods and food security[18,19]. Political unrest, armed conflict, and violence disrupt food production and distribution, contributing to increased risks of wasting[19–22]; areas in northern Nigeria, DRC, southern Pakistan, and Pakistan-border areas in Afghanistan experienced conflict and high wasting (≥10%)[23] throughout the 2000–2017 period. Particular first administrative-level units have consistently experienced climatic events or conflict, as well as persistently high wasting, including Zinder (Niger), Kanem (Chad), Unity (South Sudan), Maharashtra and Telangana (India), where wasting reached critical levels every year from 2000 to 2017. The persistent nature of wasting in these countries and areas calls for policy and programming prioritization. Organisations have assisted in reducing wasting prevalence through community-level awareness-raising, active screening, and therapeutic feeding programmes, such as the French Red Cross in villages throughout Niger's Niamey, Agadez, and Zinder provinces[24].

These maps highlight countries and specific locations that have maintained elevated levels of CGF over time, enabling policy makers, public health practitioners, and donors with tools to support efficient directing of investments and development assistance. These estimates could also aid in developing strategically-placed improved nutrition surveillance systems across countries and regions to inform the most appropriate responses for optimum impact and return on investment[25]. The effort needed to achieve WHO GNTs will depend on current rates of progress, country-specific population growth rates, nutrition policies and programmes, and the amount of resources allocated toward implementation. We believe the findings of this paper are important to further the field of demographic research and to guide global, national, and administrative-level decision making.

We intend to regularly update and eventually expand these analyses to include high-income country estimates via our user-friendly online visualisation tools. In future analyses, we plan to determine how to incorporate sub-model uncertainty in our results, stratify our CGF estimates by sex and age, assess the double burden of child undernutrition and overweight, analyse important maternal indicators that impact child nutritional status outcomes (such as anemia), and continue to monitor progress toward 2025 WHO GNTs. In doing so, we aim to provide the necessary platform to examine past, current, and future trajectories of malnutrition comprehensively to support evaluation of programme and policy success and the precise targeting of resources to highly-affected populations for the greatest impact.

1.0 GATHER Compliance

Supplementary Table 1: Guidelines for Accurate and Transparent Health Estimates Reporting (GATHER) checklist.

Item #	Checklist item	Reported on page #
**Objectives and funding**		
**1**	Define the indicator(s), populations (including age, sex, and geographic entities), and time period(s) for which estimates were made.	Manuscript: Introduction, Methods (Data)
**2**	List the funding sources for the work.	Manuscript: Acknowledgements
**Data Inputs**		
For all data inputs from multiple sources that are synthesized as part of the study:		
**3**	Describe how the data were identified and how the data were accessed.	Manuscript: Methods (Data: *Surveys and child anthropometry data*; Data availability); Supplementary Information: 2.0 Data
**4**	Specify the inclusion and exclusion criteria. Identify all ad-hoc exclusions.	Manuscript: Methods (Overview; Data: *Surveys and child anthropometry data*); Supplementary Information: 2.2 Data Sources; Supplementary Table 6; 2.3 Data Process; Supplementary Figure 1
**5**	Provide information on all included data sources and their main characteristics. For each data source used, report reference information or contact name/institution, population represented, data collection method, year(s) of data collection, sex and age range, diagnostic criteria or measurement method, and sample size, as relevant.	Supplementary Information: 2.0 Data
**6**	Identify and describe any categories of input data that have potentially important biases (e.g., based on characteristics listed in item 5).	Manuscript: Methods (Limitations);
For data inputs that contribute to the analysis but were not synthesized as part of the study:		
**7**	Describe and give sources for any other data inputs.	Manuscript: Methods (Data: *Spatial covariates*); Supplementary Information: 2.5 Covariates
For all data inputs:		
**8**	Provide all data inputs in a file format from which data can be efficiently extracted (e.g., a spreadsheet rather than a PDF), including all relevant meta-data listed in item 5. For any data inputs that cannot be shared because of ethical or legal reasons, such as third-party ownership, provide a contact name or the name of the institution that retains the right to the data.	Available at http://ghdx.healthdata.org/record/ihme-data/lmic-child-growth-failure-geospatial-estimates-2000-2017 Supplementary Information: 2.0 Data

**Data analysis**		
**9**	Provide a conceptual overview of the data analysis method. A diagram may be helpful.	Manuscript: Methods (Data), Extended Data Figure 9; Supplementary Information: Data Process, Supplementary Figure 1
**10**	Provide a detailed description of all steps of the analysis, including mathematical formulae. This description should cover, as relevant, data cleaning, data pre-processing, data adjustments and weighting of data sources, and mathematical or statistical model(s).	Manuscript: Methods (Analysis); Supplementary Information: 3.1 Seasonality adjustment, 3.2 Geostatistical model
**11**	Describe how candidate models were evaluated and how the final model(s) were selected.	Manuscript: Methods (Analysis: *Model validation*); Supplementary Information: 5.0 Model validation
**12**	Provide the results of an evaluation of model performance, if done, as well as the results of any relevant sensitivity analysis.	Manuscript: Methods (Analysis: *Model validation*); Supplementary Information: 5.0 Model validation
**13**	Describe methods for calculating uncertainty of the estimates. State which sources of uncertainty were, and were not, accounted for in the uncertainty analysis.	Manuscript: Methods (Analysis: *Geostatistical model*); Supplementary Information: 3.2.3 Model description, 3.2.6 Model fitting and estimate generation
**14**	State how analytic or statistical source code used to generate estimates can be accessed.	Available at http://ghdx.healthdata.org/record/ihme-data/lmic-child-growth-failure-geospatial-estimates-2000-2017 And at http://github.com/ihmeuw/lbd/tree/cgf-lmic-2019
**Results and Discussion**		
**15**	Provide published estimates in a file format from which data can be efficiently extracted.	Raster files for spatial data and CSVs of estimates available at http://ghdx.healthdata.org/record/ihme-data/lmic-child-growth-failure-geospatial-estimates-2000-2017
**16**	Report a quantitative measure of the uncertainty of the estimates (e.g. uncertainty intervals).	Manuscript: Figs 1d, 2d, Extended Data Fig 5d; Supplementary Information: Supplementary Figs. 21–23 and Supplementary Figs. 24–41
**17**	Interpret results in light of existing evidence. If updating a previous set of estimates, describe the reasons for changes in estimates.	Manuscript: Main Text, Methods (Overview)
**18**	Discuss limitations of the estimates. Include a discussion of any modelling assumptions or data limitations that affect interpretation of the estimates.	Manuscript: Methods (Limitations)

2.0 Data

2.1 CGF Indicator Definitions, Socio-demographic Index (SDI) Classification

We modelled the prevalence of child growth failure (CGF) indicators (stunting, wasting, and underweight; defined in Supplementary Table 2) in 105 low- and middle-income countries (LMICs) from 2000 to 2017. These countries were determined by their Socio-demographic Index (SDI), a summary measure of development which combines education, fertility, and poverty, which was developed and computed by the Global Burden of Disease (GBD) study[26]. Countries were assigned stages based on their SDI quintile, as well as their geographic continuity. The 105 LMICs (Stage 1 and Stage 2 countries) we modelled in this study are described below, along with their SDI and SDI quintile (e.g, low, low-middle, middle) (Supplementary Table 3). Stage 1 countries were included in our previous study estimating fine geospatial CGF prevalence throughout the continent of Africa[27], and Stage 2 countries encompass LMICs in other continents. French Guiana and Western Sahara were not modelled by GBD and therefore do not have a calculated SDI, but were included in this study for geographic continuity. China, Iran, Libya, and Malaysia were included despite high-middle SDI status to create better geographic continuity. Albania and Moldova were excluded despite their Middle SDI status due to geographic discontinuity with other included countries and lack of available survey data. We did not estimate for the island nations of American Samoa, Federated States of Micronesia, Fiji, Kiribati, Marshall Islands, North Korea, Samoa, Solomon Islands, or Tonga, where no available survey data could be sourced.

Supplementary Table 2: Definitions of CGF indicators.

Indicator	Definition
Stunting	Stunting is low length/height-for-age. Normal heights for age are determined by the WHO healthy reference population median[28]. Stunting is defined as height-for-age z-scores (HAZ) that were two or more standard deviations below the normal HAZ.
Wasting	Wasting is low weight-for-length/height. Normal weights for length/height are determined by the WHO healthy reference population median[28]. Wasting is defined as weight-for-height z-scores (WHZ) that were two or more standard deviations below the normal WHZ.
Underweight	Underweight is low weight-for-age. Normal weights for age are determined by the WHO healthy reference population median[28]. Underweight is defined as weight-for-age z-scores (WAZ) that were two or more standard deviations below the normal WAZ.

Supplementary Table 3: Socio-demographic Index (SDI) of countries included in model.

Country	Stage	SDI	SDI Quintile
Afghanistan	2	0.2903	Low SDI
Algeria	1	0.6958	Middle SDI
Angola	1	0.4605	Low-middle SDI
Bangladesh	2	0.4580	Low SDI
Belize	2	0.6022	Low-middle SDI
Benin	1	0.3734	Low SDI
Bhutan	2	0.5699	Low-middle SDI
Bolivia	2	0.5874	Low-middle SDI
Botswana	1	0.6632	Middle SDI
Brazil	2	0.6633	Middle SDI
Burkina Faso	1	0.2839	Low SDI
Burundi	1	0.3097	Low SDI
Cambodia	2	0.4816	Low-middle SDI
Cameroon	1	0.4820	Low-middle SDI
Cape Verde	1	0.5491	Low-middle SDI
Central African Republic	1	0.3344	Low SDI
Chad	1	0.2529	Low SDI
China	2	0.7073	High-middle SDI
Colombia	2	0.6337	Middle SDI
Comoros	1	0.4343	Low SDI
Costa Rica	2	0.6621	Middle SDI
Côte d'Ivoire	1	0.4121	Low SDI
Cuba	2	0.6877	Middle SDI
Democratic Republic of the Congo	1	0.3645	Low SDI
Djibouti	1	0.4848	Low-middle SDI
Dominican Republic	2	0.5926	Low-middle SDI
Ecuador	2	0.6356	Middle SDI
Egypt	1	0.6043	Low-middle SDI
El Salvador	2	0.5931	Low-middle SDI
Equatorial Guinea	1	0.6252	Middle SDI
Eritrea	1	0.4088	Low SDI
Ethiopia	1	0.3342	Low SDI
French Guiana	2	NA	NA
Gabon	1	0.6506	Middle SDI
Ghana	1	0.5370	Low-middle SDI
Guatemala	2	0.5242	Low-middle SDI
Guinea	1	0.3247	Low SDI
Guinea-Bissau	1	0.3490	Low SDI
Guyana	2	0.5837	Low-middle SDI
Haiti	2	0.4417	Low SDI

Country	Stage	SDI	SDI Quintile
Honduras	2	0.5123	Low-middle SDI
India	2	0.5502	Low-middle SDI
Indonesia	2	0.6476	Middle SDI
Iran	2	0.7001	High-middle SDI
Iraq	2	0.5848	Low-middle SDI
Jamaica	2	0.6785	Middle SDI
Jordan	2	0.6968	Middle SDI
Kenya	1	0.4995	Low-middle SDI
Kyrgyzstan	2	0.6066	Low-middle SDI
Laos	2	0.5188	Low-middle SDI
Lesotho	1	0.4934	Low-middle SDI
Liberia	1	0.3284	Low SDI
Libya	1	0.7609	High-middle SDI
Madagascar	1	0.3308	Low SDI
Malawi	1	0.3493	Low SDI
Malaysia	2	0.7592	High-middle SDI
Mali	1	0.2669	Low SDI
Mauritania	1	0.4706	Low-middle SDI
Mexico	2	0.6284	Middle SDI
Mongolia	2	0.6619	Middle SDI
Morocco	1	0.5792	Low-middle SDI
Mozambique	1	0.3405	Low SDI
Myanmar	2	0.5558	Low-middle SDI
Namibia	1	0.6158	Middle SDI
Nepal	2	0.4285	Low SDI
Nicaragua	2	0.5296	Low-middle SDI
Niger	1	0.1906	Low SDI
Nigeria	1	0.4934	Low-middle SDI
Pakistan	2	0.4922	Low-middle SDI
Palestine	2	0.5414	Low-middle SDI
Panama	2	0.6770	Middle SDI
Papua New Guinea	2	0.4190	Low SDI
Paraguay	2	0.6188	Middle SDI
Peru	2	0.6358	Middle SDI
Philippines	2	0.6172	Middle SDI
Republic of the Congo	1	0.5741	Low-middle SDI
Rwanda	1	0.4074	Low SDI
São Tomé and Príncipe	1	0.4883	Low-middle SDI
Senegal	1	0.3730	Low SDI
Sierra Leone	1	0.3572	Low SDI
Somalia	1	0.2348	Low SDI
South Africa	1	0.6765	Middle SDI

Country	Stage	SDI	SDI Quintile
South Sudan	1	0.2747	Low SDI
Sri Lanka	2	0.6797	Middle SDI
Sudan	1	0.4779	Low-middle SDI
Suriname	2	0.6410	Middle SDI
Swaziland (eSwatini)	1	0.5777	Low-middle SDI
Syria	2	0.6111	Middle SDI
Tajikistan	2	0.5226	Low-middle SDI
Tanzania	1	0.4122	Low SDI
Thailand	2	0.6843	Middle SDI
The Gambia	1	0.4048	Low SDI
Timor-Leste	2	0.5048	Low-middle SDI
Togo	1	0.4133	Low SDI
Trinidad and Tobago	2	0.6984	Middle SDI
Tunisia	1	0.6754	Middle SDI
Turkmenistan	2	0.6964	Middle SDI
Uganda	1	0.3877	Low SDI
Uzbekistan	2	0.6295	Middle SDI
Venezuela	2	0.6554	Middle SDI
Vietnam	2	0.6068	Middle SDI
Western Sahara	1	NA	NA
Yemen	2	0.4295	Low SDI
Zambia	1	0.4722	Low-middle SDI
Zimbabwe	1	0.4632	Low-middle SDI

2.2 Data Sources

The data sources used to model CGF indicators are described below. Information on survey locations, years, source, and number of individuals, polygons, and/or geo-positioned clusters can be found in Supplementary Table 4–5. Excluded datasets and reasons for their exclusion from our analysis are detailed in Supplementary Table 6.

Supplementary Table 4: Household surveys used in mapping.

Number identification (NID) can be used to locate a particular data source in the Global Health Data Exchange (GHDx) at http://ghdx.healthdata.org/.
**Indicates a survey from a country we previously modelled that has been added since the first publication.*
†Data source is not publicly available due to restrictions by the data provider and was used under license for the current study.

Country	Survey year(s)	Survey Name	Number of Individuals	Number of geo-positioned clusters	Number of polygons (areal)	GHDx NID
Algeria	2002–2003	Algeria Family Health Survey 2002–2003	4,754	0	47	627†
Algeria	2012–2013	Algeria Multiple Indicator Cluster Survey 2012–2013	13,909	0	7	210614
Angola	2001	Angola Multiple Indicator Cluster Survey 2001	5,461	0	18	687
Angola	2015–2016	Angola Demographic and Health Survey 2015–2016	6,583	625	0	218555
Bangladesh	1999–2000	Bangladesh Demographic and Health Survey 1999–2000	5,970	341	0	26826
Bangladesh	2004	Bangladesh Demographic and Health Survey 2004	6,186	359	0	18902
Bangladesh	2007	Bangladesh Demographic and Health Survey 2007	5,531	361	0	18913
Bangladesh	2011–2012	Bangladesh Demographic and Health Survey 2011–2012	7,992	600	0	55956
Bangladesh	2012–2013	Bangladesh Multiple Indicator Cluster Survey 2012–2013	19,397	2,625	0	151086

Country	Survey year(s)	Survey Name	Number of Individuals	Number of geo-positioned clusters	Number of polygons (areal)	GHDx NID
Bangladesh	2014	Bangladesh Demographic and Health Survey 2014	7,341	0	7	157021
Belize	2006	Belize Multiple Indicator Cluster Survey 2006	770	0	6	1089
Belize	2011	Belize Multiple Indicator Cluster Survey 2011	1,831	0	7	76699
Belize	2015–2016	Belize Multiple Indicator Cluster Survey 2015–2016	2,448	0	6	264910
Benin	2001	Benin Demographic and Health Survey 2001	4,533	247	0	18950
Benin	2006	Benin Demographic and Health Survey 2006	13,517	0	12	18959
Benin	2014	Benin Multiple Indicator Cluster Survey 2014*	12,113	0	12	206075
Benin	2017–2018	Benin Demographic and Health Survey 2017–2018*	11,832	540	0	218565
Bhutan	2010	Bhutan Multiple Indicator Cluster Survey 2010	6,171	0	20	40028
Bolivia	2003–2004	Bolivia Demographic and Health Survey 2003–2004	9,396	0	8	19001
Bolivia	2008	Bolivia Demographic and Health Survey 2008	7,880	986	0	19016
Bolivia	2012	Bolivia Health and Nutrition Assessment Survey 2012	10,941	7,320	0	285880
Botswana	2000	Botswana Multiple Indicator Cluster Survey 2000	2,877	0	14	1404
Botswana	2007–2008	Botswana Family Health Survey 2007–2008	2,167	0	323	22125†
Brazil	2002–2003	Brazil Consumer Expenditure Survey 2002–2003	17,411	0	27	33019
Brazil	2006–2007	Brazil National Demographic and Health Survey of Children and Women 2006–2007	4,549	0	5	141948
Burkina Faso	2003	Burkina Faso Core Welfare Indicators Questionnaire Survey 2003	7,797	0	232	1855†
Burkina Faso	2003	Burkina Faso Demographic and Health Survey 2003	8,808	397	0	19088

Country	Survey year(s)	Survey Name	Number of Individuals	Number of geo-positioned clusters	Number of polygons (areal)	GHDx NID
Burkina Faso	2006	Burkina Faso Multiple Indicator Cluster Survey 2006	5,101	195	0	1927
Burkina Faso	2007	Burkina Faso Core Welfare Indicators Questionnaire Survey 2007	4,417	0	13	18499†
Burkina Faso	2010–2011	Burkina Faso Demographic and Health Survey 2010–2011	6,381	540	0	19133
Burkina Faso	2014	Burkina Faso Continuous Multisectoral Survey 2014	11,007	0	13	236156
Burundi	2000	Burundi Multiple Indicator Cluster Survey 2000	2,689	0	17	1994
Burundi	2010–2011	Burundi Demographic and Health Survey 2010–2011	3,520	376	0	30431
Burundi	2016–2017	Burundi Demographic and Health Survey 2016–2017*	6,059	552	0	286766
Cambodia	2000	Cambodia Demographic and Health Survey 2000	3,785	467	0	19156
Cambodia	2003–2005	Cambodia Socio-Economic Survey 2003–2005	2,656	0	14	30963†
Cambodia	2005–2006	Cambodia Demographic and Health Survey 2005–2006	3,673	546	0	19167
Cambodia	2006–2007	Cambodia Socio-Economic Survey 2006–2007	1,593	27	260	31050†
Cambodia	2008	Cambodia Anthropometric Survey 2008 – National Institute of Statistics	8,580	0	709	135773†
Cambodia	2009	Cambodia Socio-Economic Survey 2009	5,456	0	517	31143†
Cambodia	2010–2011	Cambodia Demographic and Health Survey 2010–2011	3,816	604	0	30379
Cambodia	2014	Cambodia Demographic and Health Survey 2014	4,466	608	0	157024

Country	Survey year(s)	Survey Name	Number of Individuals	Number of geo-positioned clusters	Number of polygons (areal)	GHDx NID
Cameroon	2004	Cameroon Demographic and Health Survey 2004	3,352	446	0	19211
Cameroon	2006	Cameroon Multiple Indicator Cluster Survey 2006	6,178	0	191	2063
Cameroon	2011	Cameroon Demographic and Health Survey 2011	5,211	574	0	19274
Cameroon	2014	Cameroon Multiple Indicator Cluster Survey 2014	6,758	0	206	244455
Central African Republic	2000	Central African Republic Multiple Indicator Cluster Survey 2000	13,722	0	17	2209
Central African Republic	2006	Central African Republic Multiple Indicator Cluster Survey 2006	9,240	0	16	2223
Central African Republic	2010–2011	Central African Republic Multiple Indicator Cluster Survey 2010–2011	10,344	0	17	82832
Chad	2000	Chad Multiple Indicator Cluster Survey 2000	5,314	0	15	2244
Chad	2004	Chad Demographic and Health Survey 2004	4,725	0	9	19315
Chad	2010	Chad Multiple Indicator Cluster Survey 2010	15,641	0	60	76701
Chad	2014–2015	Chad Demographic and Health Survey 2014–2015	10,524	623	0	157025
China	2010	Chinese Family Panel Studies Baseline 2010	2,629	0	25	283812
China	1989–2011	China Health and Nutrition Survey 1989–2011	5,207	0	9	200838
China	2016	Chinese Family Panel Studies Follow-Up 2016	2,773	0	30	369294
China	2016	China Family Dynamics Survey 2016	1,577	0	301	399041†
Colombia	2000	Colombia Demographic and Health Survey 2000	4,264	0	23	19359
Colombia	2004–2005	Colombia Demographic and Health Survey 2004–2005	12,574	0	33	19324

Country	Survey year(s)	Survey Name	Number of Individuals	Number of geo-positioned clusters	Number of polygons (areal)	GHDx NID
Colombia	2009–2010	Colombia Demographic and Health Survey 2009–2010	15,798	4,279	0	21281
Comoros	2000	Comoros Multiple Indicator Cluster Survey 2000	4,623	0	3	3114
Comoros	2012–2013	Comoros Demographic and Health Survey 2012–2013	2,657	241	0	76850
Cote d'Ivoire	2006	Côte d'Ivoire Multiple Indicator Cluster Survey 2006	8,621	0	52	26433
Cote d'Ivoire	2011–2012	Côte d'Ivoire Demographic and Health Survey 2011–2012	3,228	341	0	18533
Cote d'Ivoire	2016	Cote d'Ivoire Multiple Indicator Cluster Survey 2016*	9,032	0	11	218611
Cuba	2014	Cuba Multiple Indicator Cluster Survey 2014	5,397	0	4	169975
Democratic Republic of the Congo	2001	Democratic Republic of the Congo Multiple Indicator Cluster Survey 2001	10,073	0	11	3161
Democratic Republic of the Congo	2007	Democratic Republic of the Congo Demographic and Health Survey 2007	3,647	293	0	19381
Democratic Republic of the Congo	2010	Democratic Republic of the Congo Multiple Indicator Cluster Survey 2010	10,233	357	0	26998
Democratic Republic of the Congo	2013	Democratic Republic of the Congo Demographic and Health Survey 2013–2014	7,765	492	0	76878
Djibouti	2006	Djibouti Multiple Indicator Cluster Survey 2006	2,193	35	1	3404
Djibouti	2012	Djibouti Family Health Survey 2012	3,549	0	6	218035

Country	Survey year(s)	Survey Name	Number of Individuals	Number of geo-positioned clusters	Number of polygons (areal)	GHDx NID
Dominican Republic	2000	Dominican Republic Multiple Indicator Cluster Survey 2000	1,918	0	30	27069
Dominican Republic	2002	Dominican Republic Demographic and Health Survey 2002	9,613	0	32	19444
Dominican Republic	2006	Dominican Republic National Multipurpose Household Survey 2006	3,788	0	32	3455†
Dominican Republic	2007	Dominican Republic Demographic and Health Survey 2007	9,559	1,397	0	19456
Dominican Republic	2007	Dominican Republic Special Demographic and Health Survey 2007	811	0	9	21198
Dominican Republic	2013	Dominican Republic Demographic and Health Survey 2013	3,251	513	0	77819
Dominican Republic	2013	Dominican Republic Special Demographic and Health Survey 2013	794	111	0	165645
Ecuador	2004	Ecuador Reproductive Health Survey 2004	5,297	680	0	27630
Ecuador	2005–2006	Ecuador Living Conditions Survey 2005–2006	5,614	283	88	46924
Ecuador	2012	Ecuador National Health and Nutrition Survey 2012	6,151	0	480	153674
Egypt	2000	Egypt Demographic and Health Survey 2000	10,743	985	0	19511
Egypt	2003	Egypt Interim Demographic and Health Survey 2003	5,425	876	0	19529
Egypt	2005	Egypt Demographic and Health Survey 2005	12,639	1,288	0	19521
Egypt	2008	Egypt Demographic and Health Survey 2008	10,331	1,221	0	26842
Egypt	2014	Egypt Demographic and Health Survey 2014	15,153	1,736	0	154897
Egypt	2013–2014	Egypt IPHN Rural Districts Multiple Indicator Cluster Survey 2013–2014	5,090	0	6	159617

Country	Survey year(s)	Survey Name	Number of Individuals	Number of geo-positioned clusters	Number of polygons (areal)	GHDx NID
El Salvador	2002–2003	El Salvador Reproductive Health Survey 2002–2003	5,328	0	14	27599
El Salvador	2008	El Salvador Reproductive Health Survey 2008	4,651	0	14	27606
El Salvador	2014	El Salvador Multiple Indicator Cluster Survey 2014	7,288	0	14	200636
Equatorial Guinea	2000	Equatorial Guinea Multiple Indicator Cluster Survey 2000	2,424	0	7	3655
Eritrea	2002	Eritrea Demographic and Health Survey 2002	5,727	0	6	19539†
Ethiopia	2017	Spatial heterogeneity and risk factors for stunting among children under age five in Ethiopia: A Bayesian geo-statistical model*	3,974	3,035	0	319322†
Ethiopia	2000	Ethiopia Demographic and Health Survey 2000	9,062	533	0	19571
Ethiopia	2005	Ethiopia Demographic and Health Survey 2005	4,196	520	0	19557
Ethiopia	2010–2011	Ethiopia Demographic and Health Survey 2010–2011	9,639	571	0	21301
Ethiopia	2011–2012	Ethiopia Rural Socioeconomic Survey 2011–2012	2,474	332	0	93848
Ethiopia	2014	Ethiopia Mini Demographic and Health Survey 2014*	4,888	0	11	153507†
Ethiopia	2013–2014	Ethiopia Socioeconomic Survey 2013–2014	2,792	434	0	235215
Ethiopia	2016	Ethiopia Demographic and Health Survey 2016	8,779	619	0	218568
Ethiopia	2015–2016	Ethiopia Socioeconomic Survey 2015–2016*	2,749	462	0	286657
Ethiopia	2015–2016	Ethiopia Welfare Monitoring Survey 2015–2016*	10,292	7,463	2	365281†
Gabon	2000–2001	Gabon Demographic and Health Survey 2000–2001	3,585	0	40	19579
Gabon	2012	Gabon Demographic and Health Survey 2012	3,500	324	0	76706
Ghana	2003	Ghana Demographic and Health Survey 2003	3,270	407	0	19627

Country	Survey year(s)	Survey Name	Number of Individuals	Number of geo-positioned clusters	Number of polygons (areal)	GHDx NID
Ghana	2003	Ghana Core Welfare Indicators Questionnaire Survey 2003*	24,011	0	110	23017†
Ghana	2006	Ghana Multiple Indicator Cluster Survey 2006	3,413	0	10	4694
Ghana	2008	Ghana Demographic and Health Survey 2008	2,529	400	0	21188
Ghana	2007–2008	Ghana District Multiple Indicator Cluster Survey 2007–2008	8,415	0	4	160576†
Ghana	2010–2011	Ghana – Accra Multiple Indicator Cluster Survey 2010–2011	439	5	0	56241
Ghana	2011	Ghana Multiple Indicator Cluster Survey 2011	6,902	738	0	63993
Ghana	2014	Ghana Demographic and Health Survey 2014	2,721	414	0	157027
Guatemala	2000	Guatemala Living Standards Measurement Survey 2000	5,708	0	8	45718
Guatemala	2002	Guatemala Reproductive Health Survey 2002	6,538	370	373	27563
Guatemala	2008–2009	Guatemala Reproductive Health Survey 2008–2009	8,299	0	22	4779
Guatemala	2014–2015	Guatemala Demographic and Health Survey 2014–2015	11,761	851	0	157031
Guinea	2005	Guinea Demographic and Health Survey 2005	2,718	290	0	19683
Guinea	2012	Guinea Demographic and Health Survey 2012	3,232	300	0	69761
Guinea	2016	Guinea Multiple Indicator Cluster Survey 2016*	6,756	0	8	303458
Guinea-Bissau	2000	Guinea-Bissau Multiple Indicator Cluster Survey 2000	5,728	0	9	4808
Guinea-Bissau	2006	Guinea-Bissau Multiple Indicator Cluster Survey 2006	5,670	0	9	4818
Guinea-Bissau	2014	Guinea-Bissau Multiple Indicator Cluster Survey 2014	7,579	0	9	174049
Guyana	2000	Guyana Multiple Indicator Cluster Survey 2000	2,581	0	10	4916

Country	Survey year(s)	Survey Name	Number of Individuals	Number of geo-positioned clusters	Number of polygons (areal)	GHDx NID
Guyana	2006–2007	Guyana Multiple Indicator Cluster Survey 2006–2007	2,381	0	10	4926
Guyana	2009	Guyana Demographic and Health Survey 2009	1,676	307	0	21348
Guyana	2014	Guyana Multiple Indicator Cluster Survey 2014	3,110	250	0	200598
Haiti	2000	Haiti Demographic and Health Survey 2000	5,660	317	0	19708
Haiti	2005–2006	Haiti Demographic and Health Survey 2005–2006	2,529	331	0	19720
Haiti	2012	Haiti Demographic and Health Survey 2012	4,002	435	0	65118
Haiti	2016–2017	Haiti Demographic and Health Survey 2016–2017	5,654	449	10	218574
Honduras	2001	Honduras Reproductive Health Survey 2001	5,690	0	16	27551
Honduras	2004	Honduras Survey of Living Conditions 2004	4,834	0	18	5009†
Honduras	2005–2006	Honduras Demographic and Health Survey 2005–2006	9,427	0	16	19728
Honduras	2011–2012	Honduras Demographic and Health Survey 2011–2012	9,920	1,119	0	95440
India	1998–2000	India Demographic and Health Survey 1998–1999	27,937	0	438	19950†
India	2000–2001	India Rural Survey of Diet and Nutritional Status 2000–2001	9,262	0	9	129913†
India	2004–2005	India Human Development Survey 2004–2005	14,265	0	363	26919
India	2005–2006	India Demographic and Health Survey 2005–2006	44,061	0	29	19963
India	2004–2006	India Rural Survey of Diet and Nutritional Status 2004–2006	6,670	0	9	129905†
India	2007–2011	India – Kolkata Global Enteric Multicenter Study 2007–2011	2,014	1	0	224240†

Country	Survey year(s)	Survey Name	Number of Individuals	Number of geo-positioned clusters	Number of polygons (areal)	GHDx NID
India	2011–2012	India Rural Third Repeat Survey of Diet and Nutritional Status 2011–2012	10,300	1,125	3	129770†
India	2011–2013	India – Kolkata Global Enteric Multicenter Study 2011–2013	1,172	1	0	224854†
India	2011–2013	India Human Development Survey 2011–2013	11,268	0	367	165498
India	2012–2014	India District Level Household Survey 2012–2014	73,542	0	271	165390
India	2014	India Clinical, Anthropometric and Bio-chemical Survey 2014	108,693	0	277	233917
India	2015–2016	India Demographic and Health Survey 2015–2016	237,528	28,118	0	157050
India	2015–2016	India Urban Nutrition Survey 2015–2016	12,027	564	0	334953†
Indonesia	2000	Indonesia Family Life Survey 2000	3,984	0	16	6111
Indonesia	2007	Indonesia Family Life Survey 2007–2008	4,776	0	15	6464
Indonesia	2012	Indonesia Family Life Survey East 2012	1,288	0	7	219201
Indonesia	2014–2015	Indonesia Family Life Survey 2014–2015	5,354	0	1,149	264956
Iran	2004	Iran Anthropometric Nutritional Indicators Survey 2004	29,434	0	265	159873†
Iraq	2000	Iraq Multiple Indicator Cluster Survey 2000	14,378	0	18	7054
Iraq	2004	Iraq Multiple Indicator Rapid Assessment 2004	17,585	2,027	0	23565†
Iraq	2006	Iraq Multiple Indicator Cluster Survey 2006	16,473	0	18	7028
Iraq	2011	Iraq Multiple Indicator Cluster Survey 2011	35,847	0	48	76707
Iraq	2012–2013	Iraq Household Socioeconomic Survey 2012–2013	25,777	14,018	18	235348
Iraq	2018	Iraq Multiple Indicator Cluster Survey 2018	16,582	0	18	385708
Jamaica	2000	Jamaica Survey of Living Conditions 2000	562	0	14	45856†
Jamaica	2001	Jamaica Survey of Living Conditions 2001	452	0	14	7222†

Country	Survey year(s)	Survey Name	Number of Individuals	Number of geo-positioned clusters	Number of polygons (areal)	GHDx NID
Jamaica	2002	Jamaica Survey of Living Conditions 2002	1,901	0	14	80626†
Jamaica	2004	Jamaica Survey of Living Conditions 2004	571	0	14	141572†
Jordan	2002	Jordan Demographic and Health Survey 2002	5,014	492	0	20073
Jordan	2007	Jordan Demographic and Health Survey 2007	4,803	462	0	20083
Jordan	2009	Jordan Interim Demographic and Health Survey 2009	4,470	0	12	21206
Jordan	2012	Jordan Demographic and Health Survey 2012	6,410	798	0	77517
Kenya	2000	Kenya Multiple Indicator Cluster Survey 2000	6,709	802	0	7387
Kenya	2003	Kenya Demographic and Health Survey 2003	4,957	397	0	20145
Kenya	2005–2006	Kenya Integrated Household Budget Survey 2005–2006	7,096	1,284	0	7375†
Kenya	2007	Kenya – North Eastern Province Multiple Indicator Cluster Survey 2007	920	76	0	155335
Kenya	2008	Kenya – Eastern Province Multiple Indicator Cluster Survey 2008	12,300	590	0	7401
Kenya	2008–2009	Kenya Demographic and Health Survey 2008–2009	5,373	397	0	21365
Kenya	2009	Kenya – Coast Multiple Indicator Cluster Survey 2009	446	0	1	56420
Kenya	2011	Kenya – Nyanza Province Multiple Indicator Cluster Survey 2011	4,844	289	0	135416
Kenya	2014	Kenya Demographic and Health Survey 2014	18,967	1,583	0	157057
Kenya	2013–2014	Kenya – Bungoma County Multiple Indicator Survey 2013–2014*	812	40	0	203654†
Kenya	2013–2014	Kenya – Kakamega County Multiple Indicator Survey 2013–2014*	738	48	0	203663†

Country	Survey year(s)	Survey Name	Number of Individuals	Number of geo-positioned clusters	Number of polygons (areal)	GHDx NID
Kenya	2013–2014	Kenya – Turkana County Multiple Indicator Survey 2013–2014	1,032	50	0	203664†
Kyrgyzstan	2005–2006	Kyrgyzstan Multiple Indicator Cluster Survey 2005–2006	2,945	0	8	7540
Kyrgyzstan	2012	Kyrgyzstan Demographic and Health Survey 2012	4,067	313	0	77518
Kyrgyzstan	2014	Kyrgyzstan Multiple Indicator Cluster Survey 2014	4,491	0	9	162283
Laos	2000	Laos Multiple Indicator Cluster Survey 2000	1,554	86	0	7618
Laos	2006	Laos Multiple Indicator Cluster Survey 2006	4,050	300	0	7629
Laos	2011–2012	Laos Multiple Indicator Cluster Survey 2011–2012	10,887	0	17	103973
Laos	2017	Laos Multiple Indicator Cluster Survey 2017	11,603	0	18	375362
Lesotho	2004–2005	Lesotho Demographic and Health Survey 2004–2005	1,399	353	0	20167
Lesotho	2009–2010	Lesotho Demographic and Health Survey 2009–2010	1,664	383	0	21382
Lesotho	2014	Lesotho Demographic and Health Survey 2014	1,360	369	0	157058
Liberia	2006–2007	Liberia Demographic and Health Survey 2006–2007	4,550	290	0	20191
Liberia	2013	Liberia Demographic and Health Survey 2013	3,290	322	0	77385
Madagascar	2003–2004	Madagascar Demographic and Health Survey 2003–2004	4,765	0	6	20223
Madagascar	2008–2009	Madagascar Demographic and Health Survey 2008–2009	5,179	583	0	21409
Malawi	2000	Malawi Demographic and Health Survey 2000	9,883	559	0	20252

Country	Survey year(s)	Survey Name	Number of Individuals	Number of geo-positioned clusters	Number of polygons (areal)	GHDx NID
Malawi	2004–2005	Malawi Demographic and Health Survey 2004–2005	8,971	520	0	20263
Malawi	2004–2005	Malawi Living Standards Measurement Survey 2004–2005	6,801	0	26	46317
Malawi	2006	Malawi Multiple Indicator Cluster Survey 2006	22,573	0	26	7919
Malawi	2010	Malawi Demographic and Health Survey 2010	4,838	813	0	21393
Malawi	2010–2011	Malawi Integrated Household Survey 2010–2011	7,750	768	0	93806
Malawi	2010–2011	Malawi Integrated Household Panel Survey, Short-Term Panel, 2010–2013*	4,683	549	0	336401
Malawi	2013	Malawi Integrated Household Survey 2013	2,503	547	0	224223†
Malawi	2013	Malawi Integrated Household Panel Survey, Short-Term Panel, 2010–2013*	4,683	549	0	336401
Malawi	2013–2014	Malawi Multiple Indicator Cluster Survey 2013–2014	18,673	0	31	161662
Malawi	2015–2016	Malawi Demographic and Health Survey 2015–2016	5,284	850	0	218581
Malawi	2016–2017	Malawi Integrated Household Survey 2016–2017*	6,306	0	32	327852
Malawi	2016–2017	Malawi Integrated Household Panel Survey, Long-Term Panel, 2010–2016*	1,396	0	28	327857
Mali	2001	Mali Demographic and Health Survey 2001	10,043	399	0	20315
Mali	2006	Mali Demographic and Health Survey 2006	11,677	405	0	20274
Mali	2009–2010	Mali Multiple Indicator Cluster Survey 2009–2010	23,082	0	50	270627
Mali	2012–2013	Mali Demographic and Health Survey 2012–2013	4,660	412	0	77388

Country	Survey year(s)	Survey Name	Number of Individuals	Number of geo-positioned clusters	Number of polygons (areal)	GHDx NID
Mali	2015	Mali Multiple Indicator Cluster Survey 2015*	15,881	0	8	248224
Mauritania	2000–2001	Mauritania Demographic and Health Survey 2000–2001	4,042	0	13	20322
Mauritania	2007	Mauritania Multiple Indicator Cluster Survey 2007	8,283	0	196	8115
Mauritania	2011	Mauritania Multiple Indicator Cluster Survey 2011	8,977	0	194	152783
Mauritania	2015	Mauritania Multiple Indicator Cluster Survey 2015*	10,285	0	13	267343
Mexico	2005–2006	Mexico National Survey of Health and Nutrition 2005–2006	8,454	0	576	8618
Mexico	2011–2012	Mexico National Survey of Health and Nutrition 2011–2012	8,951	0	2	81748
Mexico	2008–2013	Mexico Family Life Survey 2008–2013	3,312	0	197	160781
Mexico	2016	Mexico National Survey of Health and Nutrition Mid-way 2016	2,082	440	0	316736
Mongolia	2000	Mongolia Multiple Indicator Cluster Survey 2000	5,958	0	17	8788
Mongolia	2005	Mongolia Multiple Indicator Cluster Survey 2005	3,377	0	22	8777
Mongolia	2010	Mongolia Multiple Indicator Cluster Survey 2010	3,737	0	217	76704
Mongolia	2012	Mongolia – Khuvsgul Multiple Indicator Cluster Survey 2012	745	0	23	189045
Mongolia	2012	Mongolia – Nalaikh District Multiple Cluster Indicator Survey 2012	427	0	1	189048

Country	Survey year(s)	Survey Name	Number of Individuals	Number of geo-positioned clusters	Number of polygons (areal)	GHDx NID
Mongolia	2013	Mongolia Multiple Indicator Cluster Survey 2013	5,771	0	527	150866
Mongolia	2016	Mongolia – Khuvsgul Multiple Indicator Cluster Survey 2016	1,084	0	82	335994
Mongolia	2016	Mongolia – Nalaikh District Multiple Indicator Cluster Survey 2016	368	0	7	336042†
Morocco	2003–2004	Morocco Demographic and Health Survey 2003–2004	5,711	479	0	20361
Morocco	2010–2011	Morocco National Survey on Population and Family Health 2010–2011	6,420	0	59	126909†
Mozambique	2003	Mozambique Demographic and Health Survey 2003–2004	8,367	0	11	20394
Mozambique	2008–2009	Mozambique Multiple Indicator Cluster Survey 2008–2009	10,948	67	618	27031
Mozambique	2011	Mozambique Demographic and Health Survey 2011	9,753	609	0	55975
Myanmar	2000	Myanmar Multiple Indicator Cluster Survey 2000	8,592	0	16	8932
Myanmar	2003	Myanmar Multiple Indicator Cluster Survey 2003	5,991	0	17	141910†
Myanmar	2009–2010	Myanmar Multiple Indicator Cluster Survey 2009–2010	15,545	0	17	90696†
Myanmar	2015–2016	Myanmar Demographic and Health Survey 2015–2016	4,320	439	0	157061
Namibia	2000	Namibia Demographic and Health Survey 2000	3,075	256	0	20417
Namibia	2006–2007	Namibia Demographic and Health Survey 2006–2007	3,809	484	0	20428

Country	Survey year(s)	Survey Name	Number of Individuals	Number of geo-positioned clusters	Number of polygons (areal)	GHDx NID
Namibia	2009–2010	Namibia Household Income and Expenditure Survey 2009–2010	5,696	0	13	134371
Namibia	2013	Namibia Demographic and Health Survey 2013	1,882	504	0	150382
Nepal	2001	Nepal Demographic and Health Survey 2001	6,295	248	0	20450
Nepal	2006	Nepal Demographic and Health Survey 2006	5,319	260	0	20462
Nepal	2011	Nepal Demographic and Health Survey 2011	2,365	288	0	21240
Nepal	2014	Nepal Multiple Indicator Cluster Survey 2014	5,170	508	0	162317
Nepal	2016	Nepal Household Risk and Vulnerability Survey 2016, Wave 1	1,826	0	50	400219
Nepal	2016–2017	Nepal Demographic and Health Survey 2016–2017	2,382	375	73	286782
Nicaragua	2001	Nicaragua Living Standards Measurement Survey 2001	2,538	0	117	9422
Nicaragua	2001	Nicaragua Demographic and Health Survey 2001	6,219	0	133	20487
Nicaragua	2005	Nicaragua Living Standards Measurement Survey 2005	3,583	0	134	44645
Nicaragua	2006–2007	Nicaragua Reproductive Health Survey 2006–2007	6,286	0	141	9270
Niger	2000	Niger Multiple Indicator Cluster Survey 2000	4,929	0	8	9439
Niger	2006	Niger Demographic and Health Survey 2006	3,901	0	8	20499
Niger	2012	Niger Demographic and Health Survey 2012	5,208	0	8	74393
Nigeria	2003	Nigeria Demographic and Health Survey 2003	4,813	359	0	20567
Nigeria	2007	Nigeria Multiple Indicator Cluster Survey 2007	16,537	0	37	9516
Nigeria	2008	Nigeria Demographic and Health Survey 2008	23,492	886	0	21433
Nigeria	2010	Nigeria Malaria Indicator Survey 2010	2,072	207	0	30991
Nigeria	2011	Nigeria Multiple Indicator Cluster Survey 2011	24,478	0	37	76703

Country	Survey year(s)	Survey Name	Number of Individuals	Number of geo-positioned clusters	Number of polygons (areal)	GHDx NID
Nigeria	2013	Nigeria Demographic and Health Survey 2013	26,945	888	0	77390
Nigeria	2012–2013	Nigeria General Household Survey 2012–2013	2,801	487	0	151797
Nigeria	2015–2016	Nigeria General Household Survey 2015–2016	2,898	512	0	274160
Nigeria	2016–2017	Nigeria Multiple Indicator Cluster Survey with National Immunization Coverage Survey Supplement 2016–2017*	26,948	2,136	0	218613
Pakistan	2010	Pakistan – Balochistan Multiple Indicator Cluster Survey 2010	6,771	0	31	60942
Pakistan	2011	Pakistan National Nutrition Survey 2011	28,603	1,001	0	141521†
Pakistan	2012–2013	Pakistan Demographic and Health Survey 2012–2013	3,712	0	6	77521
Pakistan	2014	Pakistan – Sindh Multiple Indicator Cluster Survey 2014	16,086	0	28	232763
Pakistan	2014	Pakistan – Punjab Multiple Indicator Cluster Survey 2014	26,900	0	36	236266
Pakistan	2017–2018	Pakistan Demographic and Health Survey 2017–2018	4,271	553	0	286783
Palestine	2000	Palestine – West Bank and Gaza Strip Multiple Indicator Cluster Survey 2000	6,029	0	2	10001†
Palestine	2002	Palestine Nutrition Survey 2002	3,347	0	2	9989†
Palestine	2004	Palestine Demographic and Health Survey 2004	4,691	0	2	20596†
Palestine	2006–2007	Palestine Family Health Survey 2006–2007	9,480	0	16	9999†
Palestine	2010	Palestine Multiple Indicator Cluster Survey 2010	9,408	0	16	125591
Palestine	2014	Palestine Multiple Indicator Cluster Survey 2014	7,256	0	16	161590
Panama	2003	Panama Living Standard Measurement Survey 2003	2,931	0	12	10224

Country	Survey year(s)	Survey Name	Number of Individuals	Number of geo-positioned clusters	Number of polygons (areal)	GHDx NID
Panama	2008	Panama Living Standard Measurement Survey 2008	2,519	0	12	46517
Paraguay	2016	Paraguay Multiple Indicator Cluster Survey 2016	4,463	0	9	324470
Peru	2000	Peru National Living Standards Measurement Survey 2000	1,872	317	88	10460
Peru	2000	Peru Demographic and Health Survey 2000	11,897	1,383	0	20649
Peru	2003–2008	Peru Continuous Demographic and Health Survey 2003–2008	7,022	825	0	275090
Peru	2007–2008	Peru Monitoring of Nutritional Indicators in the National Household Survey 2007–2008	4,175	0	604	359163†
Peru	2009	Peru Continuous Demographic and Health Survey 2009	9,468	1,121	0	270404
Peru	2010	Peru Continuous Demographic and Health Survey 2010	8,854	0	24	270469
Peru	2009–2010	Peru Monitoring of Nutritional Indicators in the National Household Survey 2009–2011	6,062	0	833	359146†
Peru	2011	Peru Continuous Demographic and Health Survey 2011	8,806	0	24	270470
Peru	2012	Peru Continuous Demographic and Health Survey 2012	9,266	0	24	270471
Peru	2013	Peru Continuous Demographic and Health Survey 2013	9,345	0	24	146860
Peru	2014	Peru Continuous Demographic and Health Survey 2014	9,932	0	24	209930
Peru	2015	Peru Demographic and Family Health Survey 2015	23,242	1,614	0	303663

Country	Survey year(s)	Survey Name	Number of Individuals	Number of geo-positioned clusters	Number of polygons (areal)	GHDx NID
Peru	2016	Peru Demographic and Family Health Survey 2016	20,606	1,916	0	303664
Peru	2017	Peru Demographic and Family Health Survey 2017	22,334	1,878	0	358824
Republic of the Congo	2005	Congo Demographic and Health Survey 2005	4,118	0	12	19391
Republic of the Congo	2011–2012	Congo Demographic and Health Survey 2011–2012	4,549	0	12	56151
Republic of the Congo	2014–2015	Congo Multiple Indicator Cluster Survey 2014–2015*	8,893	0	11	234733
Rwanda	2000	Rwanda Demographic and Health Survey 2000	6,429	0	12	20722
Rwanda	2000	Rwanda Multiple Indicator Cluster Survey 2000	2,848	0	12	26930
Rwanda	2001	Rwanda Integrated Household Living Conditions Survey 1999–2001*	4,075	0	12	11319
Rwanda	2005	Rwanda Demographic and Health Survey 2005	3,775	455	0	20740
Rwanda	2006	Rwanda Comprehensive Food Security and Vulnerability Assessment 2006	1,869	0	281	58185
Rwanda	2010–2011	Rwanda Demographic and Health Survey 2010–2011	4,145	492	0	56040
Rwanda	2012	Rwanda Comprehensive Food Security and Vulnerability Assessment and Nutrition Survey 2012	4,446	0	743	151436
Rwanda	2014–2015	Rwanda Demographic and Health Survey 2014–2015	3,617	491	0	157063
Sao Tome and Principe	2000	Sao Tome and Principe Multiple Indicator Cluster Survey 2000	1,842	0	4	27055

Country	Survey year(s)	Survey Name	Number of Individuals	Number of geo-positioned clusters	Number of polygons (areal)	GHDx NID
Sao Tome and Principe	2008–2009	Sao Tome and Principe Demographic and Health Survey 2008–2009	1,719	0	7	26866
Sao Tome and Principe	2014	Sao Tome and Principe Multiple Indicator Cluster Survey 2014	1,965	0	7	214640
Senegal	2000	Senegal Multiple Indicator Cluster Survey 2000	8,703	0	10	27044
Senegal	2005	Senegal Demographic and Health Survey 2005	2,867	360	0	26855
Senegal	2010–2011	Senegal Demographic and Health Survey 2010–2011	3,872	385	0	56063
Senegal	2012–2013	Senegal Continuous Demographic and Health Survey 2012–2013	6,100	200	0	111432
Senegal	2014	Senegal Continuous Demographic and Health Survey 2014	6,025	196	0	191270
Senegal	2015	Senegal Continuous Demographic and Health Survey 2015	6,257	214	0	218592
Senegal	2016	Senegal Continuous Demographic and Health Survey 2016	6,085	214	0	286772
Senegal	2015–2016	Senegal – Dakar Urban Multiple Indicator Cluster Survey 2015–2016*	4,234	0	4	287639
Senegal	2017	Senegal Continuous Demographic and Health Survey 2017*	10,841	0	14	353526
Sierra Leone	2000	Sierra Leone Multiple Indicator Cluster Survey 2000	2,480	0	4	11639
Sierra Leone	2005	Sierra Leone Multiple Indicator Cluster Survey 2005	5,229	0	14	11649
Sierra Leone	2008	Sierra Leone Demographic and Health Survey 2008	2,289	340	0	21258

Country	Survey year(s)	Survey Name	Number of Individuals	Number of geo-positioned clusters	Number of polygons (areal)	GHDx NID
Sierra Leone	2010	Sierra Leone Multiple Indicator Cluster Survey 2010	8,422	0	14	76700
Sierra Leone	2013	Sierra Leone Demographic and Health Survey 2013	4,806	434	0	131467
Sierra Leone	2017	Sierra Leone Multiple Indicator Cluster Survey 2017*	11,785	0	14	218619
Somalia	2006	Somalia Multiple Indicator Cluster Survey 2006	5,867	0	18	11774
South Africa	2002	South Africa – Agincourt Integrated Family Survey 2002	197	0	1	135825
South Africa	2004	South Africa – KwaZulu Natal Income Dynamics Study 2004*	794	0	1	31142
South Africa	2004	South Africa – Agincourt Integrated Family Survey 2004	301	0	1	135826
South Africa	2008	South Africa National Income Dynamics Study – Wave 1 2008	2,161	0	47	27885
South Africa	2010–2011	South Africa National Income Dynamics Study – Wave 2 2010–2011	1,700	0	55	133731
South Africa	2012	South Africa National Income Dynamics Study – Wave 3 2012	3,190	0	52	133732
South Africa	2014–2015	South Africa National Income Dynamics Study – Wave 4 2014–2015	3,878	0	52	265153
South Africa	2016	South Africa Demographic and Health Survey 2016*	1,139	475	0	157064
South Africa	2017	South Africa National Income Dynamics Study – Wave 5 2017*	3,874	0	52	369644
South Sudan	2000	Sudan – South Multiple Indicator Cluster Survey 1999	1,130	0	45	12232

Country	Survey year(s)	Survey Name	Number of Individuals	Number of geo-positioned clusters	Number of polygons (areal)	GHDx NID
South Sudan	2010	Sudan – South Multiple Indicator Cluster Survey 2010	6,807	0	10	32189
Sri Lanka	1999–2000	Sri Lanka Integrated Survey 1999–2000	2,037	455	0	12201
Sudan	2000	Sudan Multiple Indicator Cluster Survey 2000	20,560	0	16	12243
Sudan	2010	Sudan – North Multiple Indicator Cluster Survey 2010	12,296	0	15	153643
Sudan	2014	Sudan Multiple Indicator Cluster Survey 2014	12,999	0	18	200617
Suriname	1999–2000	Suriname Multiple Indicator Cluster Survey 1999–2000	1,811	0	10	12280
Suriname	2006	Suriname Multiple Indicator Cluster Survey 2006	2,017	0	5	12289
Suriname	2010	Suriname Multiple Indicator Cluster Survey 2010	2,859	0	10	81203
Swaziland (eSwatini)	2000	Swaziland Multiple Indicator Cluster Survey 2000	3,411	0	4	12320
Swaziland (eSwatini)	2006–2007	Swaziland Demographic and Health Survey 2006–2007	2,080	268	0	20829
Swaziland (eSwatini)	2010	Swaziland Multiple Indicator Cluster Survey 2010	2,590	0	4	30325
Swaziland (eSwatini)	2014	Swaziland Multiple Indicator Cluster Survey 2014	2,668	0	4	200707
Syria	2006	Syria Multiple Indicator Cluster Survey 2006	10,784	0	60	12399
Syria	2009	Syria Family Health Survey 2009	15,305	0	14	126911†
Tajikistan	2005	Tajikistan Multiple Indicator Cluster Survey 2005	4,239	0	5	12608
Tajikistan	2007	Tajikistan Living Standards Measurement Survey 2007	149	18	0	12584

Country	Survey year(s)	Survey Name	Number of Individuals	Number of geo-positioned clusters	Number of polygons (areal)	GHDx NID
Tajikistan	2012	Tajikistan Demographic and Health Survey 2012	4,568	341	0	74460
Tajikistan	2017	Tajikistan Demographic and Health Survey 2017	5,902	365	0	341838
Tanzania	2004	Tanzania – Kagera Living Standards Measurement Study 2004	1,967	894	0	14341
Tanzania	2004–2005	Tanzania Demographic and Health Survey 2004–2005	7,344	0	26	20875
Tanzania	2006–2007	Tanzania Core Welfare Indicators Questionnaire Survey 2006–2007	9,989	0	28	31831
Tanzania	2009–2010	Tanzania Demographic and Health Survey 2009–2010	6,766	458	0	21331
Tanzania	2010–2011	Tanzania National Panel Survey 2010–2011	2,741	0	126	81005
Tanzania	2012–2013	Tanzania National Panel Survey 2012–2013	3,364	0	135	224096†
Tanzania	2015–2016	Tanzania Demographic and Health Survey 2015–2016	9,104	607	0	218593
Tanzania	2014–2016	Tanzania National Panel Survey 2014–2016	2,463	417	0	311265
Thailand	2005–2006	Thailand Multiple Indicator Cluster Survey 2005–2006	9,207	0	4	12732
Thailand	2012	Thailand Multiple Indicator Cluster Survey 2012	9,246	0	5	148649
Thailand	2015–2016	Thailand Multiple Indicator Cluster Survey 2015–2016	11,368	0	5	296646
Thailand	2016	Thailand – Bangkok Small Community Multiple Indicator Cluster Survey 2016	992	0	1	331377
The Gambia	2000	Gambia Multiple Indicator Cluster Survey 2000	3,465	0	8	3922
The Gambia	2005–2006	Gambia Multiple Indicator Cluster Survey 2005–2006	6,465	0	36	3935
The Gambia	2010	Gambia Multiple Indicator Cluster Survey 2010*	11,574	0	6	91506
The Gambia	2013	Gambia Demographic and Health Survey 2013	3,408	0	37	77384

Country	Survey year(s)	Survey Name	Number of Individuals	Number of geo-positioned clusters	Number of polygons (areal)	GHDx NID
Timor-Leste	2003	Timor-Leste Demographic and Health Survey 2003	5,321	287	92	20888†
Timor-Leste	2007–2008	Timor-Leste Living Standards and Measurement Survey 2007–2008	3,531	0	64	46682†
Timor-Leste	2009–2010	Timor-Leste Demographic and Health Survey 2009–2010	8,494	0	13	21274
Timor-Leste	2016	Timor-Leste Demographic and Health Survey 2016	6,455	455	0	286785
Togo	2006	Togo Multiple Indicator Cluster Survey 2006	4,018	0	6	12896
Togo	2010	Togo Multiple Indicator Cluster Survey 2010	4,711	0	6	40021
Togo	2013	Togo Demographic and Health Survey 2013–2014	3,238	328	0	77515
Trinidad and Tobago	2000	Trinidad and Tobago Multiple Indicator Cluster Survey 2000	806	0	15	12940
Trinidad and Tobago	2011	Trinidad and Tobago Multiple Indicator Cluster Survey 2011	1,122	0	5	332558
Tunisia	2011–2012	Tunisia Multiple Indicator Cluster Survey 2011–2012	2,758	0	9	76709
Turkmenistan	2006	Turkmenistan Multiple Indicator Cluster Survey 2006	2,048	0	6	13064
Turkmenistan	2015–2016	Turkmenistan Multiple Indicator Cluster Survey 2015–2016	3,741	0	6	264583
Uganda	2000–2001	Uganda Demographic and Health Survey 2000–2001	4,791	269	0	20993
Uganda	2006	Uganda Demographic and Health Survey 2006	2,242	333	0	21014
Uganda	2009–2010	Uganda Living Standards Measurement Survey – Integrated Survey on Agriculture 2009–2010	1,476	282	7	81004

Country	Survey year(s)	Survey Name	Number of Individuals	Number of geo-positioned clusters	Number of polygons (areal)	GHDx NID
Uganda	2011	Uganda Demographic and Health Survey 2011	2,097	392	0	56021
Uganda	2010–2011	Uganda Living Standards Measurement Survey – Integrated Survey on Agriculture 2010–2011	1,641	415	0	142934
Uganda	2011–2012	Uganda Living Standards Measurement Survey – Integrated Survey on Agriculture 2011–2012	1,590	455	0	142935
Uganda	2013–2014	Uganda Living Standards Measurement Survey – Integrated Survey on Agriculture 2013–2014	1,611	0	358	264959
Uganda	2016	Uganda Demographic and Health Survey 2016*	4,414	679	0	286780
Uzbekistan	2002	Uzbekistan Special Demographic and Health Survey 2002	2,664	218	0	21039
Uzbekistan	2006	Uzbekistan Multiple Indicator Cluster Survey 2006	4,925	0	6	13445
Vietnam	2000	Vietnam Multiple Indicator Cluster Survey 2000	3,045	0	8	13708
Vietnam	2001–2002	Vietnam National Health Survey 2001–2002	11,225	0	61	44586†
Vietnam	2010–2011	Vietnam Multiple Indicator Cluster Survey 2010–2011	3,615	0	590	57999
Yemen	2005–2006	Yemen Household Budget Survey 2005–2006	12,672	0	495	22882
Yemen	2012	Yemen – Aden Nutritional Status and Mortality Survey 2012	1,120	0	8	244469
Yemen	2012	Yemen – Hajjah Nutritional Status and Mortality Survey 2012	1,300	56	0	244471
Yemen	2012	Yemen – Rayma Nutritional Status and Mortality Survey 2012	639	0	6	244472
Yemen	2012	Yemen – Taiz Nutritional Status and Mortality Survey 2012	904	37	0	244473
Yemen	2012	Yemen – Ibb Nutritional Status and Mortality Survey 2012	1,651	55	0	246249

Country	Survey year(s)	Survey Name	Number of Individuals	Number of geo-positioned clusters	Number of polygons (areal)	GHDx NID
Yemen	2012	Yemen – Lahj Nutritional Status and Mortality Survey 2012	1,560	0	1	246254
Yemen	2013	Yemen Demographic and Health Survey 2013	14,569	0	528	112500†
Yemen	2012–2013	Yemen – Abyan Nutritional Status and Mortality Survey 2012–2013	1,442	32	0	246145
Yemen	2013	Yemen – Dhamar Nutritional Status and Mortality Survey 2013	1,877	40	0	246209†
Yemen	2013	Yemen – Mahweet Nutritional Status and Mortality Survey 2013	1,626	67	0	246250
Yemen	2014	Yemen Comprehensive Food Security Survey 2014	13,426	0	212	244480
Yemen	2014	Yemen – Hajjah Nutritional Status and Mortality Survey 2014	565	60	0	246246
Yemen	2014	Yemen – Hodeidah Nutritional Status and Mortality Survey 2014	1,412	37	0	246248
Yemen	2015	Yemen – Aden Nutritional Status and Mortality Survey 2015	336	0	6	244463
Yemen	2015	Yemen – Al-Baidha Nutritional Status and Mortality Survey 2015	628	27	16	244464
Yemen	2015	Yemen – Hajjah Nutritional Status and Mortality Survey 2015	1,018	45	0	244465
Yemen	2015	Yemen – Hodeidah Nutrition and Mortality Survey 2015	648	0	17	244467
Yemen	2015	Yemen – Lahj Nutritional Status and Mortality Survey 2015	978	0	1	244468
Zambia	2001–2002	Zambia Demographic and Health Survey 2001–2002	5,684	0	72	21102

Country	Survey year(s)	Survey Name	Number of Individuals	Number of geo-positioned clusters	Number of polygons (areal)	GHDx NID
Zambia	2002–2003	Zambia Living Conditions Monitoring Survey 2002–2003	6,603	0	72	14027†
Zambia	2004–2005	Zambia Living Conditions Monitoring Survey 2004–2005	10,642	0	137	14063†
Zambia	2006	Zambia Living Conditions Monitoring Survey 2006	7,446	0	138	14105†
Zambia	2007	Zambia Demographic and Health Survey 2007	5,450	319	0	21117
Zambia	2009	Zambia Access to ACT Initiative Survey 2009	1,332	1,322	0	162031†
Zambia	2010	Zambia Living Conditions Monitoring Survey 2010	10,974	0	72	58660†
Zambia	2013–2014	Zambia Demographic and Health Survey 2013–2014	11,924	719	0	77516
Zimbabwe	2005–2006	Zimbabwe Demographic and Health Survey 2005–2006	4,239	394	0	21163
Zimbabwe	2009	Zimbabwe Multiple Indicator Monitoring Survey 2009	6,282	0	10	35493
Zimbabwe	2010–2011	Zimbabwe Demographic and Health Survey 2010–2011	4,316	393	0	55992
Zimbabwe	2014	Zimbabwe Multiple Indicator Cluster Survey 2014	9,651	0	10	152720
Zimbabwe	2015	Zimbabwe Demographic and Health Survey 2015	5,040	399	0	157066

Supplementary Table 5: Survey Reports added to model.

Number identification (NID) can be used to locate a particular data source in the Global Health Data Exchange (GHDx) at http://ghdx.healthdata.org/.
**Indicates a survey from a country we previously modelled that has been added since the first publication.*
†Data source is not publicly available due to restrictions by the data provider and was used under license for the current study.

Country	Survey year(s)	Survey Name	Number of Individuals	Number of geo-positioned clusters	Number of polygons (areal)	GHDx NID
Burkina Faso	2009	Burkina Faso National Nutrition Survey 2009*	265,498	0	43	56884
Burkina Faso	2016	Burkina Faso National Nutrition Survey 2016*	108,449	0	13	299307
China	2002	China National Nutrition Survey 2002 – China CDC*	27,525	0	128	124479†
China	2009–2015	Secular Trends in Growth and Nutritional Outcomes of Children under Five Years Old in Xiamen, China*	71,229	0	3	398585
Mauritania	2012	Mauritania National Nutrition Survey Using SMART Methodology July 2012*	163,663	0	11	275121
Mauritania	2014	Mauritania National Nutrition Survey Using the SMART Methodology August 2014*	166,139	0	11	275123
Mongolia	2004	Mongolia National Nutrition Survey 2004*	45,316	0	21	137528†
Niger	2007	Niger Nutrition and Child Survival Survey 2007*	454,450	0	8	160103
Niger	2008	Niger Nutrition and Child Survival Survey 2008*	276,775	0	8	160198
Niger	2009	Niger Nutrition and Child Survival Survey 2009*	470,951	0	8	160053
Niger	2011	Niger Nutrition and Child Survival Survey 2011*	489,926	0	8	316438
Niger	2012	Niger Nutrition and Child Survival Survey 2012*	597,220	0	8	316440
Niger	2013	Niger Nutrition and Child Survival Survey 2013*	796,291	0	8	316442
Niger	2014	Niger Nutrition and Child Survival Survey 2014*	975,149	0	8	316444
Nigeria	2010	Nigeria Standardized Monitoring and Assessment of Relief and Transitions Survey, December 2010*	73,134	0	8	151724†

Country	Survey year(s)	Survey Name	Number of Individuals	Number of geo-positioned clusters	Number of polygons (areal)	GHDx NID
Nigeria	2011	Nigeria Standardized Monitoring and Assessment of Relief and Transitions Survey, July–August 2011*	88,246	0	7	151725†
Nigeria	2012	Nigeria Standardized Monitoring and Assessment of Relief and Transitions Survey, February–March 2012*	104,742	0	8	151727†
Nigeria	2014	Nigeria National Nutrition and Health Survey 2014*	260,912	0	37	274708
Nigeria	2015	Nigeria National Nutrition and Health Survey 2015*	264,297	0	37	274707
Pakistan	2007–2008	Pakistan – Punjab Multiple Indicator Cluster Survey 2007–2008*	71,721	0	175	387340
Philippines	2003	Philippines National Nutrition Survey 2003*	34,891	0	17	124455
Sri Lanka	2006–2007	Sri Lanka Demographic and Health Survey 2006–2007*	10,969	0	20	18815
Sri Lanka	2009	Sri Lanka Nutrition and Food Security Assessment 2009*	4,753	0	10	141592
Sri Lanka	2012	Sri Lanka National Nutrition and Micronutrient Survey 2012*	12,337	0	25	153000
Sri Lanka	2016	Sri Lanka Demographic and Health Survey 2016*	13,528	0	25	326837
Swaziland (eSwatini)	2008	Swaziland National Nutrition Survey 2008*	10,376	0	4	141312
Tajikistan	2001	Tajikistan National Nutrition Survey 2001*	46,075	0	4	141797
Thailand	2015–2016	Thailand 14 Provinces Multiple Indicator Cluster Survey 2015–2016*	32,485	0	14	317305
Tunisia	2000	Tunisia Multiple Indicator Cluster Survey 2000*	78,685	0	6	12983
Tunisia	2006	Tunisia Multiple Indicator Cluster Survey 2006*	24,013	0	9	12985
Turkmenistan	2000	Turkmenistan Demographic and Health Survey 2000*	148,724	0	6	20956
Venezuela	2016	Venezuela – Capital District, Vargas, Miranda and Zulia Baseline of Sentinel Monitoring of Nutrition Status in Children Under 5 Years, SAMAN System October–December 2016*	8,622	0	4	289278

Country	Survey year(s)	Survey Name	Number of Individuals	Number of geo-positioned clusters	Number of polygons (areal)	GHDx NID
Vietnam	2009	Vietnam Sanitation, Water Supply, and Child Nutrition Survey 2009*	10,093	0	6	152424
Vietnam	2009–2010	Vietnam General Nutrition Survey 2009–2010*	286,085	0	63	152422
Vietnam	2011	Vietnam Annual National Nutrition Monitoring 2011*	292,176	0	63	293979†
Vietnam	2012	Vietnam Nutrition Surveillance 2012*	297,529	0	63	286277
Vietnam	2012	Vietnam Annual National Nutrition Monitoring 2012*	293,659	0	63	293980†
Vietnam	2013	Vietnam Annual National Nutrition Monitoring 2013*	299,685	0	63	293981†
Vietnam	2014	Vietnam Annual National Nutrition Monitoring 2014*	98,433	0	63	293982†

Supplementary Table 6: Data excluded from model.

Country	Survey year(s)	Survey Name	Reason	GHDx NID
Afghanistan	2015–2016	Afghanistan Demographic and Health Survey 2015–2016	Age data is insufficiently granular (for calculations of height-for-age and weight-for-age z-scores)	157018
Bangladesh	2010–2013	Bangladesh – Dhaka Malnutrition and Enteric Disease Study 2009–2014	Anthropometric measurements not taken of a full set of under-fives	261683
Bangladesh	2000	Bangladesh – Khulna and Dhaka Supporting Household Activities for Health, Assets, and Revenue Survey 2000	Data does not have interview dates or age in months	153154
Bangladesh	2015	Bangladesh Integrated Household Survey 2015	Data does not have interview dates or age in months, insufficient sample weight data	283269
Bangladesh	2011–2012	Bangladesh Integrated Household Survey 2011–2012	Insufficient sample weight data	153062
Bangladesh	2002	Bangladesh – Dinajpur Supporting Household Activities for Health, Assets, and Revenue Survey, Round 1 2002	Insufficient sample weight data	163056
Bangladesh	2007–2011	Bangladesh – Mizrapur Global Enteric Multicenter Study 2007–2011	Insufficient sample weight data	224248
Bangladesh	2011–2013	Bangladesh – Mizrapur Global Enteric Multicenter Study 2011–2013	Insufficient sample weight data	224855
Bangladesh	2003	Bangladesh – Dinajpur Supporting Household Activities for Health, Assets, and Revenue Survey, Round 2 2003	Insufficient sample weight data	231832
Bangladesh	2003	Bangladesh – Dinajpur Supporting Household Activities for Health, Assets, and Revenue Survey, Round 3 2003	Insufficient sample weight data	231835
Bangladesh	2014–2017	Bangladesh – Dhaka Cohort Study of Cryptosporidiosis in Children 2014	Insufficient sample weight data	263389
Benin	2011–2012	Benin Demographic and Health Survey 2011–2012	Prevalence values for indicators were determined to be implausible	79839

Country	Survey year(s)	Survey Name	Reason	GHDx NID
Brazil	2010–2013	Brazil – Fortaleza Malnutrition and Enteric Disease Study 2009–2014	Anthropometric measurements not taken of a full set of under-fives	261873
Burkina Faso	2005	Burkina Faso Core Welfare Indicators Questionnaire Survey 2005	National prevalence values reported for one or more indicators were determined to be implausibly high based on country-level trend seen in 8 other Burkina Faso sources.	22950
Cameroon	2001	Cameroon Household Survey 2001	Anthropometric measurements not taken of a full set of under-fives	2039
Egypt	2015	Egypt Special Demographic and Health Survey 2015	Non-proportional sample allocation designed to estimate the prevalence of hepatitis and certain other NCD risk factors, such that the survey sampling was not comparable to the other surveys.	157026
Ethiopia	2004	Ethiopia Rural Household Survey 2004	Age data is insufficiently granular (for calculations of height-for-age and weight-for-age z-scores)	38496
Ghana	2009–2010	Ghana Socioeconomic Panel Survey 2009–2010	National prevalence values reported for one or more indicators were determined to be implausibly high based on country-level trend seen in 8 other country-level Ghana sources.	236205
India	2010–2013	India – Vellore Malnutrition and Enteric Disease Study 2009–2014	Anthropometric measurements not taken of a full set of under-fives	261875
India	2007–2008	India Tribal Second Repeat Survey of Diet and Nutritional Status 2007–2008	Geographies could not be mapped	129783
Kenya	2004	Kenya Greater Eldoret Health and Development Survey 2004	Insufficient sample weight data	152561
Kenya	2005	Kenya Greater Eldoret Health and Development Survey 2005	Insufficient sample weight data	152562
Kenya	2006	Kenya Greater Eldoret Health and Development Survey 2006	Insufficient sample weight data	152563

Country	Survey year(s)	Survey Name	Reason	GHDx NID
Kenya	2008–2011	Kenya – Nyanza Global Enteric Multicenter Study 2008–2011	Insufficient sample weight data	224239
Kenya	2011–2012	Kenya – Nyanza Global Enteric Multicenter Study 2011–2012	Insufficient sample weight data	224853
Lebanon	2005–2006	Palestinians in Lebanon Multiple Indicator Cluster Survey 2005–2006	Data is not representative of its geography	7688
Lebanon	2011	Palestinians in Lebanon Multiple Indicator Cluster Survey 2011	Data is not representative of its geography	76708
Mali	2007–2011	Mali – Bamako Global Enteric Multicenter Study 2007–2011	Insufficient sample weight data	224233
Mali	2011–2013	Mali – Bamako Global Enteric Multicenter Study 2011–2013	Insufficient sample weight data	224848
Mexico	2002	Mexico Family Life Survey 2002	Age data is insufficiently granular (for calculations of height-for-age and weight-for-age z-scores)	8442
Mexico	2015	Mexico Multiple Indicator Cluster Survey 2015	Geographies could not be mapped	264590
Mozambique	2007–2011	Mozambique – Manhica Global Enteric Multicenter Study 2007–2011	Insufficient sample weight data	224236
Mozambique	2011–2013	Mozambique – Manhica Global Enteric Multicenter Study 2011–2013	Insufficient sample weight data	224849
Nepal	2010–2013	Nepal – Bhaktapur Malnutrition and Enteric Disease Study 2009–2014	Anthropometric measurements not taken of a full set of under-fives	261880
Nigeria	2008–2010	Nigeria Living Standards Survey 2008–2010	Age data is insufficiently granular (for calculations of height-for-age and weight-for-age z-scores)	151719
Nigeria	2010–2011	Nigeria General Household Survey 2010–2011	Age data is insufficiently granular (for calculations of height-for-age and weight-for-age z-scores)	151802
Nigeria	2006	Nigeria Core Welfare Indicators Questionnaire Survey 2006	Insufficient sample weight data	9522

Country	Survey year(s)	Survey Name	Reason	GHDx NID
Nigeria	2011	Nigeria – Akwa Ibom Survey on Dietary Intakes, Vitamin A, and Iron Status of Women of Childbearing Age and Children 6–59 Months of Age 2011	Insufficient sample weight data	283272
Pakistan	2010–2013	Pakistan – Naushahro Feroze Malnutrition and Enteric Disease Study 2009–2014	Anthropometric measurements not taken of a full set of under-fives	261883
Pakistan	2008–2011	Pakistan – Karachi Global Enteric Multicenter Study 2008–2011	Insufficient sample weight data	224251
Pakistan	2011–2013	Pakistan – Karachi Global Enteric Multicenter Study 2011–2013	Insufficient sample weight data	224856
Peru	2010–2013	Peru – Loreto Malnutrition and Enteric Disease Study 2009–2014	Anthropometric measurements not taken of a full set of under-fives	261879
Rwanda	2009	Rwanda Comprehensive Food Security and Vulnerability Assessment and Nutrition Survey 2009	Anthropometric measurements not taken of a full set of under-fives	58188
Somalia	2007	Somalia Nutrition Surveillance and Assessment 2007	Data is duplicative	358676
Somalia	2008	Somalia Nutrition Surveillance and Assessment 2008	Data is duplicative	358679
Somalia	2009	Somalia Nutrition Surveillance and Assessment 2009	Data is duplicative	358680
Somalia	2010	Somalia Nutrition Surveillance and Assessment 2010	Data is duplicative	358681
Somalia	2001	Somalia Nutrition Surveillance and Assessment 2001–2006	Geographies could not be mapped	358670-358675
South Africa	2010–2013	South Africa – Venda Malnutrition and Enteric Disease Study 2009–2014	Anthropometric measurements not taken of a full set of under-fives	261887
South Sudan	2009	Sudan – South National Baseline Household Survey (NBHS) 2009	Data does not have interview dates or age in months	30368
Tanzania	2008–2009	Tanzania National Panel Survey 2008–2009	Age data is insufficiently granular (for calculations of height-for-age and weight-for-age z-scores)	27297

Country	Survey year(s)	Survey Name	Reason	GHDx NID
Tanzania	2004	Tanzania – Shinyanga Core Welfare Indicators Questionnaire Survey 2004	Age data is insufficiently granular (for calculations of height-for-age and weight-for-age z-scores)	31786
Tanzania	2005	Tanzania Core Welfare Indicators Questionnaire Survey 2005	Age data is insufficiently granular (for calculations of height-for-age and weight-for-age z-scores)	31797
Tanzania	2010–2013	Tanzania – Haydom Malnutrition and Enteric Disease Study 2009–2014	Anthropometric measurements not taken of a full set of under-fives	261889
Tanzania	2010	Tanzania – Kagera Living Standards Measurement Study 2010	Data is not representative of its geography	93807
The Gambia	2007–2011	Gambia – Basse Global Enteric Multicenter Study 2007–2011	Insufficient sample weight data	222752
The Gambia	2011–2013	Gambia – Basse Global Enteric Multicenter Study 2011–2013	Insufficient sample weight data	223566

2.3 Data Process

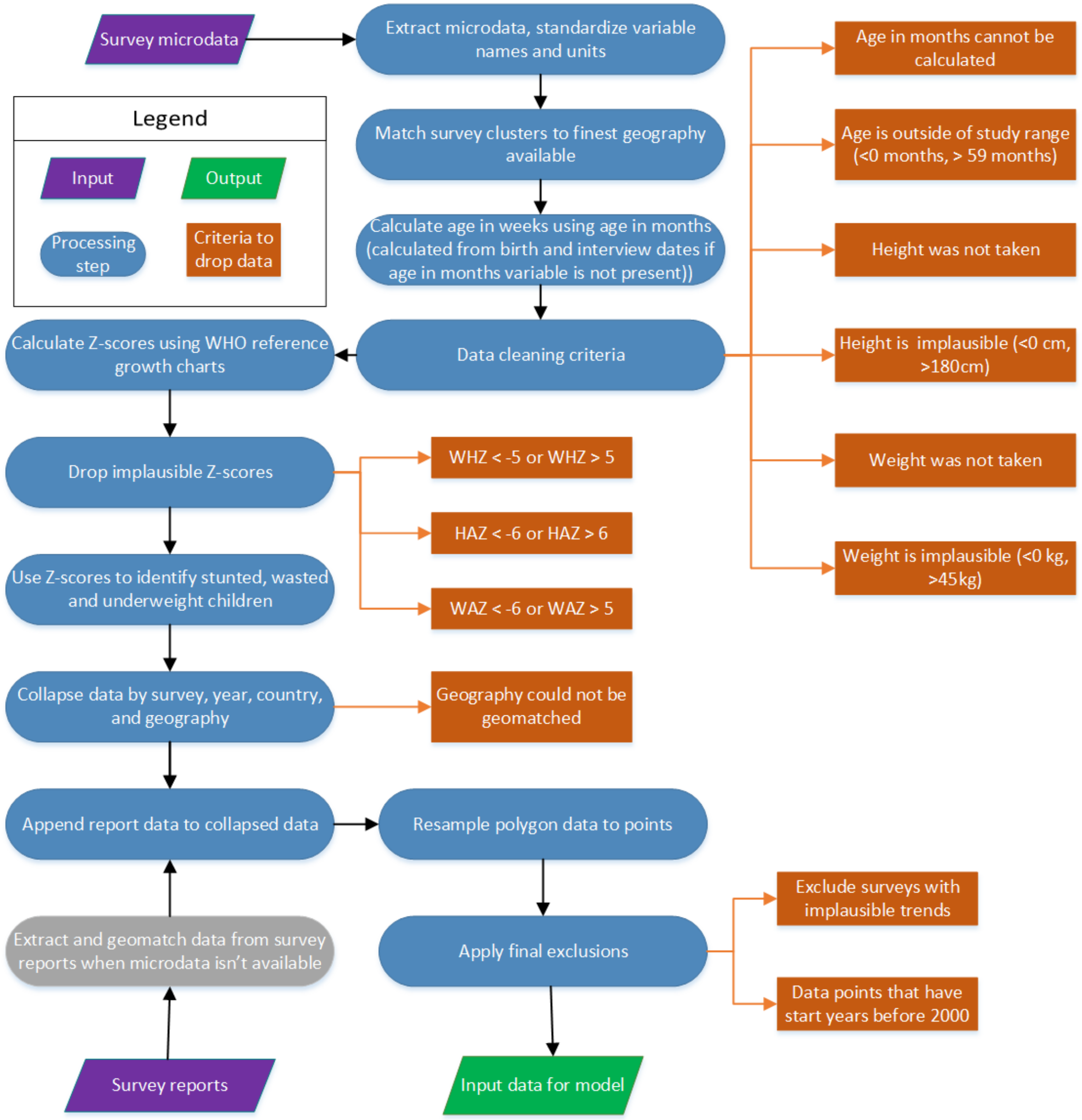

Supplementary Figure 1: Flowchart for data processes.
The data processing pipeline began with raw survey microdata and ended with the input data for the model. We extracted and standardised names and measurement units of relevant CGF data and matched the corresponding survey clusters with the finest geographies possible. Observations (representing children) were dropped due to insufficient or implausible data for age, height (≤0 cm or ≥180 cm), or weight (≤0 kg or ≥45 kg). We calculated z-scores using the height, weight, and age data, and implausible z-scores[3,29,30] (according to WHO reference population[28]) were dropped; 3.30% of children were dropped due to implausible HAZ scores (<-6 or >6), 2.37% due to implausible WHZ scores (<-5 or >5), and 1.09% due to implausible WAZ scores (<-6 or >5). Children that met the definitions of stunted, wasted, or underweight were identified, and data collapsed by survey, year, and geography. Children that could not be matched to a geography were also dropped. Survey reports that were manually extracted at an aggregated level, most often at the first administrative (Admin 1) or second administrative (Admin 2) level, were appended to the collapsed data, and all data attributed to a polygon were resampled to points. After examining diagnostic plots, a small number of surveys were dropped for exhibiting implausible trends. The final cleaned and vetted data were used as input data for the modelling.

2.4 Data Availability by Region

Supplementary Figures 2–16 show the data availability for stunting, wasting, and underweight indicators in the regions we modelled. We incorporated data from a number of survey series, which are represented in the figures. These included: the Demographic and Health Survey by Macro International (Macro DHS), the Multiple Indicator Cluster Survey by the United Nations International Children's Emergency Fund (UNICEF MICS), the Pan Arab Programme on Family Health survey by League of Arab States (PAPFAM), the Core Welfare Indicators Questionnaire by World Bank (CWQI), the Living Standards Measurement Study by World Bank (LSMS) and the Integrated Surveys on Agriculture (LSMS ISA), the Priority Survey series by World Bank (Priority Survey), the Reproductive Health Survey by Center for Disease Control (CDC RHS), the Demographic and Health Survey for Pacific countries funded by the Asian Development Bank (ADB DHS), the Family Life Survey by RAND Corporation (RAND FLS), the Global Enteric Multicenter Study by the Center for Vaccine Development (CVD GEMS), and a number of other country-specific surveys that were not clearly associated with an international survey series (Other). We greatly reduced our risk of incorporating duplicative data in our model by using almost exclusively raw survey microdata. We regularly monitored larger survey series such as Macro DHS and UNICEF MICS for new data, and worked with GBD collaborators from LMICs to identify and obtain other surveys with useful data. While it is possible that there was some overlap in patient populations for surveys that were conducted in the same countries and similar time periods, it is not possible to identify if and how frequently this occurred.

The database for stunting consists of 142,468 clusters and 18,010 polygons with a sample size totaling over 3.9 million children in LMICs.
The database for wasting consists of 142,017 clusters and 17,997 polygons with a sample size totaling over 3.9 million children in LMICs.
The database for underweight consists of 142,528 clusters and 18,192 polygons with a sample size of over 4.0 million children in LMICs.

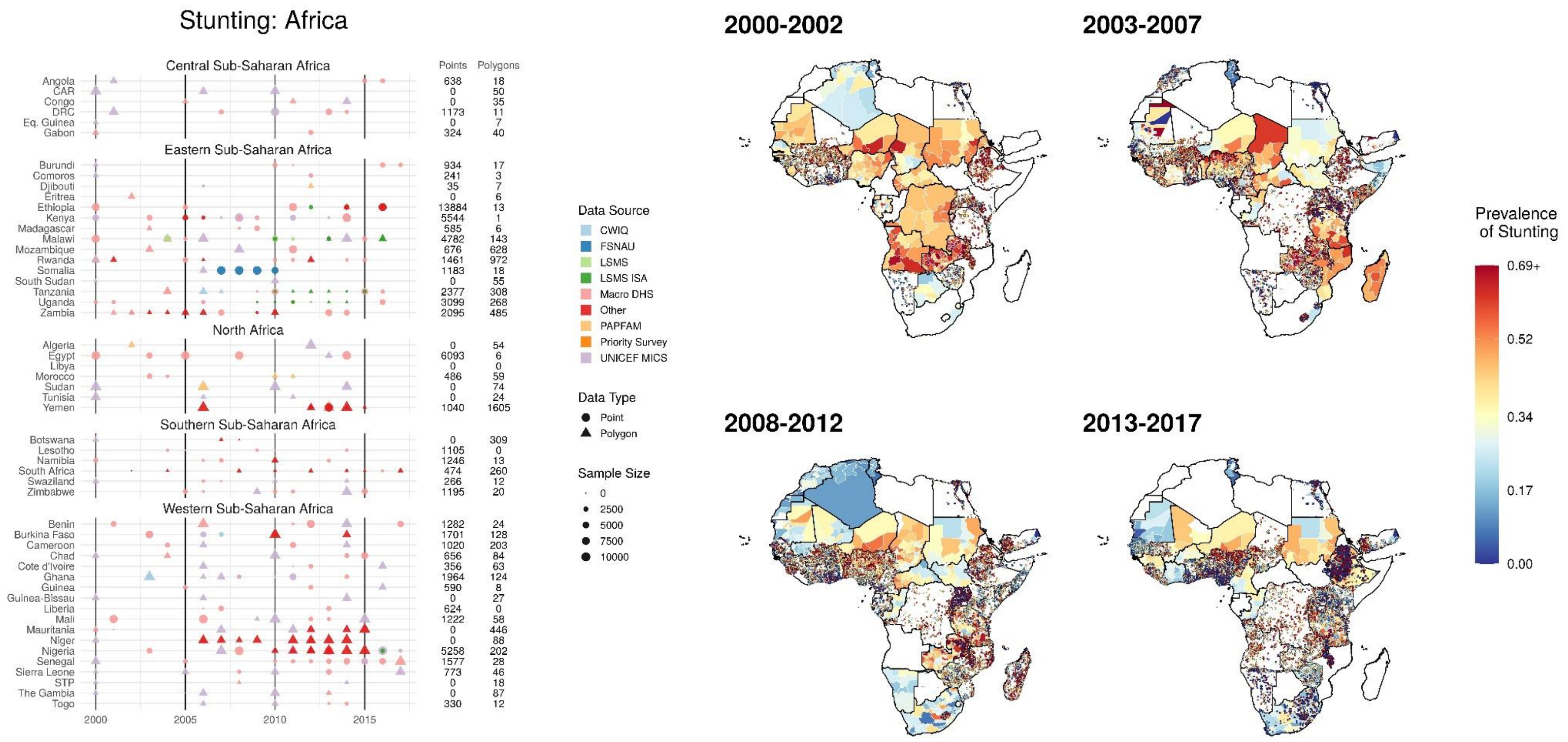

Supplementary Figure 2: Stunting data availability by type and country, 2000–2017 in Africa.

All data are shown by country and year of survey and mapped at their corresponding geo-positioned coordinate or area. The total number of points and polygons (areal) for each country are plotted by data source, type, and sample size. Sample size represents the number of individual microdata records for each survey. Mean stunting prevalence of the input coordinate or area is mapped.

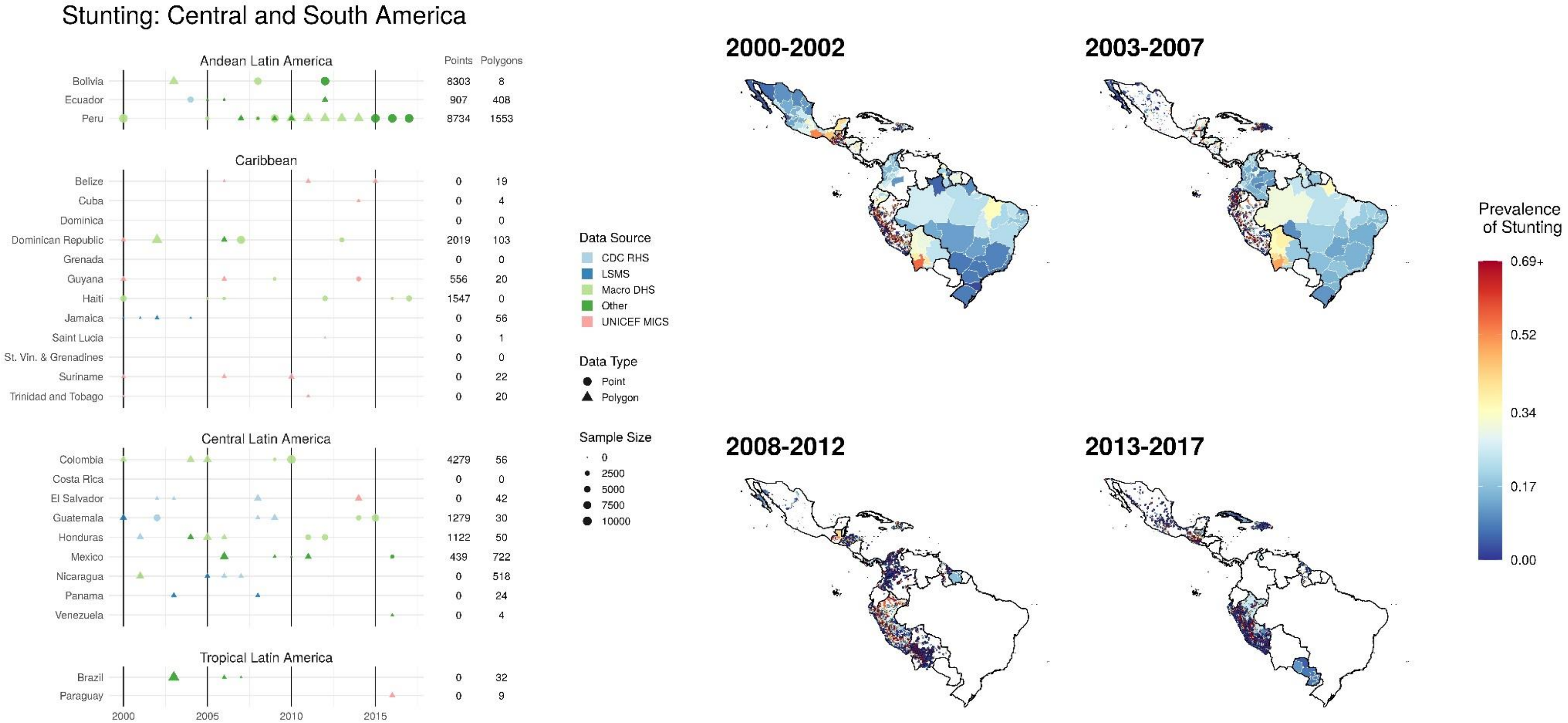

Supplementary Figure 3: Stunting data availability by type and country, 2000–2017 in Central America and the Caribbean and South America.

All data are shown by country and year of survey and mapped at their corresponding geo-positioned coordinate or area. The total number of points and polygons (areal) for each country are plotted by data source, type, and sample size. Sample size represents the number of individual microdata records for each survey. Mean stunting prevalence of the input coordinate or area is mapped.

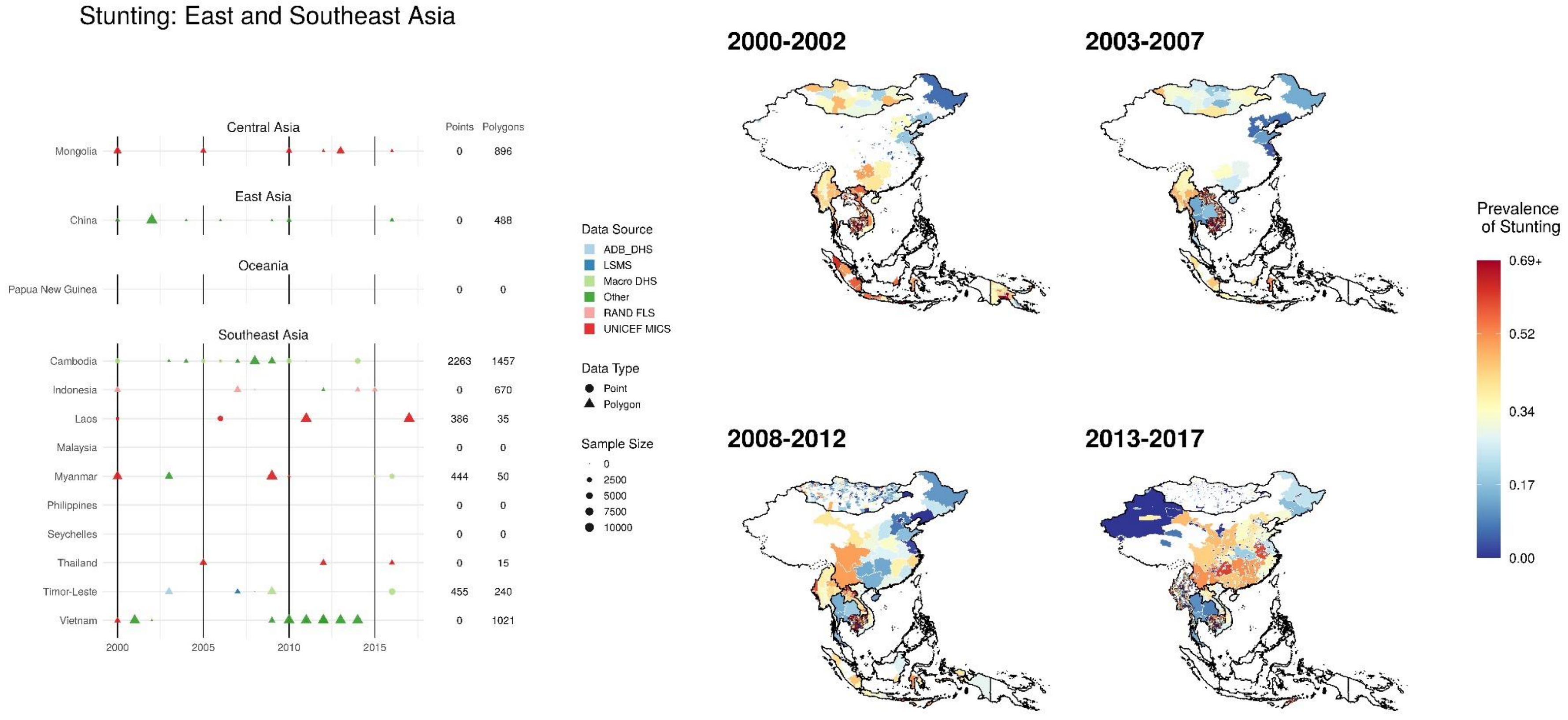

Supplementary Figure 4: Stunting data availability by type and country, 2000–2017 in East and Southeast Asia.

All data are shown by country and year of survey and mapped at their corresponding geo-positioned coordinate or area. The total number of points and polygons (areal) for each country are plotted by data source, type, and sample size. Sample size represents the number of individual microdata records for each survey. Mean stunting prevalence of the input coordinate or area is mapped.

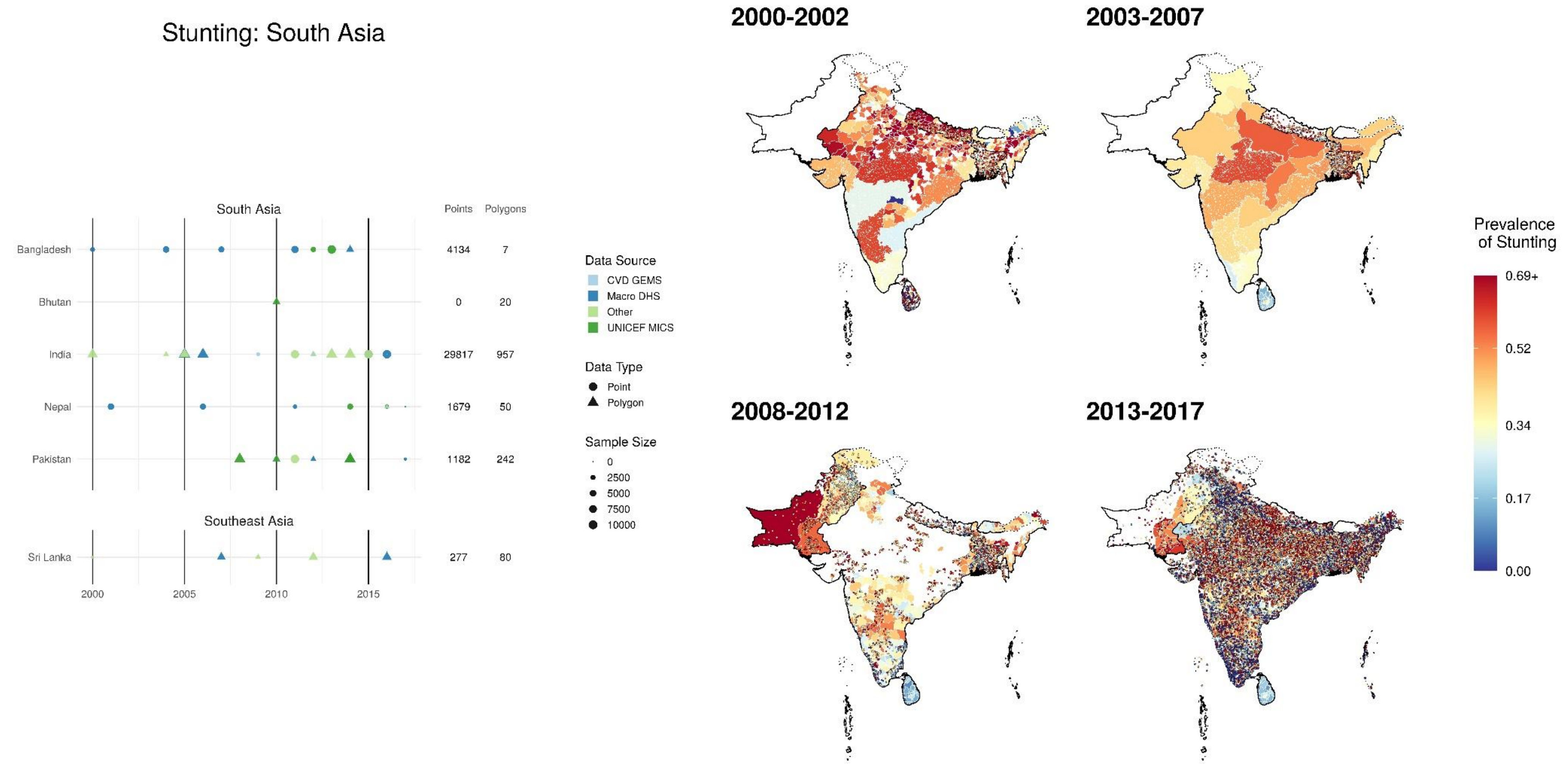

Supplementary Figure 5: Stunting data availability by type and country, 2000–2017 in South Asia.

All data are shown by country and year of survey and mapped at their corresponding geo-positioned coordinate or area. The total number of points and polygons (areal) for each country are plotted by data source, type, and sample size. Sample size represents the number of individual microdata records for each survey. Mean stunting prevalence of the input coordinate or area is mapped.

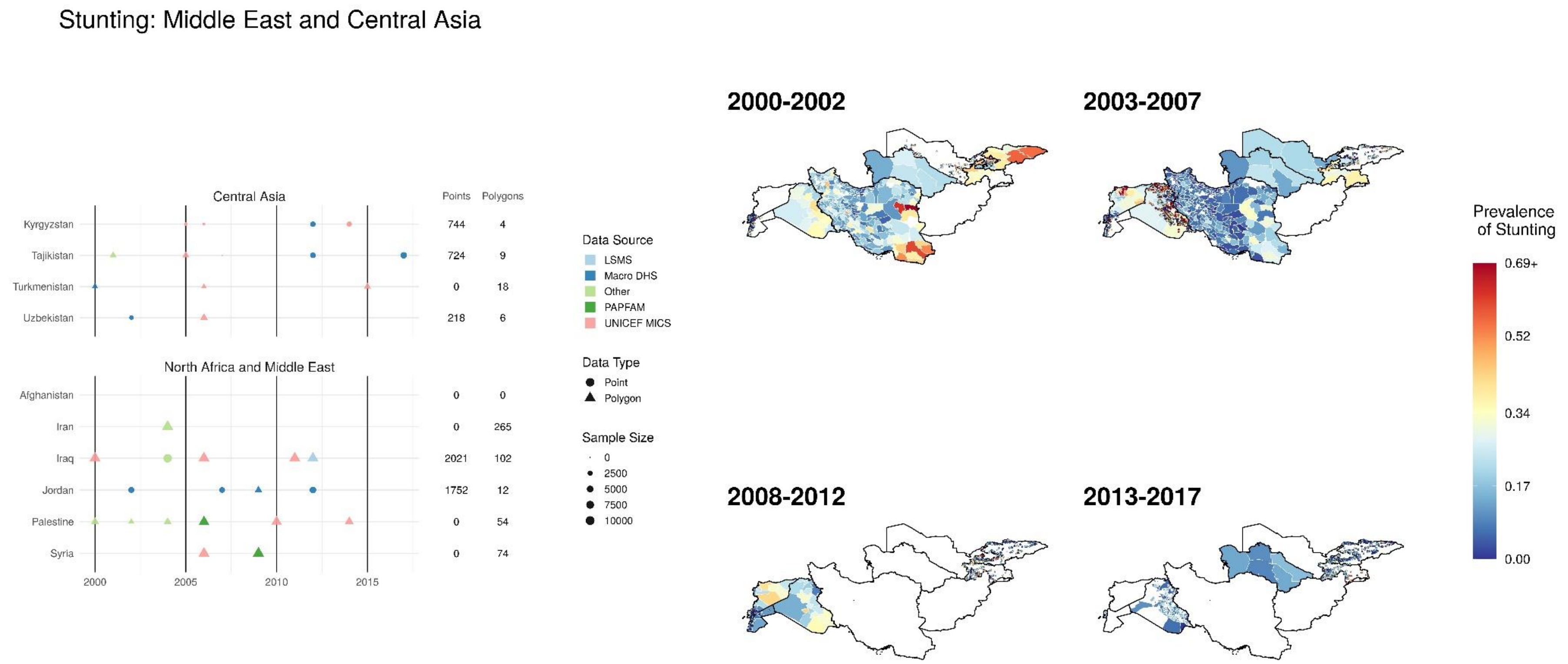

Supplementary Figure 6: Stunting data availability by type and country, 2000–2017 in Middle East and Central Asia.
All data are shown by country and year of survey and mapped at their corresponding geo-positioned coordinate or area. The total number of points and polygons (areal) for each country are plotted by data source, type, and sample size. Sample size represents the number of individual microdata records for each survey. Mean stunting prevalence of the input coordinate or area is mapped.

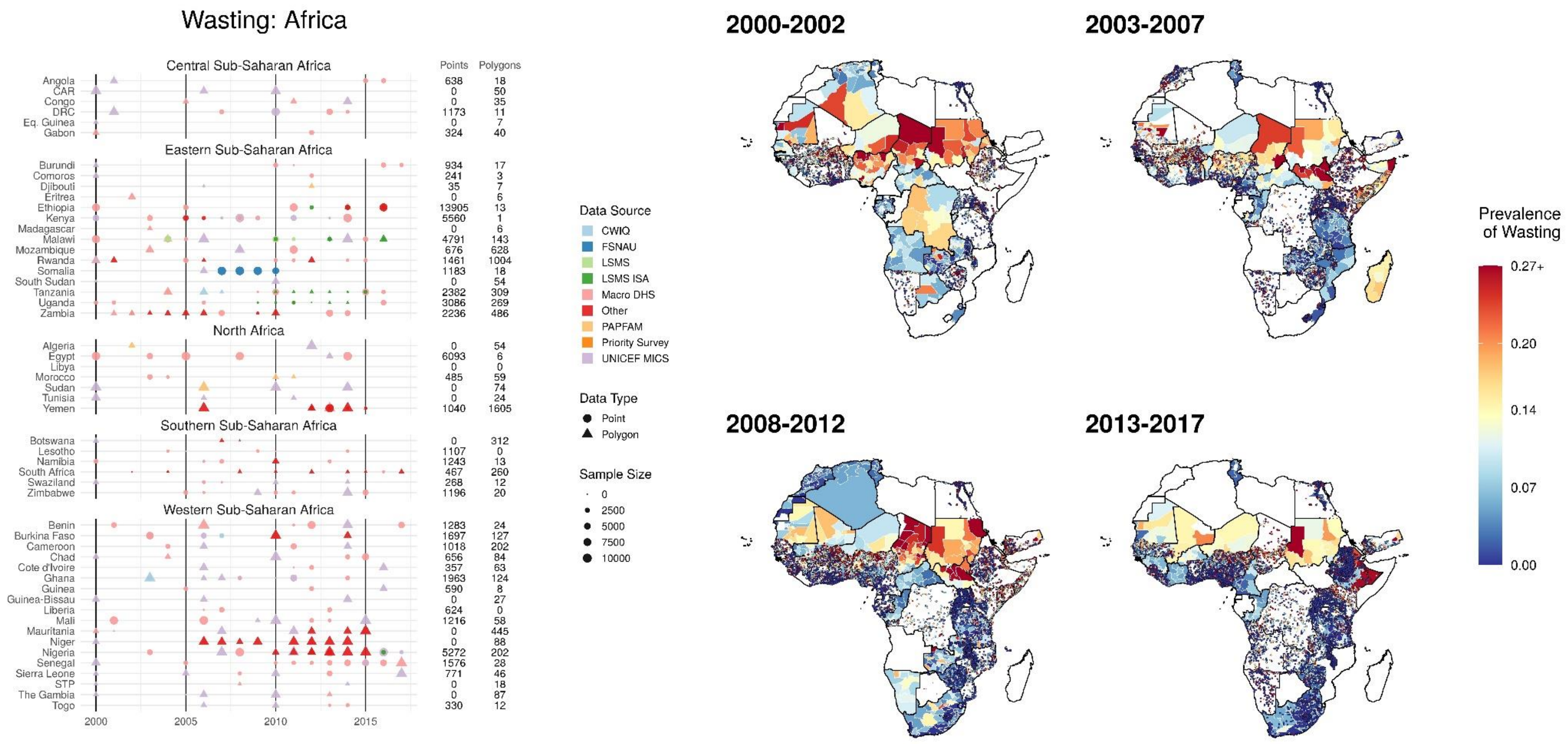

Supplementary Figure 7: Wasting data availability by type and country, 2000–2017 in Africa.

All data are shown by country and year of survey and mapped at their corresponding geo-positioned coordinate or area. The total number of points and polygons (areal) for each country are plotted by data source, type, and sample size. Sample size represents the number of individual microdata records for each survey. Mean wasting prevalence of the input coordinate or area is mapped.

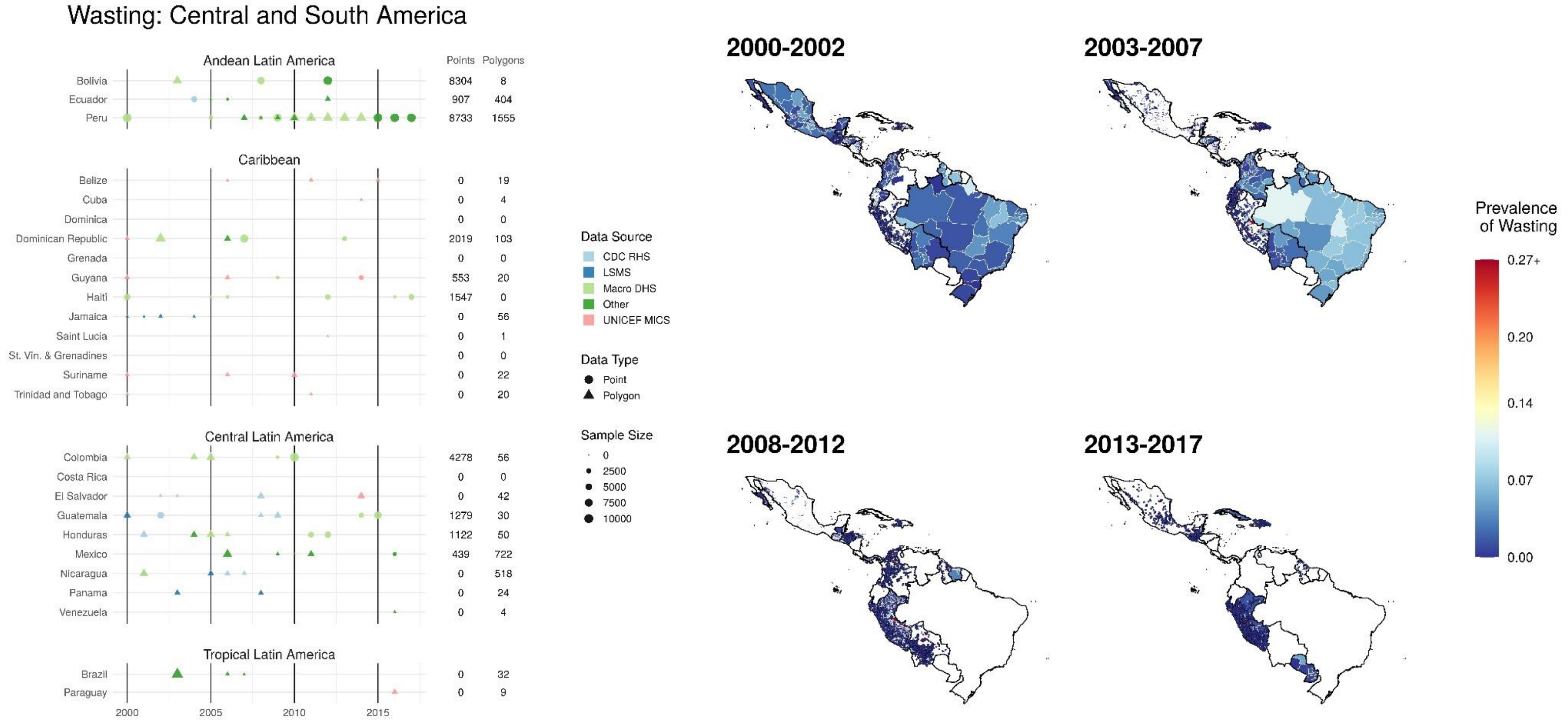

Supplementary Figure 8: Wasting data availability by type and country, 2000–2017 in Central America and the Caribbean and South America.

All data are shown by country and year of survey and mapped at their corresponding geo-positioned coordinate or area. The total number of points and polygons (areal) for each country are plotted by data source, type, and sample size. Sample size represents the number of individual microdata records for each survey. Mean wasting prevalence of the input coordinate or area is mapped.

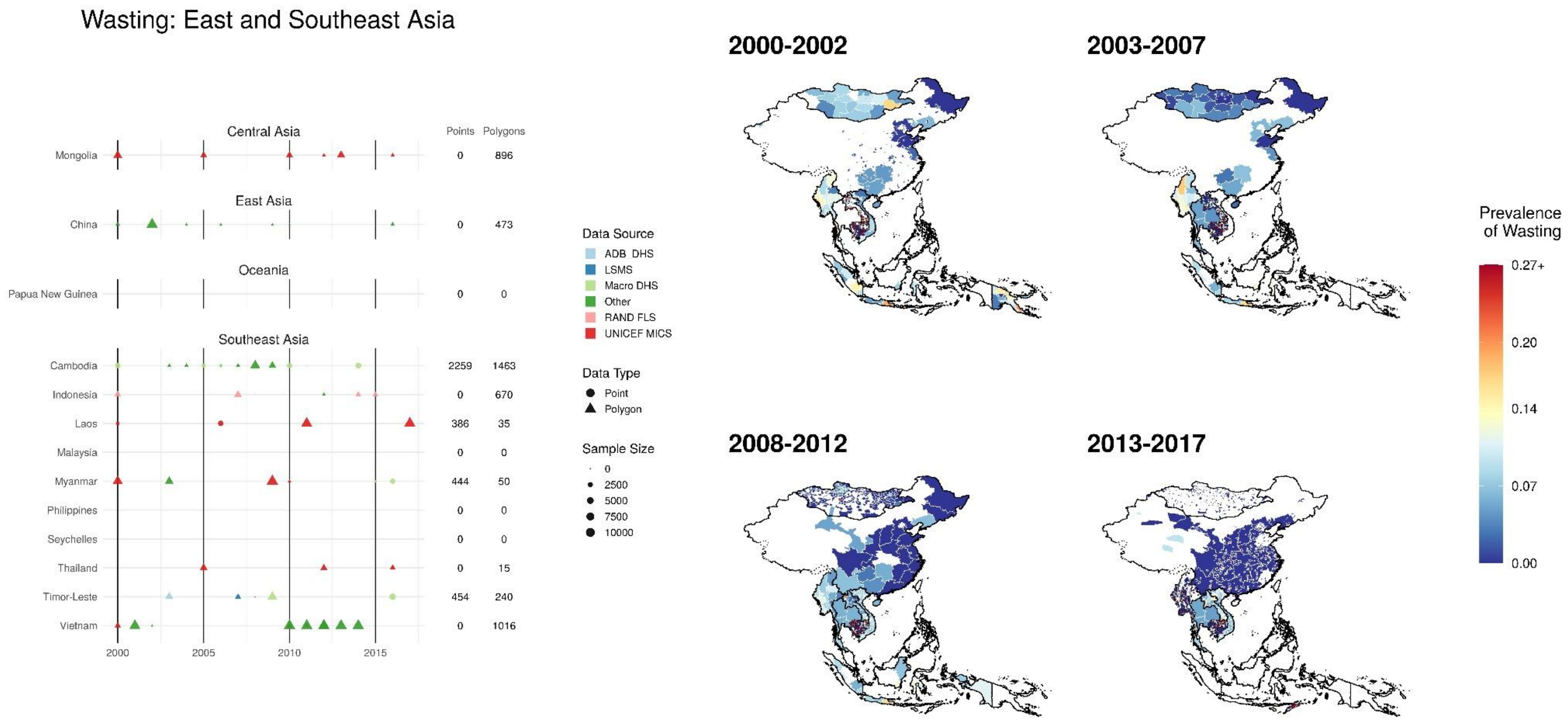

Supplementary Figure 9: Wasting data availability by type and country, 2000–2017 in East and Southeast Asia.
All data are shown by country and year of survey and mapped at their corresponding geo-positioned coordinate or area. The total number of points and polygons (areal) for each country are plotted by data source, type, and sample size. Sample size represents the number of individual microdata records for each survey. Mean wasting prevalence of the input coordinate or area is mapped.

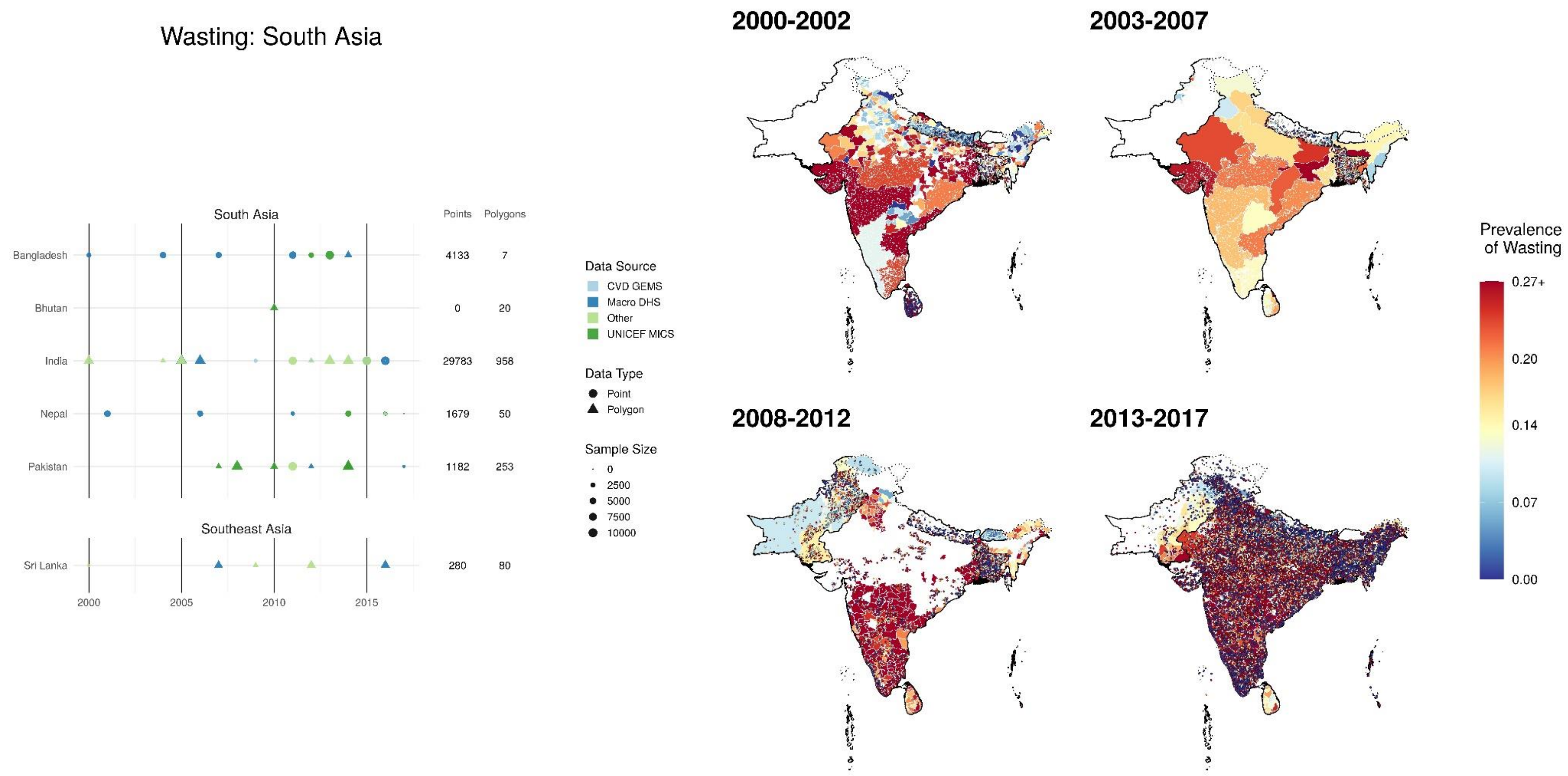

Supplementary Figure 10: Wasting data availability by type and country, 2000–2017 in South Asia.

All data are shown by country and year of survey and mapped at their corresponding geo-positioned coordinate or area. The total number of points and polygons (areal) for each country are plotted by data source, type, and sample size. Sample size represents the number of individual microdata records for each survey. Mean wasting prevalence of the input coordinate or area is mapped.

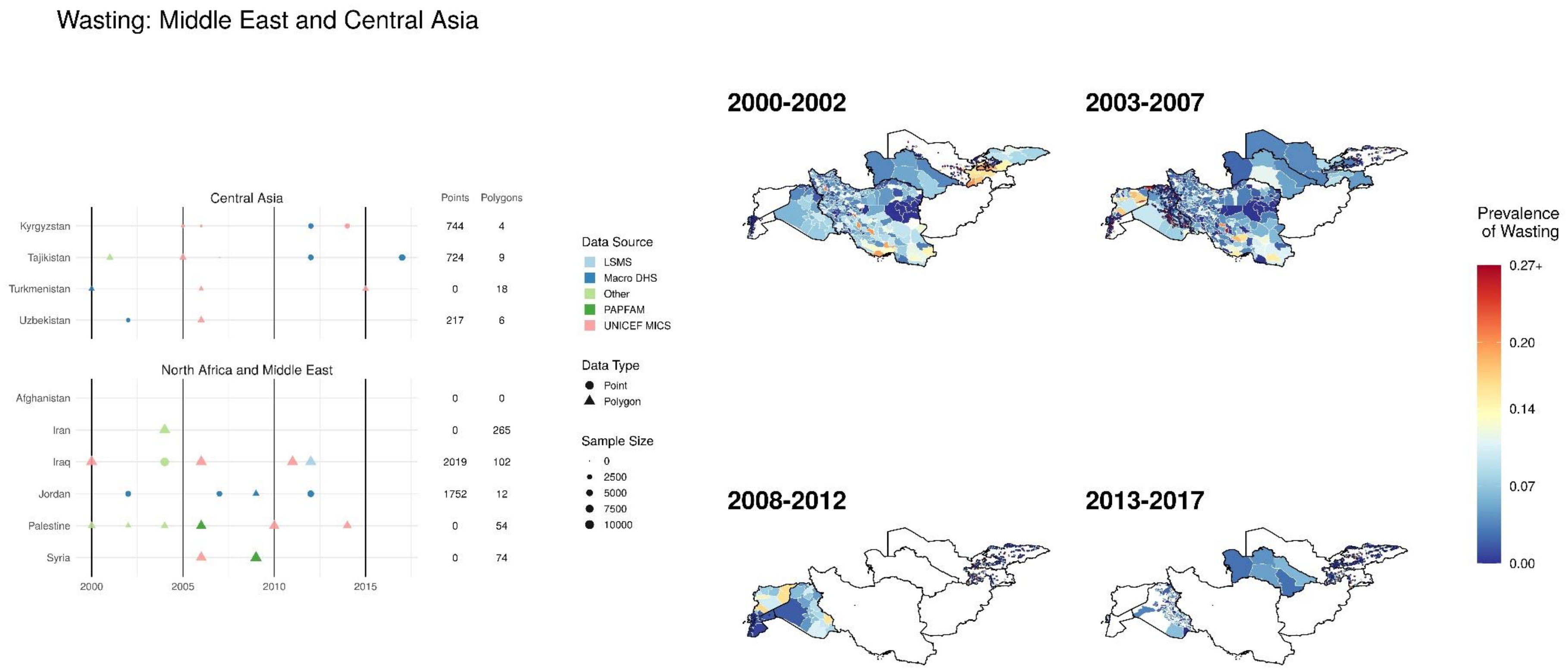

Supplementary Figure 11: Wasting data availability by type and country, 2000–2017 in Middle East and Central Asia.
All data are shown by country and year of survey and mapped at their corresponding geo-positioned coordinate or area. The total number of points and polygons (areal) for each country are plotted by data source, type, and sample size. Sample size represents the number of individual microdata records for each survey. Mean wasting prevalence of the input coordinate or area is mapped.

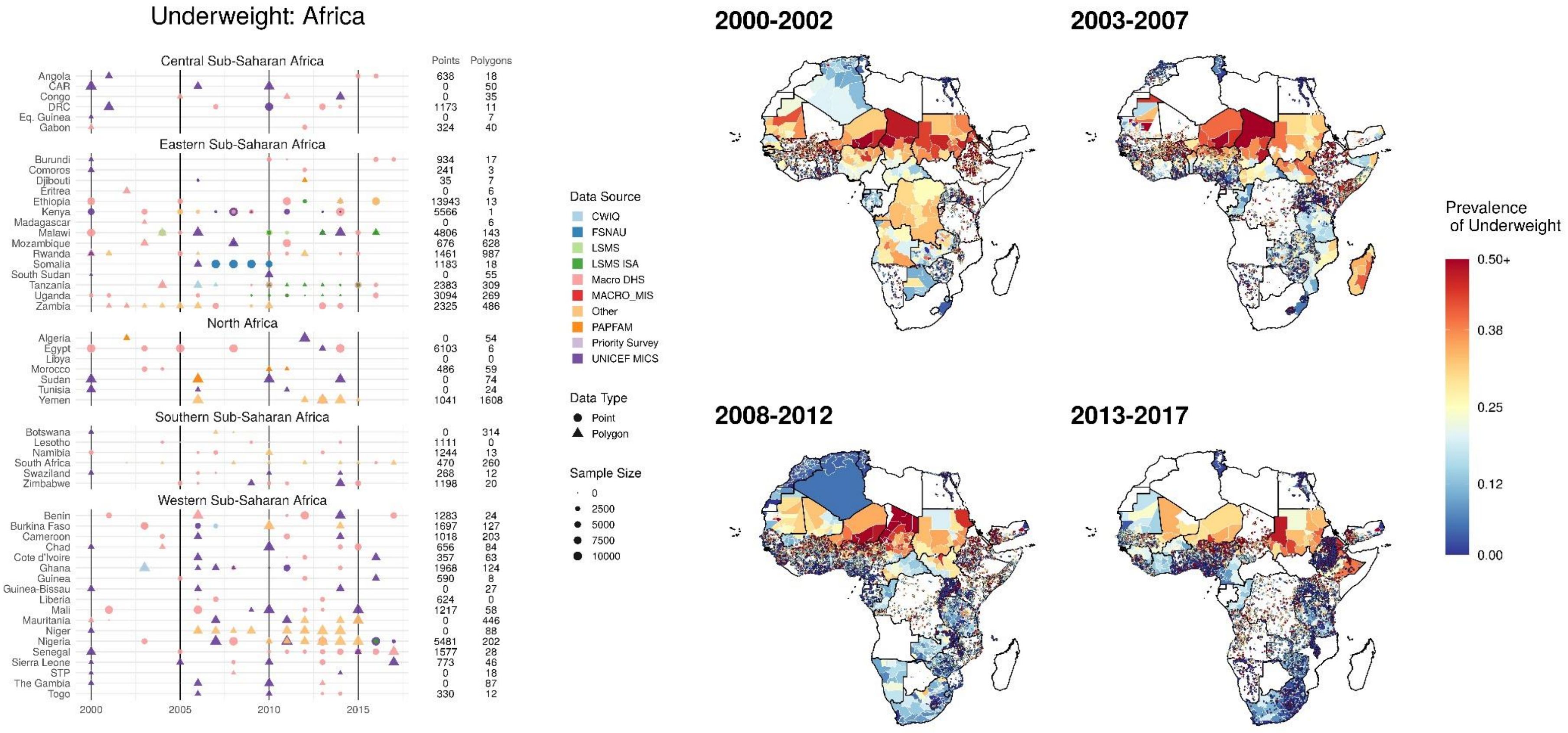

Supplementary Figure 12: Underweight data availability by type and country, 2000–2017 in Africa.

All data are shown by country and year of survey and mapped at their corresponding geo-positioned coordinate or area. The total number of points and polygons (areal) for each country are plotted by data source, type, and sample size. Sample size represents the number of individual microdata records for each survey. Mean underweight prevalence of the input coordinate or area is mapped.

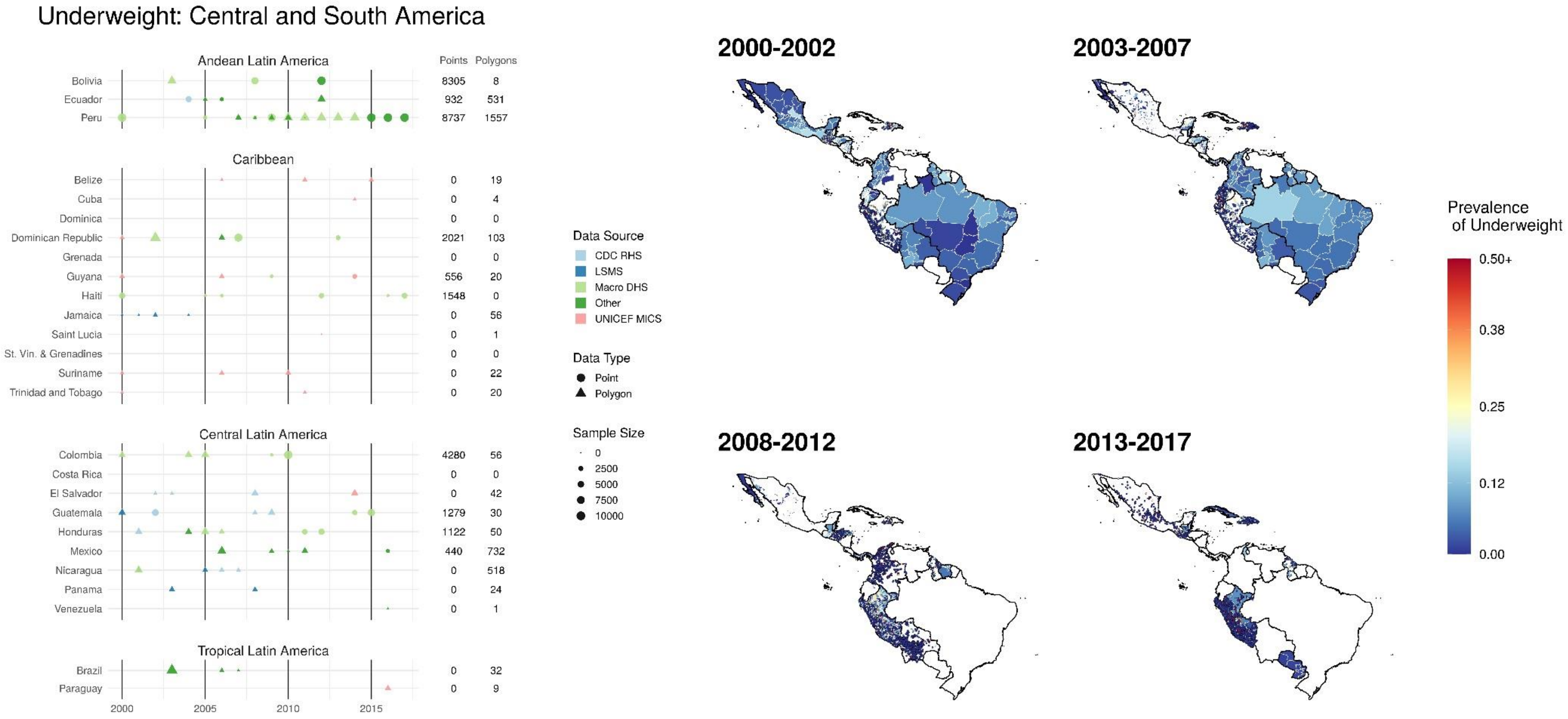

Supplementary Figure 13: Underweight data availability by type and country, 2000–2017 in Central America and the Caribbean and South America.

All data are shown by country and year of survey and mapped at their corresponding geo-positioned coordinate or area. The total number of points and polygons (areal) for each country are plotted by data source, type, and sample size. Sample size represents the number of individual microdata records for each survey. Mean underweight prevalence of the input coordinate or area is mapped.

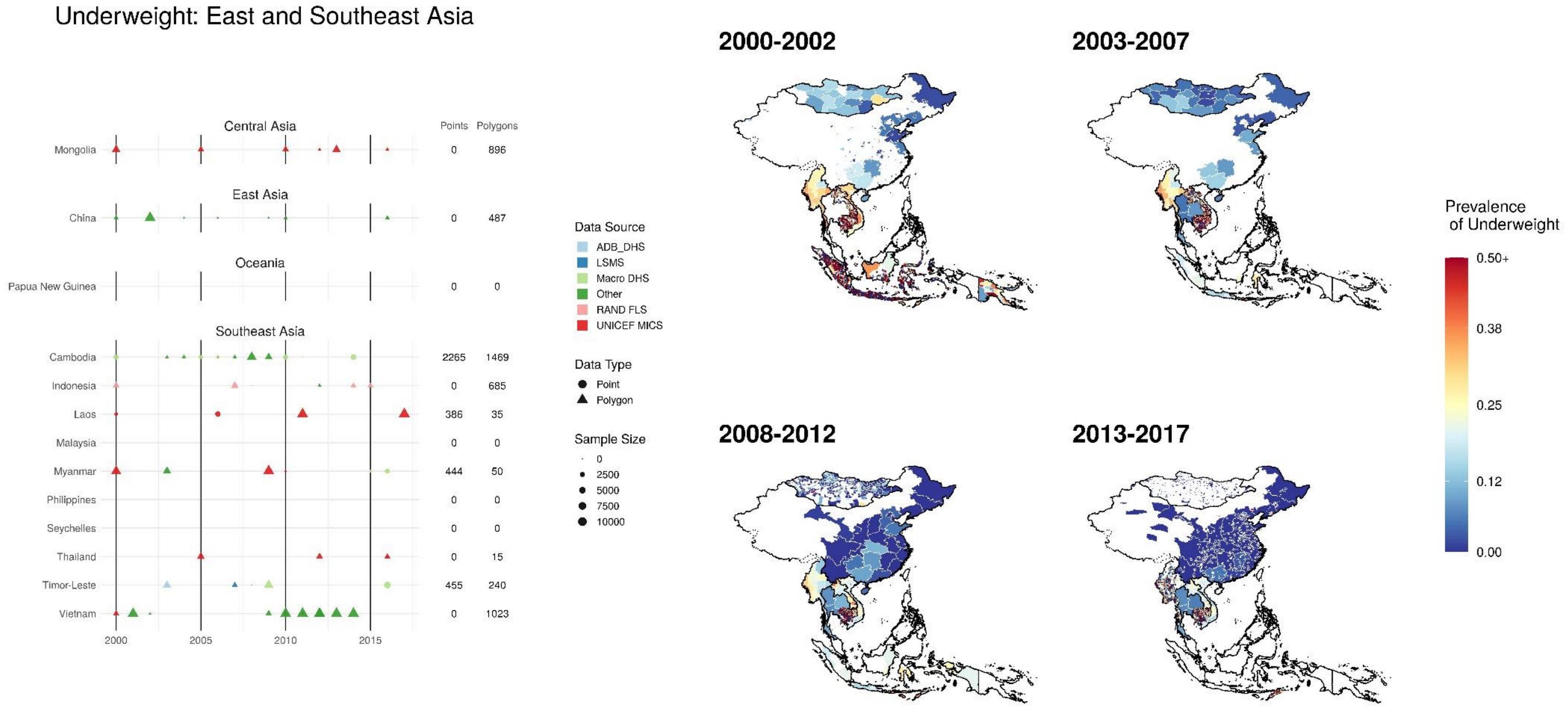

Supplementary Figure 14: Underweight data availability by type and country, 2000–2017 in East and Southeast Asia.
All data are shown by country and year of survey and mapped at their corresponding geo-positioned coordinate or area. The total number of points and polygons (areal) for each country are plotted by data source, type, and sample size. Sample size represents the number of individual microdata records for each survey. Mean underweight prevalence of the input coordinate or area is mapped.

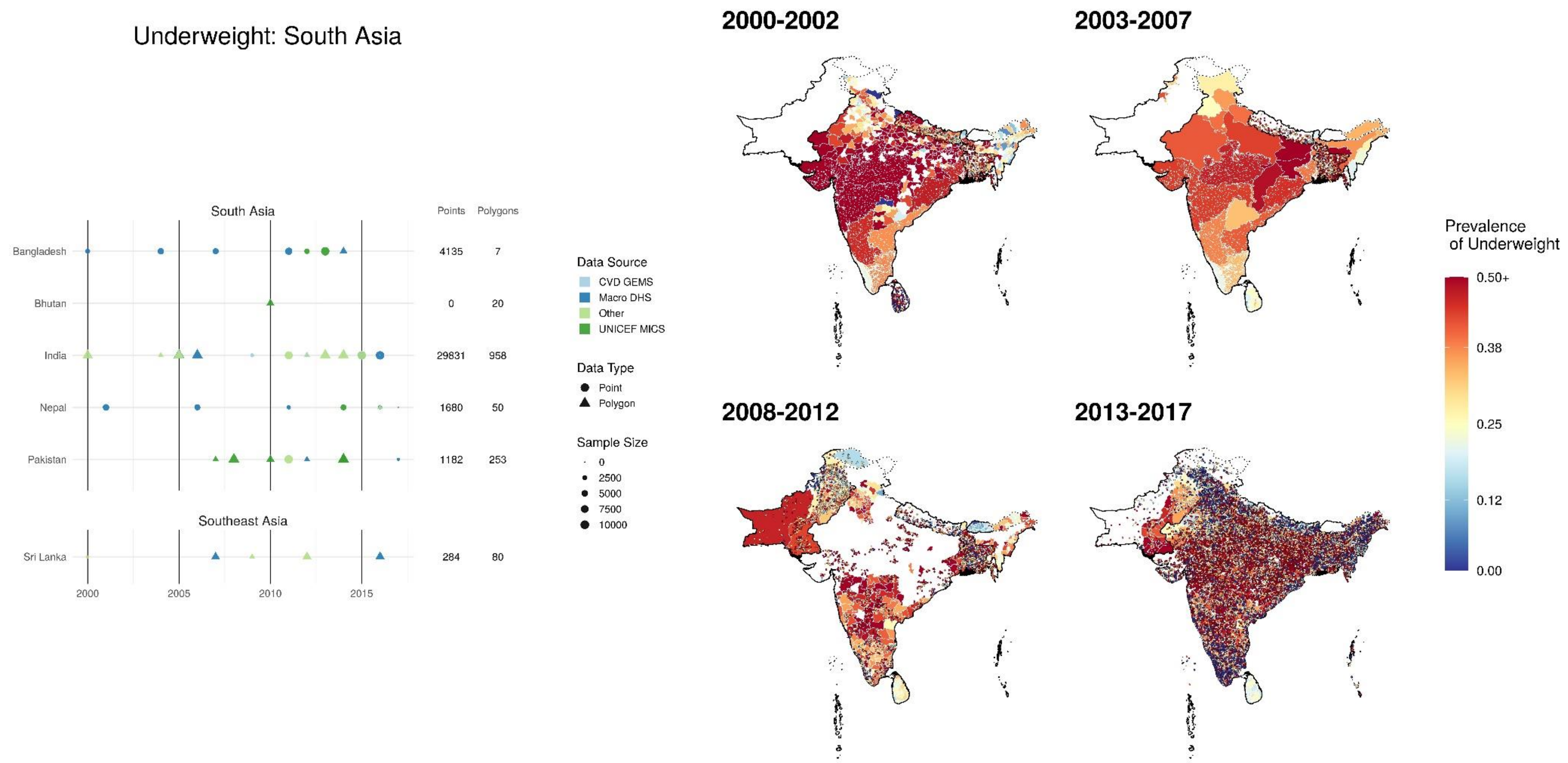

Supplementary Figure 15: Underweight data availability by type and country, 2000–2017 in South Asia.
All data are shown by country and year of survey and mapped at their corresponding geo-positioned coordinate or area. The total number of points and polygons (areal) for each country are plotted by data source, type, and sample size. Sample size represents the number of individual microdata records for each survey. Mean underweight prevalence of the input coordinate or area is mapped.

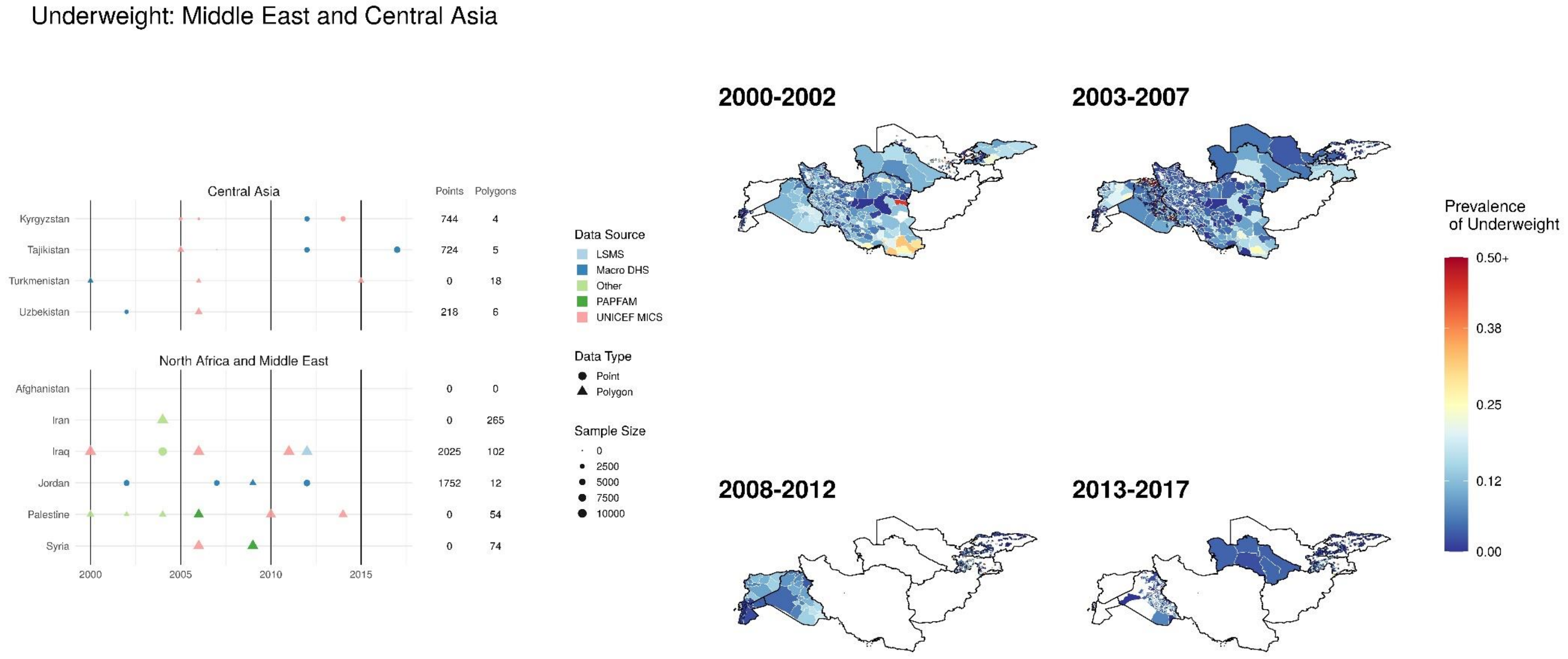

Supplementary Figure 16: Underweight data availability by type and country, 2000–2017 in Middle East and Central Asia.
All data are shown by country and year of survey and mapped at their corresponding geo-positioned coordinate or area. The total number of points and polygons (areal) for each country are plotted by data source, type, and sample size. Sample size represents the number of individual microdata records for each survey. Mean underweight prevalence of the input coordinate or area is mapped.

2.5 Covariates

A variety of environmental and socioeconomic variables were used to predict CGF outcomes. Where available, the finest spatiotemporal resolution of gridded datasets were used. In addition to the covariates detailed below, some country-level variables were included: lag distributed income per capita, and the proportion of the population with access to adequate sanitation, were included in models for stunting, wasting, and underweight.

Supplementary Table 7: Covariates used in mapping.

Covariate	Temporal resolution	Source	Reference
Average daily mean rainfall (Precipitation) **(1)**	Annual	CRUTS	Harris, I., Jones, P. d., Osborn, T. j. & Lister, D. h. Updated high-resolution grids of monthly climatic observations – the CRU TS3.10 dataset. *Int. J. Climatol*. **34**, 623–642 (2014). University of East Anglia. Climatic Research Unit TS v. 3.24 dataset. Available at:https://crudata.uea.ac.uk/cru/data/hrg/cru_ts_3.24.01/. (Accessed: 24th July 2017).
Average daily mean temperature **(2)**	Annual	CRUTS	Harris, I., Jones, P. d., Osborn, T. j. & Lister, D. h. Updated high-resolution grids of monthly climatic observations – the CRU TS3.10 dataset. *Int. J. Climatol*. **34**, 623–642 (2014).<br><br>University of East Anglia. Climatic Research Unit TS v. 3.24 dataset. Available at: https://crudata.uea.ac.uk/cru/data/hrg/cru_ts_3.24.01/. (Accessed: 24th July 2017).
Enhanced Vegetation Index (EVI) **(3)**	Annual	MODIS	Huete, A., Justice, C. & van Leeuwen, W. MODIS vegetation index (MOD 13) algorithm theoretical basis document. (1999).<br><br>USGS & NASA. Vegetation indices 16-Day L3 global 500m MOD13A1 dataset. Available at:https://lpdaac.usgs.gov/dataset_discovery/modis/modis_products_table/mod13a1. (Accessed: 25th July 2017)<br><br>Weiss, D. J. *et al*. An effective approach for gap-filling continental scale remotely sensed time-series. *Isprs J. Photogramm. Remote Sens*. **98**, 106–118 (2014).

Covariate	Temporal resolution	Source	Reference
Fertility **(4)**	Annual	WorldPop (derived)	Lloyd, C. T., Sorichetta, A. & Tatem, A. J. High resolution global gridded data for use in population studies. *Sci. Data* **4**, sdata20171 (2017).<br>World Pop. Get data. Available at: http://www.worldpop.org.uk/data/get_data/ . (Accessed: 25th July 2017)
Growing season length* **(5)**	Static	FAO	FAO. GAEZ – Global Agro-Ecological Zones data portal. Available at: http://www.fao.org/nr/gaez/about-data-portal/en/. (Accessed: 25th July 2017)<br>FAO. GAEZ – Global Agro-Ecological Zones users guide. (2012).
Irrigation* **(6)**	Static	University of Frankfurt	Goethe-Universität. Generation of a digital global map of irrigation areas. Available at: https://www.uni-frankfurt.de/45218039/Global_Irrigation_Map. (Accessed: 25th July 2017)
Malaria incidence **(7)**	Annual	Malaria Atlas Project	Bhatt, S. *et al*. The effect of malaria control on *Plasmodium falciparum* in Africa between 2000 and 2015. *Nature* **526**, 207–211 (2015).
Educational attainment in women of reproductive age (15-49 years old) **(8)**	Annual	Institute for Health Metrics and Evaluation, University of Washington	Graetz, N. *et al*. Mapping persistent local disparity in educational attainment across low- and middle-income countries. *Nature* (2019).
Nutritional yield for vitamin A* **(9)**	Static	Herrero et al (modelled)	Herrero, M. *et al*. Farming and the geography of nutrient production for human use: a transdisciplinary analysis. *Lancet Planet. Health* **1**, e33–e42 (2017).
Population **(10)**	Annual	WorldPop	Lloyd, C. T., Sorichetta, A. & Tatem, A. J. High resolution global gridded data for use in population studies. *Sci. Data* **4**, sdata20171 (2017).<br>World Pop. Get data. Available at: http://www.worldpop.org.uk/data/get_data/ . (Accessed: 25th July 2017)

Covariate	Temporal resolution	Source	Reference
Travel time to nearest settlement >50,000 inhabitants*<br>**(11)**	Static	Big Data Institute, Nuffield Department of Medicine, University of Oxford	Weiss, D. J. *et al*. A global map of travel time to cities to assess inequalities in accessibility in 2015. *Nature* **533**, 333–336 (2018).
Urbanicity<br>**(12)**	Annual	European Commission/GHS	Pesaresi, M. *et al*. Operating procedure for the production of the Global Human Settlement Layer from Landsat data of the epochs 1975, 1990, 2000, and 2014. (Publications Office of the European Union, 2016).
*Temporally dynamic covariates which were reformatted as a synoptic mean over each estimation period or as a mid-period year estimate.			

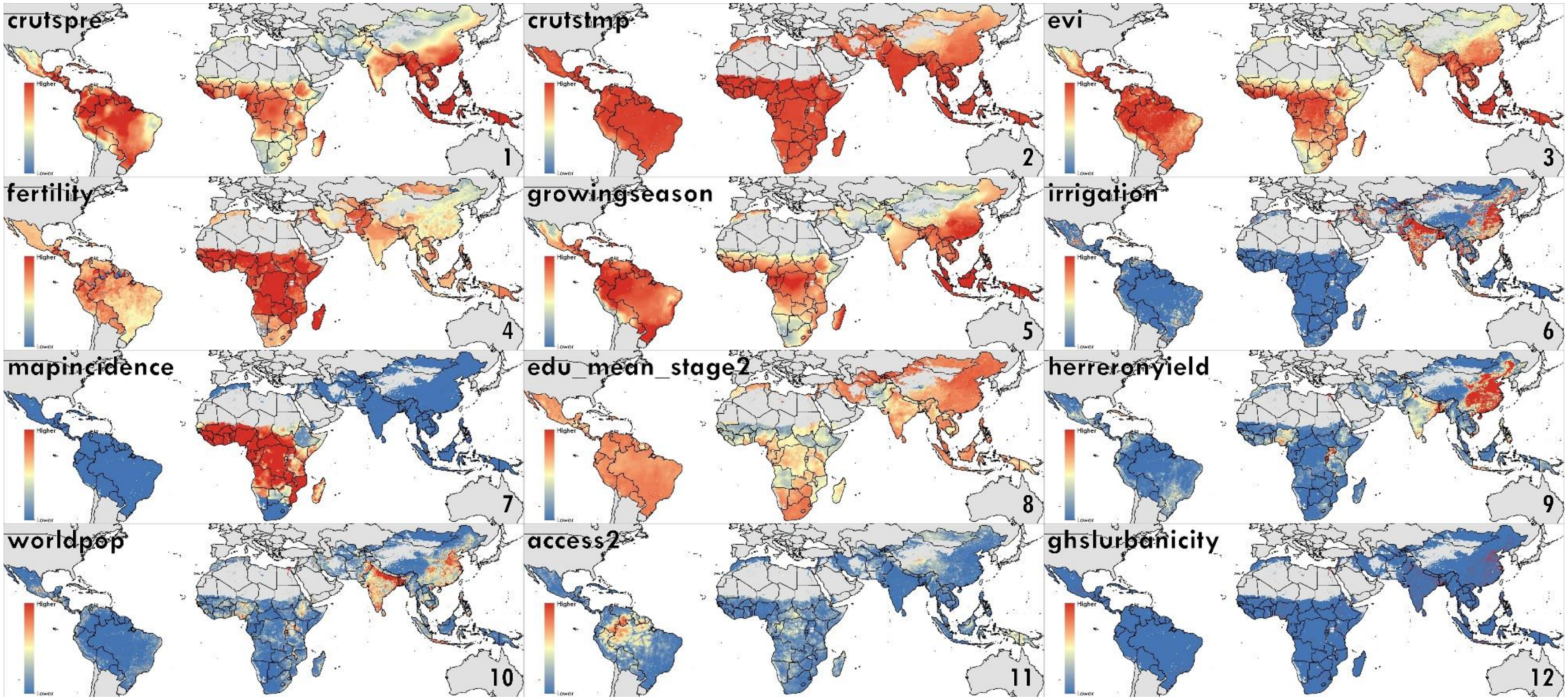

Supplementary Figure 17: Covariates.

Twelve covariate raster layers of possible environmental and socioeconomic correlates of CGF in LMICs were used as inputs for the stacking modelling process. Time-varying covariates are presented for the year 2017. For the year of production of non-time-varying covariates and additional details, please refer to the individual covariate citation in Supplementary Table 7.

3.0 Supplementary Methods

3.1 Seasonality Adjustment

Weight-for-height z-scores (WHZ) were used to calculate an individual child wasting status. As a data preprocessing step, we performed a seasonality adjustment on individual-level child weights in order to account for differences in observed child weight that may have been due to food scarcity around the month in which the survey was conducted. To adjust weight measurements, we fit a model for each region (Extended Data Fig. 10) with a 12-month seasonal spline, a country-level fixed effect, and a smooth spline over the duration of our data collection using the *mgcv* package in R and the following formula:

$$WHZ \sim s_{cc}(month) + s_{tp}(t) + as.factor(country).$$

WHZ is a child's weight-for-height z-score, $month$ is the integer-valued month of the year (1, …, 12), t is the time of the interview in integer months since the earliest observation of any child in the dataset, and $country$ is a factor variable representing the country where the observation was recorded. We modelled the periodic component on months using 12 cyclic cubic regression splines basis functions (cc) and we accounted for a smooth longer time temporal trend using four thin-plate splines (tp). The country effects and the long-term temporal spline were included only to help avoid confounding during fitting of the seasonal spline fit and neither country effects nor the long-term trend were used in the seasonal adjustment. We then adjusted all observations to account for the difference in the seasonal period between the month of the interview and an average day of the year as determined by which days align with the mean of the periodic spline.

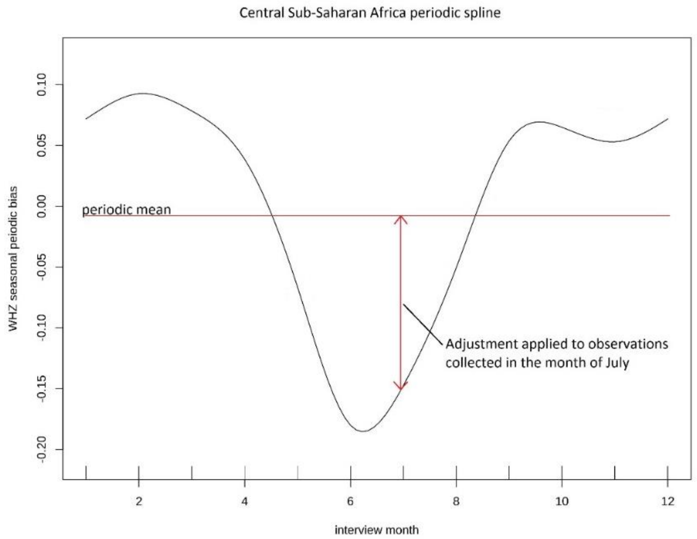

Supplementary Figure 18: Periodic seasonality adjustment.

The fitted seasonal periodic spline for wasting for Central sub-Saharan Africa region with the marked mean of the periodic function and an example of the seasonality adjustment that would be applied to weight-for-height z-scores collected in this region in the month of July.

3.2 Geostatistical Model

3.2.1 Model geographies

A total of three sub-models were run for each CGF indicator based on continuous geographic regions within LMICs chosen to align with the 14 regions as shown the Extended Data Figure 10. These regions were determined based on both proximity and epidemiological similarity. All data within the spatial region, and within a one-degree buffer from the boundaries of each region, were included in each regional model to minimize edge effects.

3.2.2 Ensemble covariate modelling

An ensemble covariate modelling method was implemented in order to select covariates and capture possible non-linear effects and complex interactions between them[31]. For each region, three sub-models were fit to our dataset, using all of our covariate data as explanatory predictors: generalised additive models (GAM), boosted regression trees (BRT), and lasso regression. Country-level fixed effects were also included in the BRT model as dummy-coded covariates. Sample weights were used in sub-models, where applicable, such that cluster locations with latitude and longitude had a sample weight of 1, while cluster locations where the latitude and longitude were generated by the polygon resampling process had a weight based on the K-means clustering process.

Each sub-model's predictive performance was tested using five-fold cross-validation to avoid overfitting. We removed 20% of the data to create five out-of-sample predications and complied these into a single comprehensive set of predictions. Additionally, the same sub-models were also run using 100% of the data, and a full set of in-sample predictions were created. The five sets of out-of-sample sub-model predictions were fed into the full geostatistical model as the explanatory covariates when performing the model fit. The in-sample predictions from the sub-models were used as the covariates when generating predictions using the fitted full geostatistical model. A recent study has shown that this ensemble approach can improve predictive validity by up to 25% over an individual model[31].

Predictions from each sub-model are generated based on patterns and relationships between the raw covariates and prevalence survey data, while predictions from the full geostatistical model are generated based on patterns and relationships between the predictions from the ensemble of sub-models and prevalence survey data. To discover the relationships between the sub-model prediction layers (used as covariates in the full geostatistical model) and the prevalence data, the only values of the covariates (sub-model prediction layers) "seen" by the model are the values underlying the locations of surveys. As such, it is possible that estimates will be generated in areas where the values of the covariates exceed the minimum and maximum values observed by the model. In these areas, the estimates are generated by extrapolating from the patterns observed within the range of covariates underlying the survey data. More information on the list of the covariates and plots of all covariates, can be found in Supplementary Table 7 and Supplementary Fig. 17.

The primary goal of using the stacking procedure in our analyses was to maximise the predictive power of the raster covariates by capturing the non-linear effects and complex interactions between covariates to optimise the model performance. Bhatt *et al*. (2017)[31] contend that the primary purpose of

the sub-model predictions is to improve the mean function of the Gaussian process. While we have determined a way to include the uncertainty from two of our sub-models (GAM and Lasso regression), we have not determined a way to include uncertainty from the BRT sub-model into our final estimates. Whereas GAM and Lasso regression seek to fit a single model that best describes the relationship between response variable and some set of predictors, BRT method fits a large number of relatively simple models whose predictions are then combined to give robust estimates of the response. While this feature of BRT model makes it a powerful tool for analysing complex data, quantifying the relative uncertainty contributed by each simple model as well as uncertainty from the complex interactions of the predictor variables is challenging[32,33].

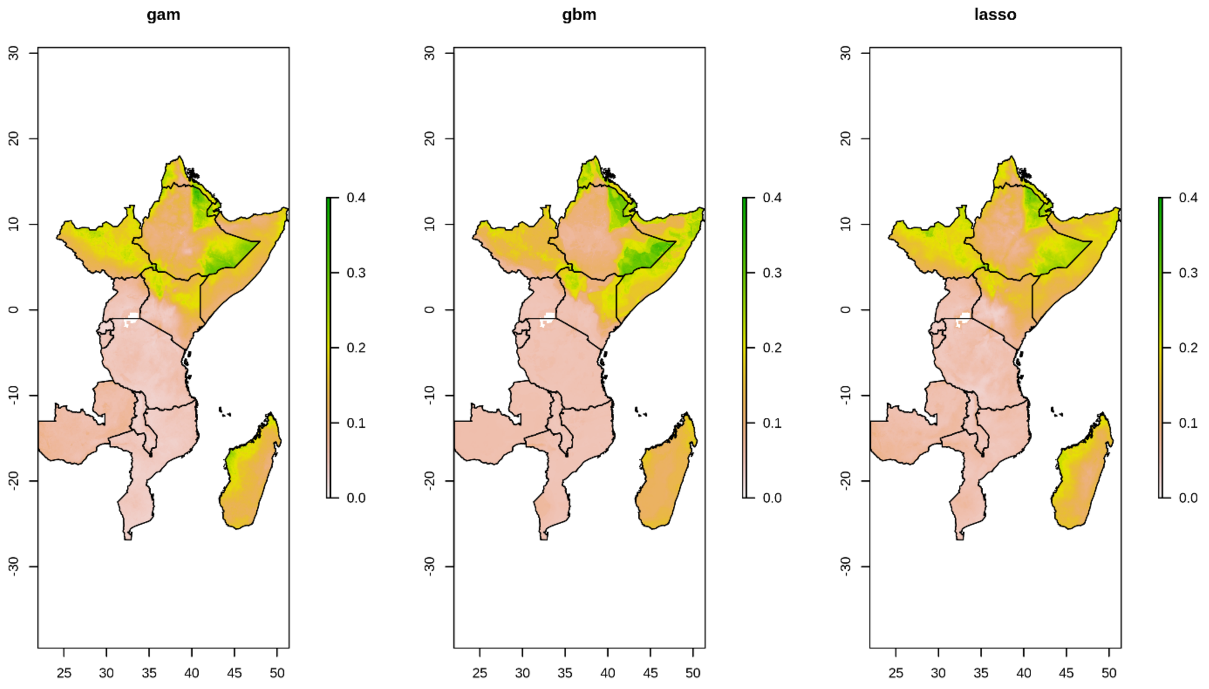

Supplementary Figure 19: Ensemble predicted rasters.

Predicted 2017 rasters, for use as covariates in the INLA (integrated nested Laplace approximation) modelling, shown for the Eastern sub-Saharan Africa region. The *gam* plot shows the predictions from a generalised additive model fit, the *gbm* plot shows the predictions from a boosted regression tree fit, the *lasso* plot shows the predictions from a lasso penalised regression model fit.

3.2.3 Model description

Binomial count data are modelled within a Bayesian hierarchical modelling framework using a logit link function and a spatially and temporally explicit hierarchical generalised linear regression model to fit prevalence of each of CGF indicators in 14 modelling regions[34]: Andean South America, Central America and the Caribbean, Central sub-Saharan Africa (SSA), East Asia, Eastern SSA, Middle East, North Africa, Oceania, Southeast Asia, South Asia, Southern SSA, Central Asia, Tropical South America, and Western-SSA (as seen in Extended Data Fig 10). For each region, we explicitly write the hierarchy that defines our Bayesian method:

For each binomial CGF indicator, we modelled mean children with stunting, wasting, or who were underweight in each survey cluster, d. Survey clusters are precisely located by their GPS coordinates and year of observation, which we map to a spatial raster location, i, at time, t. We observed the number of children reported to be stunted, wasted, or underweight, respectively, as binomial count data, C_d , among an observed sample size, N_d. As we may have observed several data clusters within a given location, i, at time, t, we refer to the probability of stunting, wasting, or underweight, p, within a given cluster, d, by its indexed location, i, and time, t, as $p_{i(d),t(d)}$.

$$C_d | p_{i(d),t(d)}, N_d \sim \text{Binomial}\left(p_{i(d),t(d)}, N_d\right) \ \forall \text{ observed clusters } d$$

$$\text{logit}\left(p_{i,t}\right) = \beta_0 + \mathbf{X}_{i,t}\boldsymbol{\beta} + Z_{i,t} + \epsilon_{\text{ctr}(i)} + \epsilon_{i,t} + Z_{i,t} \ \forall\, i \in \text{spatial domain} \ \forall\, t \in \text{time domain}$$

$$\sum_{h=1}^{3} \beta_h = 1$$

$$\epsilon_{\text{ctr}} \sim \text{iid Normal}(0, \gamma^2)$$

$$\epsilon_{i,t} \sim \text{iid Normal}(0, \sigma^2)$$

$$\mathbf{Z} \sim \text{GP}(0, \Sigma^{\text{space}} \otimes \Sigma^{\text{time}})$$

$$\Sigma^{\text{space}} = \frac{\omega^2}{\Gamma(\nu)2^{\nu-1}} \times (\kappa D)^{\nu} \times \text{K}_{\nu}(\kappa D)$$

$$\Sigma^{\text{time}}_{j,k} = \rho^{|\,k-j|}$$

For indices d, i, and t, *(index) is the value of * at that index. The probabilities, $p_{i,t}$ represent both the annual prevalence at the space-time location and the probability that an individual child was afflicted with the risk factor given that they lived at that particular location. The annual prevalence, $p_{i,t}$, of each indicator was modelled as a linear combination of the three sub-models (generalised additive model (GAM), boosted regression trees (BRT), and lasso regression), rasterised covariate values $\mathbf{X}_{i,t}$, a correlated spatiotemporal error term, $Z_{i,t}$, country random effects, $\epsilon_{ctr(i)}$ with one unstructured country random effect fit for each country in the modelling region and all ϵ_{ctr} sharing a common variance parameter, γ^2, and an independent nugget effect, $\epsilon_{i,t}$ with variance parameter, σ^2. Coefficients in β_h in the three sub-models h=1,2,3 represent their respective predictive weighting in the mean logit link, while the joint error term, $Z_{i,t}$, accounts for residual spatiotemporal autocorrelation between individual data points that remains after accounting for the predictive effect of the sub-model covariates, the country-level random effect, $\epsilon_{\text{ctr}(i)}$, and the nugget independent error term, $\epsilon_{i,t}$. The residuals, $Z_{i,t}$, are modelled as a three-dimensional Gaussian process (GP) in space-time centered at

zero and with a covariance matrix constructed from a Kronecker product of spatial and temporal covariance kernels. The spatial covariance, Σ^{space}, is modelled using an isotropic and stationary Matérn function[35], and temporal covariance, Σ^{time} fun, as an annual autoregressive (AR1) function over the 18 years represented in the model. In the stationary Matérn function, Γ is the Gamma function, K_v is the modified Bessel function of order $v > 0, \kappa > 0$ is a scaling parameter, D denotes the Euclidean distance, and ω^2 is the marginal variance. The scaling parameter, κ, is defined to be $\kappa = \sqrt{8v}/\delta$ where δ is a range parameter (which is about the distance where the covariance function approaches 0.1) and v is a scaling constant, which is set to 2 rather than fit from the data[36,37]. This is parameter is difficult to reliably fit, as documented by many other analyses[36,38,39] that set this to 2. The number of rows and the number of columns of the spatial Matérn covariance matrix are both equal to the number of spatial mesh points for a given modelling region. In the AR1 function, ρ is the autocorrelation function (ACF), and k and j are points in the time series where $|k - j|$ defines the lag. The number of rows and the number of columns of the AR1 covariance matrix are both equal to the number of temporal mesh points (18). The number of rows and the number of columns of the space-time covariance matrix, $\Sigma^{\text{space}} \otimes \Sigma^{\text{time}}$, for a given modelling region are both equal to: (the number of spatial mesh points times the number of temporal mesh points).

This approach leveraged the data's residual correlation structure to more accurately predict prevalence estimates for locations with no data, while also propagating the dependence in the data through to uncertainty estimates[40]. The posterior distributions were fit using computationally efficient and accurate approximations in R-INLA[41,42] (integrated nested Laplace approximation) with the stochastic partial differential equations (SPDE)[37] approximation to the Gaussian process residuals using R project v.3.5.1. The SPDE approach using INLA has been demonstrated elsewhere, including the estimation of health indicators, particulate air matter, and population age structure[27,43–46]. Uncertainty intervals (UIs) were generated from 1,000 draws (i.e., statistically plausible candidate maps)[47] created from the posterior-estimated distributions of modelled parameters.

3.2.4 Priors

The following priors were used for all three of our CGF models:

$$\beta_0 \sim N(\mu = 0, \sigma^2 = 3^2),$$

$$\boldsymbol{\beta} \sim \text{iid } N\left(\mu = \frac{1}{\text{\# ensemble models}}, \sigma^2 = 3^2\right),$$

$$log\left(\frac{1+\rho}{1-\rho}\right) \sim N(\mu = 0, \sigma^2 = 1/0.15),$$

$$log\left(\frac{1}{\sigma_{nug}^2}\right) \sim loggamma(\alpha = 1, \gamma = 2).$$

$$\theta_1 = log(\tau) \sim N(\mu_{\theta_1}, \sigma_{\theta_1}^2)$$

$$\theta_2 = log(\kappa) \sim N(\mu_2, \sigma_{\theta_2}^2).$$

Given that our covariates used in INLA (i.e. the predicted outputs from the ensemble models) should be on the same scale as our predictive target, we believe that the intercept in our model should be close to zero and that the regression coefficients should sum to 1. As such, we have chosen the prior for our intercept to be $\mathrm{N}(0, \sigma^2 = 3^2)$, and the prior for the fixed effect coefficients to be $\mathrm{N}(\frac{1}{\#\text{ ensemble models}}, \sigma^2 = 3^2)$. The prior on the temporal correlation parameter, ρ, is chosen to be mean zero, showing no prior preference for either positive or negative auto-correlation structure, and with a distribution that is wide enough such that within three standard deviations of the mean, the prior includes values of ρ ranging from -0.95 to 0.95. The priors on the random effect variances were chosen to be relatively loose given that we believe our fixed effects covariates should be well-correlated with our outcome of interest, which might suggest relatively small random effects values. At the same time, we wanted to avoid using a prior that was so diffuse as to actually put high prior weight on large random effect variances. For stability, we used the uncorrelated multivariate normal priors that INLA automatically determines (based on the finite elements mesh) for the log-transformed spatial hyperparameters, κ and τ. In our parameterisation, we represent α and γ in the *loggamma* distribution as scale and shape, respectively.

Supplementary Table 8: Spatial hyperparameter priors by region.

Region	μ_{θ_1}	$\sigma^2_{\theta_1}$	μ_2	$\sigma^2_{\theta_2}$
Andean South America	0.011191	10	-1.2767	10
Central America and the Caribbean	0.209786	10	-1.4753	10
Central sub-Saharan Africa	-0.20487	10	-1.06064	10
East Asia	0.377015	10	-1.64253	10
Eastern sub-Saharan Africa	0.137024	10	-1.40254	10
Middle East	-0.20057	10	-1.06494	10
North Africa	0.296578	10	-1.56209	10
Southeast Asia and Oceania	0.423677	10	-1.68919	10
South and Central Asia	0.122237	10	-1.38775	10
Southern sub-Saharan Africa	-0.41501	10	-0.8505	10
Tropical South America	0.130726	10	-1.39624	10
Western sub-Saharan Africa	0.201186	10	-1.4667	10

3.2.5 Mesh construction

We constructed the finite elements mesh for the stochastic partial differential equation approximation to the Gaussian process regression using a simplified polygon boundary (in which coastlines and complex boundaries were smoothed) for each of the regions within our model. We set the inner mesh triangle maximum edge length (the mesh size for areas over land) to be 0.75 degrees, and the buffer maximum edge length (the mesh size for areas over the ocean) to be 5.0 degrees. An example finite elements mesh constructed for Eastern sub-Saharan mesh can be found in Supplementary Fig. 20.

3.2.6 Model fitting and estimate generation

Models were fit in INLA with methods consistent with those used in geospatial modelling of CGF, under-5 mortality, and educational attainment in Africa, published previously[27,43,48].

Where possible, the point data (GPS-positioned data) were used in the analyses. In instances where this was not possible, the data were matched to the smallest possible areal unit. The areal data were then resampled to generate pseudo-point data based on the underlying population distribution within the polygon. The methods for the resampling are consistent with those previously used in geospatial modelling of under-5 mortality[43]. Resampling K-means weights were used within the INLA fit by multiplying the corresponding log-likelihood evaluation for the specific observation by the observation's K-means weight. These weights were used to ensure that we did not artificially inflate the amount of information in the dataset by effectively using them to inflate the dispersion in the log-likelihood for resampled-polygon points. While the model this induces is not necessarily generative, it does yield a well-defined target distribution. This is analogous to how weighting is often done in generalised additive models[49]. Data points that could be geo-referenced to latitude-longitude locations were assigned a weight of 1, ensuring that when the log-likelihood contribution from an observation was evaluated it contributed only to the log-likelihood at that observation's space-time location. For cluster locations generated based on the polygon resampling process, the log-likelihood of those points contributed proportionate to the K-means weights, effectively diffusing the evaluation of the observation across the polygon.

As part of the ensemble modelling process, prediction surfaces from the out-of-sample ensemble sub-models were used as covariates in the spatiotemporal model. Estimates of the fixed effects' beta coefficients were derived from the contribution of each of the sub-models to INLA's predicted prevalence estimates, in conjunction with parameter estimates of the contribution of location and time. To create final estimates, the in-sample prediction surfaces of prevalence from the sub-models (serving as covariates) were used as covariates in conjunction with the fitted random effects from INLA to predict and calculate estimates of prevalence for each grid cell in each year.

Our implementation of INLA using the R-INLA software relies on a Gaussian approximation of the full conditional distribution of latent variables, and uses the empirical Bayes approximation for the hyperparemeters[41]. We have tried the full hyperparameter grid integration and central composite design (CCD) integration in various settings and have found our models to be nearly indistinguishable. Due to its computing resource efficiency, we used the empirical Bayes procedure. In a very similar setting with malaria household survey data, other authors (including the senior author here) compared the INLA results directly with those from Hamiltonian Markov Chain Monte Carlo and found nearly identical results between the two fits[50].

All estimates were generated by taking 1,000 draws from the posterior distribution, which yielded 1,000 candidate maps used to summarise the grid cell- and aggregated-level statistics. For estimates at the grid cell level, these draws were used directly to generate estimates and uncertainty. Aggregated estimates, in which grid cell-level estimates were summarised to administrative boundaries, were generated by creating population-weighted averages for each administrative boundary, for each draw. 95% uncertainty intervals around the mean of our estimates were generated.

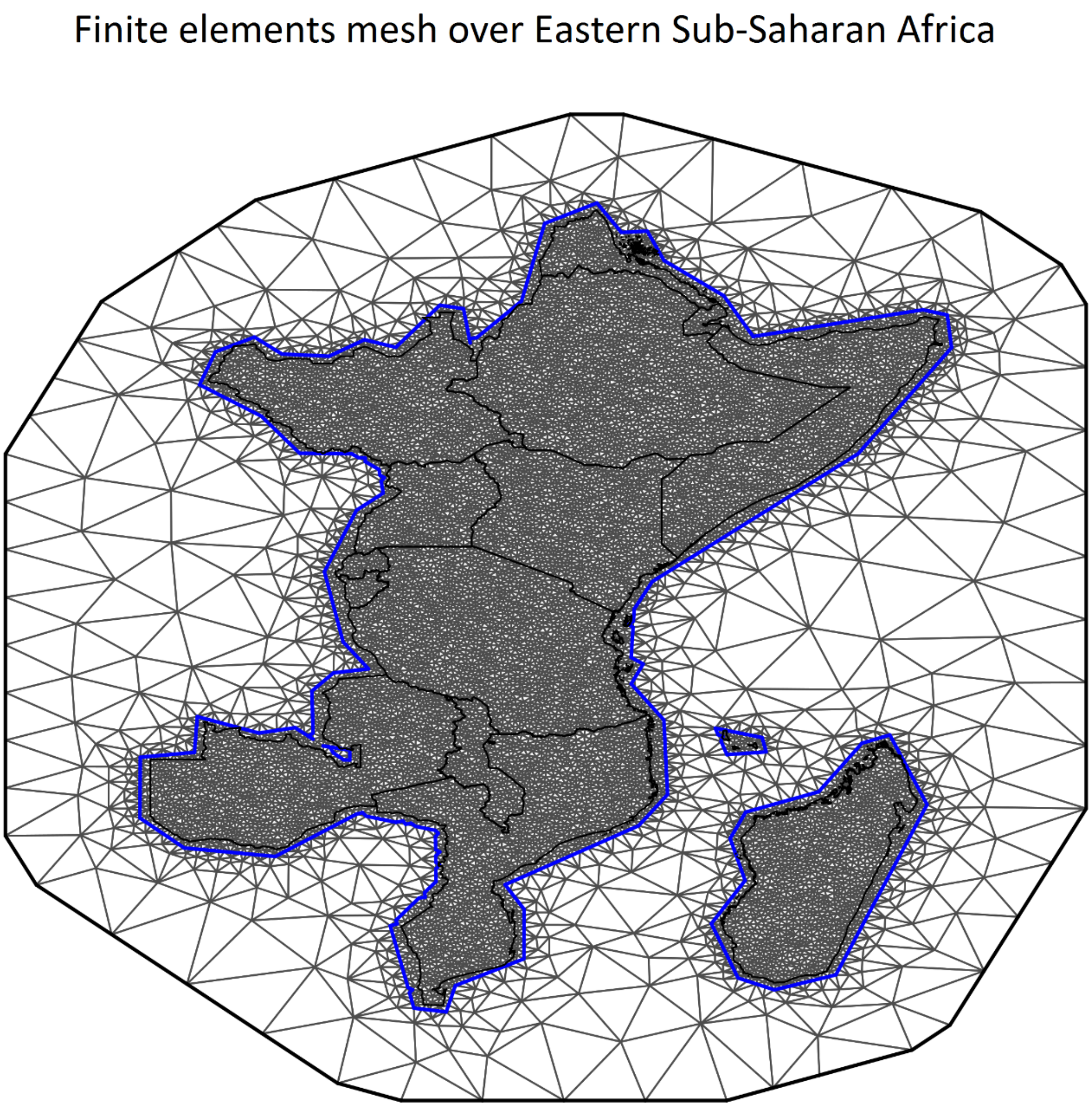

Supplementary Figure 20: Finite elements mesh.

The finite elements mesh used to fit the space-time correlated error for the Eastern sub-Saharan Africa (ESSA) region overlaid on the countries in ESSA. Both the fine-scale mesh over land in the modelling region and the coarser buffer region mesh are shown. The simplified region polygon used to determine the boundary for the modelling region is shown in blue.

3.3 Model Results

Supplementary Table 9: Stunting fitted parameters.

Lower, median, and upper quantiles (percentiles 0.025, 0.50, 0.975) are displayed for the main parameters from the stunting models by region. The fixed effects covariates corresponding to the predicted ensemble rasters are shown in the first five columns, while fitted values for the spatiotemporal field hyperparameters and the precisions (inverse variance) for our random effects are shown in the last five columns.

Regions	Percentiles	int	gam	gbm	lasso	Nominal Range	Nominal Variance	AR1 rho	Precision for IID.ID	Precision for CTRY.ID
North Africa	**0.025**	-0.39	0.17	0.30	0.16	2.49	0.11	0.35	0.04	0.39
	**0.50**	-0.15	0.28	0.42	0.31	3.21	0.14	0.61	0.03	0.18
	**0.975**	0.09	0.38	0.53	0.46	4.06	0.18	0.75	0.03	0.09
Central sub-Saharan Africa	**0.025**	-0.23	-0.07	0.42	0.21	2.76	0.05	0.77	0.02	0.28
	**0.50**	-0.04	0.07	0.55	0.38	3.48	0.07	0.84	0.02	0.14
	**0.975**	0.15	0.21	0.69	0.54	4.44	0.08	0.89	0.02	0.08
Middle East	**0.025**	-0.13	0.13	0.53	0.06	2.67	0.22	0.69	0.04	0.56
	**0.50**	0.20	0.21	0.63	0.16	3.34	0.28	0.79	0.03	0.24
	**0.975**	0.53	0.29	0.73	0.25	4.19	0.38	0.86	0.03	0.11
Central America and the Caribbean	**0.025**	-0.83	-0.04	0.37	0.35	4.18	0.22	0.87	0.19	0.77
	**0.50**	-0.40	0.07	0.47	0.46	6.06	0.33	0.92	0.15	0.30
	**0.975**	0.03	0.18	0.58	0.57	8.07	0.53	0.96	0.09	0.15
Western sub-Saharan Africa	**0.025**	-0.17	0.01	0.57	0.16	2.38	0.07	0.71	0.04	0.19
	**0.50**	-0.03	0.09	0.65	0.26	2.72	0.09	0.77	0.04	0.11
	**0.975**	0.11	0.18	0.72	0.36	3.17	0.10	0.82	0.03	0.06
Andean South America	**0.025**	-0.41	0.04	0.36	0.28	3.32	0.11	0.87	0.07	0.94
	**0.50**	0.03	0.14	0.47	0.40	4.40	0.15	0.92	0.06	0.36
	**0.975**	0.46	0.23	0.57	0.51	5.97	0.22	0.95	0.05	0.15
Southern sub-Saharan Africa	**0.025**	-0.26	-0.10	0.19	0.25	2.92	0.03	0.77	0.03	0.47
	**0.50**	0.04	0.10	0.40	0.50	4.40	0.04	0.89	0.02	0.20
	**0.975**	0.34	0.30	0.61	0.74	6.71	0.07	0.95	0.02	0.10
	**0.025**	-0.59	-0.13	0.67	-0.13	7.86	0.04	0.61	0.03	0.68

Eastern sub-Saharan Africa	**0.50**	-0.17	0.06	0.86	0.08	13.24	0.09	0.83	0.03	0.27
	**0.975**	0.25	0.26	1.05	0.28	21.90	0.17	0.93	0.02	0.11
Tropical South America	**0.025**	-0.23	0.07	0.47	0.20	2.47	0.10	0.88	0.07	0.20
	**0.50**	-0.08	0.15	0.55	0.30	2.96	0.11	0.90	0.07	0.11
	**0.975**	0.06	0.23	0.62	0.40	3.41	0.13	0.92	0.06	0.07
East Asia	**0.025**	-0.36	0.02	0.54	0.09	3.21	0.16	0.78	0.05	0.44
	**0.50**	-0.06	0.13	0.64	0.23	4.15	0.22	0.89	0.04	0.20
	**0.975**	0.23	0.24	0.75	0.36	5.55	0.30	0.94	0.03	0.10
Southeast Asia and Oceania	**0.025**	-0.46	0.06	0.69	0.05	4.01	0.13	0.87	0.10	0.47
	**0.50**	-0.18	0.13	0.75	0.12	4.75	0.17	0.90	0.09	0.22
	**0.975**	0.09	0.19	0.81	0.20	5.66	0.22	0.93	0.09	0.11
South and Central Asia	**0.025**	-0.57	-0.06	0.19	0.54	4.80	0.09	0.91	0.05	0.57
	**0.50**	-0.24	0.05	0.29	0.66	6.29	0.13	0.95	0.04	0.25
	**0.975**	0.09	0.15	0.38	0.79	8.44	0.19	0.97	0.03	0.12

Supplementary Table 10: Wasting fitted parameters.

Lower, median, and upper quantiles (percentiles 0.025, 0.50, 0.975) are displayed for the main parameters from the wasting models by region. The fixed effects covariates corresponding to the predicted ensemble rasters are shown in the first five columns, while fitted values for the spatiotemporal field hyperparameters and the precisions (inverse variance) for our random effects are shown in the last five columns.

Regions	Percentiles	int	gam	gbm	lasso	Nominal Range	Nominal Variance	AR1 rho	Precision for IID.ID	Precision for CTRY.ID
North Africa	**0.025**	-0.41	0.29	0.50	-0.13	2.25	0.16	0.05	0.03	0.43
	**0.50**	-0.16	0.40	0.60	0.00	2.93	0.20	0.43	0.03	0.20
	**0.975**	0.10	0.51	0.71	0.13	3.81	0.25	0.65	0.02	0.10
Central sub-Saharan Africa	**0.025**	-0.31	-0.17	0.37	0.29	3.66	0.06	0.53	0.03	0.34
	**0.50**	-0.09	-0.01	0.54	0.47	4.92	0.09	0.75	0.03	0.17
	**0.975**	0.13	0.15	0.71	0.66	6.72	0.12	0.86	0.02	0.09
Middle East	**0.025**	-0.36	0.12	0.33	0.09	2.66	0.13	0.38	0.04	0.72
	**0.50**	0.00	0.27	0.47	0.26	4.01	0.21	0.68	0.03	0.31

	**0.975**	0.37	0.41	0.62	0.43	6.15	0.33	0.83	0.02	0.15
**Central America and the Caribbean**	**0.025**	-0.47	0.10	0.05	0.16	2.09	0.09	0.61	0.12	0.47
	**0.50**	-0.15	0.32	0.30	0.38	3.56	0.14	0.81	0.09	0.20
	**0.975**	0.17	0.55	0.56	0.59	6.13	0.24	0.91	0.06	0.09
**Western sub-Saharan Africa**	**0.025**	-0.28	-0.17	0.47	0.35	2.19	0.09	0.32	0.06	0.23
	**0.50**	-0.12	-0.06	0.58	0.48	2.57	0.11	0.56	0.05	0.13
	**0.975**	0.03	0.04	0.70	0.61	2.98	0.13	0.70	0.05	0.07
**Andean South America**	**0.025**	-0.25	-0.08	0.09	0.40	5.83	0.10	0.72	0.07	0.75
	**0.50**	0.21	0.09	0.30	0.60	10.01	0.19	0.89	0.05	0.28
	**0.975**	0.68	0.27	0.51	0.81	17.79	0.36	0.96	0.04	0.11
**Southern sub-Saharan Africa**	**0.025**	-0.34	-0.20	0.43	0.15	2.61	0.05	0.50	0.05	0.46
	**0.50**	-0.04	0.02	0.61	0.37	4.25	0.09	0.75	0.04	0.19
	**0.975**	0.26	0.24	0.80	0.59	6.95	0.14	0.89	0.03	0.09
**Eastern sub-Saharan Africa**	**0.025**	-1.18	-0.11	0.42	-0.15	26.16	0.03	0.41	0.06	0.93
	**0.50**	-0.47	0.17	0.66	0.16	49.64	0.10	0.82	0.04	0.35
	**0.975**	0.24	0.46	0.90	0.47	92.90	0.34	0.95	0.03	0.13
**Tropical South America**	**0.025**	-0.19	-0.03	0.28	0.42	2.20	0.11	0.77	0.05	0.30
	**0.50**	-0.01	0.08	0.38	0.54	2.64	0.13	0.83	0.04	0.17
	**0.975**	0.17	0.20	0.47	0.66	3.20	0.15	0.88	0.04	0.10
**East Asia**	**0.025**	-0.45	-0.21	0.58	0.18	1.83	0.11	0.59	0.09	0.48
	**0.50**	-0.15	-0.07	0.72	0.35	3.46	0.18	0.80	0.07	0.21
	**0.975**	0.16	0.08	0.85	0.52	6.07	0.27	0.90	0.05	0.10
**Southeast Asia and Oceania**	**0.025**	-0.64	-0.13	0.61	0.23	3.11	0.11	0.83	0.08	0.43
	**0.50**	-0.39	-0.04	0.70	0.34	3.80	0.14	0.88	0.07	0.20
	**0.975**	-0.14	0.05	0.79	0.44	4.58	0.17	0.91	0.07	0.10
**South and Central Asia**	**0.025**	-0.41	-0.08	0.44	0.21	3.03	0.07	0.55	0.06	0.42
	**0.50**	-0.13	0.05	0.59	0.37	4.02	0.10	0.76	0.05	0.19
	**0.975**	0.14	0.17	0.73	0.52	5.33	0.13	0.86	0.04	0.09

Supplementary Table 11: Underweight fitted parameters.

Lower, median, and upper quantiles (percentiles 0.025, 0.50, 0.975) are displayed for the main parameters from the underweight models by region. The fixed effects covariates corresponding to the predicted ensemble rasters are shown in the first five columns, while fitted values for the spatiotemporal field hyperparameters and the precisions (inverse variance) for our random effects are shown in the last five columns.

Regions	Percentiles	int	gam	gbm	lasso	Nominal Range	Nominal Variance	AR1 rho	Variance for IID.ID	Variance for CTRY.ID
North Africa	**0.025**	-0.48	0.42	0.13	0.17	3.68	0.13	0.27	0.02	0.58
	**0.50**	-0.19	0.52	0.20	0.28	5.02	0.18	0.64	0.02	0.26
	**0.975**	0.11	0.61	0.27	0.39	6.56	0.24	0.81	0.02	0.13
Central sub-Saharan Africa	**0.025**	-0.29	-0.03	0.54	0.09	3.08	0.04	0.83	0.02	0.29
	**0.50**	-0.09	0.09	0.67	0.24	4.11	0.06	0.89	0.02	0.15
	**0.975**	0.11	0.21	0.79	0.39	5.45	0.08	0.93	0.02	0.08
Middle East	**0.025**	-0.23	0.18	0.29	0.15	3.56	0.16	0.46	0.03	0.71
	**0.50**	0.17	0.29	0.43	0.28	5.44	0.26	0.70	0.02	0.32
	**0.975**	0.56	0.40	0.56	0.41	8.06	0.44	0.83	0.02	0.15
Central America and the Caribbean	**0.025**	-0.59	-0.01	0.23	0.33	2.86	0.12	0.83	0.11	0.49
	**0.50**	-0.24	0.14	0.38	0.49	4.38	0.19	0.91	0.09	0.22
	**0.975**	0.11	0.29	0.52	0.64	6.47	0.30	0.95	0.07	0.10
Western sub-Saharan Africa	**0.025**	-0.25	-0.06	0.43	0.36	2.40	0.08	0.81	0.05	0.21
	**0.50**	-0.10	0.02	0.51	0.47	2.80	0.10	0.85	0.05	0.11
	**0.975**	0.05	0.11	0.59	0.57	3.23	0.11	0.88	0.04	0.07
Andean South America	**0.025**	-0.41	0.05	0.38	0.08	2.42	0.07	0.89	0.04	0.77
	**0.50**	-0.01	0.20	0.54	0.26	3.66	0.10	0.94	0.04	0.29
	**0.975**	0.39	0.34	0.70	0.44	5.58	0.14	0.97	0.03	0.12
Southern sub-Saharan Africa	**0.025**	-0.35	-0.07	0.29	0.11	3.22	0.03	0.79	0.03	0.49
	**0.50**	-0.03	0.16	0.50	0.34	5.43	0.05	0.90	0.03	0.21
	**0.975**	0.28	0.39	0.70	0.57	9.30	0.10	0.96	0.02	0.09
Eastern sub-Saharan Africa	**0.025**	-0.69	-0.37	0.75	-0.14	-1.04	-190.21	0.71	0.06	0.91
	**0.50**	-0.27	-0.10	1.01	0.09	0.14	-23.96	0.95	0.04	0.34

	**0.975**	0.16	0.17	1.27	0.31	21.22	0.00	0.99	0.03	0.14
**Tropical South America**	**0.025**	-0.18	0.05	0.50	0.24	2.63	0.10	0.89	0.07	0.26
	**0.50**	-0.01	0.11	0.57	0.32	3.09	0.11	0.92	0.06	0.14
	**0.975**	0.16	0.18	0.63	0.40	3.73	0.14	0.94	0.05	0.08
**East Asia**	**0.025**	-0.65	-0.13	0.51	0.26	3.33	0.28	0.73	0.07	0.63
	**0.50**	-0.30	-0.01	0.62	0.39	4.23	0.37	0.84	0.06	0.27
	**0.975**	0.05	0.11	0.72	0.52	5.58	0.49	0.90	0.05	0.12
**Southeast Asia and Oceania**	**0.025**	-0.74	0.05	0.62	0.15	2.83	0.13	0.81	0.07	0.92
	**0.50**	-0.43	0.10	0.68	0.22	3.34	0.15	0.85	0.07	0.35
	**0.975**	-0.13	0.16	0.74	0.29	3.84	0.18	0.91	0.06	0.18
**South and Central Asia**	**0.025**	-0.53	-0.11	0.23	0.55	4.05	0.11	0.90	0.05	0.53
	**0.50**	-0.21	-0.01	0.33	0.67	5.09	0.14	0.93	0.04	0.24
	**0.975**	0.11	0.09	0.43	0.80	6.55	0.20	0.96	0.03	0.12

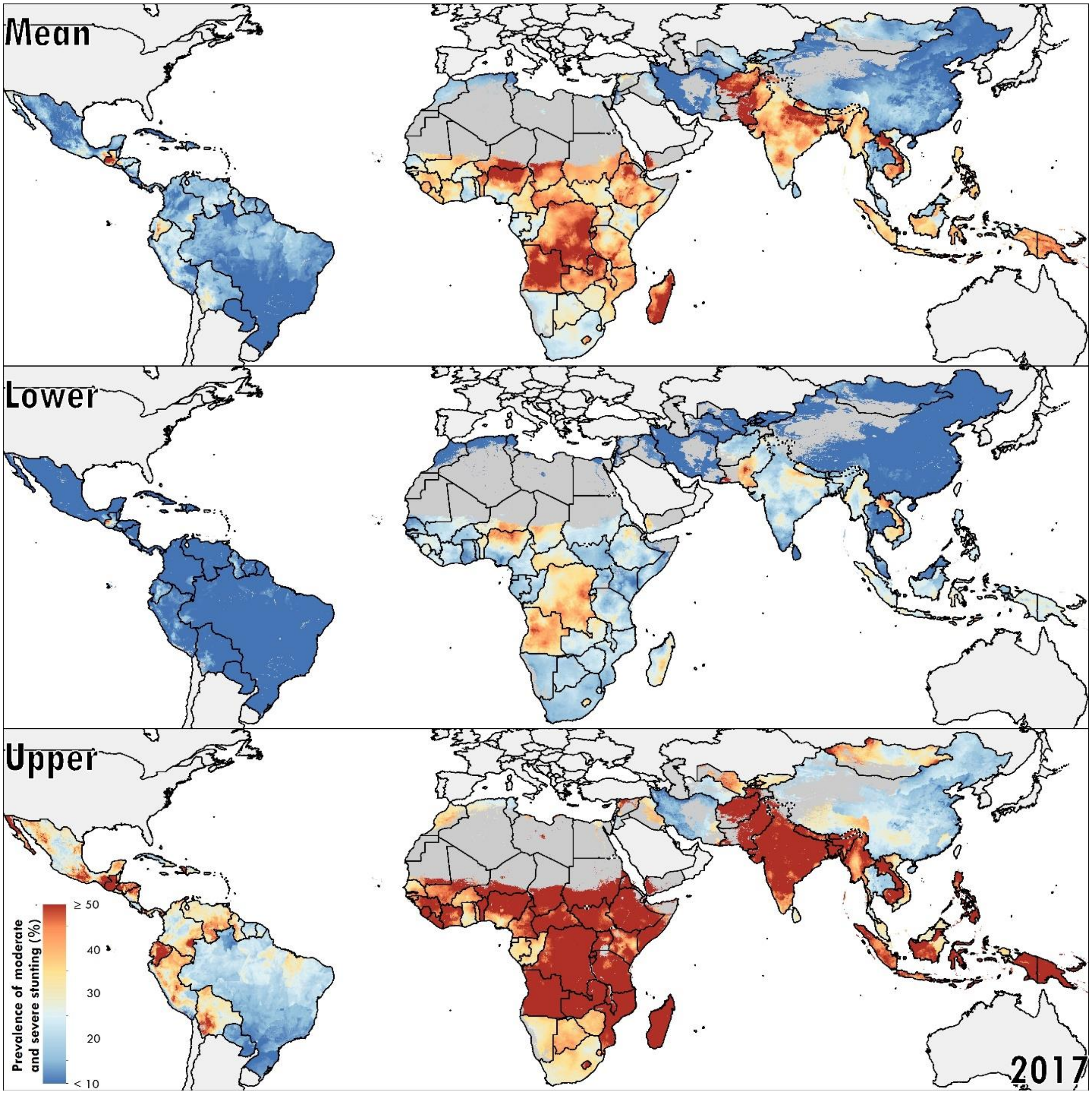

Supplementary Figure 21: Stunting posterior means and upper and lower 95% uncertainty intervals for 2017.

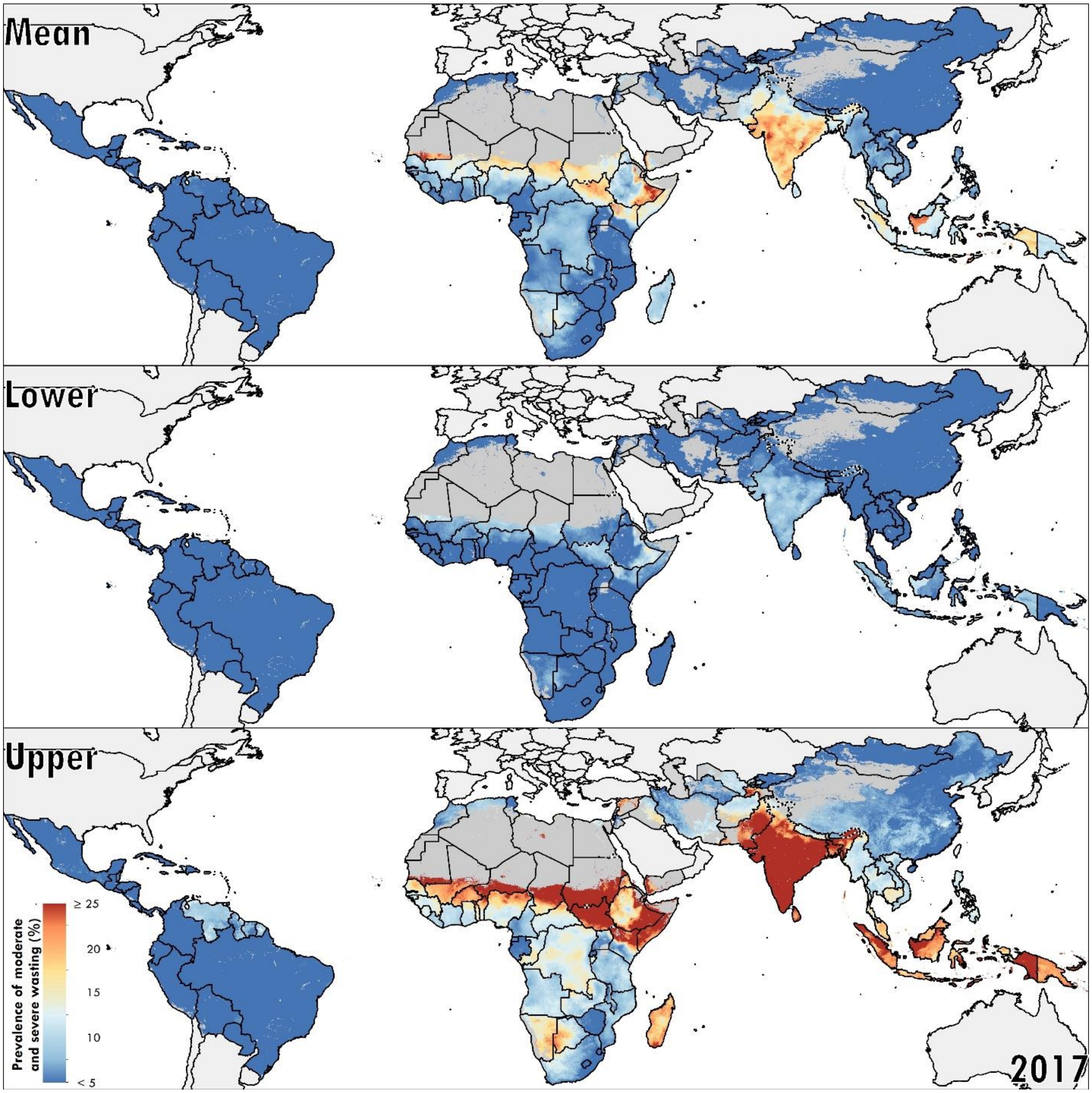

Supplementary Figure 22: Wasting posterior means and upper and lower 95% uncertainty intervals for 2017.

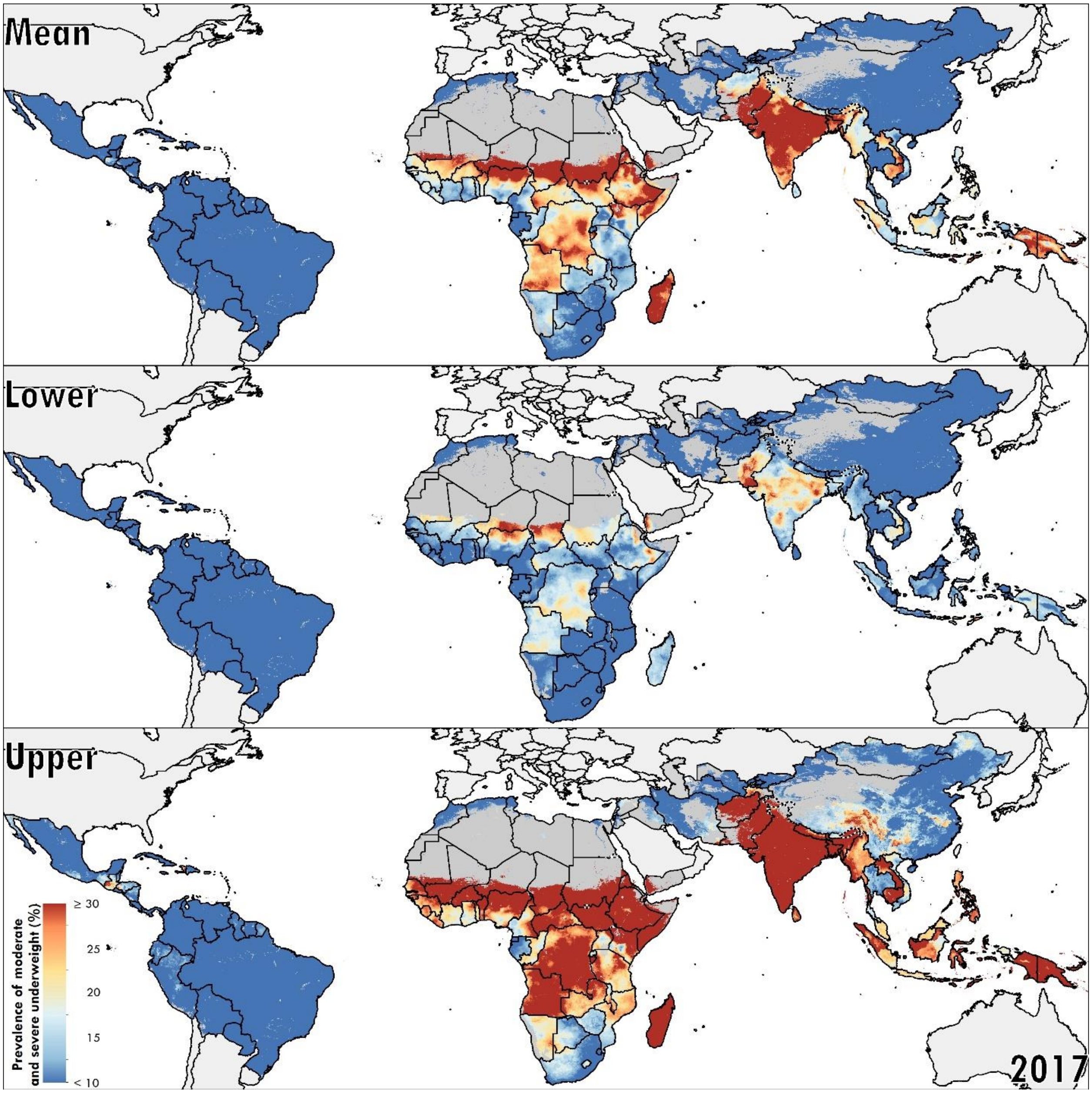

Supplementary Figure 23: Underweight posterior means and upper and lower 95% uncertainty intervals for 2017.

4.0 Supplementary Results

4.1 Additional information for figure descriptions

For Figure 1d (overlapping population-weighted quartiles for child stunting and relative 95% uncertainty for 2017):

Quartile cut-offs were 16.3% (25th percentile), 27.3% (50th percentile), 39.9% (75th percentile) for the stunting prevalence axis, and 0.758 (25th percentile), 1.094 (50th percentile), and 1.352 (75th percentile) for the relative uncertainty axis (calculated as the absolute range of the uncertainty intervals divided by the estimate).

For Figure 2d (overlapping population-weighted quartiles for child wasting and relative 95% uncertainty for 2017):

Quartile cut-offs were 3.5% (25th percentile), 6.8% (50th percentile), 12.6% (75th percentile) for the wasting prevalence axis, and 1.178 (25th percentile), 1.439 (50th percentile), and 1.758 (75th percentile) for the relative uncertainty axis (calculated as the absolute range of the uncertainty intervals divided by the estimate).

For Extended Data Figure 5d (overlapping population-weighted quartiles for child underweight and relative 95% uncertainty for 2017):

Quartile cut-offs were 5.5% (25th percentile), 10.8% (50th percentile), 26.3% (75th percentile) for the underweight prevalence axis, and 0.858 (25th percentile), 1.122 (50th percentile), and 1.703 (75th percentile) for the relative uncertainty axis (calculated as the absolute range of the uncertainty intervals divided by the estimate).

For Figures 1g, 2g, and Extended Data Figure 5g (2000-2017 annualised decreases in CGF indicators relative to rates needed during 2017–2025 to meet WHO GNT):

100% indicates the annualised decrease from 2000 to 2017 is equivalent to the pace of progress required during 2017–2025 to meet WHO GNT by 2025 (40% reduction in stunting or underweight; wasting prevalence less than 5%) relative to 2010. Blue and green grid cells exceeded this pace; yellow grid cells proceeded at a slower rate than required; orange grid cells were non-decreasing; and purple grid cells were estimated to have met the target by 2017 (‘Met GNT’).

For Figures 1h, 2h, and Extended Data Figure 5h:

Grid-cell-level (5 × 5-km resolution) predicted prevalence in 2025 is based on annualised decrease achieved from 2000 to 2017 and projected from 2017.

For Figures 1, 2, and Extended Data Figures 5, 7, and 8:
Maps reflect administrative boundaries, land cover, lakes, and population; grey-coloured grid cells had fewer than ten people per 1 × 1-km grid cell and were classified as "barren or sparsely vegetated"[51–57], or were not included in these analyses. Interactive visualisation tools are available at https://vizhub.healthdata.org/lbd/cgf.

4.2 Countries estimated to meet WHO GNT in 2017 and 2025 at various spatial levels

Supplementary Table 12: Countries predicted to have met WHO GNTs by 2017, with >95% probability.

"Yes" indicates that the country was estimated to have met WHO GNT for either stunting, wasting, or underweight in 2017 at the national level (Admin 0), in all first administrative-level units (Admin 1), and/or in all second administrative-level units (Admin 2), with >95% probability.

Country	Stunting			Wasting			Underweight		
	Admin 0	*Admin 1*	*Admin 2*	*Admin 0*	*Admin 1*	*Admin 2*	*Admin 0*	*Admin 1*	*Admin 2*
Afghanistan									
Algeria	Yes	Yes					Yes	Yes	
Angola									
Bangladesh									
Belize	Yes			Yes	Yes	Yes	Yes	Yes	Yes
Benin									
Bhutan							Yes	Yes	
Bolivia	Yes			Yes	Yes	Yes	Yes	Yes	Yes
Botswana							Yes	Yes	Yes
Brazil	Yes	Yes		Yes	Yes		Yes	Yes	
Burkina Faso									
Burundi									
Cambodia									
Cameroon							Yes		
Cape Verde	Yes	Yes	Yes	Yes			Yes	Yes	Yes
Central African Republic									

Country	Stunting			Wasting			Underweight		
	Admin 0	*Admin 1*	*Admin 2*	*Admin 0*	*Admin 1*	*Admin 2*	*Admin 0*	*Admin 1*	*Admin 2*
Chad									
China	Yes	Yes		Yes			Yes	Yes	
Colombia	Yes			Yes			Yes		
Comoros									
Costa Rica	Yes	Yes	Yes	Yes	Yes	Yes	Yes	Yes	Yes
Côte d'Ivoire							Yes	Yes	Yes
Cuba	Yes	Yes		Yes	Yes		Yes	Yes	
Democratic Republic of the Congo									
Djibouti									
Dominican Republic	Yes			Yes	Yes	Yes	Yes	Yes	Yes
Ecuador	Yes			Yes			Yes	Yes	Yes
Egypt	Yes						Yes	Yes	
El Salvador	Yes	Yes		Yes	Yes	Yes	Yes	Yes	Yes
Equatorial Guinea	Yes			Yes			Yes		
Eritrea									
Ethiopia									
French Guiana									
Gabon	Yes			Yes			Yes	Yes	Yes
Ghana	Yes						Yes	Yes	
Guatemala				Yes	Yes	Yes	Yes		
Guinea							Yes		
Guinea-Bissau							Yes		
Guyana	Yes						Yes	Yes	
Haiti							Yes		
Honduras	Yes			Yes	Yes	Yes	Yes	Yes	
India									
Indonesia							Yes		
Iran	Yes	Yes		Yes			Yes	Yes	
Iraq	Yes						Yes	Yes	Yes

Country	Stunting			Wasting			Underweight		
	Admin 0	*Admin 1*	*Admin 2*	*Admin 0*	*Admin 1*	*Admin 2*	*Admin 0*	*Admin 1*	*Admin 2*
Jamaica	Yes			Yes			Yes		
Jordan	Yes	Yes	Yes	Yes	Yes		Yes	Yes	Yes
Kenya							Yes		
Kyrgyzstan	Yes	Yes	Yes	Yes	Yes	Yes	Yes	Yes	Yes
Laos									
Lesotho				Yes	Yes	Yes	Yes	Yes	Yes
Liberia							Yes		
Libya							Yes	Yes	Yes
Madagascar									
Malawi				Yes			Yes		
Malaysia	Yes						Yes		
Mali									
Mauritania									
Mexico	Yes			Yes			Yes	Yes	
Mongolia	Yes			Yes	Yes		Yes	Yes	Yes
Morocco	Yes			Yes			Yes	Yes	Yes
Mozambique							Yes	Yes	
Myanmar									
Namibia	Yes						Yes		
Nepal									
Nicaragua	Yes			Yes	Yes	Yes	Yes	Yes	Yes
Niger									
Nigeria									
Pakistan									
Palestine	Yes	Yes	Yes	Yes	Yes	Yes	Yes	Yes	Yes
Panama	Yes			Yes	Yes	Yes	Yes	Yes	Yes
Papua New Guinea									
Paraguay	Yes	Yes	Yes	Yes	Yes	Yes	Yes	Yes	Yes
Peru	Yes			Yes	Yes	Yes	Yes	Yes	Yes

Country	Stunting			Wasting			Underweight		
	Admin 0	*Admin 1*	*Admin 2*	*Admin 0*	*Admin 1*	*Admin 2*	*Admin 0*	*Admin 1*	*Admin 2*
Philippines									
Republic of the Congo	Yes						Yes	Yes	
Rwanda				Yes	Yes		Yes	Yes	Yes
São Tomé and Príncipe	Yes	Yes	Yes	Yes	Yes	Yes	Yes	Yes	Yes
Senegal	Yes						Yes		
Sierra Leone							Yes		
Somalia									
South Africa	Yes			Yes			Yes	Yes	Yes
South Sudan									
Sri Lanka	Yes	Yes					Yes		
Sudan									
Suriname	Yes	Yes					Yes	Yes	Yes
Swaziland (eSwatini)				Yes	Yes	Yes	Yes	Yes	Yes
Syria							Yes	Yes	Yes
Tajikistan							Yes	Yes	
Tanzania							Yes		
Thailand	Yes						Yes	Yes	
The Gambia							Yes		
Timor-Leste									
Togo							Yes		
Trinidad and Tobago	Yes						Yes		
Tunisia	Yes	Yes		Yes			Yes	Yes	
Turkmenistan	Yes	Yes	Yes	Yes	Yes	Yes	Yes	Yes	Yes
Uganda				Yes			Yes		
Uzbekistan	Yes						Yes	Yes	Yes
Venezuela	Yes						Yes		
Vietnam	Yes						Yes	Yes	
Western Sahara									
Yemen									

Country	Stunting			Wasting			Underweight		
	Admin 0	*Admin 1*	*Admin 2*	*Admin 0*	*Admin 1*	*Admin 2*	*Admin 0*	*Admin 1*	*Admin 2*
Zambia							Yes		
Zimbabwe				Yes	Yes		Yes	Yes	Yes

Supplementary Table 13: Countries predicted to meet WHO GNTs by 2025, with >95% probability.

"Yes" indicates that the country is predicted to meet WHO GNT for either stunting, wasting, or underweight in 2025 at the national level (Admin 0), in all first administrative-level units (Admin 1), and/or in all second administrative-level units (Admin 2), with >95% probability.

Country	Stunting			Wasting			Underweight		
	Admin 0	*Admin 1*	*Admin 2*	*Admin 0*	*Admin 1*	*Admin 2*	*Admin 0*	*Admin 1*	*Admin 2*
Afghanistan									
Algeria	Yes	Yes					Yes	Yes	
Angola							Yes		
Bangladesh									
Belize	Yes			Yes	Yes	Yes	Yes	Yes	Yes
Benin									
Bhutan							Yes	Yes	
Bolivia	Yes			Yes	Yes		Yes	Yes	Yes
Botswana							Yes	Yes	
Brazil	Yes	Yes		Yes			Yes	Yes	
Burkina Faso	Yes								
Burundi									
Cambodia									
Cameroon							Yes		
Cape Verde	Yes	Yes	Yes	Yes			Yes	Yes	Yes
Central African Republic									
Chad									
China	Yes	Yes		Yes			Yes	Yes	
Colombia	Yes			Yes			Yes		

Country	Stunting			Wasting			Underweight		
	Admin 0	*Admin 1*	*Admin 2*	*Admin 0*	*Admin 1*	*Admin 2*	*Admin 0*	*Admin 1*	*Admin 2*
Comoros									
Costa Rica	Yes	Yes		Yes	Yes		Yes	Yes	Yes
Côte d'Ivoire							Yes	Yes	
Cuba	Yes	Yes		Yes	Yes		Yes	Yes	
Democratic Republic of the Congo							Yes		
Djibouti									
Dominican Republic	Yes			Yes			Yes	Yes	Yes
Ecuador	Yes			Yes			Yes	Yes	Yes
Egypt	Yes						Yes		
El Salvador	Yes	Yes		Yes	Yes		Yes	Yes	
Equatorial Guinea	Yes			Yes			Yes		
Eritrea									
Ethiopia									
French Guiana									
Gabon	Yes			Yes			Yes	Yes	Yes
Ghana	Yes						Yes	Yes	
Guatemala				Yes	Yes	Yes	Yes		
Guinea							Yes		
Guinea-Bissau									
Guyana	Yes						Yes	Yes	
Haiti							Yes		
Honduras	Yes			Yes	Yes		Yes	Yes	
India									
Indonesia							Yes		
Iran	Yes	Yes					Yes	Yes	
Iraq	Yes						Yes	Yes	Yes
Jamaica	Yes			Yes			Yes		
Jordan	Yes	Yes	Yes	Yes			Yes	Yes	Yes
Kenya							Yes		

Country	Stunting			Wasting			Underweight		
	Admin 0	*Admin 1*	*Admin 2*	*Admin 0*	*Admin 1*	*Admin 2*	*Admin 0*	*Admin 1*	*Admin 2*
Kyrgyzstan	Yes	Yes	Yes	Yes			Yes	Yes	Yes
Laos							Yes		
Lesotho				Yes	Yes	Yes	Yes	Yes	Yes
Liberia							Yes		
Libya							Yes	Yes	Yes
Madagascar									
Malawi				Yes			Yes		
Malaysia	Yes						Yes		
Mali									
Mauritania									
Mexico	Yes			Yes			Yes	Yes	
Mongolia	Yes			Yes			Yes	Yes	
Morocco	Yes			Yes			Yes	Yes	Yes
Mozambique				Yes			Yes	Yes	Yes
Myanmar							Yes		
Namibia	Yes						Yes		
Nepal									
Nicaragua	Yes			Yes			Yes	Yes	Yes
Niger									
Nigeria									
Pakistan									
Palestine	Yes	Yes	Yes	Yes	Yes	Yes	Yes	Yes	Yes
Panama	Yes			Yes	Yes	Yes	Yes	Yes	Yes
Papua New Guinea									
Paraguay	Yes	Yes	Yes	Yes	Yes	Yes	Yes	Yes	Yes
Peru	Yes	Yes	Yes	Yes	Yes	Yes	Yes	Yes	Yes
Philippines							Yes		
Republic of the Congo	Yes						Yes	Yes	
Rwanda				Yes	Yes	Yes	Yes	Yes	Yes

Country	Stunting			Wasting			Underweight		
	Admin 0	*Admin 1*	*Admin 2*	*Admin 0*	*Admin 1*	*Admin 2*	*Admin 0*	*Admin 1*	*Admin 2*
São Tomé and Príncipe	Yes	Yes	Yes	Yes	Yes	Yes	Yes	Yes	Yes
Senegal	Yes						Yes		
Sierra Leone							Yes	Yes	
Somalia									
South Africa	Yes			Yes			Yes	Yes	Yes
South Sudan									
Sri Lanka	Yes	Yes					Yes		
Sudan									
Suriname	Yes	Yes					Yes	Yes	Yes
Swaziland (eSwatini)				Yes	Yes	Yes	Yes	Yes	Yes
Syria							Yes		
Tajikistan							Yes		
Tanzania							Yes		
Thailand	Yes						Yes		
The Gambia									
Timor-Leste									
Togo							Yes	Yes	
Trinidad and Tobago	Yes						Yes		
Tunisia	Yes	Yes		Yes			Yes	Yes	
Turkmenistan	Yes	Yes	Yes	Yes	Yes	Yes	Yes	Yes	Yes
Uganda				Yes			Yes		
Uzbekistan	Yes						Yes	Yes	Yes
Venezuela	Yes						Yes		
Vietnam	Yes						Yes	Yes	
Western Sahara									
Yemen									
Zambia							Yes		
Zimbabwe				Yes	Yes		Yes	Yes	Yes

5.0 Model Validation

5.1 In-sample metrics

In order to assess the in-sample performance of our models and compare to national-level estimates produced by GBD, we generated a suite of diagnostic plots for each CGF indicator estimates in each of the regions and countries modelled.

To explore residual error over space and time, absolute error (data minus predicted posterior mean estimates at the corresponding grid cells) were produced.

5.2 Metrics of predictive validity

In order to assess the predictive validity of our estimates, we validated our models using spatially-stratified five-fold out-of-sample cross-validation[58]. To construct each spatial fold, we used a modified bi-tree algorithm to spatially aggregate data points. This algorithm recursively partitions two-dimensional space, alternating between horizontal and vertical splits on the weighted data sample size medians, until the data contained within each spatial partition are of a similar sample size. The depth of recursive partitioning is constrained by the target sample size within a partition and the minimum number of clusters or pseudo-clusters allowed within each spatial partition (in this case, a minimum sample size of 500 was used). These spatial partitions are then allocated to one of five folds for cross-validation. For validation, each geostatistical model was run five times, each time holding out data from one of the folds, generating a set of out-of-sample predictions for the held-out data. For each indicator, a full suite of out-of-sample predictions over the entire dataset was generated by combining the out-of-sample predictions from the five cross-validation runs.

Using these out-of-sample predictions, we then calculated mean error (ME, or bias), root-mean-squared-error (RMSE, which summarises total variance), coefficient of variation (CoV, defined to be the standard deviation divided by the mean and multiplied by 100, which is a measure of relative variability), and 95% coverage of our predictive intervals (the proportion of observed out-of-sample data that fall within our predicted 95% credible intervals) aggregated up to different administrative levels (levels 0, 1, and 2) as defined by Database of Global Administrative Areas (GADM)[54]. Administrative level 0 (Admin 0) borders correspond to national boundaries, first administrative level (Admin 1) borders generally correspond to regions, provinces, or state-level boundaries within a country, and second administrative level (Admin 2) borders correspond to the next finer unit-level, often districts, within regions. These metrics are summarised in Supplementary Tables 14–22 for each CGF indicator and are calculated across all regions. Included in the sample tables for comparison are the same metrics calculated on in-sample predictions.

5.2.1 Stunting validation metrics

The out-of-sample (OOS) column indicates whether the metric was calculated using in-sample or out-of-sample predictions. Mean error, root-mean-squared-error (RMSE), Correlation (Corr), and coefficient of variation (Cov) are in proportion.

Supplementary Table 14: Predictive metrics for stunting aggregated to the national level (Admin 0).

Year	OOS	Median SS	Mean err.	RMSE	Corr.	95% Cov.
**2000**	FALSE	4728.000	0.001	0.015	0.994	0.957
**2005**	FALSE	4947.500	0.003	0.015	0.994	0.954
**2010**	FALSE	6275.000	0.000	0.019	0.983	0.949
**2017**	FALSE	6043.000	-0.002	0.013	0.995	0.940

Supplementary Table 15: Predictive metrics for stunting aggregated to the first administrative level (Admin 1).

Year	OOS	Median SS	Mean err.	RMSE	Corr.	95% Cov.
**2000**	FALSE	238.000	0.001	0.038	0.970	0.954
**2005**	FALSE	303.000	0.003	0.034	0.974	0.955
**2010**	FALSE	326.000	0.000	0.040	0.953	0.948
**2017**	FALSE	346.000	-0.002	0.026	0.984	0.940

Supplementary Table 16: Predictive metrics for stunting aggregated to the second administrative level (Admin 2).

Year	OOS	Median SS	Mean err.	RMSE	Corr.	95% Cov.
**2000**	FALSE	24.867	0.001	0.061	0.930	0.955
**2005**	FALSE	13.994	0.003	0.056	0.932	0.955
**2010**	FALSE	34.940	0.000	0.063	0.896	0.949
**2017**	FALSE	43.559	-0.002	0.053	0.936	0.940

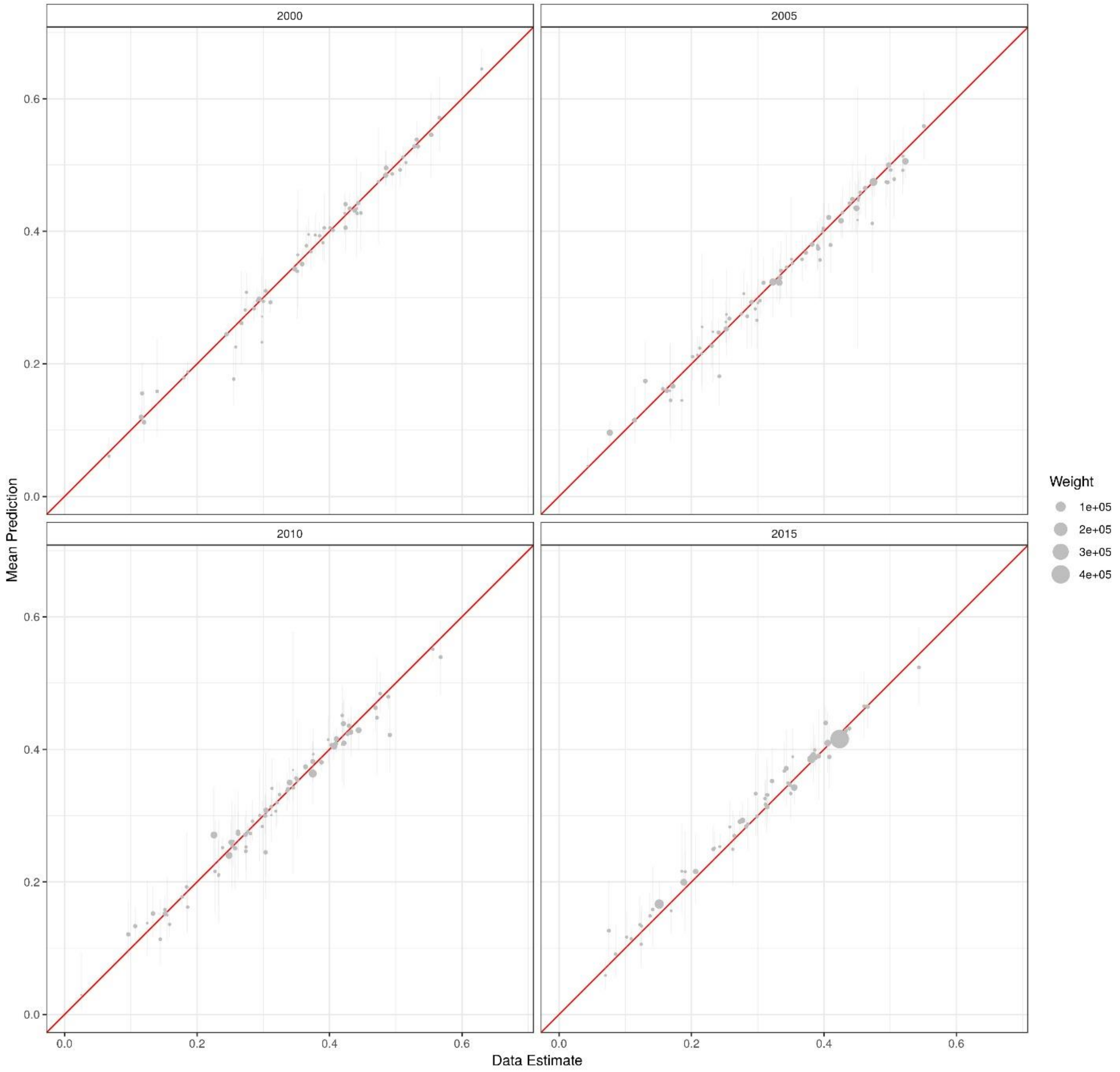

Supplementary Figure 24: Stunting national-level (Admin 0) aggregation in-sample.

Comparison of in-sample stunting predictions aggregated to Admin 0 with 95% uncertainty intervals plotted against Admin 0 aggregated data observations.

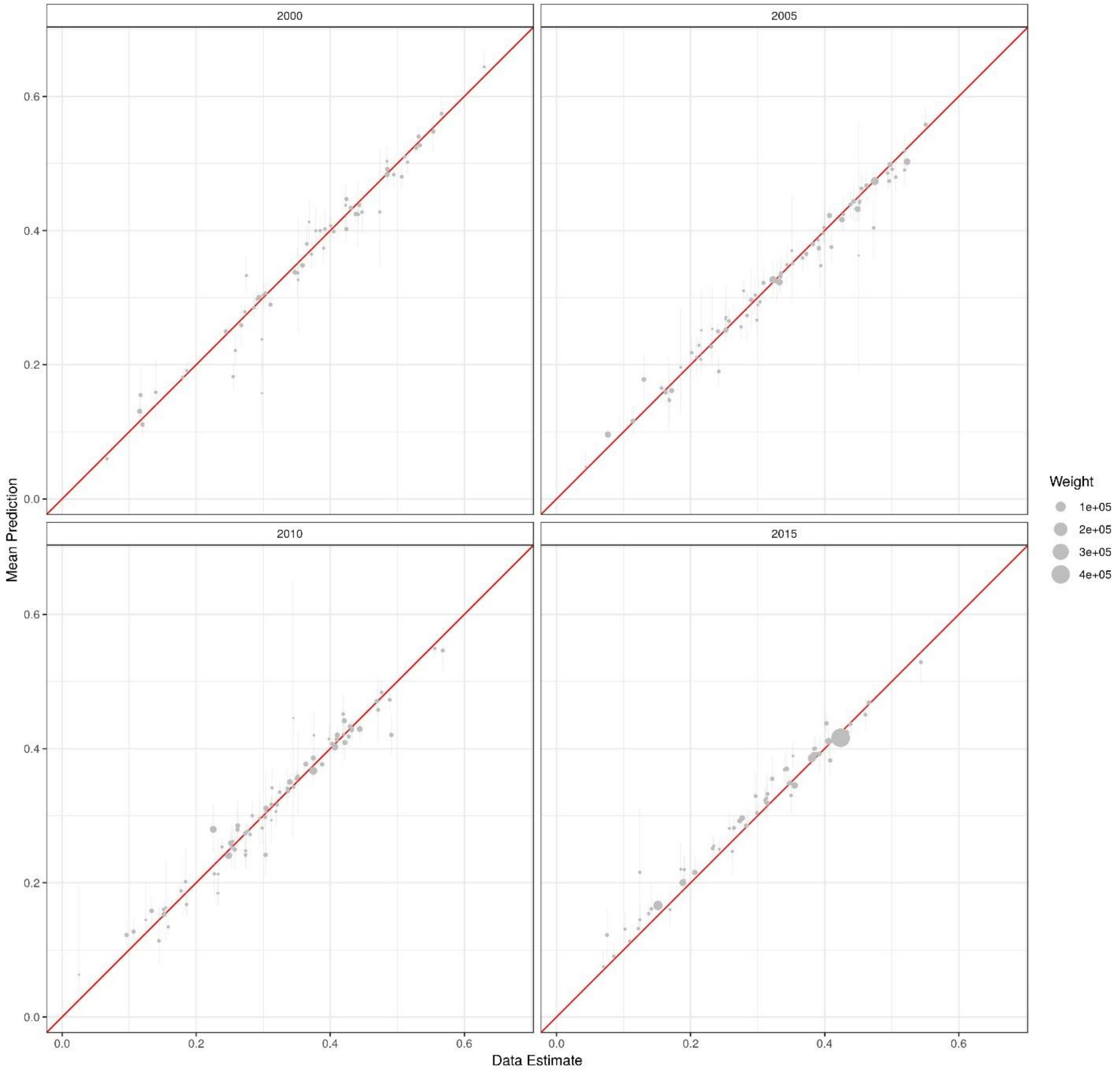

Supplementary Figure 25: Stunting national-level (Admin 0) aggregation out-of-sample.

Comparison of out-of-sample stunting predictions aggregated to Admin 0 with 95% uncertainty intervals plotted against Admin 0 aggregated data observations.

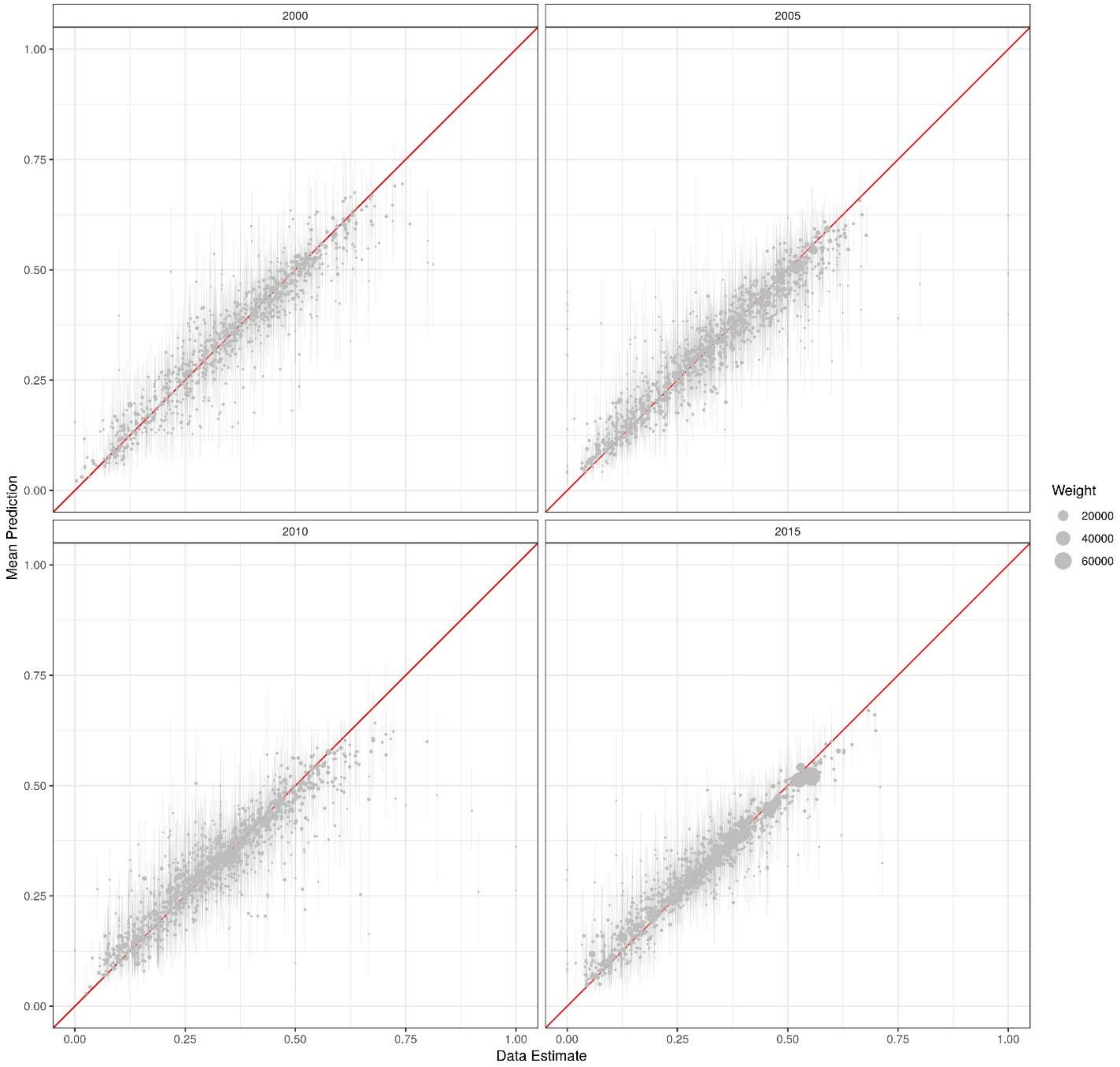

Supplementary Figure 26: Stunting first-administrative-level (Admin 1) aggregation in-sample.

Comparison of in-sample stunting predictions aggregated to Admin 1 with 95% uncertainty intervals plotted against Admin 1 aggregated data observations.

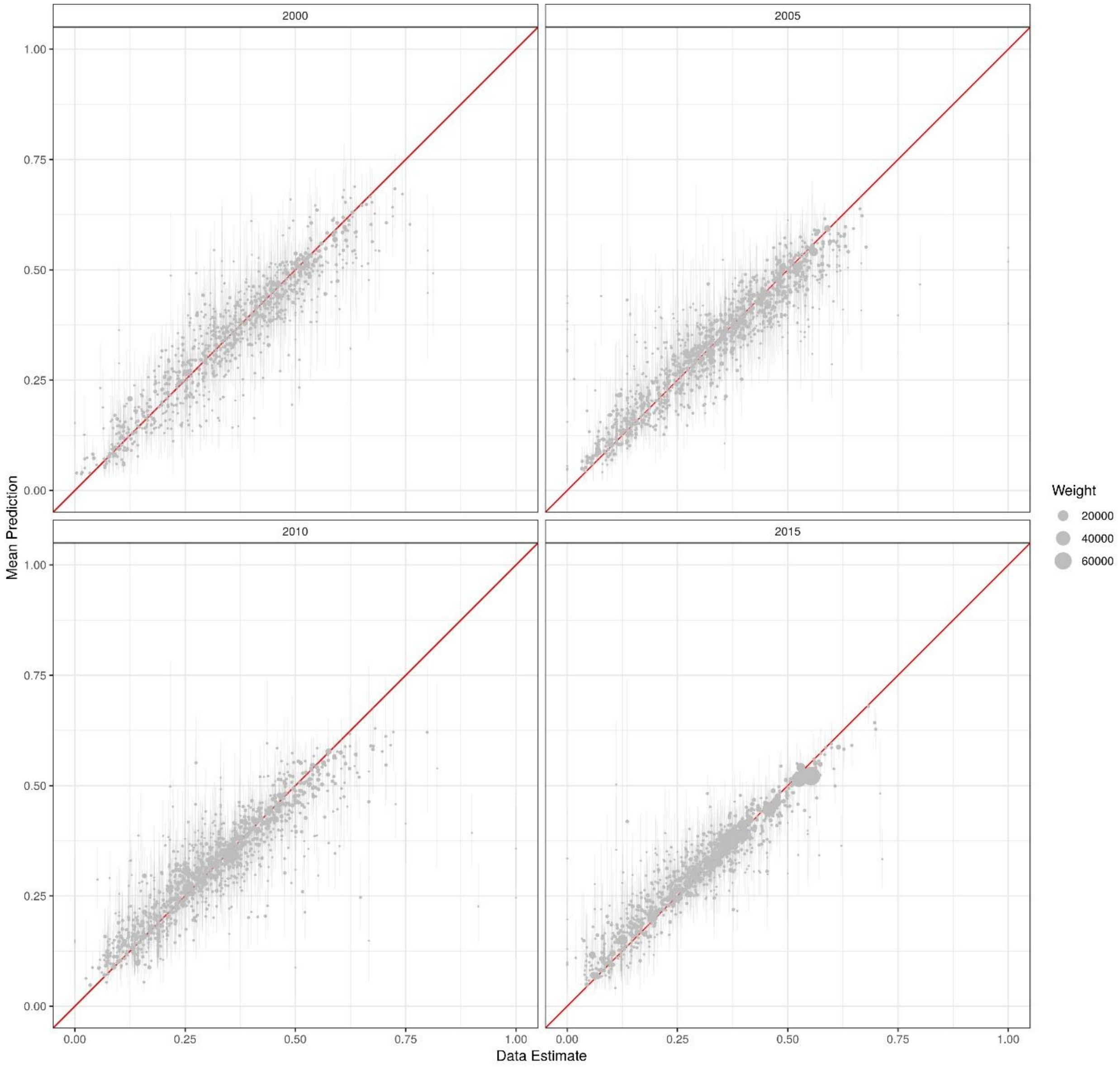

Supplementary Figure 27: Stunting first-administrative-level (Admin 1) aggregation out-of-sample.

Comparison of out-of-sample stunting predictions aggregated to Admin 1 with 95% uncertainty intervals plotted against Admin 1 aggregated data observations.

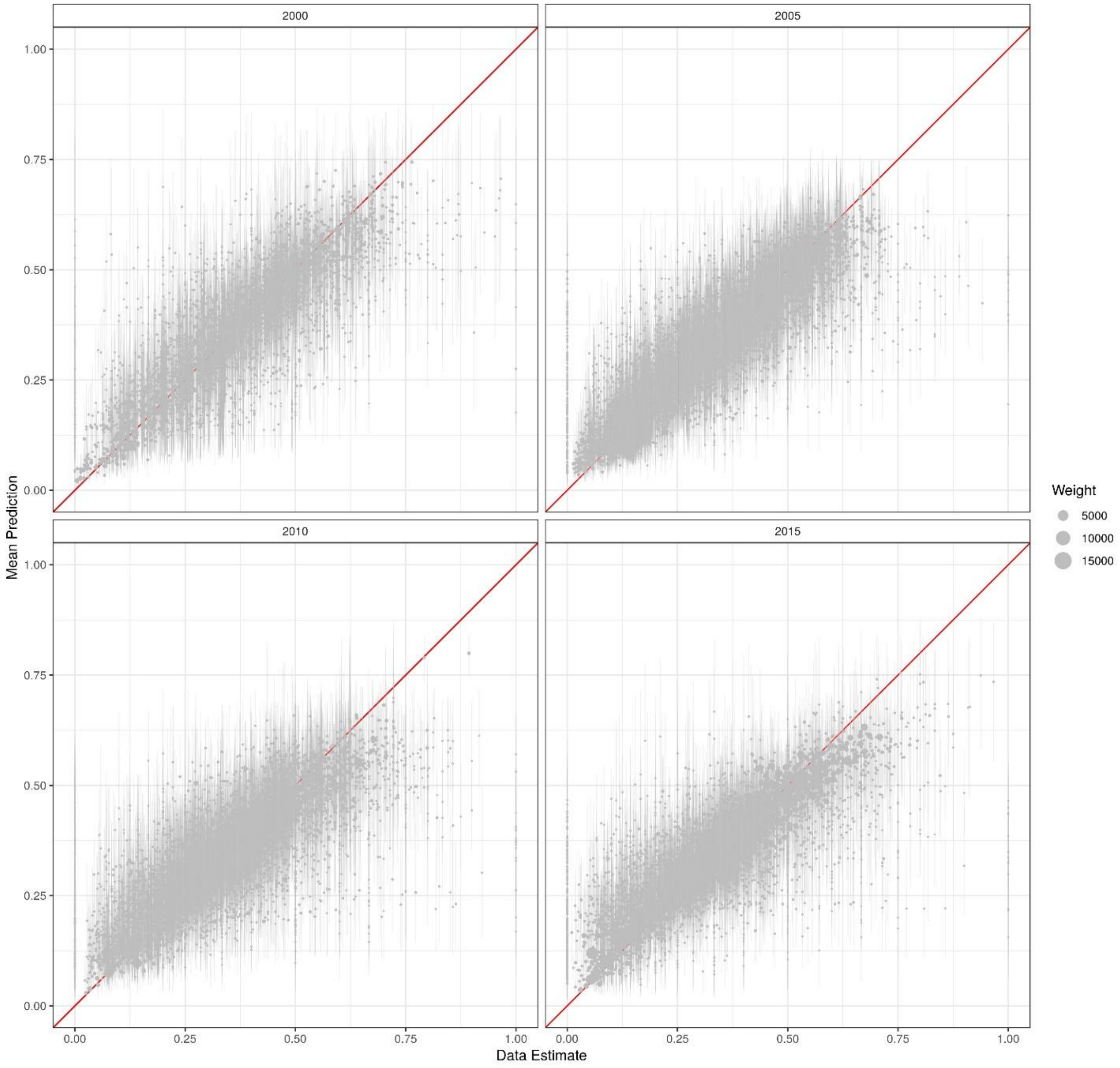

Supplementary Figure 28: Stunting second-administrative-level (Admin 2) aggregation in-sample.

Comparison of in-sample stunting predictions aggregated to Admin 2 with 95% uncertainty intervals plotted against Admin 2 aggregated data observations.

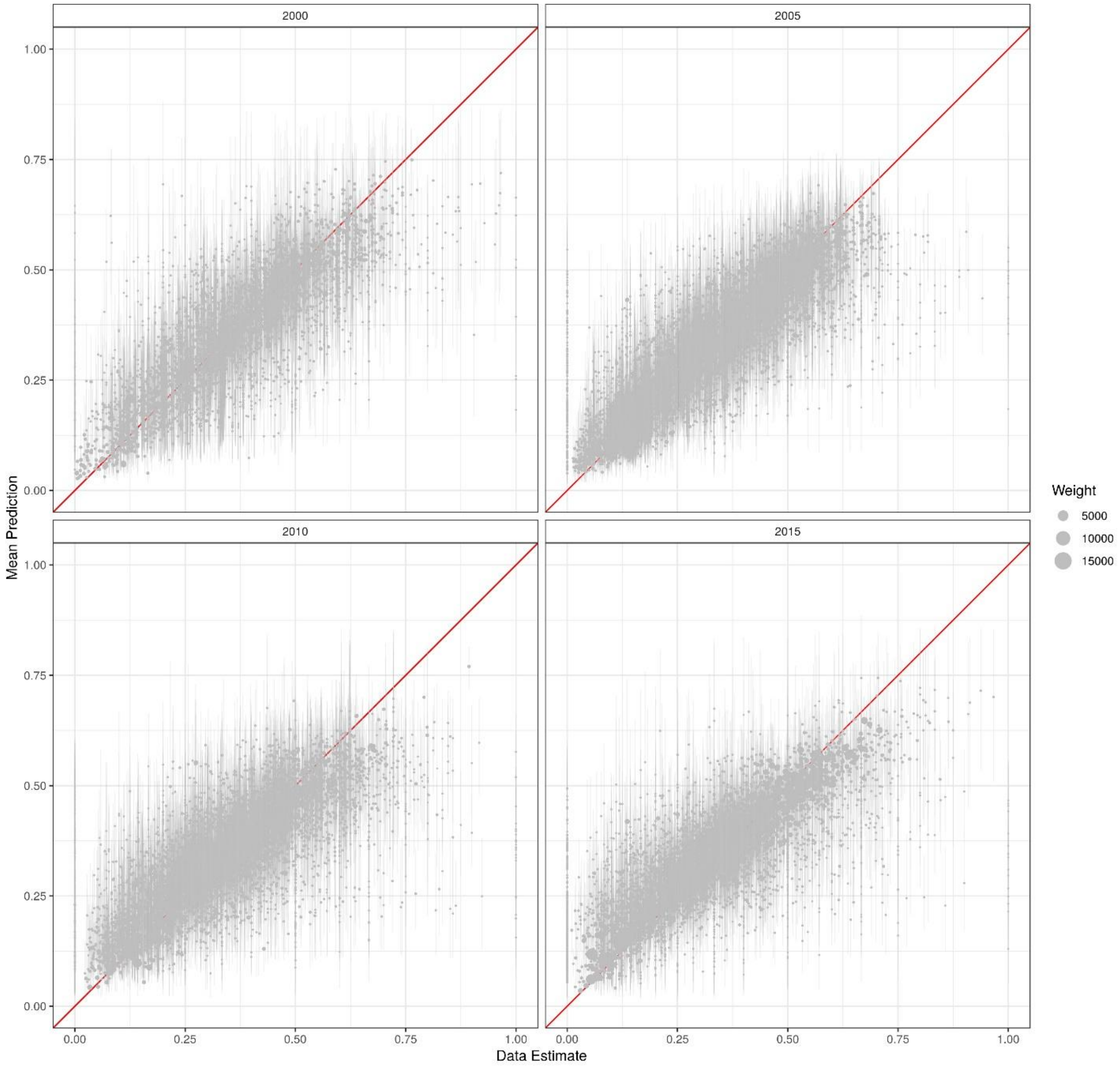

Supplementary Figure 29: Stunting second-administrative-level (Admin 2) aggregation out-of-sample.

Comparison of out-of-sample stunting predictions aggregated to Admin 2 with 95% uncertainty intervals plotted against Admin 2 aggregated data observations.

5.2.2 Wasting validation metrics

The out-of-sample (OOS) column indicates whether the metric was calculated using in-sample or out-of-sample predictions. Mean error, root-mean-squared-error (RMSE), Correlation (Corr), and coefficient of variation (Cov) are in proportion.

Supplementary Table 17: Predictive metrics for wasting aggregated to the national level (Admin 0).

Year	OOS	Median SS	Mean err.	RMSE	Corr.	95% Cov.
**2000**	FALSE	4722.000	0.001	0.008	0.992	0.962
**2005**	FALSE	5130.500	0.002	0.012	0.975	0.946
**2010**	FALSE	6268.000	-0.001	0.010	0.984	0.964
**2017**	FALSE	6052.000	0.000	0.006	0.997	0.946

Supplementary Table 18: Predictive metrics for wasting aggregated to the first administrative level (Admin 1).

Year	OOS	Median SS	Mean err.	RMSE	Corr.	95% Cov.
**2000**	FALSE	239.139	0.001	0.022	0.945	0.964
**2005**	FALSE	307.000	0.002	0.022	0.934	0.946
**2010**	FALSE	329.822	-0.001	0.020	0.946	0.962
**2017**	FALSE	346.000	0.000	0.018	0.977	0.945

Supplementary Table 19: Predictive metrics for wasting aggregated to the second administrative level (Admin 2).

Year	OOS	Median SS	Mean err.	RMSE	Corr.	95% Cov.
**2000**	FALSE	24.097	0.001	0.032	0.895	0.963
**2005**	FALSE	13.794	0.002	0.034	0.863	0.946
**2010**	FALSE	34.989	-0.001	0.033	0.880	0.963
**2017**	FALSE	44.000	0.000	0.033	0.928	0.947

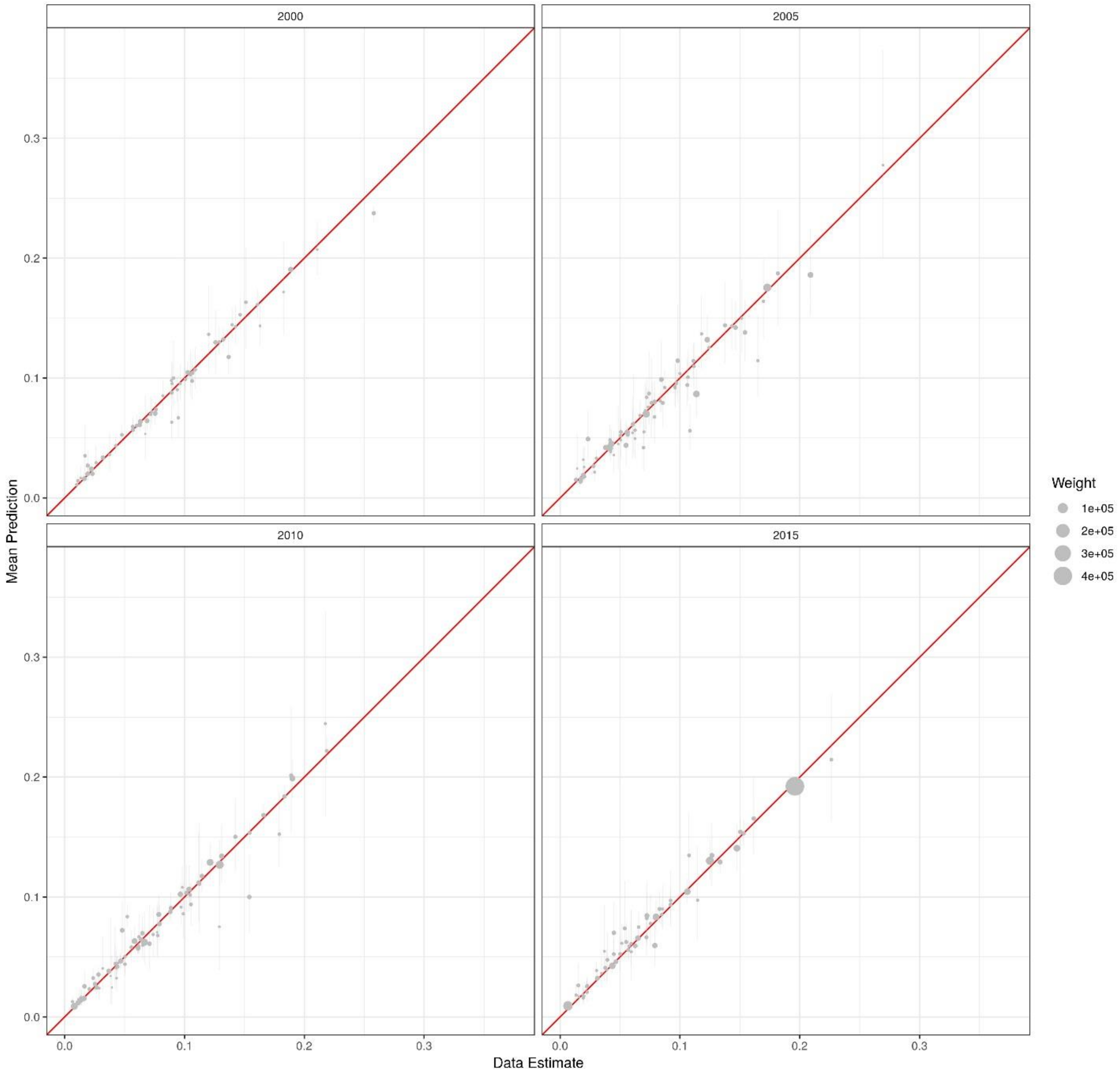

Supplementary Figure 30: Wasting national-level (Admin 0) aggregation in-sample.

Comparison of in-sample wasting predictions aggregated to Admin 0 with 95% uncertainty intervals plotted against Admin 0 aggregated data observations.

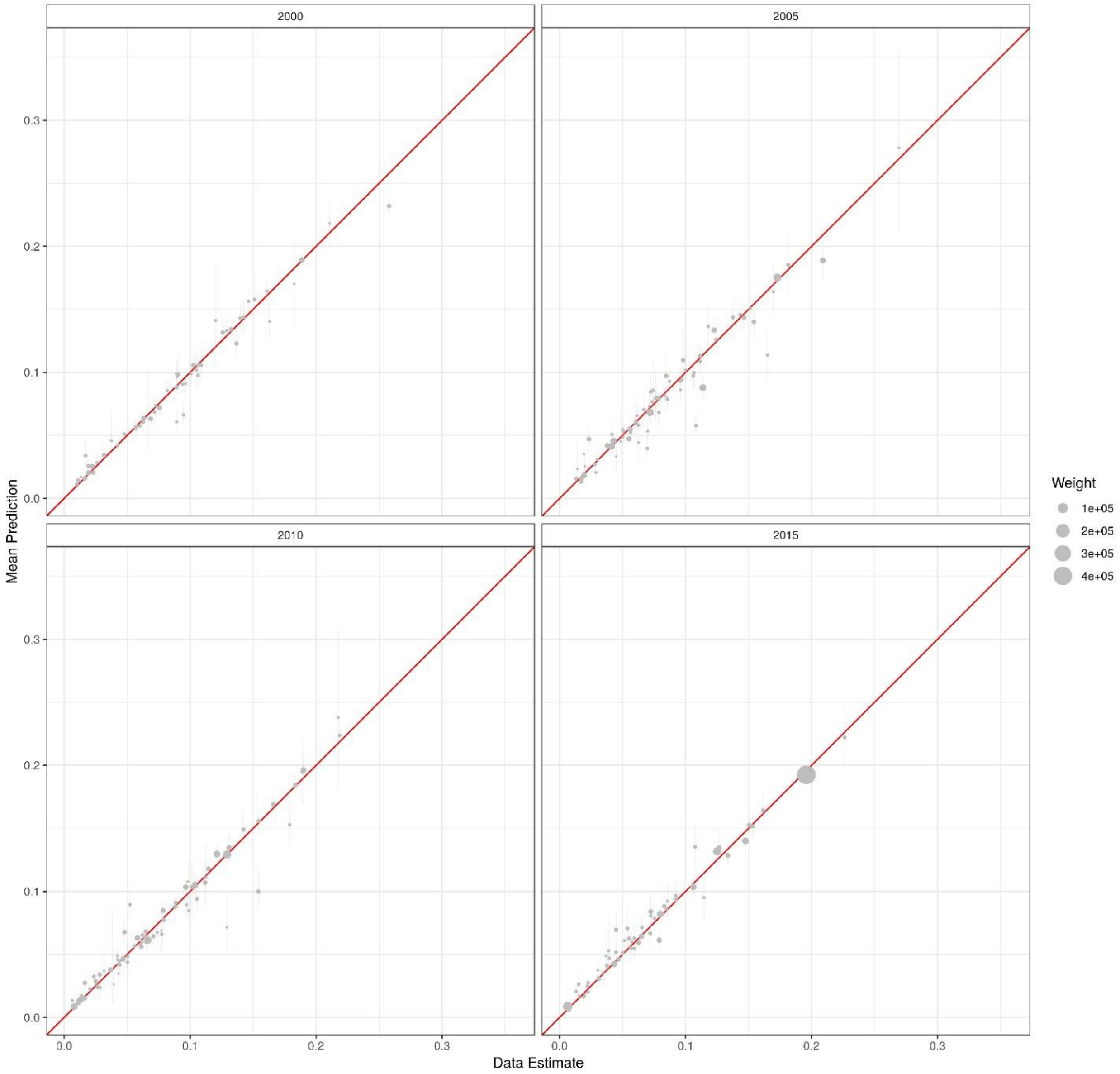

Supplementary Figure 31: Wasting national-level (Admin 0) aggregation out-of-sample.

Comparison of out-of-sample wasting predictions aggregated to Admin 0 with 95% uncertainty intervals plotted against Admin 0 aggregated data observations.

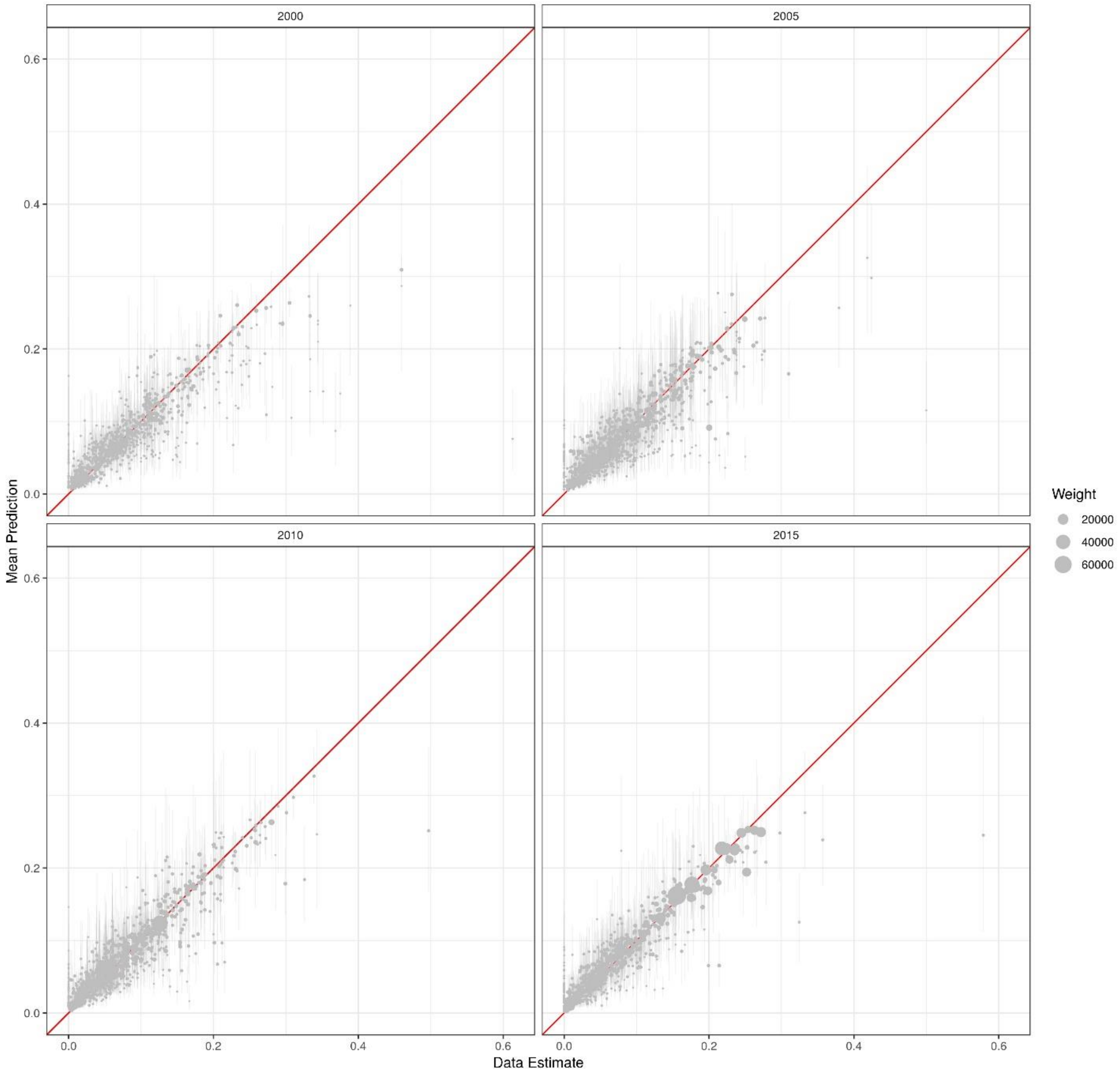

Supplementary Figure 32: Wasting first-administrative-level (Admin 1) aggregation in-sample.

Comparison of in-sample wasting predictions aggregated to Admin 1 with 95% uncertainty intervals plotted against Admin 1 aggregated data observations.

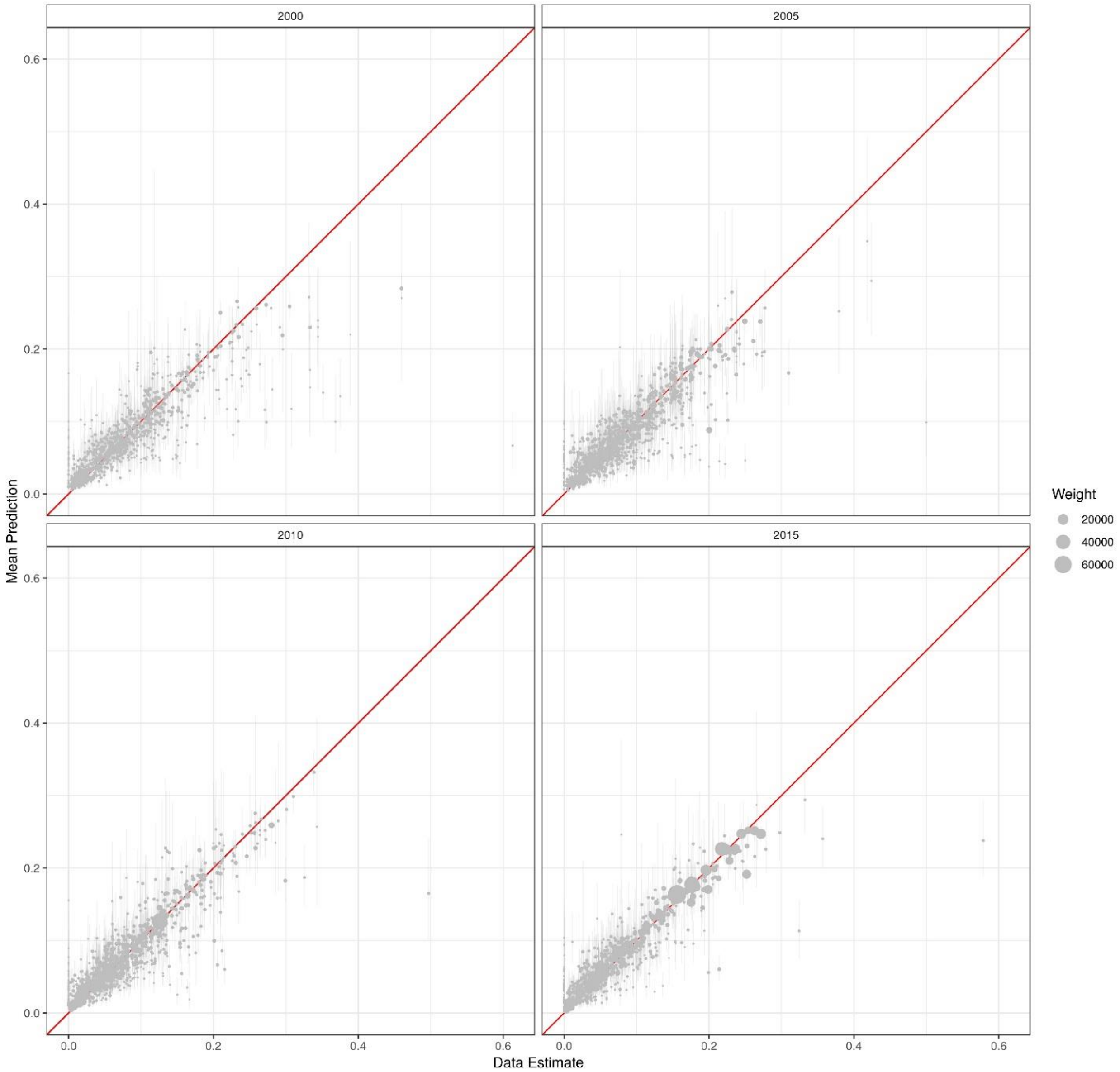

Supplementary Figure 33: Wasting first-administrative-level (Admin 1) aggregation out-of-sample.

Comparison of out-of-sample wasting predictions aggregated to Admin 1 with 95% uncertainty intervals plotted against Admin 1 aggregated data observations.

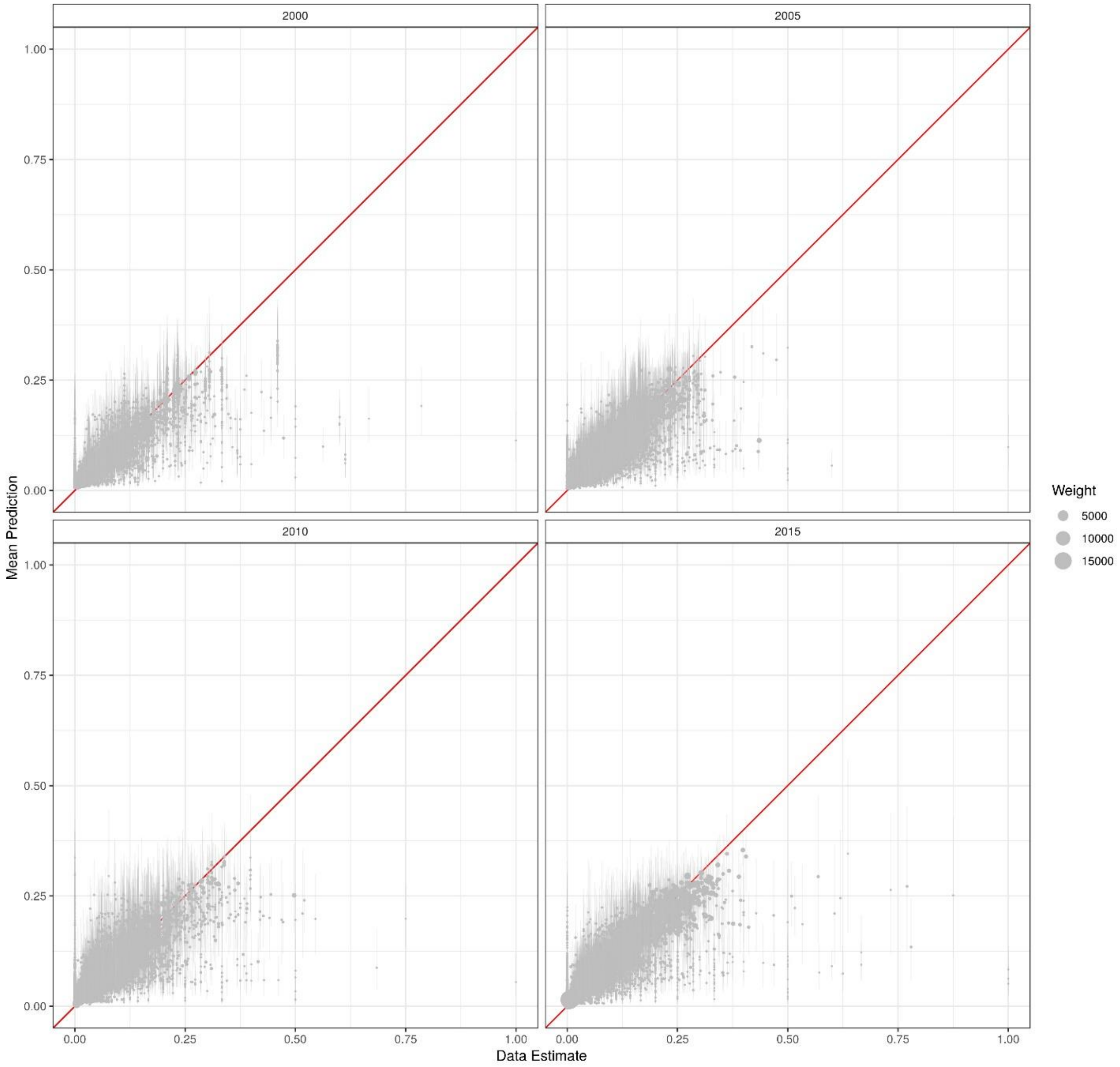

Supplementary Figure 34: Wasting second-administrative-level (Admin 2) aggregation in-sample.

Comparison of in-sample wasting predictions aggregated to Admin 2 with 95% uncertainty intervals plotted against Admin 2 aggregated data observations.

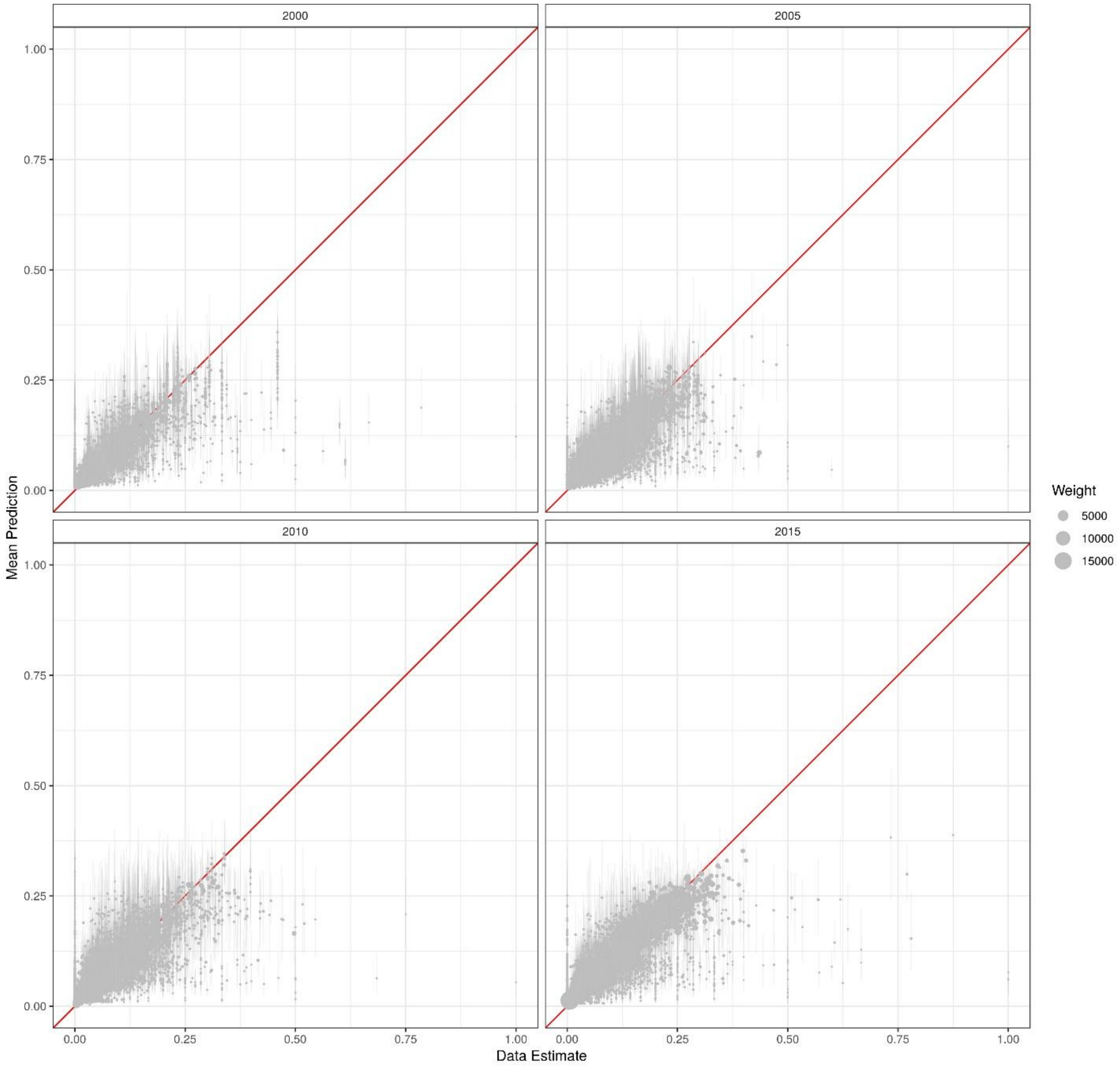

Supplementary Figure 35: Wasting second-administrative-level (Admin 2) aggregation out-of-sample.

Comparison of out-of-sample wasting predictions aggregated to Admin 2 with 95% uncertainty intervals plotted against Admin 2 aggregated data observations.

5.2.3 Underweight validation metrics

The out-of-sample (OOS) column indicates whether the metric was calculated using in-sample or out-of-sample predictions. Mean error, root-mean-squared-error (RMSE), Correlation (Corr), and coefficient of variation (Cov) are in proportion.

Supplementary Table 20: Predictive metrics for underweight aggregated to the national level (Admin 0).

Year	OOS	Median SS	Mean err.	RMSE	Corr.	95% Cov.
**2000**	FALSE	4747.000	0.001	0.010	0.997	0.953
**2005**	FALSE	5125.500	0.001	0.011	0.997	0.940
**2010**	FALSE	6447.000	-0.001	0.009	0.997	0.963
**2017**	FALSE	6118.000	0.002	0.008	0.999	0.941

Supplementary Table 21: Predictive metrics for underweight aggregated to the first administrative level (Admin 1).

Year	OOS	Median SS	Mean err.	RMSE	Corr.	95% Cov.
**2000**	FALSE	255.000	0.001	0.031	0.980	0.953
**2005**	FALSE	316.396	0.001	0.026	0.986	0.939
**2010**	FALSE	339.871	-0.001	0.021	0.986	0.963
**2017**	FALSE	351.500	0.002	0.020	0.991	0.942

Supplementary Table 22: Predictive metrics for underweight aggregated to the second administrative level (Admin 2).

Year	OOS	Median SS	Mean err.	RMSE	Corr.	95% Cov.
**2000**	FALSE	27.000	0.001	0.051	0.951	0.953
**2005**	FALSE	14.128	0.001	0.049	0.954	0.938
**2010**	FALSE	36.000	-0.001	0.042	0.951	0.963
**2017**	FALSE	44.221	0.002	0.044	0.960	0.942

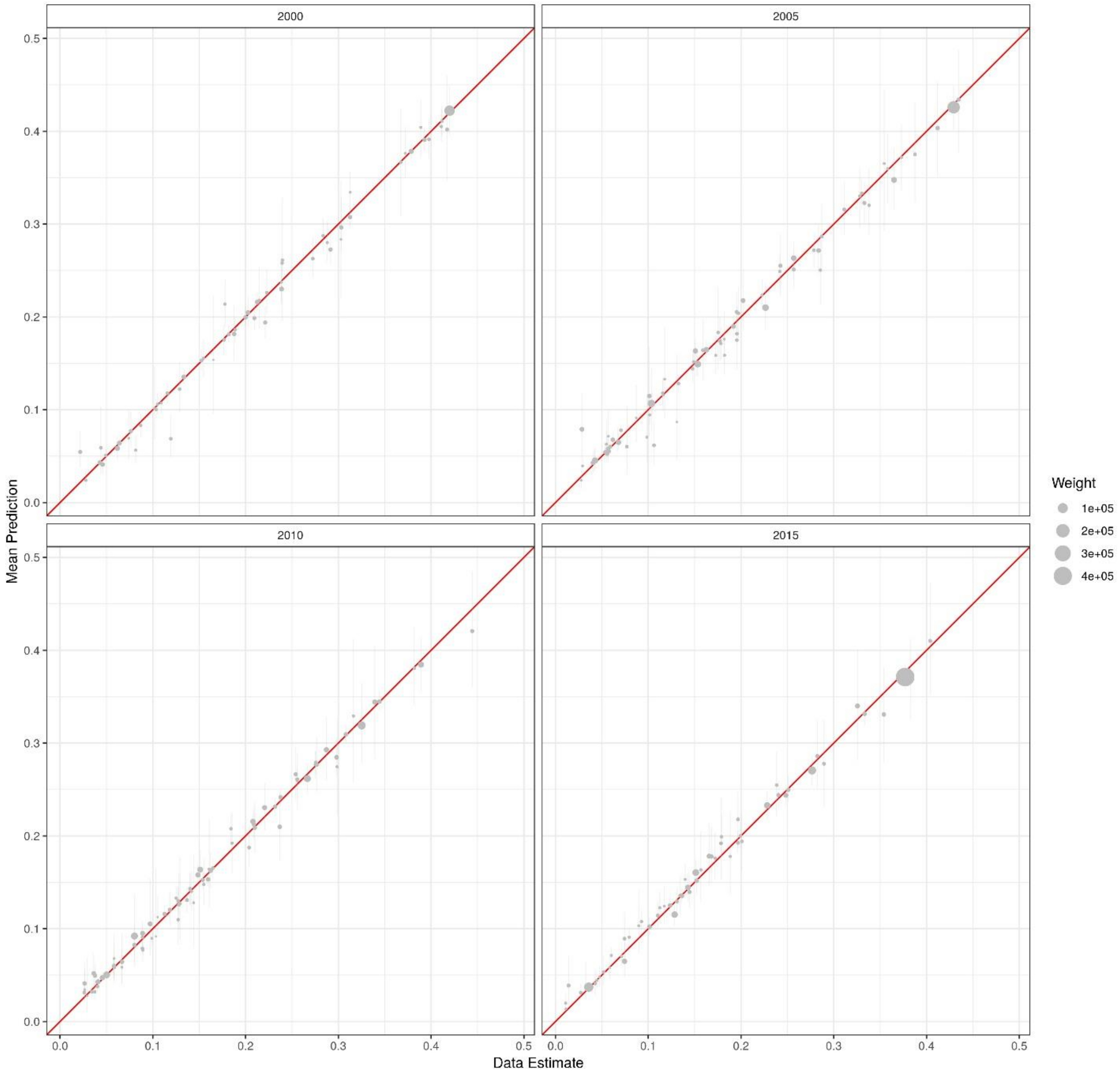

Supplementary Figure 36: Underweight national-level (Admin 0) aggregation in-sample.

Comparison of in-sample underweight predictions aggregated to Admin 0 with 95% uncertainty intervals plotted against Admin 0 aggregated data observations.

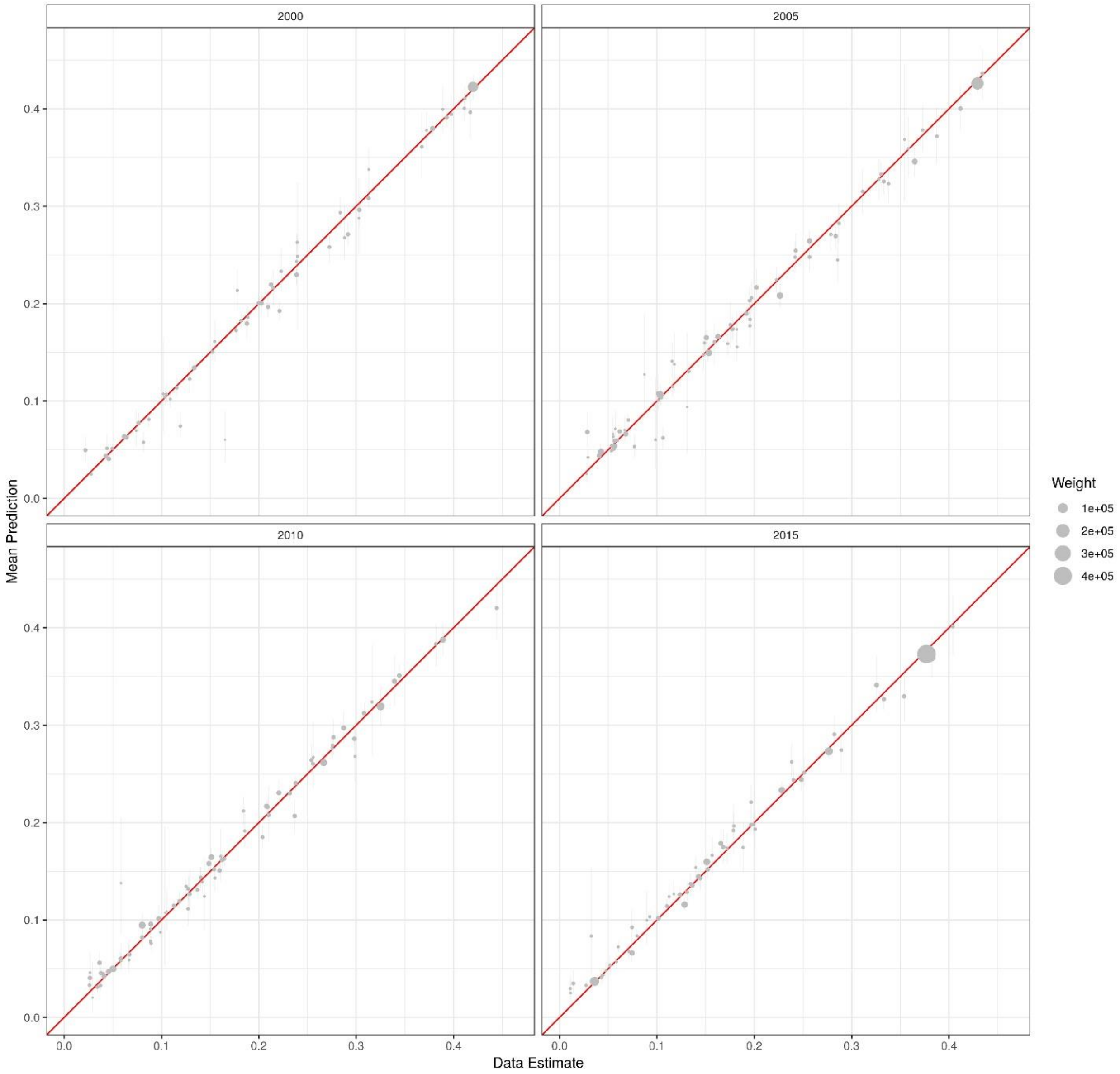

Supplementary Figure 37: Underweight national-level (Admin 0) aggregation out-of-sample.

Comparison of out-of-sample underweight predictions aggregated to Admin 0 with 95% uncertainty intervals plotted against Admin 0 aggregated data observations.

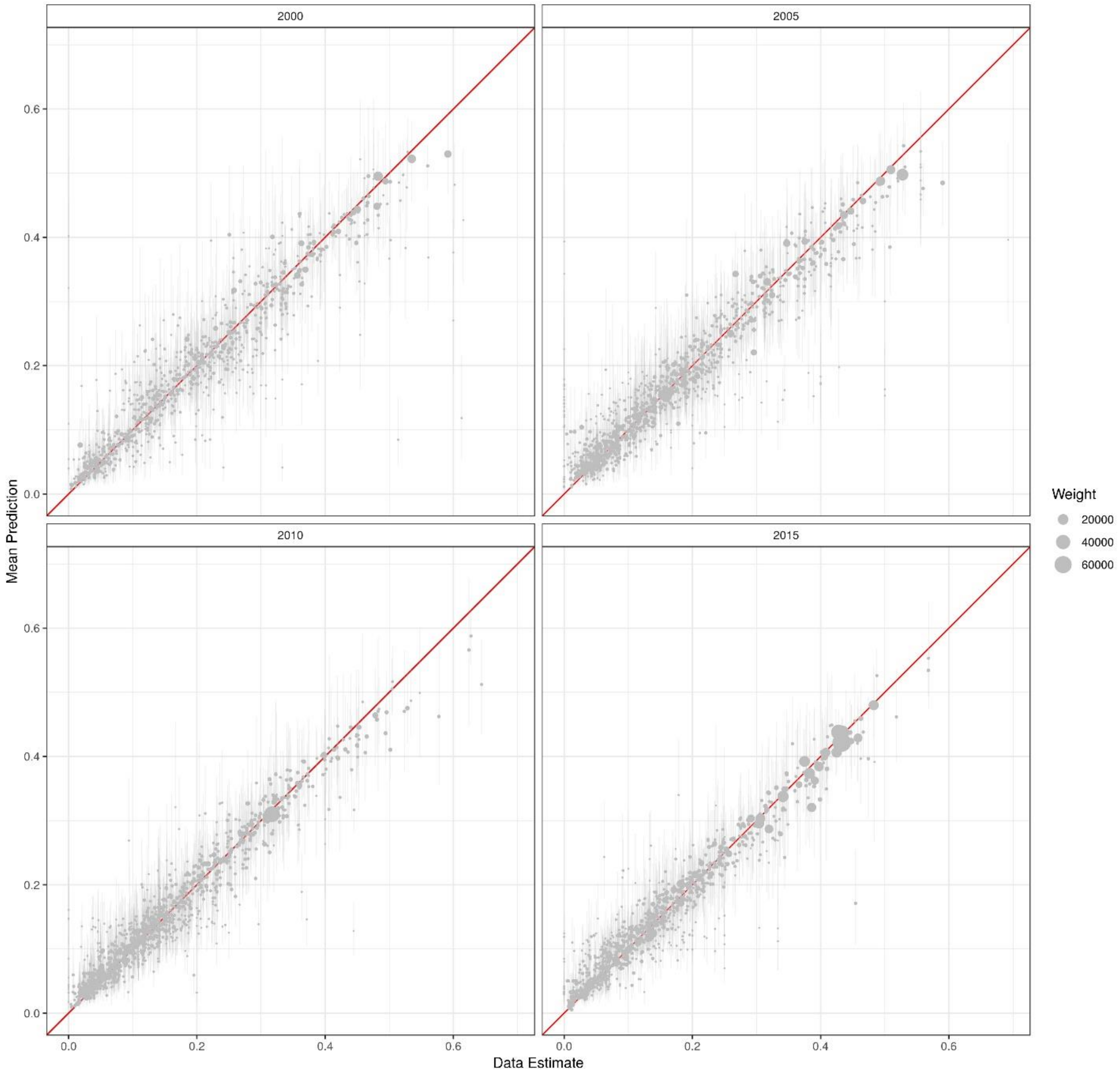

Supplementary Figure 38: Underweight first-administrative-level (Admin 1) aggregation in-sample.

Comparison of in-sample underweight predictions aggregated to Admin 1 with 95% uncertainty intervals plotted against Admin 1 aggregated data observations.

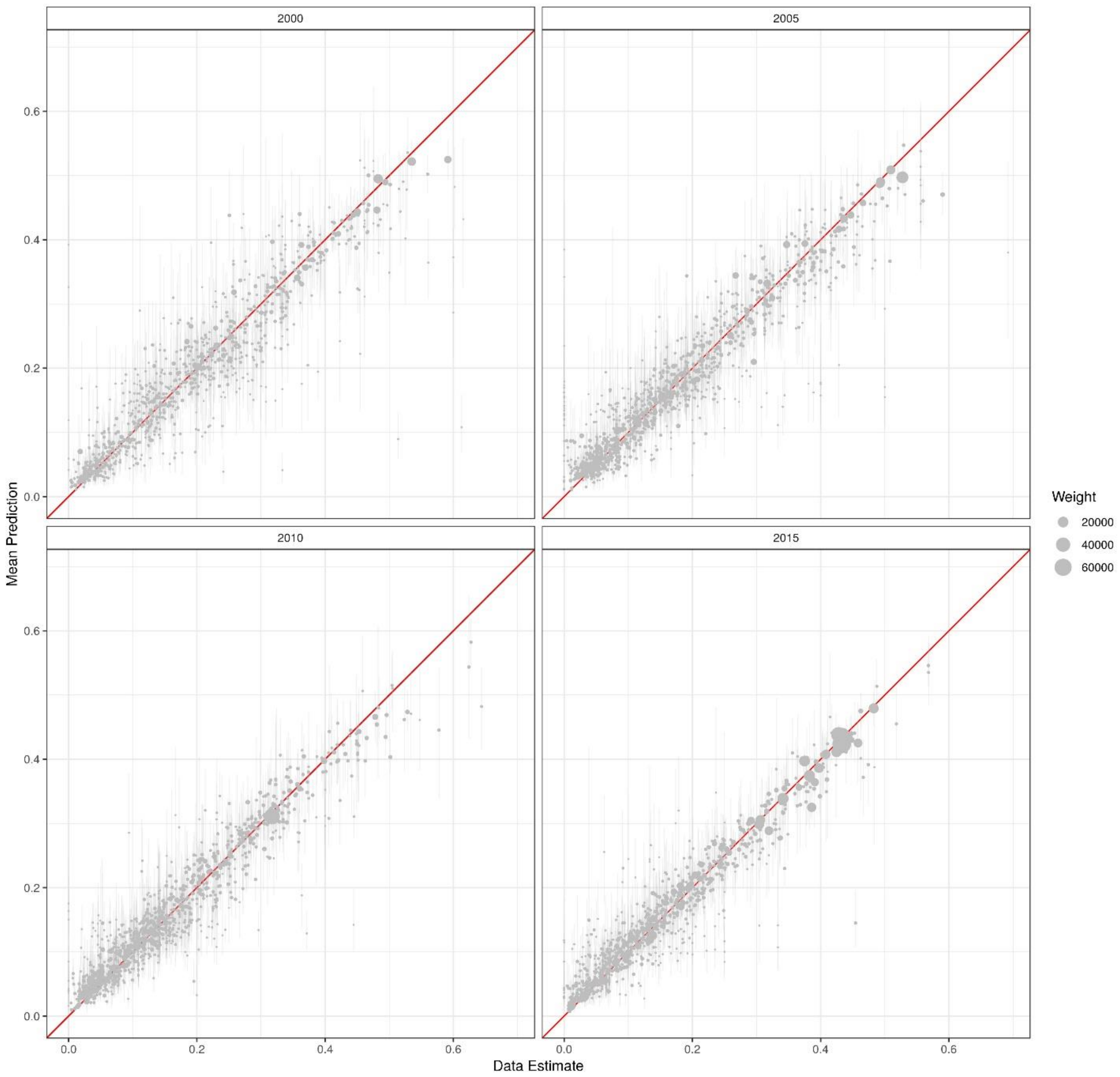

Supplementary Figure 39: Underweight first-administrative-level (Admin 1) aggregation out-of-sample.

Comparison of out-of-sample underweight predictions aggregated to Admin 1 with 95% uncertainty intervals plotted against Admin 1 aggregated data observations.

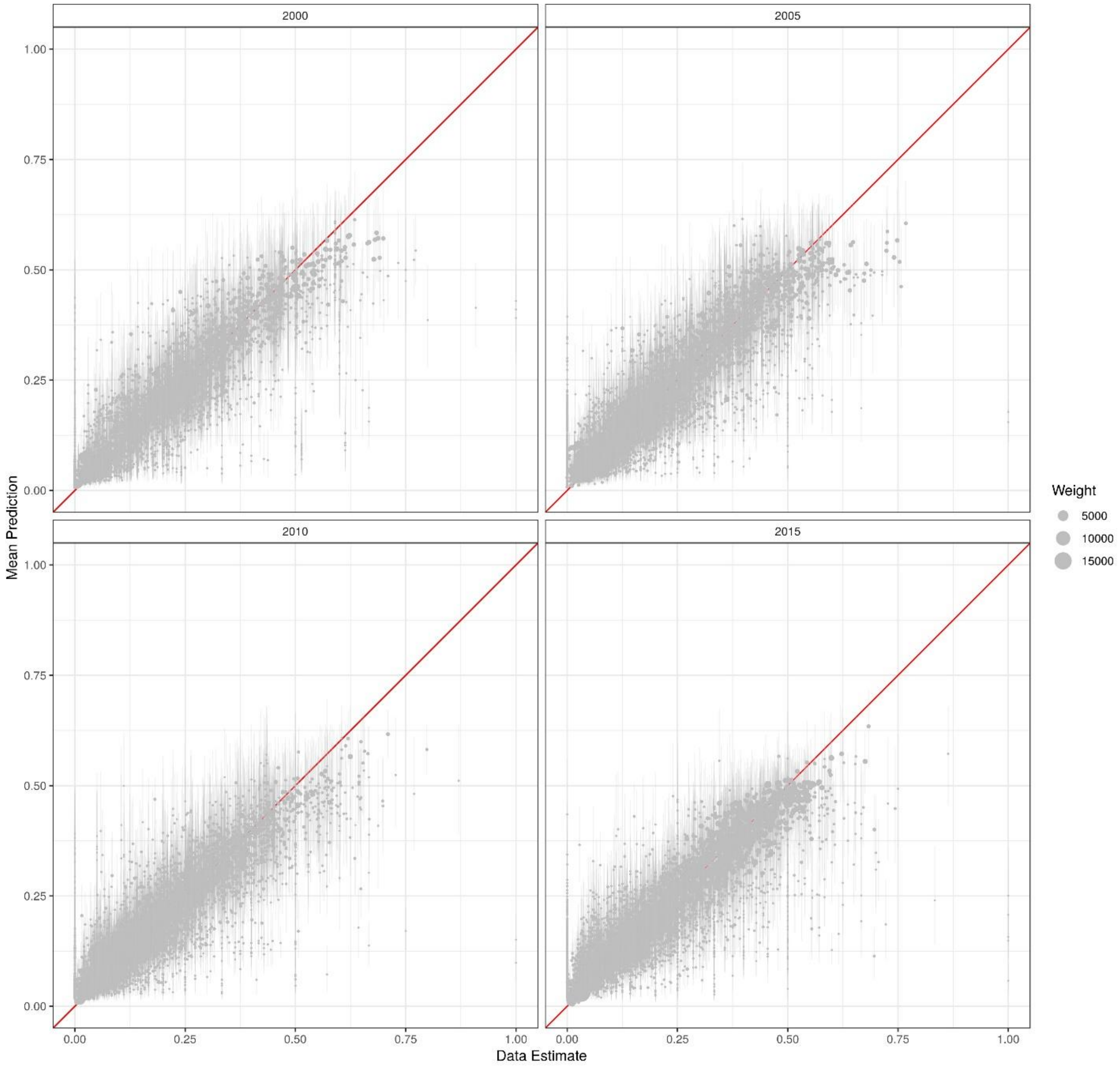

Supplementary Figure 40: Underweight second-administrative-level (Admin 2) aggregation in-sample.

Comparison of in-sample underweight predictions aggregated to Admin 2 with 95% uncertainty intervals plotted against Admin 2 aggregated data observations.

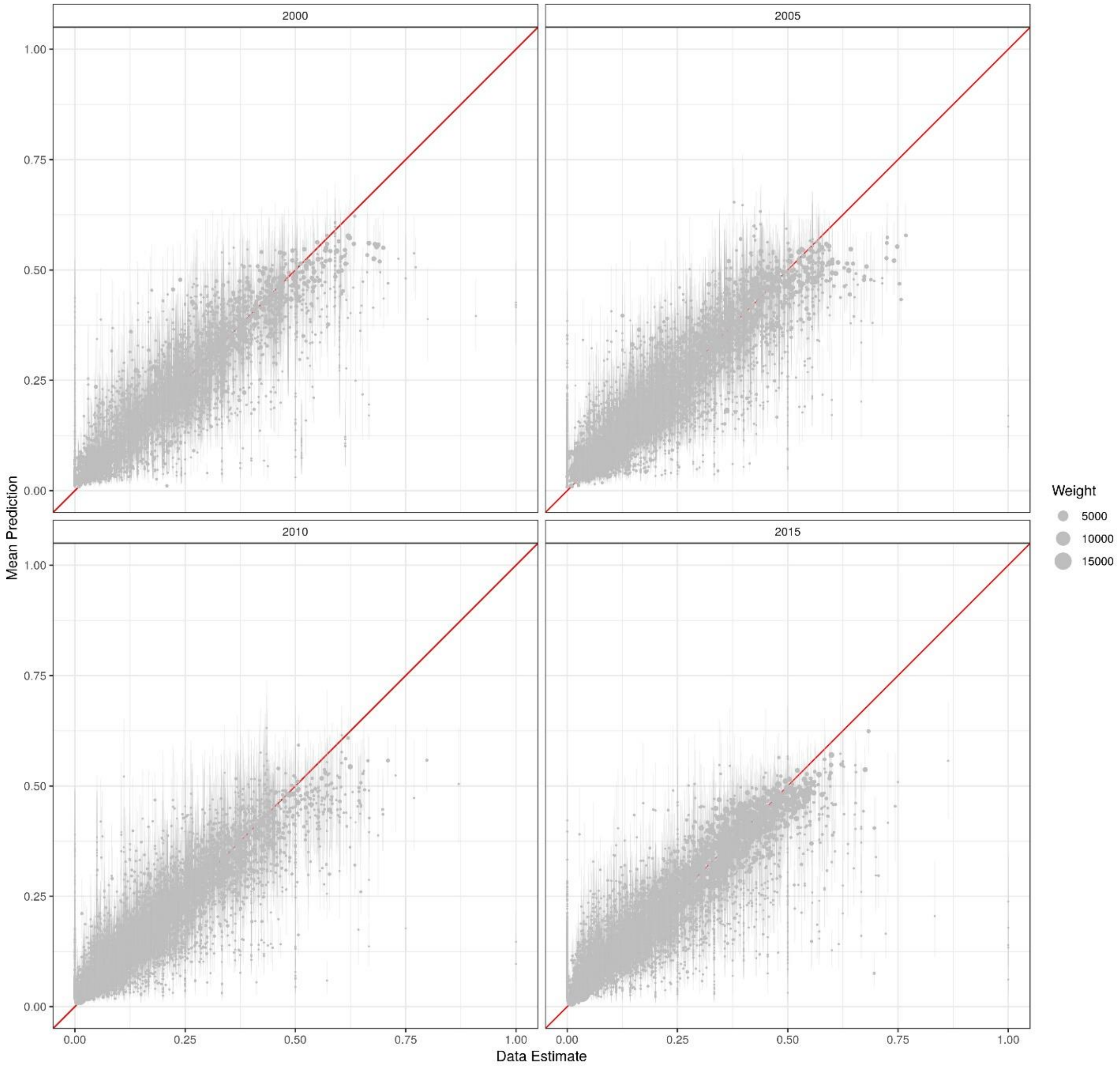

Supplementary Figure 41: Underweight second-administrative-level (Admin 2) aggregation out-of-sample.

Comparison of out-of-sample underweight predictions aggregated to Admin 2 with 95% uncertainty intervals plotted against Admin 2 aggregated data observations.

6.0 Post-estimation Calibration to National and Subnational Estimates

In order to leverage national-level data included in GBD 2017[26], but outside the scope of our current geospatial modelling framework, and to ensure perfect calibration between these estimates and GBD 2017 estimates, we performed a post hoc calibration to each of our 1,000 candidate maps. For each posterior draw, we calculated population-weighted grid cell aggregations at the level of GBD estimates (at national or subnational level) and compared these estimates in each year to the analogous and available GBD 2017 estimates from 2000 to 2017. We defined the calibration factor to be the ratio between the GBD 2017 estimates and our current estimates and linearly interpolated calibration factors in each country between the available years. Finally, we multiplied each of our grid cells in a country-year by its associated calibration factor. This ensures perfect calibration between our geospatial estimates and GBD 2017 estimates, while preserving our estimated within-country geospatial and temporal variation.

7.0 Supplementary References

1. Caulfield, L. E., Richard, S. A., Rivera, J. A., Musgrove, P. & Black, R. E. Stunting, Wasting, and Micronutrient Deficiency Disorders. in *Disease Control Priorities in Developing Countries* (eds. Jamison, D. T. et al.) (World Bank, 2006).
2. Black, R. E. Would control of childhood infectious diseases reduce malnutrition? *Acta Paediatr.* **80**, 133–140 (1991).
3. Waterlow, J. C. *et al.* The presentation and use of height and weight data for comparing the nutritional status of groups of children under the age of 10 years. 489–498 (1977).
4. WHO. *Physical status: the use and interpretation of anthropometry: report of a WHO Expert Committee*. http://apps.who.int/iris/bitstream/handle/10665/37003/WHO_TRS_854.pdf;jsessionid=2FE8F4F177D025F6433656F4F7577F3F?sequence=1 (1995).
5. WHO. *Nutrition Landscape Information System (NLIS) Country Profile Indicators: Interpretation Guide*. https://www.who.int/nutrition/nlis/en/ (2010).
6. Stewart, C. P., Iannotti, L., Dewey, K. G., Michaelsen, K. F. & Onyango, A. W. Contextualising complementary feeding in a broader framework for stunting prevention. *Matern. Child. Nutr.* **Suppl 2**, 27–45 (2013).
7. Bhutta, Z. A. *et al.* What works? Interventions for maternal and child undernutrition and survival. *Lancet* **371**, 417–440 (2008).
8. *2017 Global Nutrition Report: Nourishing the SDGs*. http://globalnutritionreport.org/wp-content/uploads/2017/11/Report_2017-2.pdf (2017).
9. Marini, A., Rokx, C. & Gallagher, P. *Standing Tall: Peru's Success in Overcoming its Stunting Crisis*. http://documents.worldbank.org/curated/en/815411500045862444/pdf/FINAL-Peru-Nutrition-Book-in-English-with-Cover-October-12.pdf (2017).
10. National Institution for Transforming India (NITI Aayog); Government of India. Nourishing India: National Nutrition Strategy. http://niti.gov.in/writereaddata/files/document_publication/Nutrition_Strategy_Booklet.pdf (2017).
11. Government of India, Department of Women and Child Development, Ministry of Human Resource Development. National Nutrition Policy. http://wcd.nic.in/sites/default/files/nnp_0.pdf (1993).
12. Sahu, S. K. *et al.* Malnutrition among under-five children in India and strategies for control. *J. Nat. Sci. Biol. Med.* **6**, 18–23 (2015).
13. Kumar, S. & Bhawani, L. Managing child malnutrition in a drought affected district of Rajasthan--a case study. *Indian J. Public Health* **49**, 198–206 (2005).
14. Black, R. E. & Heidkamp, R. Causes of Stunting and Preventive Dietary Interventions in Pregnancy and Early Childhood. *Nestle Nutr. Inst. Workshop Ser.* **89**, 105–113 (2018).
15. Black, R. E. *et al.* Maternal and child undernutrition: global and regional exposures and health consequences. *Lancet* **371**, 243–260 (2008).
16. Neufeld, L. M. & Osemdar, S. J. M. World Nutrition Situation: Global, Regional and Country Trends in Underweight and Stunting as Indicators of Nutrition and Health of Populations. in *International Nutrition: Achieving Millennium Goals and Beyond* vol. 78 11–19 (2013).
17. Chotard, S., Mason, J. B., Oliphant, N. P., Mebrahtu, S. & Hailey, P. Fluctuations in wasting in vulnerable child populations in the Greater Horn of Africa. *Food Nutr. Bull.* **31**, S219–S233 (2010).
18. FAO, IFAD, UNICEF, WFP & WHO. *The State of Food Security and Nutrition in the World 2017. Building Resilience for Peace and Food Security*. http://www.fao.org/3/a-I7695e.pdf (2017).

19. Kinyoki, D. K. *et al.* Conflict in Somalia: impact on child undernutrition. *BMJ Glob. Health* **2**, e000262 (2017).
20. Dunn, G. The impact of the Boko Haram insurgency in Northeast Nigeria on childhood wasting: a double-difference study. *Confl. Health* **12**, (2018).
21. Akseer, N. *et al.* Geospatial inequalities and determinants of nutritional status among women and children in Afghanistan: an observational study. *Lancet Glob. Health* **6**, PE447-E459 (2018).
22. ACLED. The Armed Conflict Location and Event Data Project (ACLED). https://www.acleddata.com/dashboard/ (2017).
23. de Onis, M. *et al.* Prevalence thresholds for wasting, overweight and stunting in children under 5 years. *Public Health Nutr.* 1–5 (2018).
24. IFRC. Tackling malnutrition in Niger. https://www.ifrc.org/PageFiles/113731/Tackling%20malnutrition%20in%20Niger-EN.pdf.
25. *2018 Global Nutrition Report: Shining a light to spur action on nutrition*. https://globalnutritionreport.org/reports/global-nutrition-report-2018/ (2018).
26. Dicker, D. *et al.* Global, regional, and national age-sex-specific mortality and life expectancy, 1950–2017: a systematic analysis for the Global Burden of Disease Study 2017. *Lancet* **392**, 1684–1735 (2018).
27. Osgood-Zimmerman, A. *et al.* Mapping child growth failure in Africa between 2000 and 2015. *Nature* **555**, 41–47 (2018).
28. WHO Multicentre Growth Reference Study Group. WHO Child Growth Standards based on length/height, weight and age. *Acta Paediatr.* **95**, 76–85 (2006).
29. WHO & UNICEF. *WHO Child Growth Standards and the Identification of Severe Acute Malnutrition in Infants and Children: A Joint Statement*. http://www.who.int/nutrition/publications/severemalnutrition/9789241598163/en/ (2009).
30. Wang, Y. & Chen, H.-J. Use of percentiles and z-scores in anthropometry. in *Handbook of Anthropometry* (ed. Preedy, V. R.) 29–48 (Springer New York, 2012).
31. Bhatt, S. *et al.* Improved prediction accuracy for disease risk mapping using Gaussian process stacked generalization. *J. R. Soc. Interface* **14**, 20170520 (2017).
32. Elith, J., Leathwick, J. R. & Hastie, T. A working guide to boosted regression trees. *J. Anim. Ecol.* **77**, 802–813 (2008).
33. Leathwick, J. R., Elith, J., Francis, M. P., Hastie, T. & Taylor, P. Variation in demersal fish species richness in the oceans surrounding New Zealand: an analysis using boosted regression trees. *Mar. Ecol. Prog. Ser.* **321**, 267–281 (2006).
34. Murray, C. J. L. *et al.* GBD 2010: design, definitions, and metrics. *Lancet* **380**, 2063–2066 (2012).
35. Stein, M. L. *Interpolation of Spatial Data*. (Springer New York, 1999).
36. Lindgren, F. & Rue, H. Bayesian Spatial Modelling with R-INLA. *J. Stat. Softw.* **63**, (2015).
37. Lindgren, F., Rue, H. & Lindström, J. An explicit link between Gaussian fields and Gaussian Markov random fields: the stochastic partial differential equation approach. *J. R. Stat. Soc. Ser. B Stat. Methodol.* **73**, 423–498 (2011).
38. Rozanov, Y. A. *Markov Random Fields*. (Springer-Verlag, 1982).
39. Whittle, P. On Stationary Processes in the Plane. *Biometrika* **41**, 434–449 (1954).
40. Hotez, P. J. & Ribeiro, P. J. *Model-based Geostatistics*. (Springer New York, 2007). doi:10.1007/978-0-387-48536-2.
41. Rue, H., Martino, S. & Chopin, N. Approximate Bayesian inference for latent Gaussian models by using integrated nested Laplace approximations. *J. R. Stat. Soc. Ser. B Stat. Methodol.* **71**, 319–392 (2009).
42. Martins, T. G., Simpson, D., Lindgren, F. & Rue, H. Bayesian computing with INLA: new features. *Comput Stat Data Anal* **67**, 68–83 (2013).

43. Golding, N. *et al.* Mapping under-5 and neonatal mortality in Africa, 2000–15: a baseline analysis for the Sustainable Development Goals. *Lancet* **390**, 2171–2182 (2017).
44. Cameletti, M., Lindgren, F., Simpson, D. & Rue, H. Spatio-temporal modeling of particulate matter concentration through the SPDE approach. *AStA Adv. Stat. Anal.* **97**, 109–131 (2013).
45. Alegana, V. A. *et al.* Fine resolution mapping of population age-structures for health and development applications. *J. R. Soceity Interface* **12**, 20150073 (2015).
46. Kinyoki, D. K. *et al.* Assessing comorbidity and correlates of wasting and stunting among children in Somalia using cross-sectional household surveys: 2007 to 2010. *BMJ Open* **6**, e009854 (2016).
47. Patil, A. P., Gething, P. W., Piel, F. B. & Hay, S. I. Bayesian geostatistics in health cartography: the perspective of malaria. *Trends Parasitol.* **27**, 246–253 (2011).
48. Graetz, N. *et al.* Mapping local variation in educational attainment across Africa. *Nature* **555**, 48 (2018).
49. Wood, S. N. Package 'mgcv'. http://cran.r-project.org/web/packages/mgcv/mgcv.pdf (2019).
50. Bhatt, S. *et al.* The effect of malaria control on Plasmodium falciparum in Africa between 2000 and 2015. *Nature* **526**, 207–211 (2015).
51. WorldPop. WorldPop dataset. http://www.worldpop.org.uk/data/get_data/ (2017).
52. Tatem, A. J. WorldPop, open data for spatial demography. *Sci. Data* **4**, 170004 (2017).
53. GeoNetwork. The Global Administrative Unit Layers (GAUL). http://www.fao.org/geonetwork/srv/en/main.home (2015).
54. Global Administrative Areas (GADM). GADM database of global administrative areas. http://www.gadm.org (2018).
55. Land Processes Distributed Active Archive Center (LPDAAC). Combined MODIS 5.1 dataset. *MCD12Q1 | LP DAAC :: NASA Land Data Products and Services*.
56. Lehner, B. & Döll, P. Development and validation of a global database of lakes, reservoirs and wetlands. *J. Hydrol.* **296**, 1–22 (2004).
57. World Wildlife Fund & Center for Environmental Systems Research, University of Kassel, Germany. Global Lakes and Wetlands Database, Level 3. https://www.worldwildlife.org/pages/global-lakes-and-wetlands-database (2004).
58. Roberts, D. R. *et al.* Cross-validation strategies for data with temporal, spatial, hierarchical, or phylogenetic structure. *Ecography* **40**, 913–929 (2017).

8.0 Author Contributions

Managing the estimation or publications process

Damaris K Kinyoki, Lauren E Schaeffer, Laurie B Marczak, Megan F Schipp, Nicholas J Kassebaum, and Simon I Hay.

Writing the first draft of the manuscript

Damaris K Kinyoki, Lauren E Schaeffer, Laurie B Marczak, Megan F Schipp, and Simon I Hay.

Primary responsibility for this manuscript focused on: applying analytical methods to produce estimates

Damaris K Kinyoki, Aaron E Osgood-Zimmerman, Michael Collison, Nathaniel J Henry, and Natalia V Bhattacharjee.

Primary responsibility for this manuscript focused on: seeking, cataloguing, extracting, or cleaning data; designing or coding figures and tables

Damaris K Kinyoki, Brandon V Pickering, Alice Lazzar-Atwood, and Lucas Earl.

Managing the overall research enterprise

Laurie B Marczak, Lalit Dandona, Tsegaye Gebrehiwot, Aubrey J Levine, George Mensah, Ali Mokdad, Mohsen Naghavi, Benn Sartorius, Nicholas J Kassebaum, and Simon I Hay.

Providing data or critical feedback on data sources

Damaris K Kinyoki, Fares Alahdab, Mehran Alijanzadeh, Khalid Altirkawi, Catalina Liliana Andrei, Jalal Arabloo, Olatunde Aremu, Bahram Armoon, Marcel Ausloos, Marco Avila, Ashish Awasthi, Beatriz Paulina Ayala Quintanilla, Samad Azari, Neeraj Bedi, Bayu Begashaw Bekele, Boris Bikbov, Somayeh Bohlouli, Gabrielle Britton, Roy Burstein, Carlos Castañeda-Orjuela, Lucia Cuevas-Nasu, Lalit Dandona, Rakhi Dandona, Aso Darwesh, Rajat Das Gupta, Jan-Walter De Neve, Kebede Deribe, Manisha Dubey, Lucas Earl, Andem Effiong, Maysaa El Sayed Zaki, Anwar Faraj, Mohammad Fareed, Andre Faro, Irina Filip, Nataliya Foigt, Takeshi Fukumoto, Tsegaye Gebrehiwot, Alireza Ghajar, Sameer Gopalani, Ayman Grada, Arvin Haj-Mirzaian, Arya Haj-Mirzaian, Mario Herrero, Claudiu Herteliu, Long Hoang Nguyen, Michael Hole, Mehdi Hosseinzadeh, Guoqing Hu, Sheikh Mohammed Shariful Islam, Mihajlo Jakovljevic, Jost B Jonas, Jacek Jozwiak, Tanuj Kanchan, Manoochehr Karami, Amir Kasaeian, Yousef Khader, Ejaz Khan, Yun Jin Kim, Kewal Krishan, Barthelemy Kuate Defo, G Anil Kumar, Manasi Kumar, Sheetal D Lad, Huong Lan Nguyen, Paul Lee, Chi Linh Hoang, Reza Malekzadeh, Abdullah Mamun, Francisco Rogerlândio Martins-Melo, Anthony Masaka, Benjamin Massenburg, Benjamin Mayala, Fabiola Mejía-Rodriguez, Mulugeta Melku, Walter Mendoza, Tomasz Miazgowski, Erkin Mirrakhimov, Babak Moazen, Shafiu Mohammed, Ali Mokdad, Maziar Moradi-Lakeh, Seyyed Meysam Mousavi, Ulrich Mueller, Mehdi Naderi, Mohsen Naghavi, Ionut Negoi, Malihe Nourollahpour Shiadeh, Felix Ogbo, Andrew T Olagunju, Bolajoko Olusanya, Jacob Olusanya, Eduardo Ortiz-Panozo, Mahesh P A, Adrian Pana, Anamika Pandey, Sanghamitra Pati, Ellen G. Piwoz, Akram Pourshams, Hedley Quintana, Amir Radfar, Alireza Rafiei,

Fatemeh Rajati, David Rawaf, Salman Rawaf, Rahul Rawat, Carlos Rios-González, Leonardo Roever, Ali Rostami, Siamak Sabour, Nasir Salam, Abdallah M Samy, Benn Sartorius, Brijesh Sathian, David C Schwebel, Sadaf Sepanlou, Masood Ali Shaikh, Teresa Shamah-Levy, Diego Augusto Santos Silva, Dhirendra Narain Sinha, Agus Sudaryanto, Mohamad-Hani Temsah, Abdullah Terkawi, Zemenu Tessema, Andrew Thorne-Lyman, Marcos Roberto Tovani-Palone, Bach Tran, Irfan Ullah, Olalekan Uthman, Masoud Vaezghasemi, Pascual Valdez, Vasiliy Vlassov, Yasir Waheed, Gelin Xu, Tomohide Yamada, Naohiro Yonemoto, Mustafa Younis, Chuanhua Yu, Yunquan Zhang, Nicholas J Kassebaum, and Simon I Hay.

Development of methods or computational machinery

Damaris K Kinyoki, Michael Collison, Suleman Atique, Samad Azari, Natalia Bhattacharjee, Roy Burstein, Aso Darwesh, Aniruddha Deshpande, Lucas Earl, Anwar Faraj, Mehdi Hosseinzadeh, Ali Mokdad, Jonathan Mosser, Carlos Rios-González, Abdallah M Samy, Zemenu Tessema, John VanderHeide, Nicholas J Kassebaum, and Simon I Hay.

Providing critical feedback on methods or results

Damaris K Kinyoki, Lauren E Schaeffer, Michael Collison, Zegeye Abebe, Victor Adekanmbi, Keivan Ahmadi, Fares Alahdab, Mehran Alijanzadeh, Vahid Alipour, Rajaa Al-Raddadi, Khalid Altirkawi, Catalina Liliana Andrei, Carl Abelardo T Antonio, Jalal Arabloo, Olatunde Aremu, Suleman Atique, Marcel Ausloos, Ashish Awasthi, Beatriz Paulina Ayala Quintanilla, Samad Azari, Alaa Badawi, Kaleab Baye, Bayu Begashaw Bekele, Michelle Bell, Natalia Bhattacharjee, Krittika Bhattacharyya, Boris Bikbov, Somayeh Bohlouli, Gabrielle Britton, Roy Burstein, Zahid Butt, Ester Cerin, Dinh Toi Chu, Michael A Cork, Lalit Dandona, Rakhi Dandona, Farah Daoud, Aso Darwesh, Rajat Das Gupta, Diego De Leo, Jan-Walter De Neve, Kebede Deribe, Beruk Desalegn, Aniruddha Deshpande, Melaku Desta, Daniel Diaz, Manisha Dubey, Laura Dwyer-Lindgren, Andem Effiong, Maysaa El Sayed Zaki, Maha El Tantawi, Anwar Faraj, Mohammad Fareed, Andre Faro, Seyed-Mohammad Fereshtehnejad, Irina Filip, Florian Fischer, Nataliya Foigt, Morenike Folayan, Takeshi Fukumoto, Tsegaye Gebrehiwot, Kebede Embaye Gezae, Alireza Ghajar, Paramjit Gill, Philimon Gona, Sameer Gopalani, Ayman Grada, Yuming Guo, Arvin Haj-Mirzaian, Arya Haj-Mirzaian, Samer Hamidi, Bernardo Hernandez, Claudiu Herteliu, Long Hoang Nguyen, Michael Hole, Naznin Hossain, Mehdi Hosseinzadeh, Guoqing Hu, Sheikh Mohammed Shariful Islam, Mihajlo Jakovljevic, Ravi Prakash Jha, Jost B Jonas, Jacek Jozwiak, Amaha Kahsay, Tanuj Kanchan, Manoochehr Karami, Amir Kasaeian, Yousef Khader, Ejaz Khan, Mona Khater, Yun Jin Kim, Ruth Kimokoti, Sonali Kochhar, Hamidreza Komaki, Ai Koyanagi, Kewal Krishan, Barthelemy Kuate Defo, G Anil Kumar, Manasi Kumar, Sheetal D Lad, Huong Lan Nguyen, Qiangyi Li, Shanshan Li, Chi Linh Hoang, Rakesh Lodha, Marek Majdan, Reza Malekzadeh, Abdullah Mamun, Mohammad Ali Mansournia, Francisco Rogerlândio Martins-Melo, Anthony Masaka, Benjamin Massenburg, Benjamin Mayala, Fabiola Mejía-Rodriguez, Mulugeta Melku, Walter Mendoza, George Mensah, Tomasz Miazgowski, Ted R Miller, GK Mini, Erkin Mirrakhimov, Babak Moazen, Shafiu Mohammed, Farnam Mohebi, Ali Mokdad, Yoshan Moodley, Ghobad Moradi, Maziar Moradi-Lakeh, Paula Moraga, Shane Morrison, Jonathan Mosser, Seyyed Meysam Mousavi, Ulrich Mueller, Ghulam Mustafa, Mehdi Naderi, Mohsen Naghavi, Farid Najafi, Duduzile Ndwandwe, Ionut Negoi, Jing Nie, Chukwudi Nnaji, Malihe Nourollahpour Shiadeh, Peter

Nyasulu, Felix Ogbo, Andrew T Olagunju, Bolajoko Olusanya, Jacob Olusanya, Eduardo Ortiz-Panozo, Stanislav Otstavnov, Mahesh P A, Adrian Pana, Anamika Pandey, Sanghamitra Pati, George Patton, Norberto Perico, David Pigott, Meghdad Pirsaheb, Ellen G. Piwoz, Hedley Quintana, Amir Radfar, Alireza Rafiei, Vafa Rahimi-Movaghar, Rajesh Kumar Rai, David Rawaf, Salman Rawaf, Rahul Rawat, Giuseppe Remuzzi, Andre Renzaho, Carlos Rios-González, Leonardo Roever, Jennifer Ross, Ali Rostami, Siamak Sabour, Yahya Safari, Roya Safari-Faramani, Amirhossein Sahebkar, Payman Salamati, Yahya Salimi, Hamideh Salimzadeh, Abdallah M Samy, Benn Sartorius, Brijesh Sathian, David C Schwebel, Sadaf Sepanlou, Masood Ali Shaikh, Kiomars Sharafi, Rajesh Sharma, Aziz Sheikh, Jasvinder Singh, Dhirendra Narain Sinha, Agus Sudaryanto, Muawiyyah Babale Sufiyan, Rafael Tabarés-Seisdedos, Birkneh Tilahun Tadesse, Mohamad-Hani Temsah, Zemenu Tessema, Andrew Thorne-Lyman, Marcos Roberto Tovani-Palone, Bach Tran, Irfan Ullah, Olalekan Uthman, Masoud Vaezghasemi, Pascual Valdez, Yousef Veisani, Vasiliy Vlassov, Yasir Waheed, Yafeng Wang, Yuan-Pang Wang, Gelin Xu, Tomohide Yamada, Engida Yisma, Naohiro Yonemoto, Mustafa Younis, Chuanhua Yu, Telma Zahirian Moghadam, Sojib Bin Zaman, Mohammad Zamani, Yunquan Zhang, Nicholas J Kassebaum, and Simon I Hay.

Drafting the manuscript or revising is critically for important intellectual content

Damaris K Kinyoki, Lauren E Schaeffer, Victor Adekanmbi, Fares Alahdab, Catalina Liliana Andrei, Carl Abelardo T Antonio, Jalal Arabloo, Olatunde Aremu, Mehran Asadi, Marcel Ausloos, Ashish Awasthi, Quique Bassat, Kaleab Baye, Neeraj Bedi, Michelle Bell, Krittika Bhattacharyya, Zahid Butt, Carlos Castañeda-Orjuela, Ester Cerin, Dinh Toi Chu, Aso Darwesh, Rajat Das Gupta, Jan-Walter De Neve, Kebede Deribe, Beruk Desalegn, Melaku Desta, Daniel Diaz, Manisha Dubey, Laura Dwyer-Lindgren, Lucas Earl, Andem Effiong, Maysaa El Sayed Zaki, Maha El Tantawi, Anwar Faraj, Andre Faro, Seyed-Mohammad Fereshtehnejad, Irina Filip, Florian Fischer, Nataliya Foigt, Morenike Folayan, Takeshi Fukumoto, Tsegaye Gebrehiwot, Philimon Gona, Sameer Gopalani, Ayman Grada, Yuming Guo, Claudiu Herteliu, Long Hoang Nguyen, Michael Hole, Naznin Hossain, Mehdi Hosseinzadeh, Sheikh Mohammed Shariful Islam, Mihajlo Jakovljevic, Ravi Prakash Jha, Jost B Jonas, Jacek Jozwiak, Tanuj Kanchan, Manoochehr Karami, Amir Kasaeian, Yousef Khader, Ejaz Khan, Mona Khater, Yun Jin Kim, Hamidreza Komaki, Ai Koyanagi, Barthelemy Kuate Defo, Manasi Kumar, Huong Lan Nguyen, Paul Lee, Shanshan Li, Chi Linh Hoang, Shai Linn, Abdullah Mamun, Francisco Rogerlândio Martins-Melo, Anthony Masaka, Benjamin Massenburg, Mulugeta Melku, Walter Mendoza, Tomasz Miazgowski, Ted R Miller, Babak Moazen, Shafiu Mohammed, Farnam Mohebi, Ali Mokdad, Ghobad Moradi, Maziar Moradi-Lakeh, Paula Moraga, Shane Morrison, Jonathan Mosser, Mehdi Naderi, Ionut Negoi, Chukwudi Nnaji, Felix Ogbo, Andrew T Olagunju, Bolajoko Olusanya, Jacob Olusanya, Eduardo Ortiz-Panozo, Mahesh P A, Adrian Pana, Sanghamitra Pati, George Patton, David Pigott, Ellen G. Piwoz, Hedley Quintana, Amir Radfar, Alireza Rafiei, Vafa Rahimi-Movaghar, Rahul Rawat, Andre Renzaho, Carlos Rios-González, Leonardo Roever, Jennifer Ross, Siamak Sabour, Mahdi Safdarian, Nasir Salam, Hamideh Salimzadeh, Abdallah M Samy, Megan Schipp, David C Schwebel, Masood Ali Shaikh, Aziz Sheikh, Diego Augusto Santos Silva, Jasvinder Singh, Dhirendra Narain Sinha, Muawiyyah Babale Sufiyan, Mohamad-Hani Temsah, Zemenu Tessema, Marcos Roberto Tovani-Palone, Bach Tran, Khanh Bao Tran, Irfan Ullah, Olalekan Uthman, Masoud Vaezghasemi, Francesco S Violante, Vasiliy Vlassov, Yuan-Pang Wang, Gelin Xu, Mustafa Younis, Sojib Bin Zaman, Nicholas J Kassebaum, and Simon I Hay.
